# Regioselectivity of the Reaction between β‑Enamino Diketones and Methylhydrazine Explained

**DOI:** 10.1021/acs.joc.5c03194

**Published:** 2026-03-14

**Authors:** Vinicius Martinelli, Isaac F. Leach, Julia Poletto, Wagner E. Richter, Fernanda A. Rosa, Rodrigo M. Pontes

**Affiliations:** † Departamento de Química, 42487 Universidade Estadual de Maringá, 87020-900 Maringá, Brasil; ‡ Dipartimento di Chimica, Biologia e Biotecnologie, Università degli Studi di Perugia, 06123 Perugia, Italy

## Abstract

The complete mechanism for the reaction of β-enamino diketone (BED) with methylhydrazine in H_2_O/MeOH and acetonitrile is elucidated via state-of-the-art quantum chemical calculations. Our results show that the mechanism branches into multiple pathways with distinct energetic profiles, leading to a product distribution governed by a delicate balance of electronic effects. The initial branching point is determined by which the nitrogen atom of the asymmetric methylhydrazine (NH_2_ or NHMe) attacks the BED *β*-carbon. Following the elimination of HNMe_2_, a cyclization step occurs, where the remaining methylhydrazine nitrogen attacks one of two distinct carbonyl carbons, leading to a product distribution, where three of four possible regioisomers are observed experimentally. The activation energy for this cyclization is influenced by the electronic properties of the substituents on both the carbonyl carbon (methoxycarbonyl or chlorophenyl) and the nucleophilic nitrogen (H or Me). Critically, this cyclization is contingent on a proton transfer from nitrogen to the carbonylic oxygen, promoted by a proton transfer catalyst (PTC). In H_2_O/MeOH, the protic solvent molecules act catalytically, whereas in acetonitrile, an acidic nitrogen center, as in methylhydrazine or dimethylamine, is required. The importance of the proton transfer catalyst is confirmed by experimentally determined product ratios of the reaction in acetonitrile with catalytic acetic acid.

## Graphical abstract

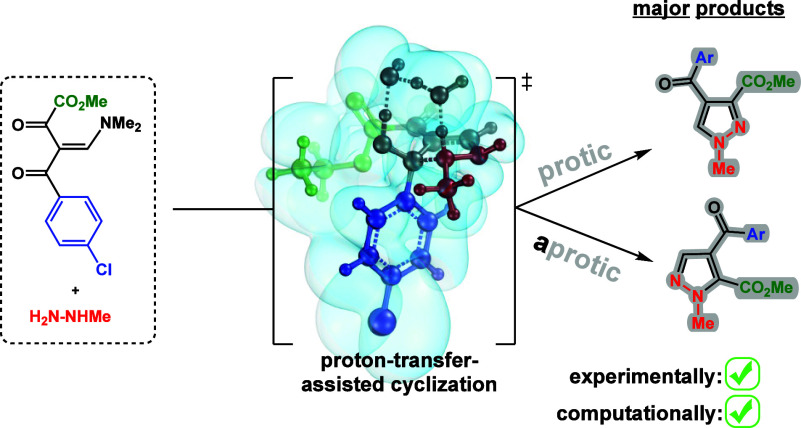

## Introduction

*β*-Enamino diketones (BEDs) represent a highly versatile class of compounds, providing synthetic access to a diverse array of heterocyclic structures, including pyrazoles,^1^ pyrimidines,^2^ pyrroles,^3^ and others^4,5^ (Scheme 1). These heterocycles serve as crucial building blocks for pharmaceutical drugs owing to their medicinal properties.^6–9^ This versatility comes from the core structure of BEDs which features at least three electrophilic centers: two carbonyl groups (often nonequivalent) and the *β*-carbon. This diverse array of reactive sites allows for extensive chemical modification, facilitating the synthesis of heterocycles with tailored substituents.^3,10^ This tunability is particularly valuable as substitution directly influences pharmaceutical activity.^6^

**Scheme 1:**
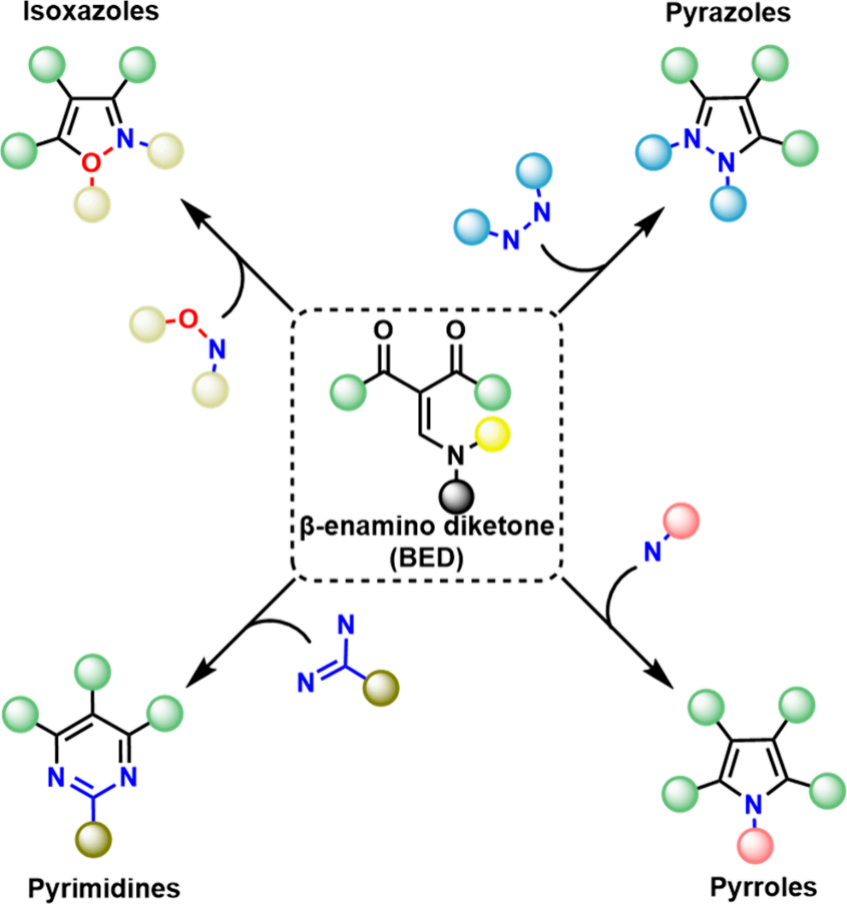
Synthesis of Heterocycles from BED and Different Nucleophiles

Although synthetically significant, the mechanisms for heterocycle formation from BEDs still lack support from comprehensive experimental data. The prevailing mechanism proposes an initial addition of a nucleophile to the *β*-carbon, followed by elimination of the amine group. Support for this step includes observed amine exchanges e.g., NMe_2_ replaced by NHPh or NH^*t*^Bu,^10,11^ mass spectrometry data,^12^ and analysis of analogous BED structures.^13–16^ The resulting intermediate then undergoes cyclization via nucleophilic attack to one of the carbonyl groups. Under specific conditions—such as in the presence of a Lewis acid e.g., BF_3_, or when a strong electron withdrawing group e.g., CF_3_ is attached to the carbonyl—the initial attack may instead occur first at the carbonyl, followed by cyclization at the second carbonyl.^10,11,17^ However, the absence of kinetic data and direct isolation/characterization of intermediates prevents definitive confirmation of the mechanism and obscures crucial details such as the identity of the rate-determining step.

The structural richness of BEDs, combined with the asymmetry of nucleophiles like monosubstituted hydrazine derivatives, leads to multiple potential sites for initial nucleophilic attack and subsequent cyclization. Consequently, reactions often produce mixtures of regioisomeric products.^18–20^ This was demonstrated in reactions of BEDs possessing asymmetrical carbonyls with methylhydrazine: the lone pairs of the two nitrogen atoms of methylhydrazine can both attack the *β*-carbon, and the two distinct carbonyls offer alternative cyclization pathways, yielding four possible products; three of which were observed experimentally (Scheme 2).^18,19^

**Scheme 2:**
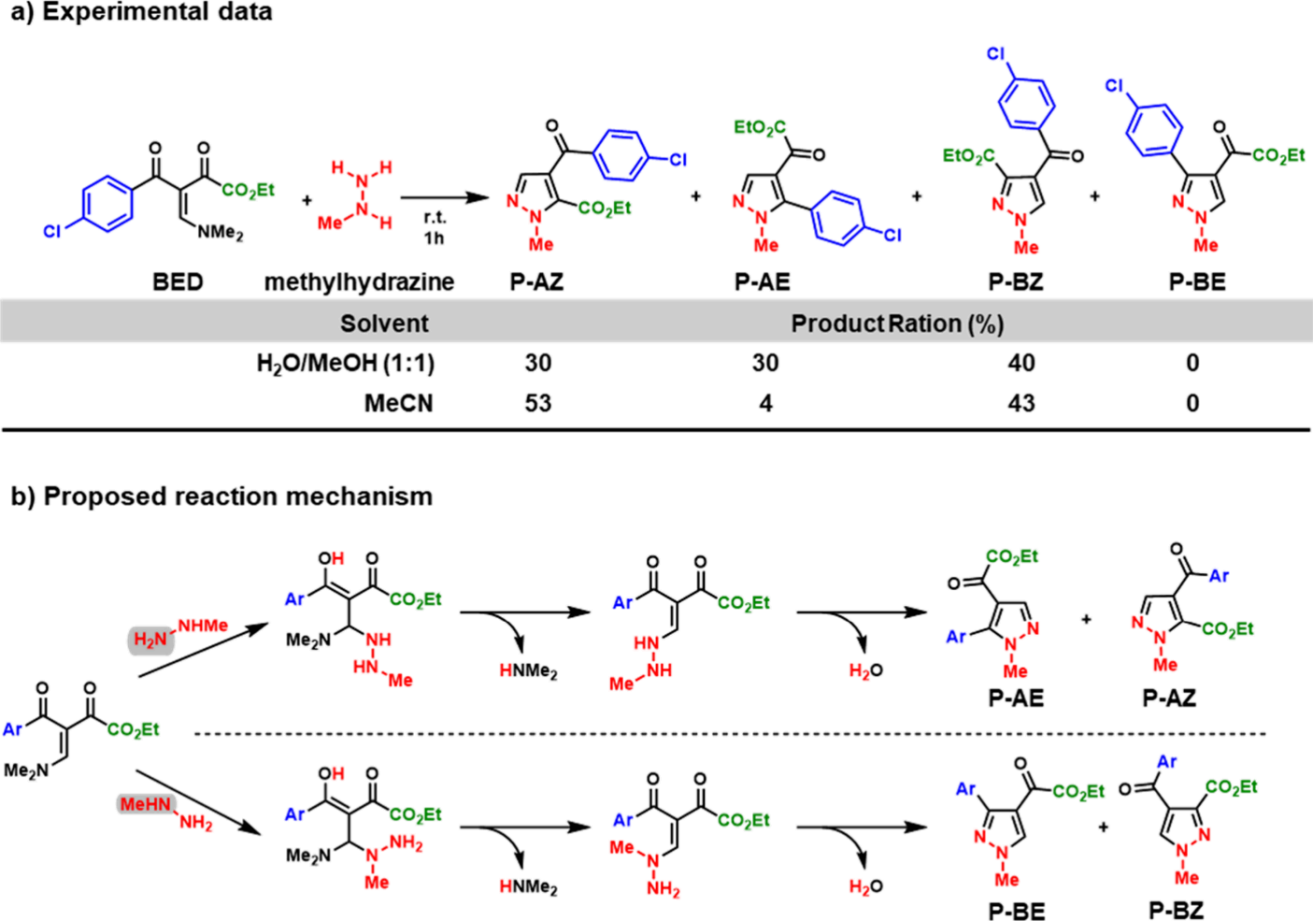
(a) Reaction between BED and Methylhydrazine in H_2_O/MeOH and MeCN and (b) Proposed Mechanistic Bifurcation Based on the Initial Nucleophilic Attack by the NH_2_ (Path A) or NHMe (Path B) Nitrogen of Methylhydrazine, Leading to Distinct Regioisomeric Products **P-AE**, **P-AZ**, **P-BZ**, and the Unobserved **P-BE** ^a^

a Reaction mechanism shows the experimentally observed product distribution. Adapted from ref 19. Copyright [2022] American Chemical Society.

The product distribution is highly sensitive to several factors: the substituents on the carbonyl groups modulate their electrophilicity, influencing cyclization site preference;^21^ the nucleophile’s nature strongly influences the initial site of attack;^18^ and, though less intuitively, the solvent properties (polarity and proticity) also profoundly impact the outcome. Shifting from polar protic to polar aprotic solvents can alter the major product or even enable selective formation of a single isomer with appropriate solvent combinations.^20^

Clearly, the product distribution and reaction pathway are governed by a delicate interplay of electronic effects. Understanding and manipulating interactions provides a powerful means to control regiochemistry in the synthesis of valuable heterocycles, and modern computational methods are well positioned to tackle such problems. Despite this, there is – to the best of our knowledge – only one study computationally investigating the electronic effects governing these reactions.^22^ In this work, Rosa and co-workers used Density Functional Theory (DFT) to probe the selectivity of the cyclization step, leaving the remaining steps – including nucleophilic addition to the *β*-carbon – untreated. The product distribution was proposed to be thermodynamically driven.^22^

In 2022, Rosa and co-workers reported the reaction between the BED methyl (Z)-3-(4-chlorobenzoyl)-4-(dimethylamino)-2-oxobut-3-enoate, hereafter BED, and methylhydrazine performed in two solvents—a H_2_O/MeOH (1:1) mixture and MeCN. The reaction yielded three distinct products under both conditions. The polar protic solvent mixture (H_2_O/MeOH) resulted in a more even product distribution, while the polar aprotic solvent (MeCN) strongly favored two of the three products (Scheme 2a).^19^ This interesting case study suggests the reaction mechanism diverges depending on which nitrogen atom of methylhydrazine attacks the *β*-carbon of the substrate, forming different intermediates that subsequently cyclize onto distinct carbonyl groups to yield the observed products (Scheme 2b).

To elucidate the origin of the product distribution, we computed the reaction mechanisms for both pathways, aiming to identify the key steps governing product formation. We employed a combination of DFT and local coupled cluster methods to provide an accurate and complete overview of the reactive potential energy surface. A variety of electronic structure analyses were used to uncover the underlying electronic interactions and driving forces behind the observed product ratio.

Our results, based on the accepted mechanism, suggest the product distribution is kinetically, not thermodynamically, controlled. The cyclization step is critical and requires proton transfer assistance. The solvent itself (H_2_O/MeOH) can facilitate this via proton shuttling, as demonstrated by calculations including explicitly modeled solvent molecules. In the aprotic solvent (MeCN), assistance likely comes from a nitrogen-containing molecule (e.g., methylhydrazine). The calculated energy barriers, consistent with the experimental selectivity, reflect a delicate balance between the electronic properties of the nucleophilic sites of methylhydrazine (NH_2_ and NHMe) and the carbonyl moieties.

## Results and Discussion

The BED molecule has two possible isomeric configurations *E* and *Z*. Our calculations demonstrate that the *E* is 1.5 kcal/mol more stable than *Z* (Figure S1). Therefore, **BED-E** is assumed throughout this work.

The generally accepted reaction mechanism begins with addition of methylhydrazine to the *β*-carbon of the **BED-E**. Methylhydrazine’s asymmetric nitrogen atoms provide two distinct reaction pathways: attack by NH_2_ (Path A) leads to products **P-AE** and **P-AZ**, while attack by NHMe (Path B) leads to **P-BE** and **P-BZ** (Scheme 2b, top and bottom, respectively). Notably, among the four possible products, only **P-BE** is unobserved experimentally. For the reaction in H_2_O/MeOH (1:1), the observed product distribution is **P-AZ:P-AE:P-BZ** = 30:30:40.

NH_2_-attack to the β-carbon (Path A), (key intermediates shown in Figure 1; see Figure S4 for the complete mechanism), has an activation barrier of 16.0 kcal/mol. This is followed by a proton transfer from the NH_2_ group to a carbonyl oxygen leading to the formation of intermediate **GS2A** in an endergonic step (12.2 kcal/mol).

**Figure 1:**
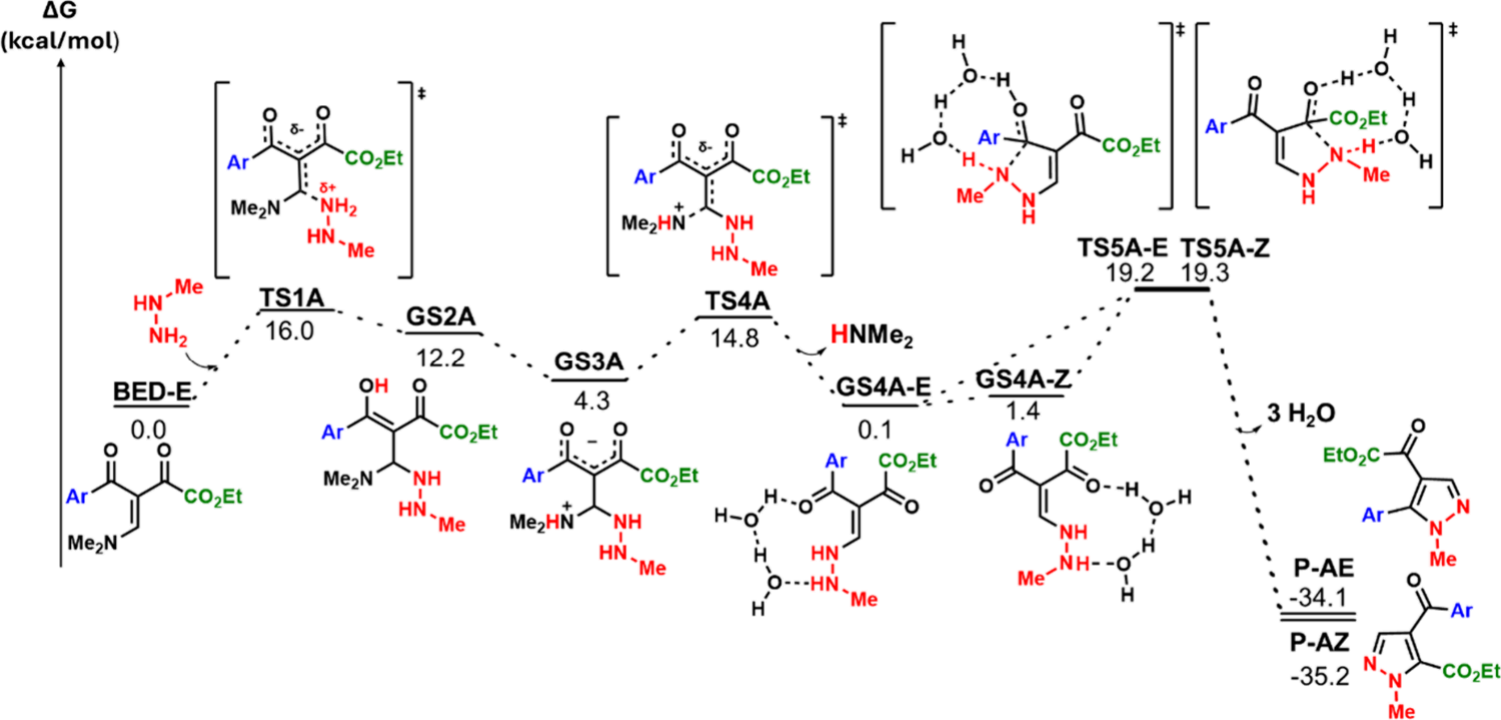
Energy profile for the reaction of the methylhydrazine (NH_2_ moiety) nucleophilic attack to the *β*-carbon (path A). Computed with ωB97X-D3­(BJ)/def2-TZVP, CPCM­(water).

Following initial nucleophilic attack, rotation of the C–C σ-bond brings the NMe_2_ group proximal to the newly formed hydroxyl group. A proton transfer from this hydroxyl to the NMe_2_ nitrogen then occurs, forming intermediate **GS3A** in an exergonic step (Δ*G* = −7.9 kcal/mol). Finally, **GS3A** undergoes elimination of HNMe_2_ via **TS4A** to yield intermediate **GS4A**. There are two possible conformations for this intermediate (**GS4A-E** and **GS4A-Z**). Our calculations suggest that isomerization of **GS4A-E** to **GS4A-Z** is a more facile process (Δ*G* ^‡^ = 13.8 kcal/mol, Figure S20a) than cyclization (Δ*G* ^‡^ = 19.1 kcal/mol). Therefore, they exist in conformational equilibrium. Cyclization of the *E*-isomer yields **P-AE**, while the *Z*-isomer yields **P-AZ**.

The cyclization transition state (**TS5**) is concerted, involving simultaneous nucleophilic addition of nitrogen to the carbonyl carbon *and* proton transfer from nitrogen to oxygen. Although our results suggest a direct proton transfer from NHMe to the carbonylic oxygen is possible, it does not seem to be a viable process at room temperature since the energy barriers are exceedingly high (between 30 and 40 kcal/mol, Figures S12 and S13). Alternatively, the proton transfer can be assisted by oxygen-based proton donors such as H_2_O and MeOH, or nitrogen-based groups such as methylhydrazine, dimethylamine or transient species participating in the mechanism such as **GS3A** or **GS4A**. For the reaction performed in H_2_O/MeOH, we modeled the proton transfer catalyst (PTC). with H_2_O molecules due to its high concentration when compared to PTCs with NH groups, such as methylhydrazine. We initially considered different numbers of explicitly modeled water molecules (1–3). We noted that the cyclization energy barriers do exhibit some dependence on the number of water molecules used (max variation of 6.6 kcal/mol is seen for TS5B-E). However, importantly, the overall trends remained the same (Figures S2–S7) and they all agree with the experimental observations. The results that most closely reproduce the experimental trends were obtained with two water molecules, and as such, this solvent model was used throughout, unless otherwise specified.

The rate-determining step is the cyclization transition state (**TS5A-E** or **TS5A-Z**, Figure 1). The energy barriers for both cyclization pathways differ by only 0.1 kcal/mol. In contrast, the thermodynamics of product formation differ by 1.1 kcal/mol (**P-AE**: −34.1 kcal/mol vs **P-AZ**: −35.2 kcal/mol). These results indicate kinetic control over product selectivity, in contrast to prior propositions of thermodynamic control.^22^

The initial steps of NHMe-attack (Path B, Figure 2, see Figure S5 for complete mechanism) mirror Path A (Figure 1): nucleophilic addition, proton transfers, conformational reorganization and HNMe_2_ elimination. However, we note some key energetic differences: nucleophilic addition to the *β*-carbon barrier (14.1 kcal/mol vs 16.0 kcal/mol in Path A) and HNMe_2_ elimination barrier (12.6 kcal/mol vs 10.5 kcal/mol in Path A).

**Figure 2:**
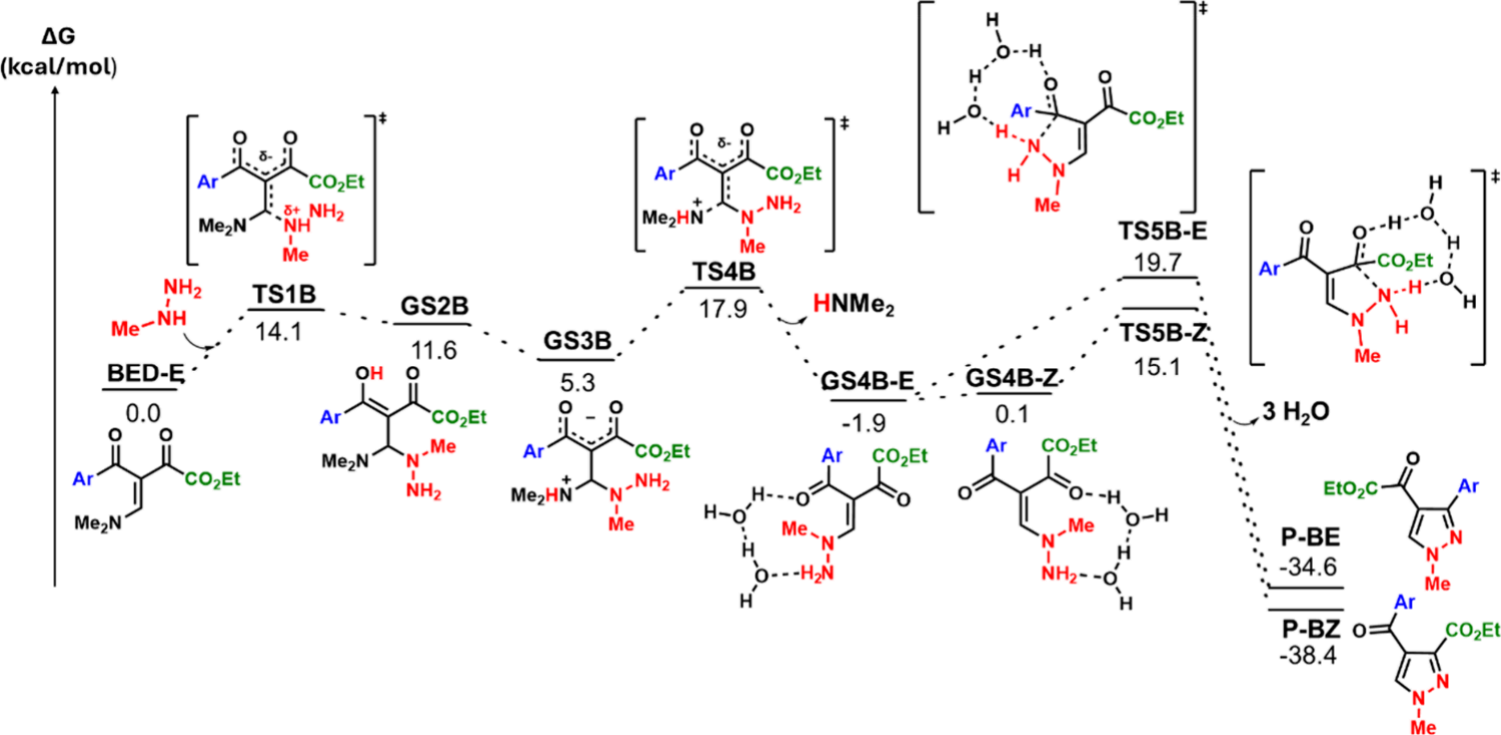
Energy profile for the reaction considering the methylhydrazine NHMe moiety attack to the *β*-carbon (path B). Computed with ωB97X-D3­(BJ)/def2-TZVP, CPCM (water).

The **GS4B** isomers follow similar stability trends to **GS4A** where the *Z* conformer is less stable than the *E* conformer (Δ*G* = +2.0 kcal/mol), but much more reactive toward cyclization (**GS4B-Z** barrier of 15.0 kcal/mol vs **GS4B-E** barrier of 21.6 kcal/mol). The isomerization of **GS4B-E** to **GS4B-Z** is also an easier process (Δ*G* ^‡^ = 10.0 kcal/mol, Figure S20b) than cyclization (Δ*G* ^‡^ = 21.6 kcal/mol). Therefore, isomerization of **GS4B-E** to **GS4B-Z** followed by cyclization is predicted to be the most efficient path, aligning well with the experimental observations of **P-BZ** as the only product formed. Notably, the low cyclization barrier for **GS4B-Z** shifts the rate-determining step to **TS4B**. This pathway has an overall energy barrier of 17.9 kcal/mol – the lowest found among all routes.

In every case, the experimental product ratios reported by Rosa and co-workers^19^ agree well with the computed barriers. The major product **P-BZ** (40%) has the lowest calculated barrier (17.9 kcal/mol). The equally abundant **P-AE** and **P-AZ** (30% each) correspond to nearly identical barriers (19.2 vs 19.3 kcal/mol). Lastly, the undetected **P-BE** exhibits both the highest barrier (19.7 kcal/mol) *and* competition with the kinetically favored **P-BZ** pathway which has a barrier 1.8 kcal/mol lower in energy. In contrast to the previously reported results,^22^ thermodynamic stabilities show poor correlation: **P-BE** is computed to be more stable than the observed **P-AE** yet remains experimentally undetected – further confirming kinetic control. We note that these differences are primarily due to the explicit modeling of the PTC, as confirmed by calculations employing our improved protocol on the same substrates initially investigated by Rosa and co-workers (Figures S37 and S38).

### Reaction of BED with Methylhydrazine in MeCN

While implicit solvent effects were modeled for both H_2_O/MeOH and MeCN (Figures 1 and 2), the initial steps (up to intermediate **GS4**) do not involve explicit solvent molecules. Consequently, the calculated energy profiles for these initial stages in acetonitrile (Figures S8 and S9) are largely comparable to those in H_2_O/MeOH.

However, the cyclization mechanism of **GS4** diverges because in the previous case, the solvent was directly involved in the proton transfer step as the PTC. This is not the case for acetonitrile. Even though trace amounts of water can exist as solvent impurity or from the dehydration step leading to product formation, nitrogenous bases e.g., methylhydrazine or dimethylamine, are more likely to act as PTC because they are present in stoichiometric concentrations. Since several of our attempts to optimize the transition states with methylhydrazine acting as PTC were unsuccessful, and given the presence of multiple acidic nitrogen centers, we chose to model this step using NH_3_ as a proxy for the PTC. It is worth noting that unlike the case with H_2_O as PTC, these NH bearing molecules are not present in large excess. Therefore, the probability of having more than one molecule acting as PTC is low. Thus, we considered only one NH_3_ molecule catalyzing the proton transfer.

When analyzing the energy profile for the cyclization step (Figure 3a) and the electron density of the bond critical points (BCP) for the breaking N–H and the forming O–H bonds (Figure 3b), we note that NH_3_ results in a much earlier NH deprotonation, evidenced by the earlier decrease of the N–H BCP electron density, compared to the system with H_2_O as PTC. In contrast, the protonation of the carbonylic oxygen occurs later, evidenced by the later increase of the O–H BCP electron density. In other words, NH_3_ results in an *asynchronous* transition state, whereas H_2_O results in a more synchronous process (see Section 6 of Supporting Information for more details).

**Figure 3:**
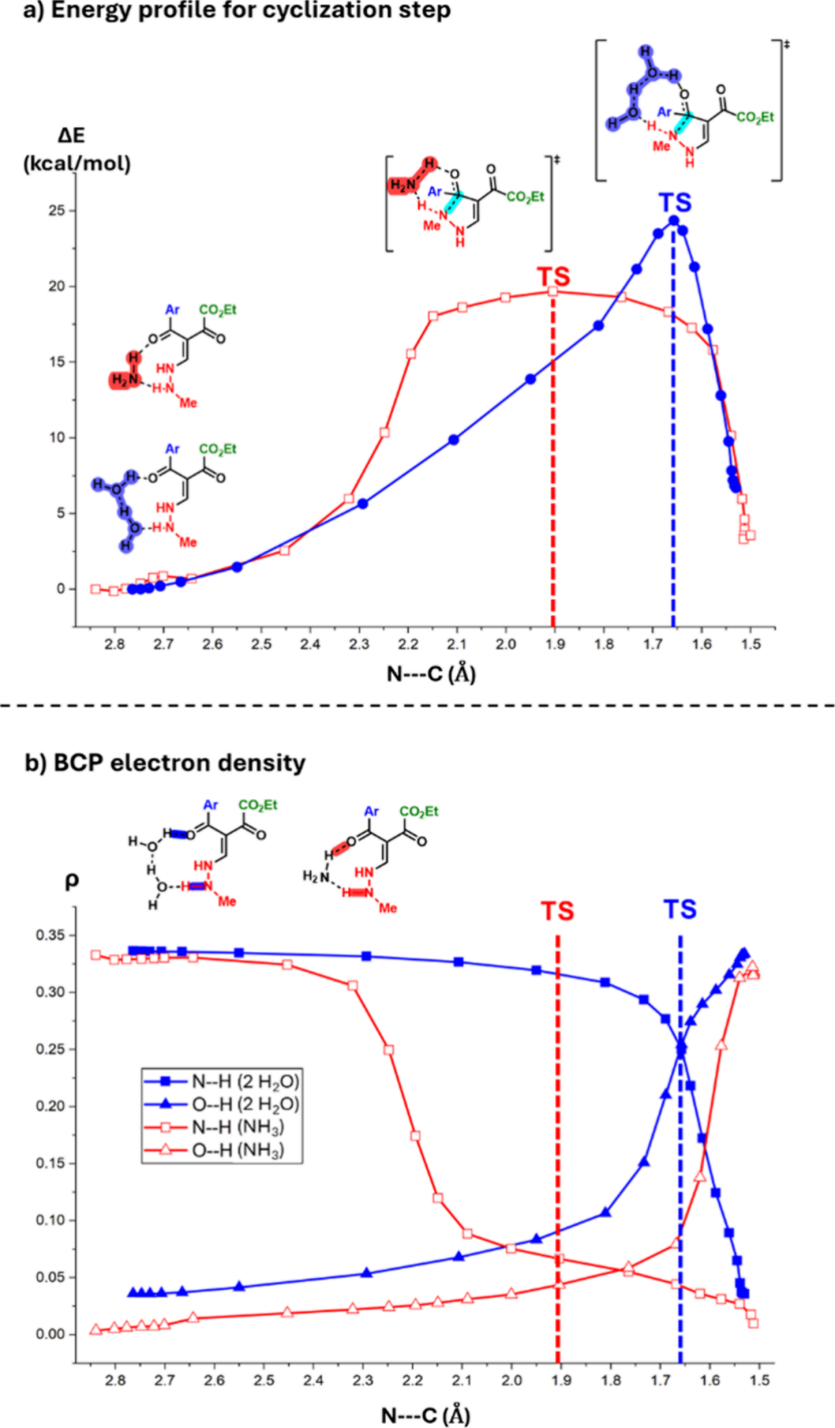
(a) Energy profile for the cyclization step considering the PTC as two H_2_O molecules (blue line) or one NH_3_ molecule (red line). The broader energy profile of the red line indicates the asynchronicity of the process, whereas the sharper energy profile of the blue line indicates a synchronous process. (b) Electron density of the N–H and O–H bond critical points.

To probe the origin of this difference in synchronicity, we employed localized orbital (IBO)^23^ analysis of the transition states. The IBO analysis (Figure 4) shows that the ∼90° angle between the H_2_O oxygen lone pair (lp) and the H_2_O O–H σ-bond allows for a more compact and efficient orbital overlap between the water molecules with the nucleophilic NH (NHMe or NH_2_) and the carbonylic oxygen. We also note that the large excess of H_2_O molecules available allows for the usage of as many molecules as necessary in order to promote the most efficient proton transfer possible. In contrast, the NH containing PTCs are available in a much smaller concentration since they are present only in stoichiometric concentrations, and they have a larger angle between the N lp and the N–H σ-bond. Consequently, there is a small probability of multiple molecules being available in order to promote an efficient proton transfer step, and the orbital interaction between the NH containing PTC with the nucleophilic NH and the carbonylic oxygen is less effective. This difference in orbital overlap is particularly evident when we compare the cyclization transition state employing one NH_3_ with that employing one H_2_O as PTC (Figure S31).Therefore, after the NH_3_ deprotonates the nucleophilic NH, it must reorganize itself in space in order to transfer a proton to the carbonylic oxygen, resulting in an asynchronous process.

**Figure 4:**
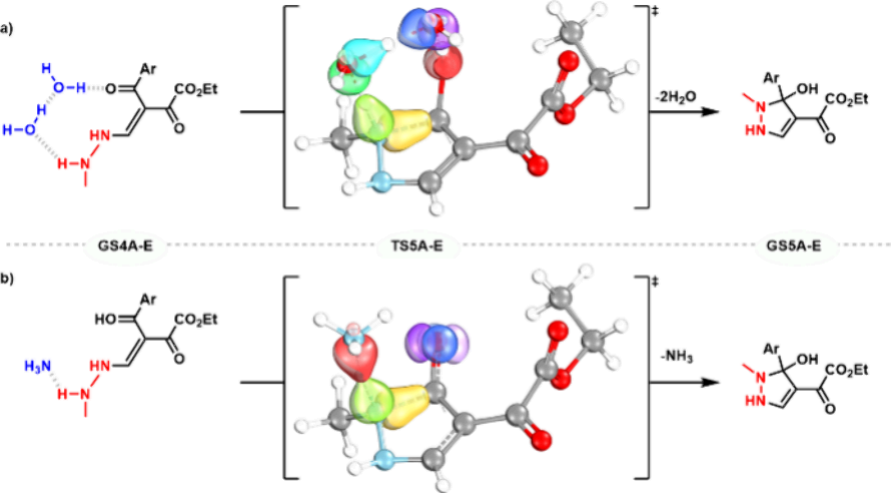
Comparison of intrinsic bond orbital (IBO) involvement in the cyclization transition state with (a) 2× H_2_O (synchronous, 7 IBOs) and (b) NH_3_ (asynchronous, 5 IBOs) as PTC.

The differing basicities of the PTCs is reflected in unique profiles of electronic density variation along this reaction path (Figures S35 and S36). As cyclization proceeds, charge is transferred from the nucleophilic nitrogen to the carbonyl carbon, with a portion accumulating on the carbonyl oxygen. In the presence of H_2_O, synchronous proton transfer rapidly stabilizes this charge buildup. Conversely, the higher basicity of NH_3_ promotes early deprotonation of the nucleophilic NH moiety, resulting in charge accumulation on the nitrogen and enhancing its nucleophilicity. This enhanced nucleophilic character correlates with the lower cyclization barriers observed with NH_3_—with the exception of TS5A-E, where both PTCs yield nearly identical barriers. Notably, **GS4A-Z** benefits most from this effect, exhibiting a cyclization barrier 3.5 kcal/mol lower than that in the presence of H_2_O (Compare Figures S8 and S4).

As cyclization is rate-limiting when the reaction is catalyzed by H_2_O, changing the nature of this step (by changing the PTC to NH_3_) affects the overall energy barriers. For the mechanisms employing NH_3_ as PTC, the computed barriers are 16.4, 19.9, 17.4, and 20.6 kcal/mol leading to products **P-AZ**, **P-AE**, **P-BZ**, and **P**-**BE**, respectively (see Figures S8 and S9). These results align with the trends in the experimental yields (53, 4, 43, and 0%, Scheme 2). As in the H_2_O catalyzed systems, once again the thermodynamics poorly correlate with yields.

Finally, we also investigated Paths A and B employing HNMe_2_ as PTC, which is a molecule eliminated in the previous step **TS4A/B** (Figures S14 and S15). The key conclusions remain the same, cyclization of intermediates **GS4A-Z** and **GS4B-Z** are favored compared to their counterparts **GS4A-E** and **GS4B-E**. These results corroborate our model using NH_3_ as the PTC.

Encouraged by these results, we hypothesized that if we could induce a synchronous proton transfer during cyclization, we would obtain product distribution similar to those obtained using H_2_O/MeOH as solvent, even when employing a polar aprotic solvent such as MeCN. The literature reports acetic acid as an efficient proton transfer catalyst.^24^ Therefore, we performed the reaction in MeCN as described previously,^19^ adding acetic acid to the reaction mixture in catalytic concentration (10 mol %) aiming to catalyze the proton transfer. To our delight, the product ratios obtained with this reaction protocol are indeed similar to the reaction performed with H_2_O/MeOH as solvent (Table 1).

**Table 1:**
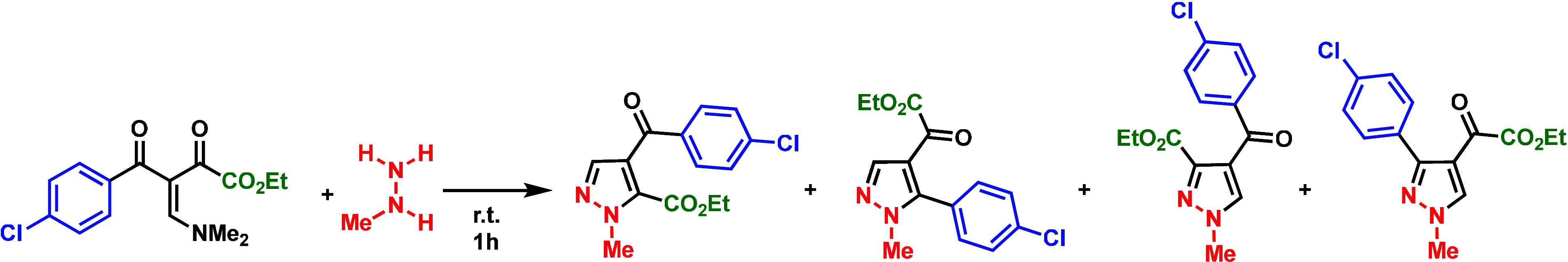
Impact of Solvent and PTC Additive on the Product Ratios

**entry**	**solvent**	**additive**	**yield (%) (P-AZ:P-AE:P-BZ:P-BE)**
1	H_2_O/MeOH (1:1)		30:30:40:0 ^19^
2	MeCN		53:04:43:0 ^19^
3	MeCN	AcOH (10 mol %)	20:28:52:0^a^

a Determined by ^1^H NMR of crude products.

The energy profiles for the reaction in MeCN catalyzed with AcOH (Figures S10 and S11) show that the cyclization step leading to products **P-AE** and **P-AZ** in Path A have comparable relative energies (9.4 and 9.3 kcal/mol respectively). In Path B, the cyclization step leading to product **P-BZ** has a barrier 1.6 kcal/mol lower than the competing one leading to product **P-BE** which is consistent with the majority formation of **P-BZ**. This energy profile is similar to the one reported for the reactions using H_2_O as PTC, which correlates with the similarity in product ratios.

## Conclusions

This work not only resolves the long-standing regioselectivity puzzle for BEDs but also establishes a new paradigm for understanding and controlling proton-transfer-mediated reactions in complex synthetic systems. We have identified that the cyclization activation barriers, which dictate the product distribution, are highly sensitive to the Proton Transfer Catalyst (PTC). Further, the PTC modulates the synchronicity of the transition states, with water as PTC resulting in a highly synchronized process. This synchronicity seems to be a consequence of (i) the orbital configuration of water, and (ii) concentration effects. These results provide a clear mechanistic framework for controlling reaction outcomes with BEDs. We hypothesize that the product ratio can also be tuned by manipulating the electronic landscape through methods such as Lewis acid coordination and precise pH control, establishing a foundation for designing optimized synthetic protocols for reactions where a concerted proton transfer step is rate-determining, paving the way for more rational catalyst design.

### Computational Methods

The acquisition of accurate molecular geometries is a prerequisite for constructing reliable energy diagrams. Owing to the conformational flexibility inherent in these systems, extensive conformational searches were performed (for all relevant species, reactants, intermediates, transition states, and products) using CREST,^25^ with GFN2-xTB.^26^ A selection of conformers were optimized and ranked by the composite PBEh-3c^27^ method in the ORCA 6 package.^28,29^ The lowest energy conformer was subsequently optimized at the ωB97X-D3­(BJ)/def2-TZVP level.^30–32^ Electronic energies for mechanistically critical steps were also computed using DLPNO–CCSD­(T)/cc-pVTZ,^33,34^ and the corresponding auxiliary basis set cc-pVTZ/C. Solvent effects were modeled implicitly using CPCM solvation model.^35^ We also computed the energy profiles for the reaction employing H_2_O as PTC with B3LYP-D3­(BJ)^36^ and M06–2X^37^ functionals, both employing def2-TZVP basis set and CPCM­(water) (Figures S16–S19). The key conclusions of this work were found to be largely independent of the nature of the method used.

For computing the transition states employing the PTCs, we used the Nudged Elastic Band (NEB)^38^ method at B3LYP-D3­(BJ)/def2-TZVP theory level. The converged Climbing Image was submitted to a transition state optimization at ωB97X-D3­(BJ)/def2-TZVP level of theory. Intrinsic Reaction Coordinate (IRC) calculations were performed on the cyclization transition states (Figures S33 and S34) to ensure they connect the expected reactant and products.

To further investigate the properties of the cyclization transition states, we used the reaction path generated by the NEB calculation in order to perform topology, IBO change and charge variation analysis. These analyses were performed with B3LYP-D3BJ/def2-TZVP level of theory due to prohibitive computational cost of NEB at ωB97X-D3­(BJ)/def2-TZVP level of theory.

## Supplementary Material



## Data Availability

The data underlying this study are available in the published article and its Supporting Information.

## Notes

General remarks, experimental and computational details, spectra, and XYZ coordinates (PDF)
