# Supplementary material for: Regioselectivity of the Reaction between β‑Enamino Diketones and Methylhydrazine Explained
Source: J Org Chem. 2026 Mar 14;91(12):4331–8. doi: 10.1021/acs.joc.5c03194 (PMC13036766; doi:10.1021/acs.joc.5c03194)
Supplement: Supplementary file 1 [file jo5c03194_si_001.pdf]

Supporting Information

for

Regioselectivity of the Reaction between β-Enamino Diketones and Methylhydrazine Explained

Vinicius Martinelli,[a]* Isaac F. Leach,[b] Julia Poletto,[a] Wagner E. Richter,[a] Fernanda A. Rosa,[a] Rodrigo M. Pontes[a]

[a] Departamento de Química, Universidade Estadual de Maringá, 87020-900, Maringá, Brasil.

[b] Dipartimento di Chimica, Biologia e Biotecnologie, Università degli Studi di Perugia, 06123 Perugia, Italy.

Table of Content

S1.Experimental general information…………………………………………S3

S2.Computational protocol……………………………………………….....S4

S3.Complete mechanism computed at ωB97X-D3(BJ)/def2-TZVP level of theory…………………………………………………………………………….....S5

S4.Simplified energy diagrams computed at different theory levels…...........S12

S5.Isomerization of GS4 intermediate……………………………………..S15

S6.Electronic properties for the cyclization transition states considering different PTCs………………………………………………………………………....S15

S7.Interaction Region Indicator (IRI) for the cyclization transition states…………………………………………………………………………S24

S8.Intrinsic Reaction Coordinate (IRC) for the cyclization transition states…………………………………………………………………………S25

S9.Charge Variation Analysis…………………………………………………S26

S10.Kinetic investigation of different BEDs…………………………………S28

S11.References………………………………………………………......S31

S12.^{1}H and ^{13}C{^{1}H} NMR Spectra for *N*-methylpyrazoles………………....S33

S13.XYZ Structures……………………………………………………...S40

S1. Experimental general information

The reactions performed with the solvents H_2O/MeOH (1:1) and MeCN, in the absence of AcOH as PTC, resulting in the products **P-AE**, **P-AZ** and **P-BZ**, were originally reported by Rosa and colleagues in ref. 1.

For the reaction employing AcOH as PTC, performed in this work, all reagents were used as obtained from commercial suppliers without further purification. Solvents were dried and purified according to recommended procedures.[2] The reactions were monitored by thin-layer chromatography using Merck TLC silica gel plates and visualized with UV light (254 nm). The column chromatography used was silica gel 60, with 230-400 mesh (Merck). ^{1}H NMR and ^{13}C{^{1}H} NMR experiments were run on Bruker Avance III HD apparatus operating at ^{1}H 500.13 MHz and ^{13}C 125.77 MHz and all structural assignments were made with additional information from gHSQC and gHMBC experiments. Chemical shifts are reported in δ (parts per million), using $CDCl_3$ as solvent, and employing the following abbreviations: s (singlet), d (doublet), t (triplet) and q (quartet).

S1.1. Experimental Procedure and Characterization

S1.1.1 General Procedure for the Synthesis of *N*-methylpyrazole Regioisomers employing AcOH as PTC

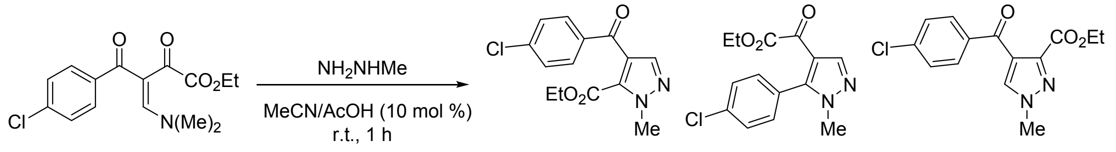

1 **P-AZ** **P-AE** **P-BZ**

To a solution of tertiary β-enamino diketone **1**[3] (0.309 g, 1.0 mmol, 1.0 equiv), in MeCN and 10 mol % of acetic acid (0.006 g, 0.1 mmol), methylhydrazine was added (1.2 mmol, 1.2 equiv, 0.055 g). Then, the mixture was stirred at room temperature for 1 hour (monitored by TLC). After this time, the reaction mixture was diluted with saturated NaCl (aq.) (25 mL) and extracted with dichloromethane (3 × 20 mL). The organic layer was dried over anhydrous sodium sulfate, concentrated under reduced pressure and the product was isolated on a silica gel chromatography column using a 70:30 mixture of hexane: ethyl acetate as the eluent.

Ethyl 4-(4-chlorobenzoyl)-1-methyl-1*H*-pyrazole-5-carboxylate (P-AZ): white solid (MeCN/AcOH (10 mol %): 0.054 g, 18% yield).**^{1}H NMR (500.13 MHz, $CDCl_3$):** δ 7.75 (d, 2H, *J* = 8.4 Hz, 4-ClC_6H_4), 7.67 (s, 1H, H3), 7.42 (d, 2H, *J* = 8.4 Hz, 4-ClC_6H_4), 4.16 (s, 3H, CH_3), 4.06 (q, 2H, *J* = 7.2 Hz, $CO_2\underline{CH_2}CH_3$), 0.96 (t, 3H, *J* = 7.2 Hz, $CO_2CH_2\underline{CH_3}$). **^{13}C{^{1}H} NMR (125.77 MHz, $CDCl_3$):** δ 188.7 (C4'), 159.6 (C5'), 139.6 (C3), 139.1 (4-ClC_6H_4), 136.9 (4-ClC_6H_4), 132.9 (C5),

130.8 (4-ClC_6H_4), 128.9 (4-ClC_6H_4), 124.1 (C4), 61.9 ($CO_2\underline{CH_2}CH_3$), 39.7 (CH_3), 13.5 ($CO_2CH_2\underline{CH_3}$).

Ethyl 2-(5-(4-chlorophenyl)-1-methyl-1*H*-pyrazol-4-yl)-2-oxoacetate (P-AE): white solid (MeCN/AcOH (10 mol %): 0.075 g, 26% yield). **^{1}H NMR (500.13 MHz, $CDCl_3$):** δ 8.27 (s, 1H, H3), 7.47 (d, 2H, *J* = 8.4 Hz, 4-ClC_6H_4), 7.31 (d, 2H, *J* = 8.4 Hz, 4-ClC_6H_4), 4.21 (q, 2H, *J* = 7.1 Hz, $CO_2\underline{CH_2}CH_3$), 3.75 (s, 3H, CH_3), 1.31 (t, 3H, *J* = 7.1 Hz, $CO_2CH_2\underline{CH_3}$). **^{13}C{^{1}H} NMR (125.77 MHz, $CDCl_3$):** δ 178.6 (C4'), 162.8 ($\underline{C}O_2CH_2CH_3$), 146.4 (C5), 142.8 (C3), 136.4 (4-ClC_6H_4), 131.3 (4-ClC_6H_4), 129.1 (4-ClC_6H_4), 126.6 (4-ClC_6H_4), 117.4 (C4), 62.3 ($CO_2\underline{CH_2}CH_3$), 37.4 (CH_3), 14.1 ($CO_2CH_2\underline{CH_3}$).

Ethyl 4-(4-chlorobenzoyl)-1-methyl-1*H*-pyrazole-3-carboxylate (P-BZ): white solid (MeCN/AcOH (10 mol %): 0.140 g, 48% yield). **^{1}H NMR (500.13 MHz, $CDCl_3$):** δ 7.73 (d, 2H, *J* = 8.5 Hz, 4-ClC_6H_4), 7.72 (s, 1H, H5), 7.39 (d, 2H, *J* = 8.5 Hz, 4-ClC_6H_4), 4.13 (q, 2H, *J* = 7.1 Hz, $CO_2\underline{CH_2}CH_3$), 4.01 (s, 3H, CH_3), 1.06 (t, 3H, *J* = 7.1 Hz, $CO_2CH_2\underline{CH_3}$). **^{13}C{^{1}H} NMR (125.77 MHz, $CDCl_3$):** δ 188.5 (C4'), 161.3 ($\underline{C}O_2CH_2CH_3$), 142.6 (C3), 139.5 (4-ClC_6H_4), 136.9 (4-ClC_6H_4), 133.7 (C5), 130.7 (4-ClC_6H_4), 128.8 (4-ClC_6H_4), 123.3 (C4), 61.5 ($CO_2\underline{CH_2}CH_3$), 40.0 (CH_3), 13.9 ($CO_2CH_2\underline{CH_3}$).

S2. Computational protocol

Due to the high flexibility of the molecules participating in this work, conformational search for every molecule was performed using CREST module[4] of XTB package, employing the GFB-xTB tight theory level. [5] Solvation effects for water were initially accounted for using Analytical Linearized Poisson-Boltzmann (ALPB) model.

The initial batch of conformers resulted from CREST search were clustered by geometrical similarity up to a new batch of 50 conformers. This new batch was reoptimized using the low-cost method PBEh-3c,[6] with solvation effects for water accounted for using the Conductor-like Polarizable Continuum (C-PCM) formalism,[6] this calculations were performed in ORCA 6.[8,9] The new lowest energy conformer was finally reoptimized employing the DFT functional ωB97X-D3(BJ)[10,11] with the basis set def2-TZVP,[12] and solvation effects accounted for with the C-PCM formalism. This DFT functional has been benchmarked on wide variety of systems, and is known to perform particularly well for organic molecules.[13-16] Computations considering the solvent acetonitrile were performed using the best geometries obtained from the previous conformational search. The free energy of key mechanistic steps had the electronic contribution corrected with single point with DLPNO-CCSD(T) theory, using the basis set cc-pVTZ and cc-pVTZ/C as an auxiliary basis set, with the CPCM theory for solvation effects. To further verify the consistency of our results, we also computed the most important steps for the reaction employing H_2O as PTC using the well-known functionals B3LYP and M06-2X.

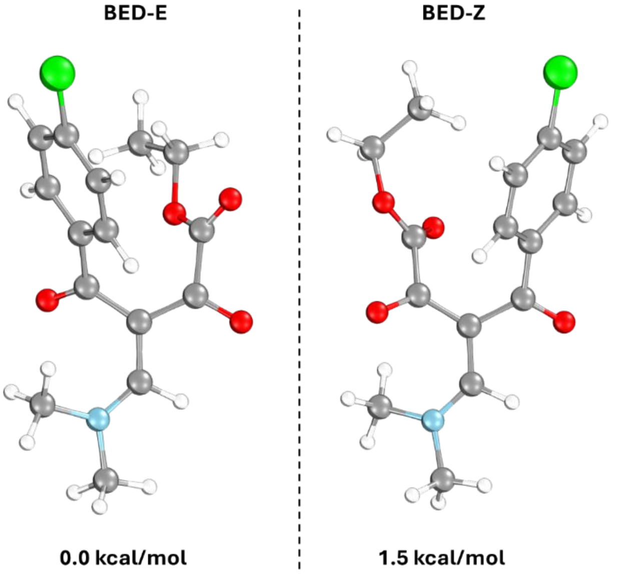

Figure S1. Minimum energy configurations (*E* left and *Z* right) for the BED. Computed at DLPNO-CCSD(T)/cc-PVTZ//ωB97X-D3(BJ)/def2-TZVP, CPCM(water) level of theory.

S3. Complete mechanism computed at ωB97X-D3(BJ)/def2-TZVP level of theory

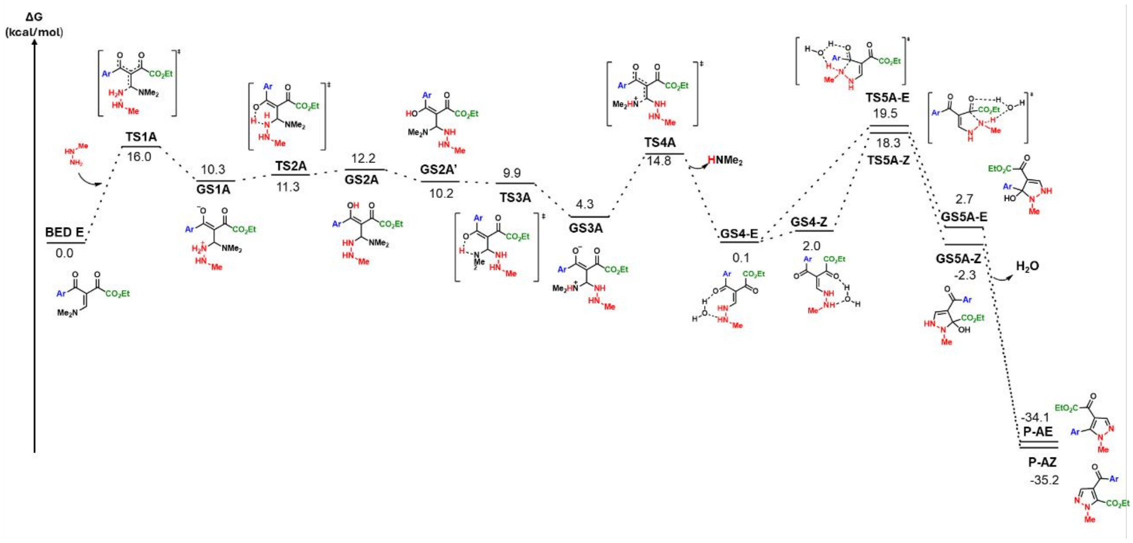

Figure S2. Complete energy profile for the catalyzed Path A with one H_2O as proton transfer catalyst. Computed at ωB97X-D3(BJ)/def2-TZVP, CPCM(water). Values between parenthesis are relative energies corrected with DLPNO-CCSD(T)/cc-pVTZ, CPCM(water).

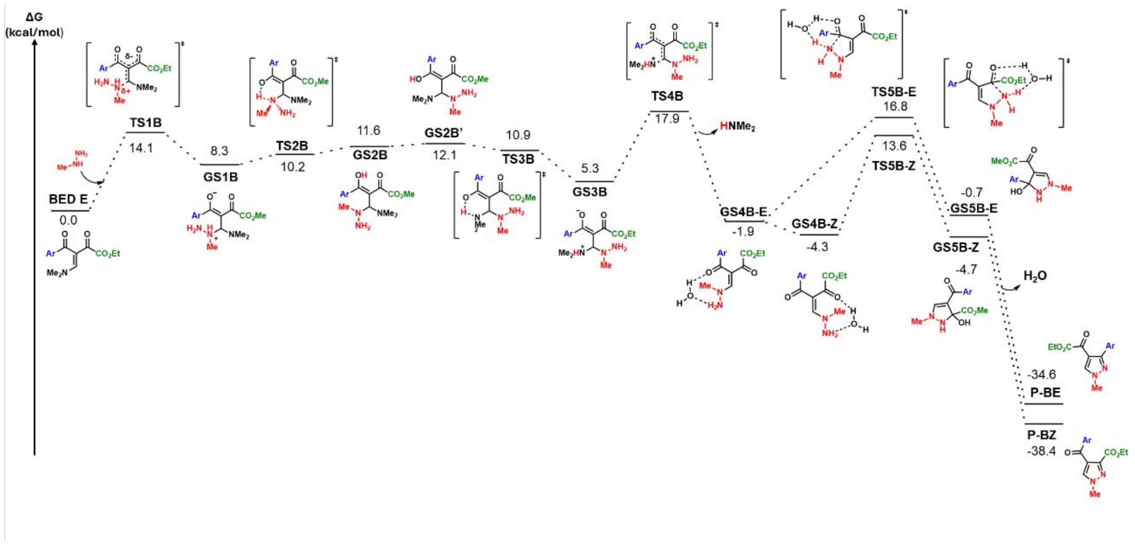

Figure S3. Complete energy profile for the catalyzed Path B with one H_2O as proton transfer catalyst. Computed at ωB97X-D3(BJ)/def2-TZVP, CPCM(water). Values between parenthesis are relative energies corrected with DLPNO-CCSD(T)/cc-pVTZ, CPCM(water).

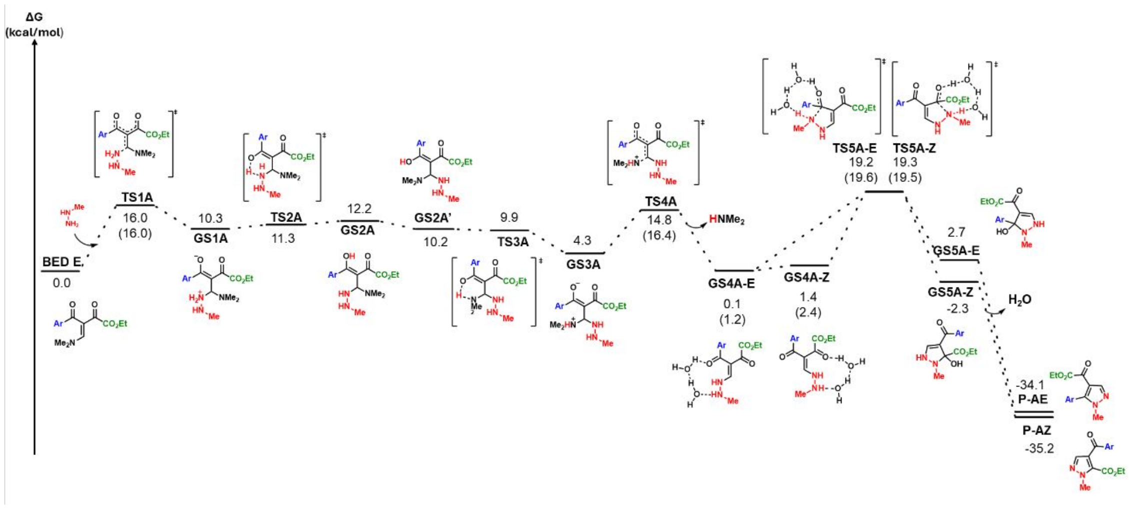

Figure S4. Complete energy profile for the catalyzed Path A with 2xH_2O as proton transfer catalyst. Computed at ωB97X-D3(BJ)/def2-TZVP, CPCM(water). Values between parenthesis are relative energies corrected with DLPNO-CCSD(T)/cc-pVTZ, CPCM(water).

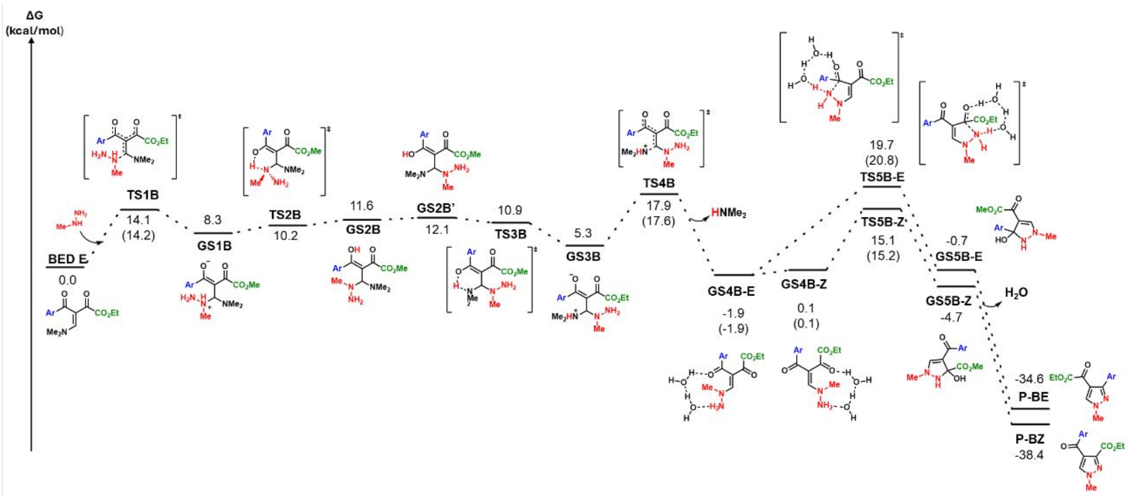

Figure S5. Complete energy profile for the catalyzed Path B with 2xH_2O as proton transfer catalyst. Computed at ωB97X-D3(BJ)/def2-TZVP, CPCM(water). Values between parenthesis are relative energies corrected with DLPNO-CCSD(T)/cc-pVTZ, CPCM(water).

+

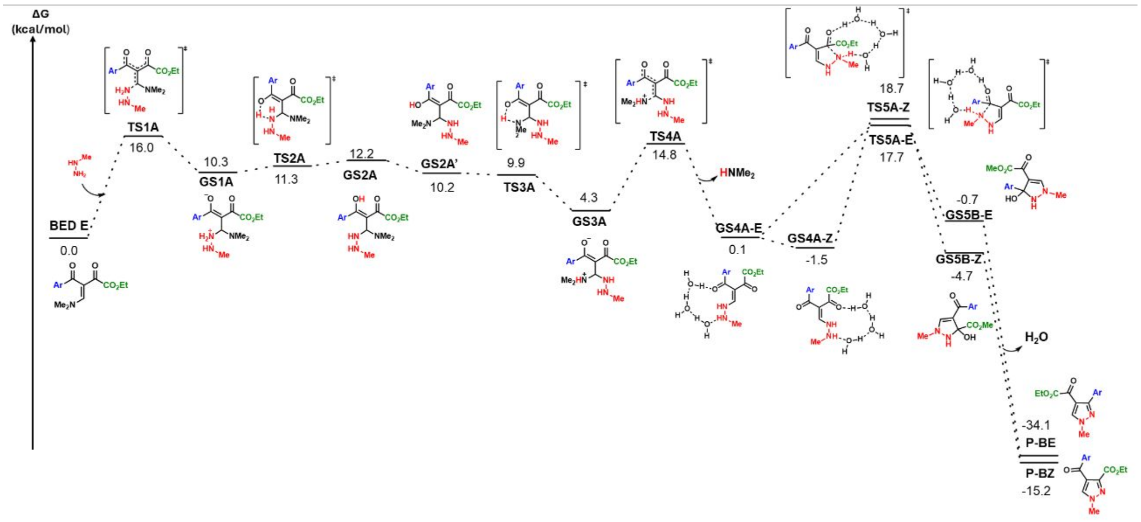

Figure S6. Complete energy profile for the catalyzed Path A with 3xH_2O as proton transfer catalyst. Computed at ωB97X-D3(BJ)/def2-TZVP, CPCM(water). Values between parenthesis are relative energies corrected with DLPNO-CCSD(T)/cc-pVTZ, CPCM(water).

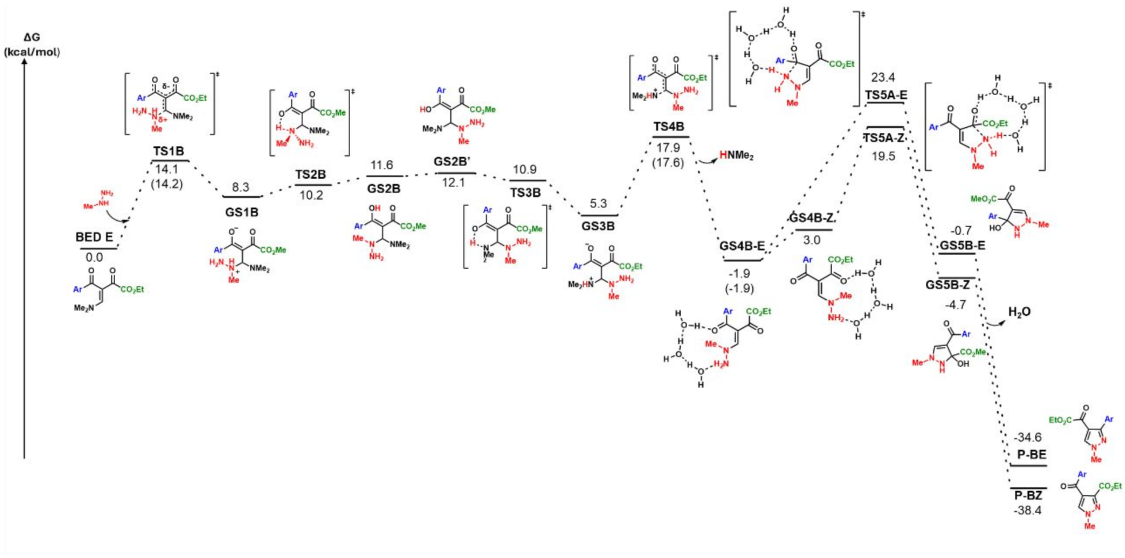

Figure S7. Complete energy profile for the catalyzed Path B with 3xH_2O as proton transfer catalyst. Computed at ωB97X-D3(BJ)/def2-TZVP, CPCM(water). Values between parenthesis are relative energies corrected with DLPNO-CCSD(T)/cc-pVTZ, CPCM(water).

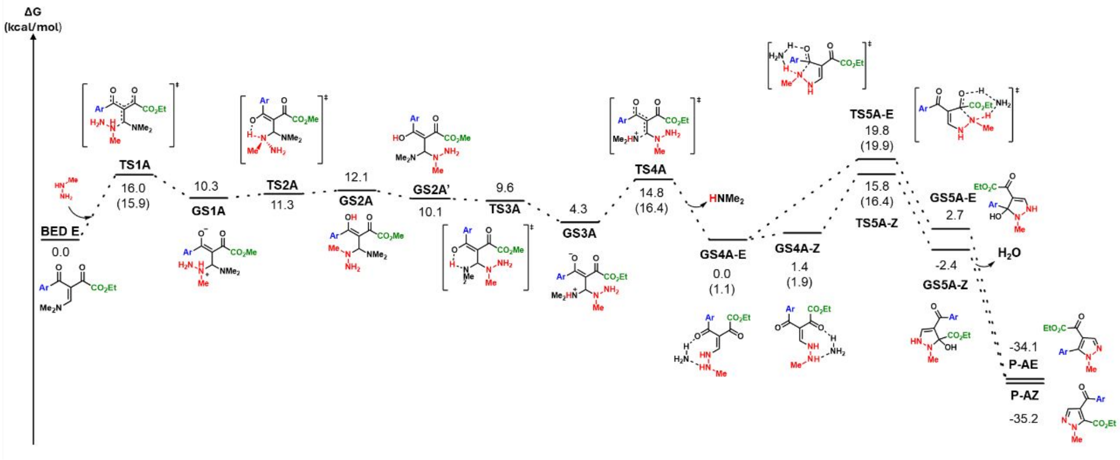

Figure S8. Complete energy profile for the NH_3 proton transfer catalyzed Path A. Computed at ωB97X-D3(BJ)/def2-TZVP, CPCM(Acetonitrile). Values between parenthesis are relative energies corrected with DLPNO-CCSD(T)/cc-pVTZ, CPCM(Acetonitrile).

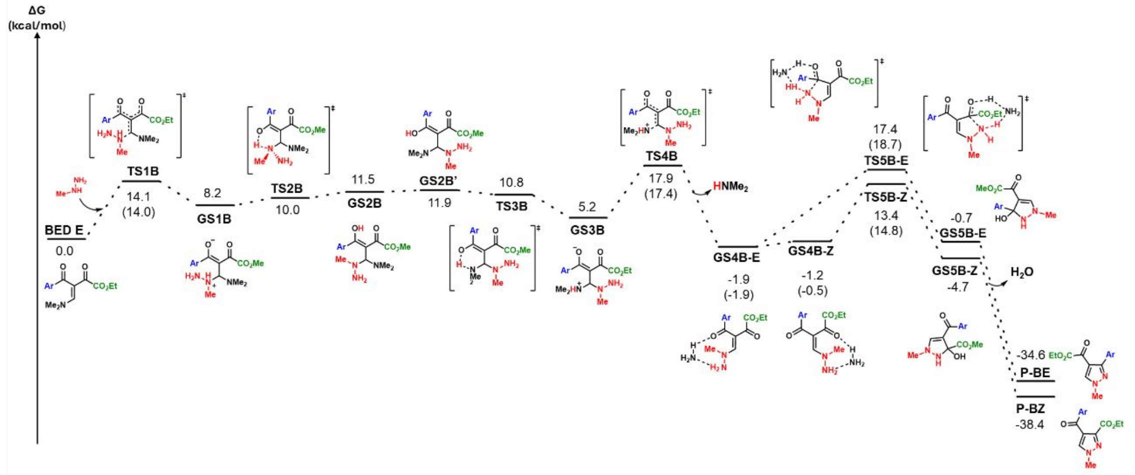

Figure S9. Complete energy profile for the NH_3 proton transfer catalyzed Path B. Computed at ωB97X-D3(BJ)/def2-TZVP, CPCM(Acetonitrile). Values between parenthesis are relative energies corrected with DLPNO-CCSD(T)/cc-pVTZ, CPCM(Acetonitrile).

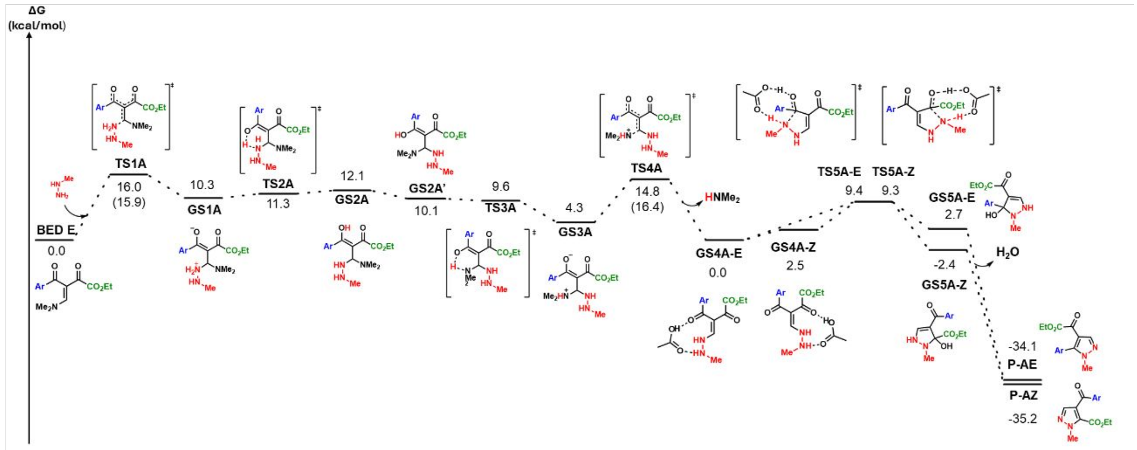

Figure S10. Complete energy profile for the AcOH proton transfer catalyzed Path A. Computed at ωB97X-D3(BJ)/def2-TZVP, CPCM(Acetonitrile). Values between parenthesis are relative energies corrected with DLPNO-CCSD(T)/cc-pVTZ, CPCM(Acetonitrile).

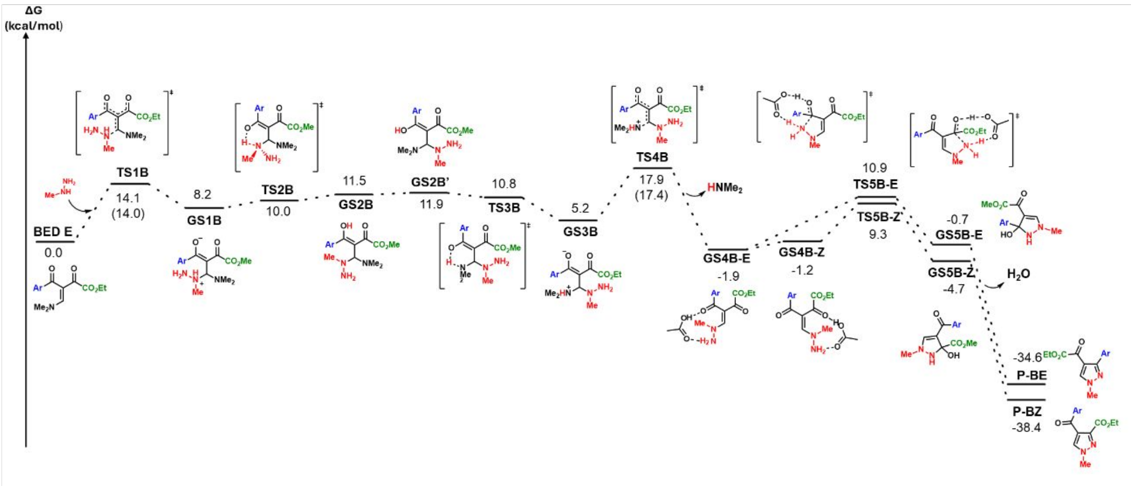

Figure S11. Complete energy profile for the AcOH proton transfer catalyzed Path B. Computed at ωB97X-D3(BJ)/def2-TZVP, CPCM(Acetonitrile). Values between parenthesis are relative energies corrected with DLPNO-CCSD(T)/cc-pVTZ, CPCM(Acetonitrile).

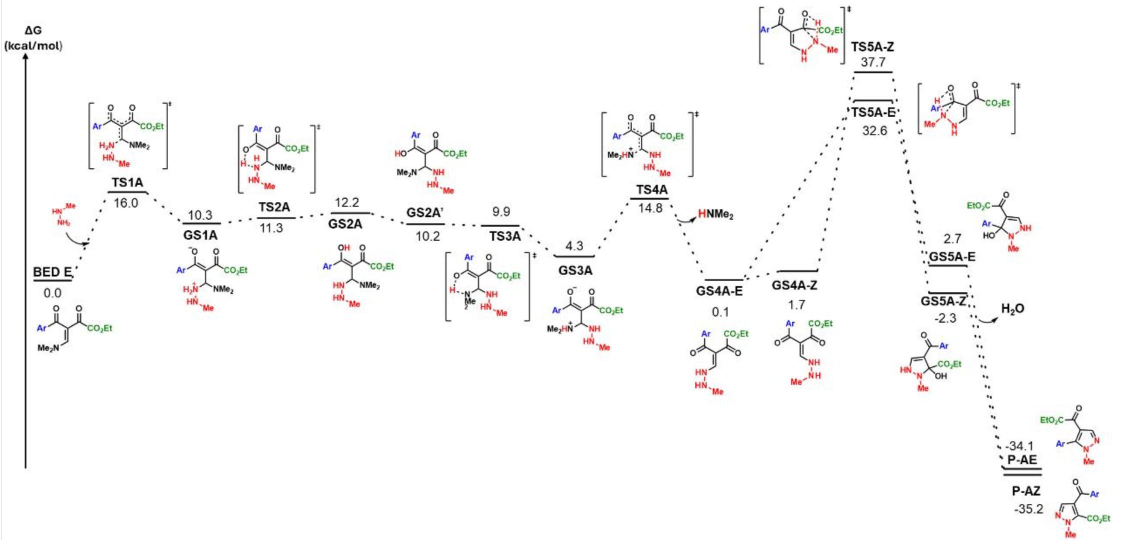

Figure S12. Complete energy profile for Path A in the absence of a PTC. Computed at ωB97X-D3(BJ)/def2-TZVP, CPCM(water).

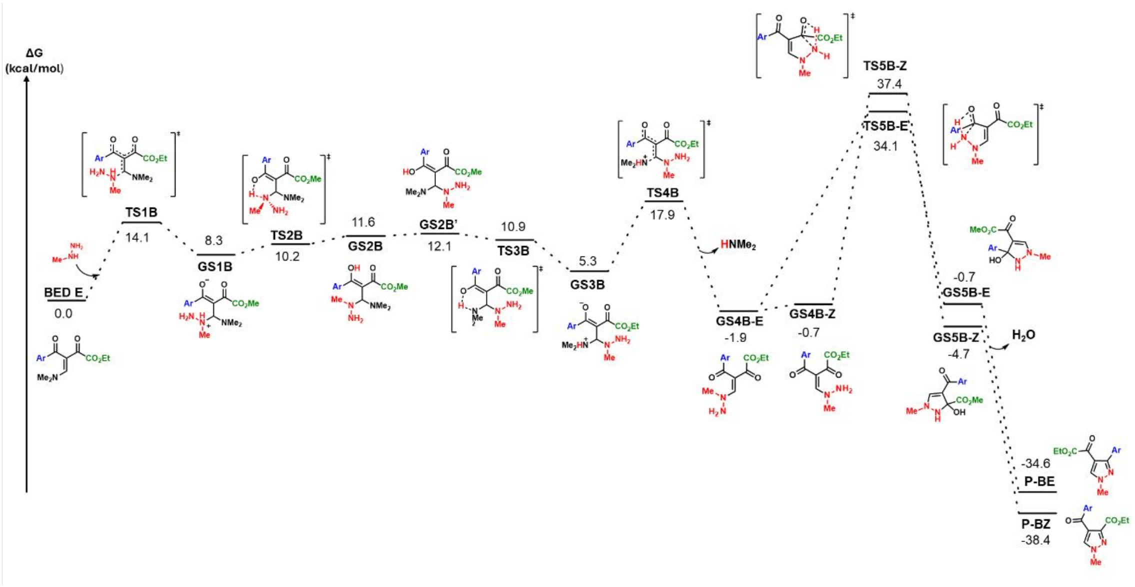

Figure S13. Complete energy profile for Path B in the absence of a PTC. Computed at ωB97X-D3(BJ)/def2-TZVP, CPCM(water).

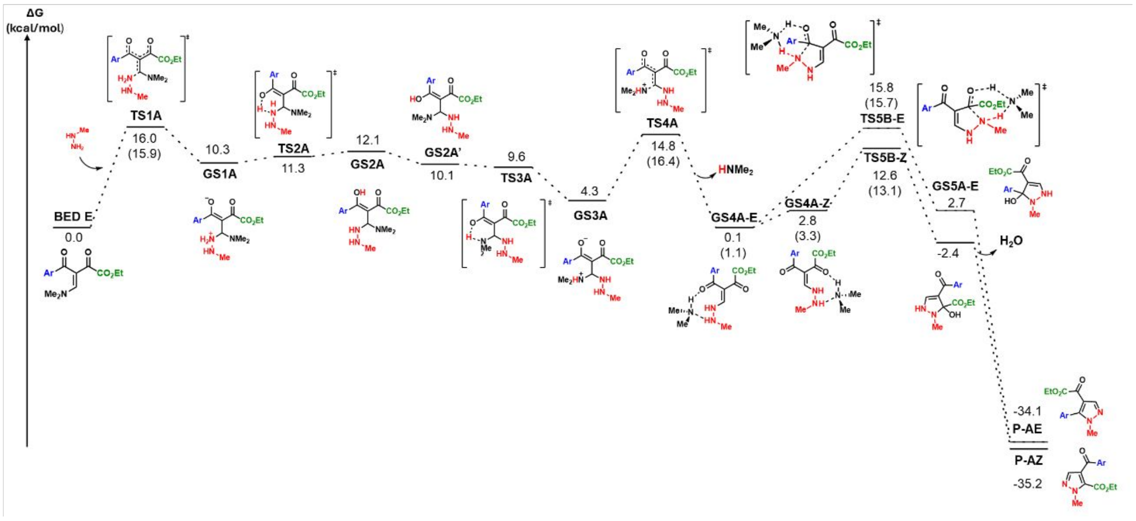

Figure S14. Complete energy profile for Path A with $HNMe_2$ as PTC. Computed at ωB97X-D3(BJ)/def2-TZVP, CPCM(water).

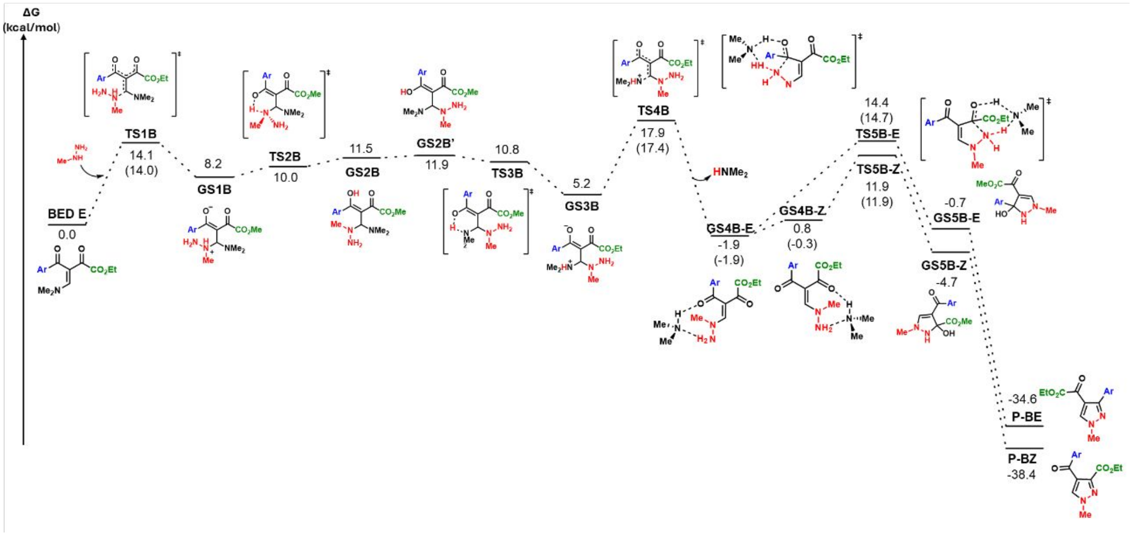

Figure S15. Complete energy profile for Path B with $HNMe_2$ as PTC. Computed at ωB97X-D3(BJ)/def2-TZVP, CPCM(water).

Figures S14 and S15 exhibit the energy profile for Paths A and B, respectively, employing $HNMe_2$ as PTC, which is a molecule eliminated in the previous step TS4A/B. Compared to NH_3, $HNMe_2$ provides lower cyclization barriers. However, the main conclusions remain the same, $HNMe_2$ still favors cyclization of **GS4A-Z** and **GS4B-Z** compared to their counterparts **GS4A-E** and **GS4B-E**. Because of the smaller cyclization barrier, the rate determining step changes to **TS4A** and **TS4B**. These results still agree with experimental product yield (**P-AZ:P-AE:P-BZ:P-BE**= 53:04:43:0). We note that the products originating from Path A are majority (**P-AZ** (53%) + **P-AE** (04%) = 57%) compared to Path B (**P-BZ** (43%) + **P-BE** (0%) = 43%). This is consistent with the smaller kinetic barrier of Path A (16.4 kcal/mol) compared to Path B (17.9 kcal/mol).

S4. Simplified energy diagrams computed at different theory levels.

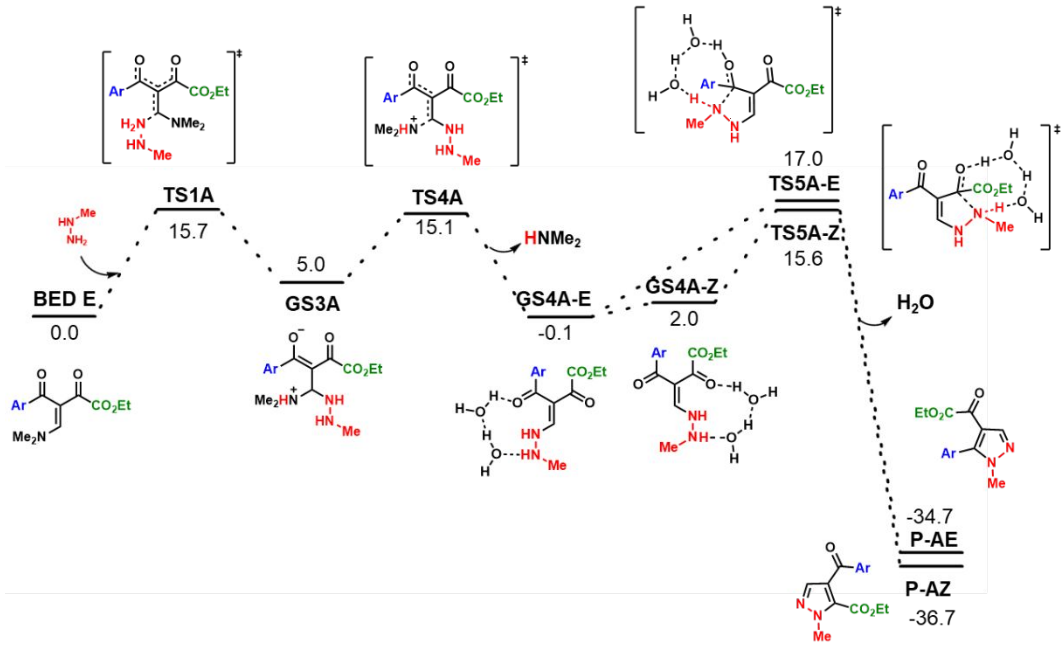

Figure S16. Energy diagram considering only the most important intermediates of Path A, computed at M06-2X/def2-TZVP, CPCM(water).

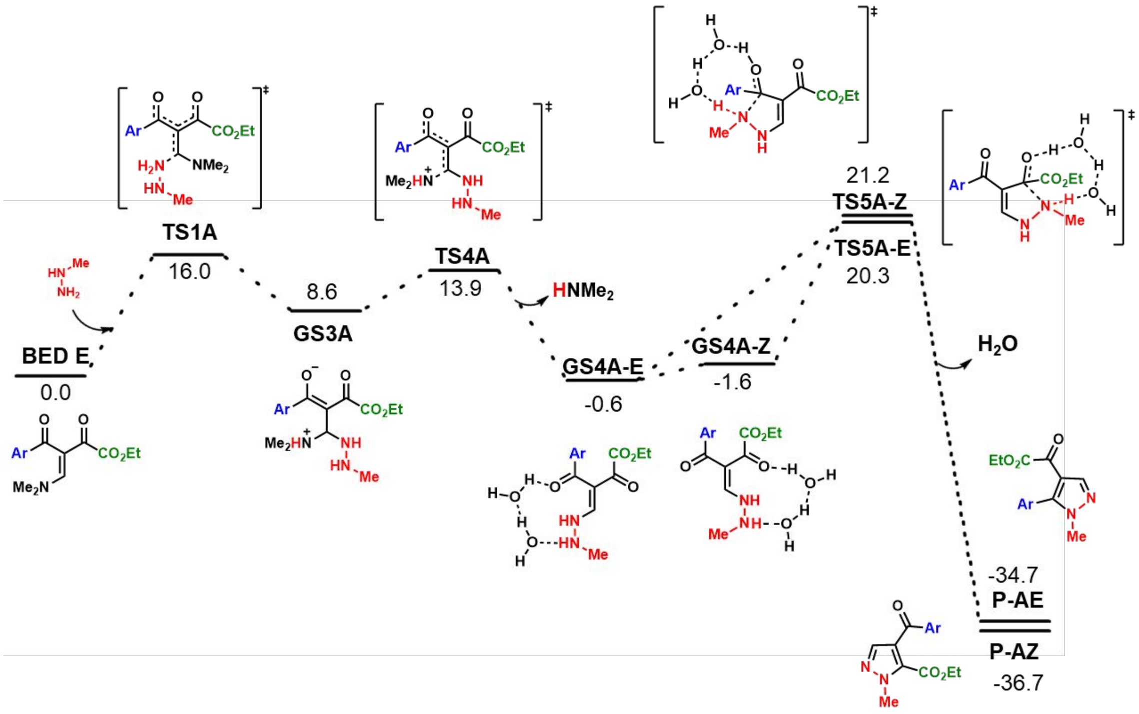

Figure S17. Energy diagram considering only the most important intermediates of Path A, computed at B3LYP-D3BJ/def2-TZVP, CPCM(water).

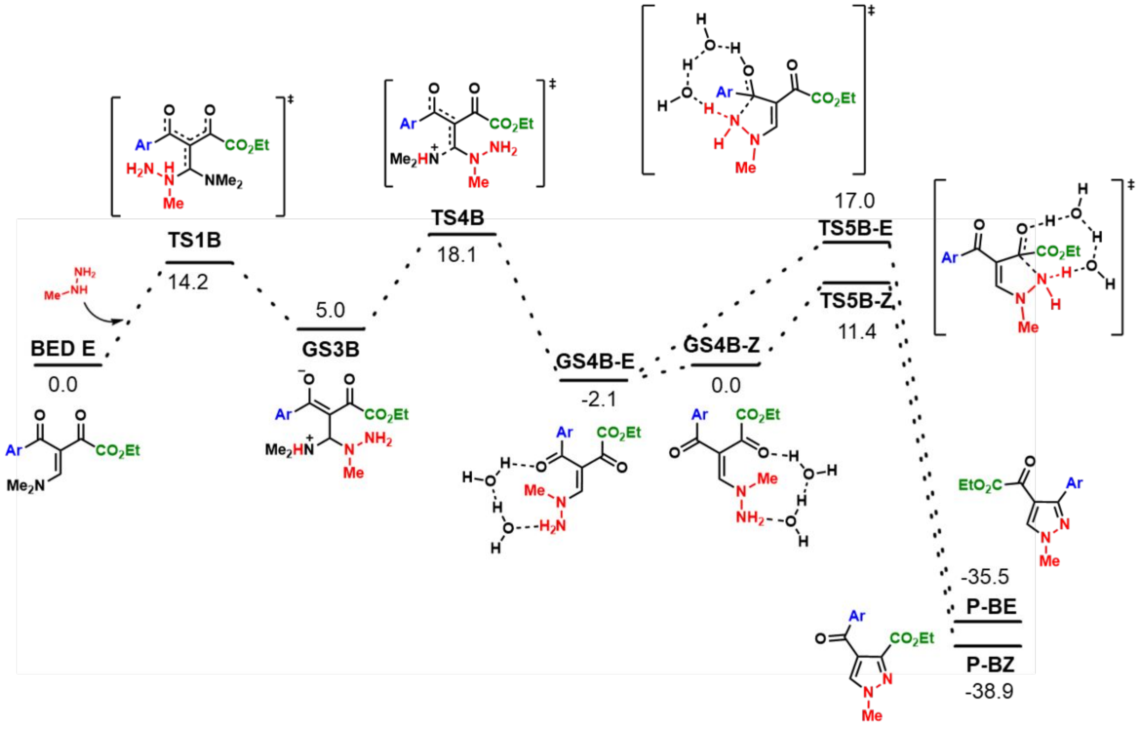

Figure S18. Energy diagram considering only the most important intermediates of Path B, computed at M06-2X/def2-TZVP, CPCM(water).

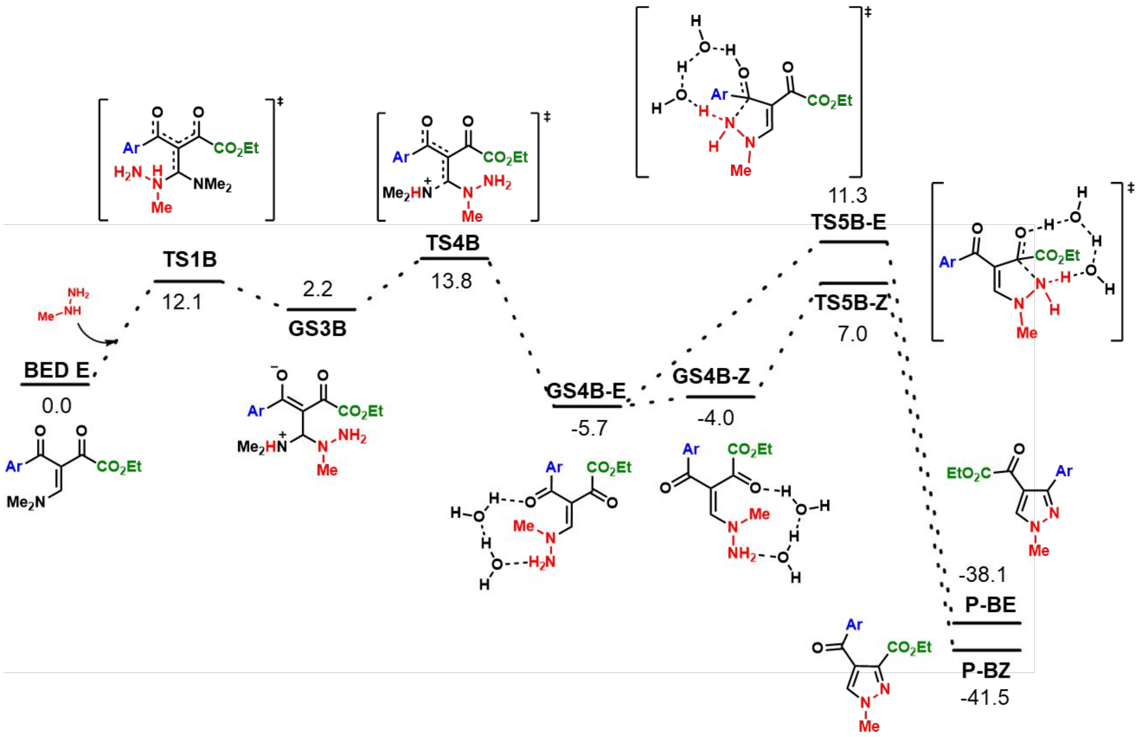

Figure S19. Energy diagram considering only the most important intermediates of Path B, computed at B3LYP-D3BJ/def2-TZVP, CPCM(water).

Upon analyzing Figures S11-S14, we note the different theory levels provide similar trends as ωB97X. The most important one is that intermediates **GS4A-Z** and **GS4B-Z** are higher in energy, but more reactive towards cyclization (lower cyclization energy barrier) compared to their *E* equivalents (**GS4A-E** and **GS4B-E**). For Path A, these properties of intermediates **GS4A-E** and **GS4A-Z** results in similar relative energies for the cyclization transition state. For Path B, the increased reactivity of **GS4B-Z** largely surpasses its decreased stability, resulting in a more favorable reaction path.

S5.Isomerization of GS4 intermediate.

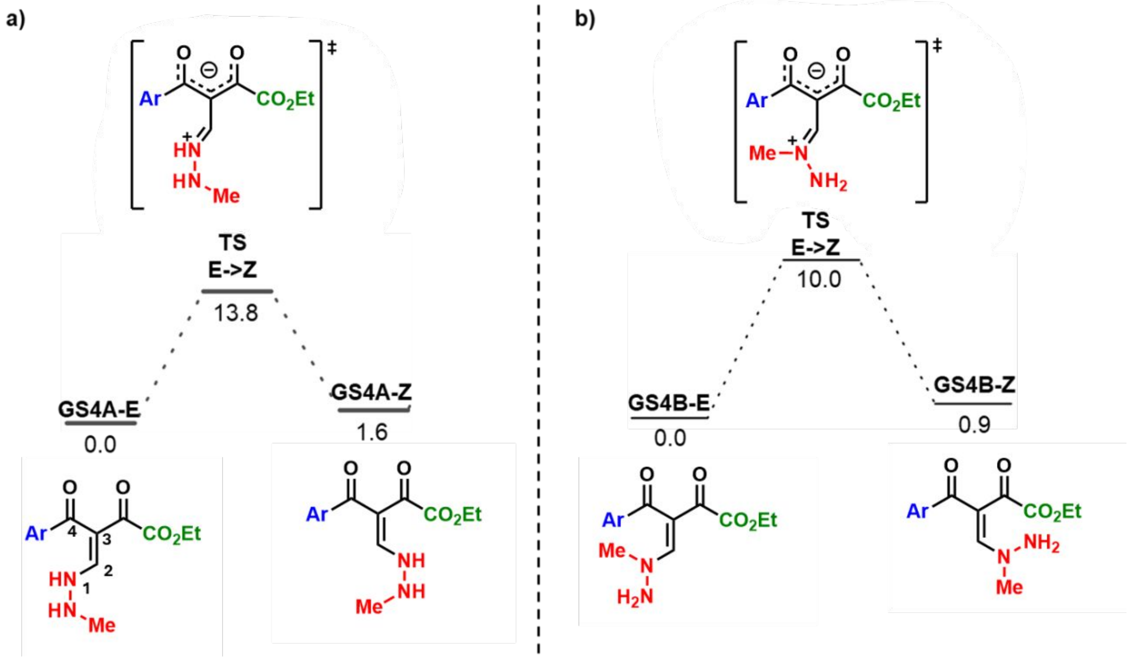

Figure S20. Isomerization mechanism for GS4A-E to GS4A-Z (**a**), and GS4B-E to GS4B-Z. Computed at ωB97X-D3BJ/def2-TZVP, CPCM(water).

The isomerization transition state was obtained from a 360º scan in 36 steps, of the dihedral angle between the planes composed by the atoms 1-2-3 and 2-3-4. The highest energy point was used for a transition state optimization computation. No PTC molecule was included.

Note that the isomerization barriers are lower than the cyclization barriers. Therefore, even if a lower energy isomerization mechanism exists, the conclusions should not be affected.

S6.Electronic properties for the cyclization transition states considering different PTCs.

In this section, we investigate the synchronicity of the cyclization transition state using two different analysis: electron density variation of the BCPs involved in the transition state, that is, the forming N-C and O-H; and breaking N-H bonds. The second analysis is the IBOs change along the reaction coordinate.

In the absence of a PTC, the N-H and O-H BCP's electron density starts to decrease and increase respectively, in a very synchronized fashion (Figure S21). This is consistent with the fact that the proton transfer occurs directly between these atoms (N and O). The N-C BCPs is formed and starts to increase its electron density only after the proton exchange. The IBOs change analysis shows as the nitrogen lone pair attacks the carbonyl, the C=O π evolves into a O lone pair which then overlaps with the N-H proton forming the new O-H σ

bond (Figure S22). The proton exchange step is very synchronized while the N-C bond formation occurs a little later, which is consistent with the BCP's electron density variation.

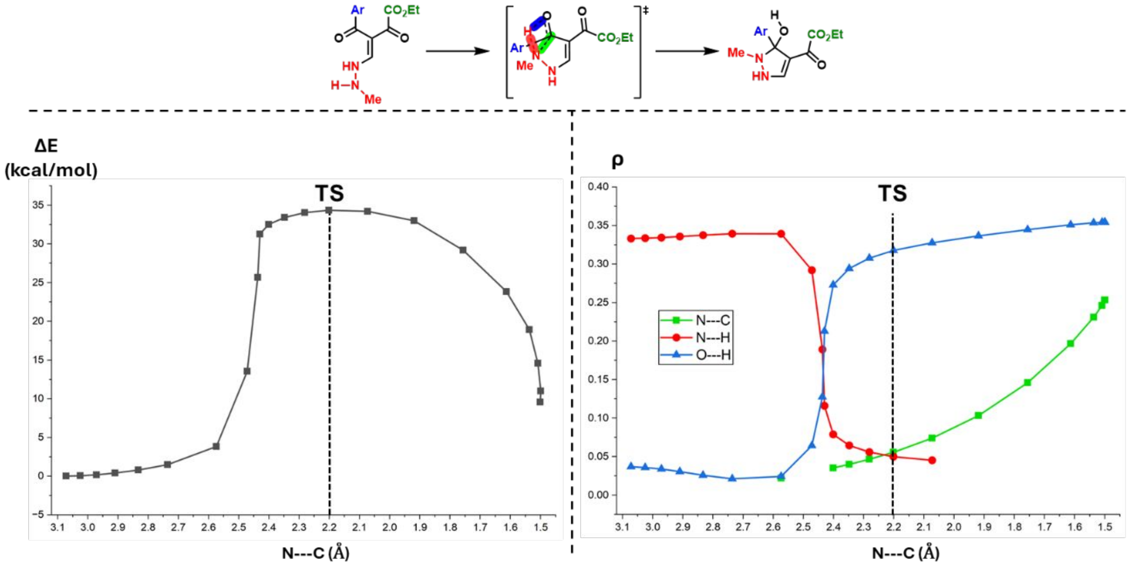

Figure S21. Cyclization step for intermediate **GS4A-E** with no PTC catalyzing the proton transfer. Left panel: Relative electronic energy variation (kcal/mol) connecting intermediate GS4A-E to intermediate **GS5A-E**, crossing the transition state **TS5A-E**. Right panel: Electron density variation for the N---C, N---H and O---H BCPs involved in the transition state. Computed at B3LYP-D3(BJ)/def2-TZVP, CPCM(water).

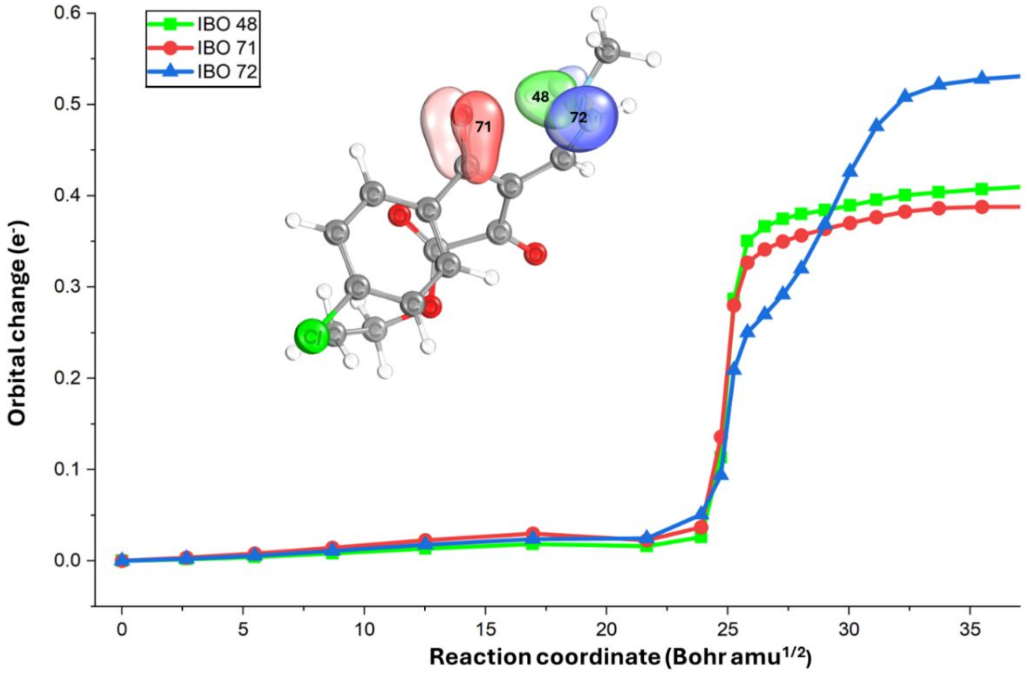

Figure S22. IBOs change along the reaction coordinate for TS5A-E with no PTC. Computed at B3LYP-D3(BJ)/def2-TZVP, CPCM(water).

The inclusion of one H_2O molecule as PTC drastically changes the profile of the transition state (Figure S23). The proton exchange occurs in a much later stage (with a N---C distance of 1.66 Å) which is basically at the point of the transition state. The O-H BCP starts to increase its electron density earlier than the N-H BPC's decrease, which offsets the synchronicity of the proton transfer a little towards the O-H bond formation. The N-C BCP on the other hand exists much earlier than the proton transfer occurs. At the TS point, all three BCPs have similar electron density which characterizes a synchronous overall transition state. The IBOs change shows the first process to occur is the N lp attacking the carbonyl (Figure S24), evidenced initial change of IBO 77. Next there is the evolution of the C=O π into a O lp, evidenced by the change of IBO 73, ending with a synchronized proton transfer from N to O, evidenced by a synchronized change of IBOs 50, 76 and 38.

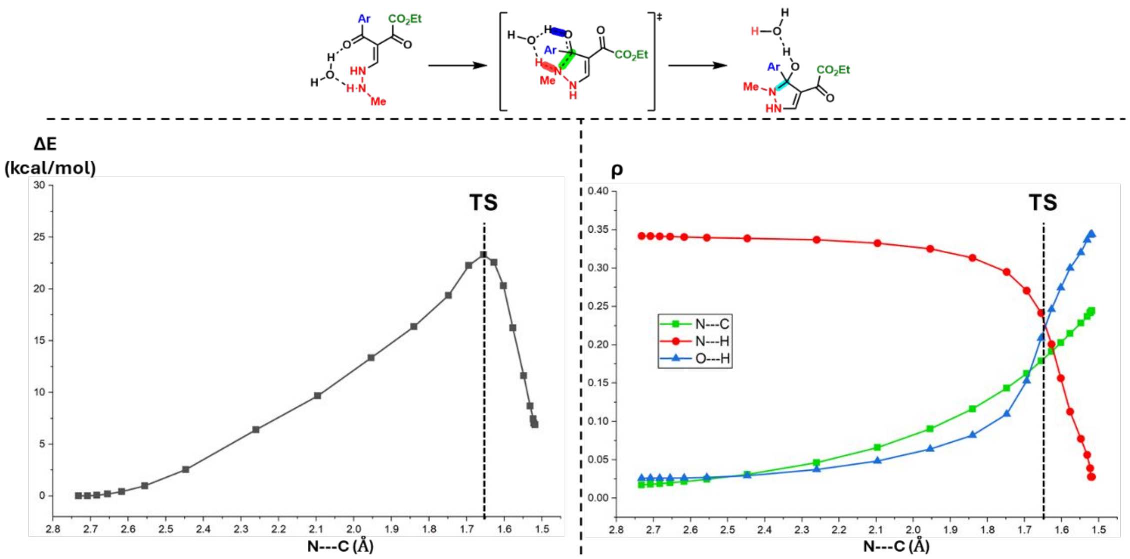

Figure S23. Cyclization step for intermediate **GS4A-E** with one H_2O molecule as PTC. Left panel: Relative electronic energy variation (kcal/mol) connecting intermediate GS4A-E to intermediate **GS5A-E**, crossing the transition state **TS5A-E**. Right panel: Electron density variation for the N---C, N---H and O---H BCPs involved in the transition state. Computed at B3LYP-D3(BJ)/def2-TZVP, CPCM(water).

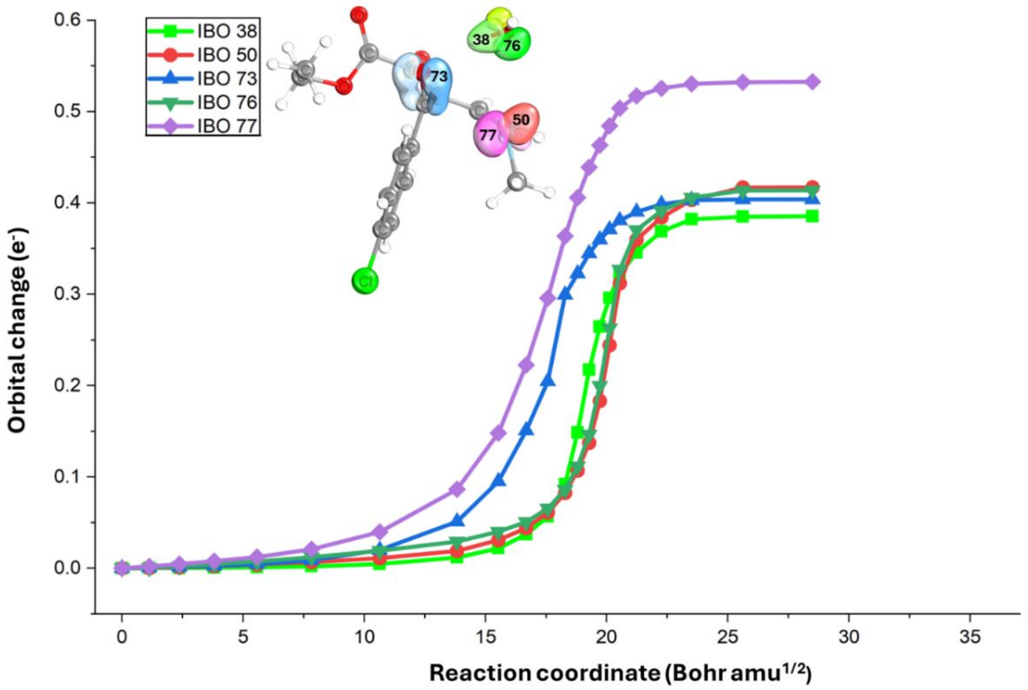

Figure S24. IBOs change along the reaction coordinate for TS5A-E with 1xH_2O as PTC. Computed at B3LYP-D3(BJ)/def2-TZVP, CPCM(water).

Upon inclusion a second water molecule as PTC, the profile of the electron density variation of the BCPs changes little compared to a single H_2O molecule (compare Figures S23 and S25). We note a slightly larger offset of the proton transfer synchronicity towards the O-H bond formation, evidenced by the increased height at which lines blue and red crosses. The IBO change also shows a similar profile (compared Figures S24 and S26).

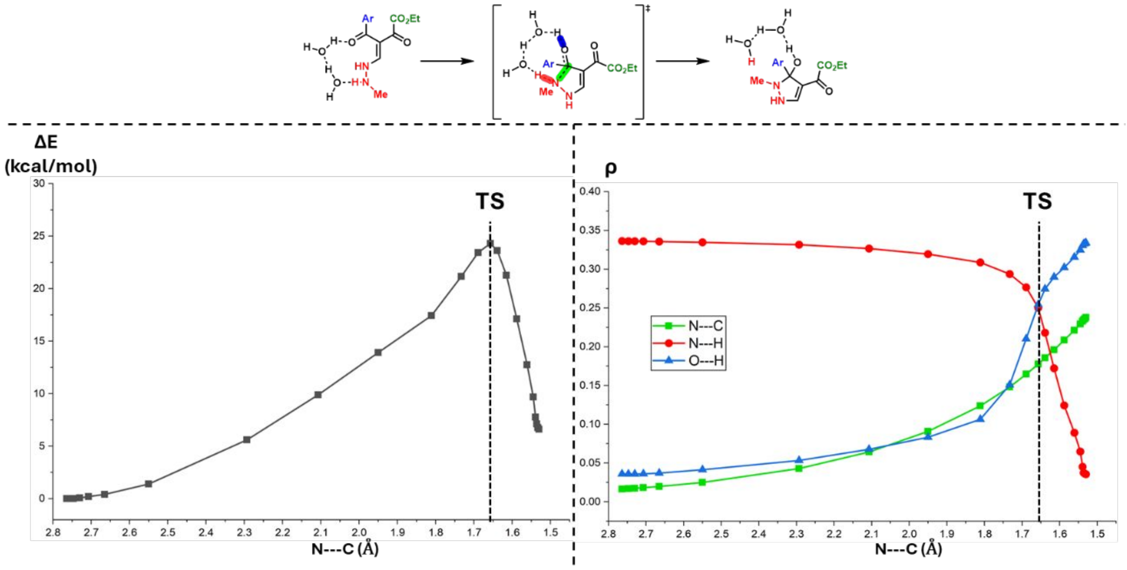

Figure S25. Cyclization step for intermediate **GS4A-E** with two H_2O molecule as PTC. Left panel: Relative electronic energy variation (kcal/mol) connecting intermediate GS4A-E to intermediate **GS5A-E**, crossing the transition state **TS5A-E**. Right panel: Electron density variation for the N---C, N---H and O---H BCPs involved in the transition state. Computed at B3LYP-D3(BJ)/def2-TZVP, CPCM(water).

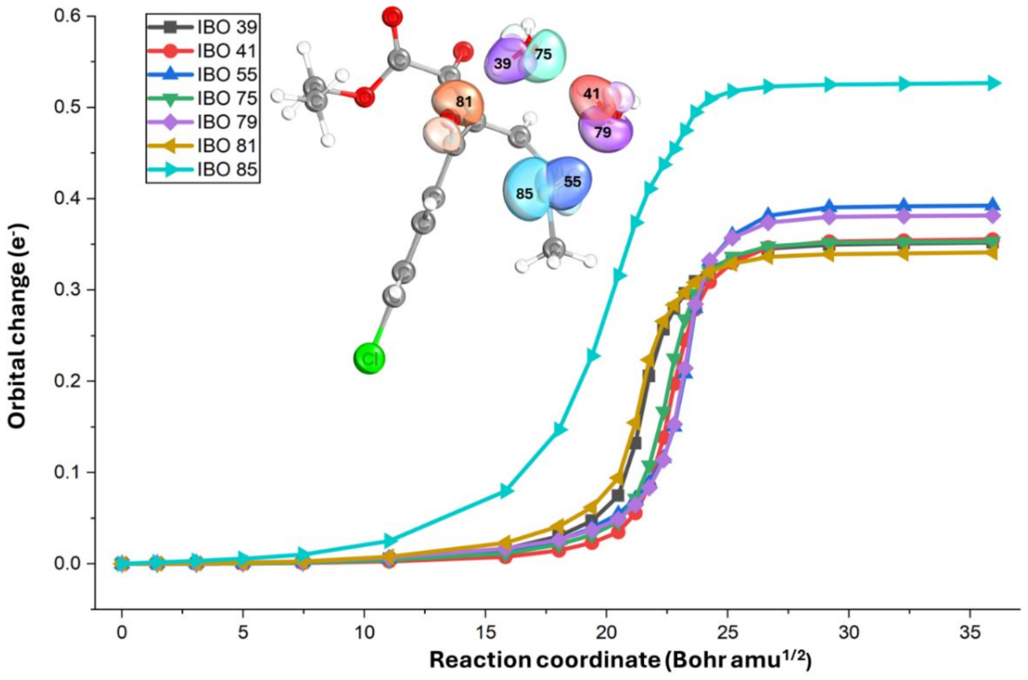

Figure S26. IBOs change along the reaction coordinate for TS5A-E with $2xH_2O$ as PTC. Computed at B3LYP-D3(BJ)/def2-TZVP, CPCM(water).

When the PTC is one NH_3 molecule, the reaction profile changes drastically (Figure S25). We note the N-H BCP starts do decrease its electron density much earlier than the O-H increase, suggesting NH deprotonation occurs much sooner than O-H protonation, that is, a very asynchronous process. The IBOs change (Figure S26) shows that the first orbitals to change are 82 and 50, involved in the NH deprotonation. Next, there is the interaction of N lp with the carbonyl, evidence by the change of IBO 78, followed by the transfer of a proton to the oxygen, evidence by the late change of IBO 55. These results support a very asynchronous process.

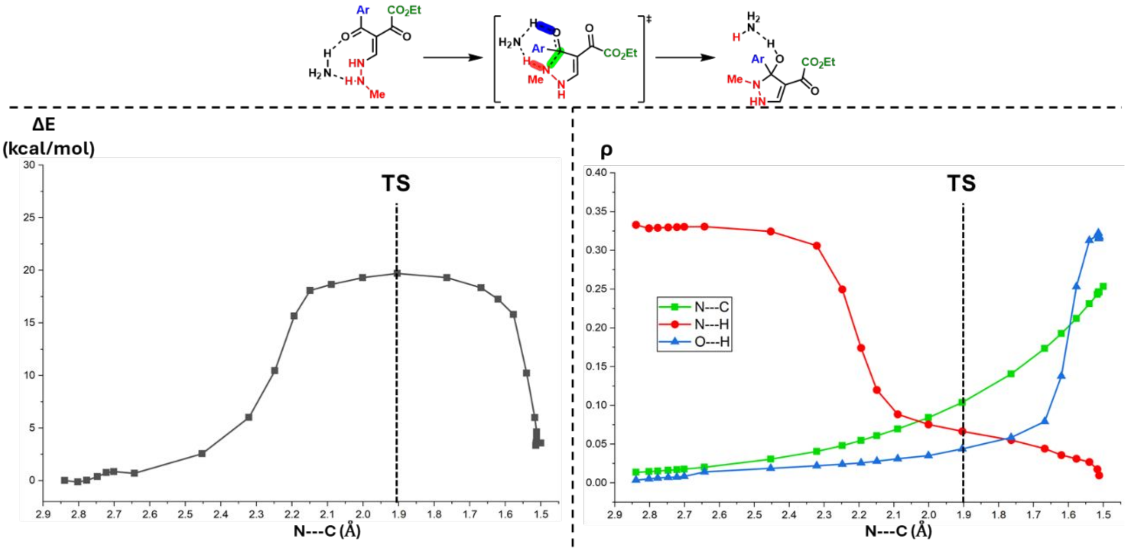

Figure S27. Cyclization step for intermediate **GS4A-E** with one NH_3 molecule as PTC. Left panel: Relative electronic energy variation (kcal/mol) connecting intermediate GS4A-E to intermediate **GS5A-E**, crossing the transition state **TS5A-E**. Right panel: Electron density variation for the N---C, N---H and O---H BCPs involved in the transition state. Computed at B3LYP-D3(BJ)/def2-TZVP, CPCM(water).

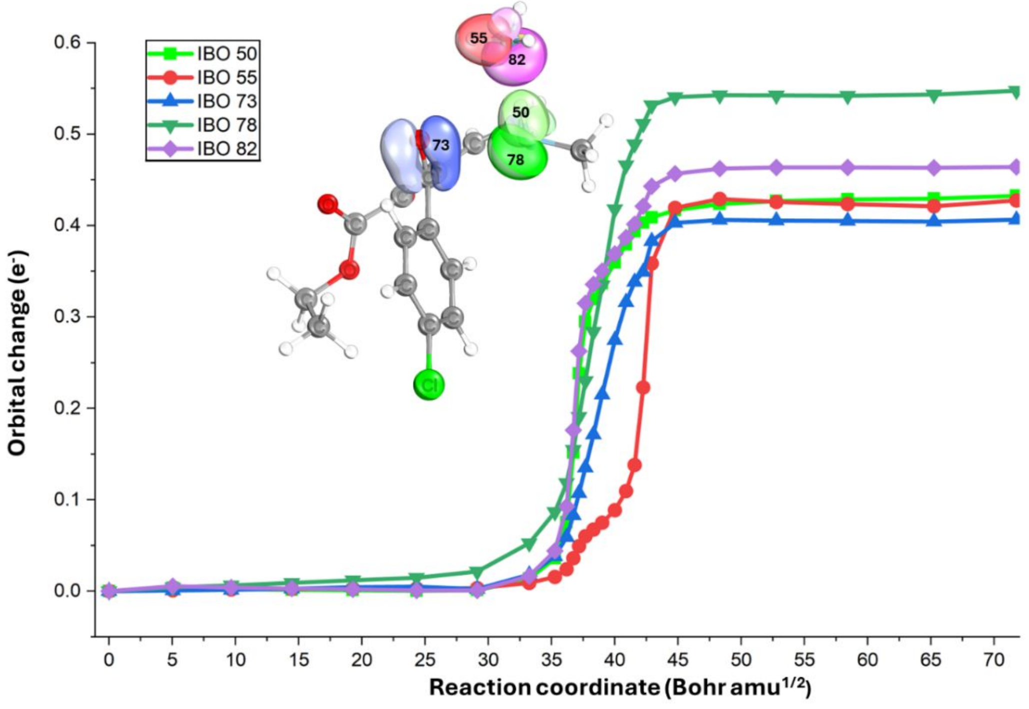

Figure S28. IBOs change along the reaction coordinate for TS5A-E with NH_3 as PTC. Computed at B3LYP-D3(BJ)/def2-TZVP, CPCM(acetonitrile).

Finally, the inclusion of one AcOH molecule as PTC also results in a asynchronous proton transfer step, however, unlike NH_3, the first step is the

protonation of the carbonyl oxygen, evidenced by the much earlier increase of the O-H BCP electron density (Figure S29). Therefore, this asynchronicity leans towards the 2xH_2O molecule profile, which agrees with their similar product ratios (Table 1 of main text). IBO changes show the orbitals involved in the oxygen protonation (80 and 40) changes earlier than the orbitals involved in the NH deprotonation (54 and 94) (Figure S30).

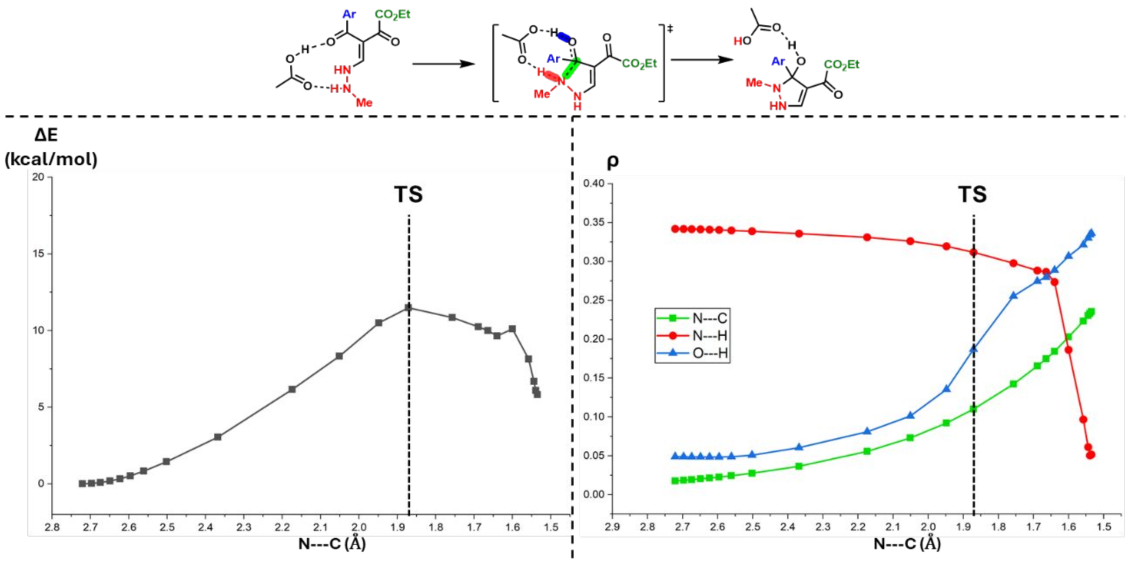

Figure S29. Cyclization step for intermediate **GS4A-E** with one AcOH molecule as PTC. Left panel: Relative electronic energy variation (kcal/mol) connecting intermediate GS4A-E to intermediate **GS5A-E**, crossing the transition state **TS5A-E**. Right panel: Electron density variation for the N---C, N---H and O---H BCPs involved in the transition state. Computed at B3LYP-D3(BJ)/def2-TZVP, CPCM(water).

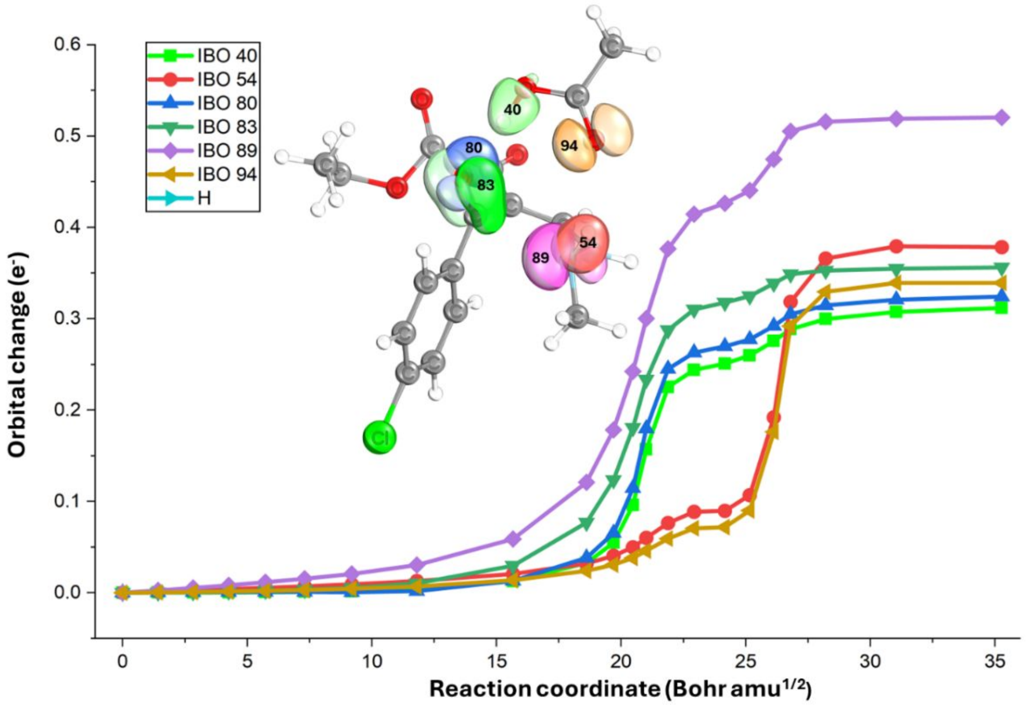

Figure S30. IBOs change along the reaction coordinate for TS5A-E with AcOH as PTC. Computed at B3LYP-D3(BJ)/def2-TZVP, CPCM(acetonitrile).

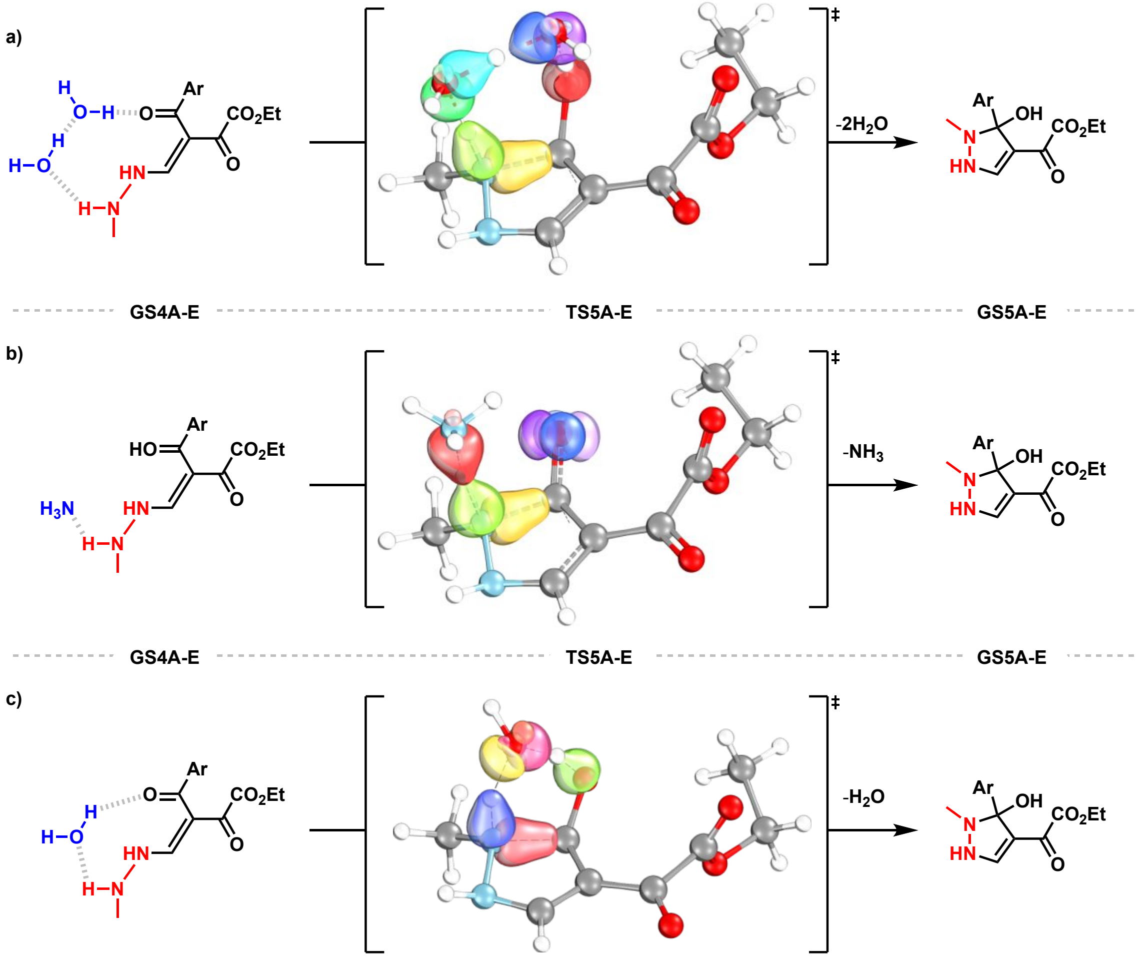

Figure S31. Comparison of Intrinsic Bond Orbital (IBO) involvement in the cyclization transition state with (**a**) 2xH_2O (synchronous, 7 IBOs), (**b**) NH_3 (asynchronous, 5 IBOs) and (**c**) 1xH_2O (synchronous, 5 IBOs) as PTC. Note the different orbital overlap between 1 NH_3 and 1 H_2O.

S7.Interaction Region Indicator (IRI) for the cyclization transition states.

TS5A-E

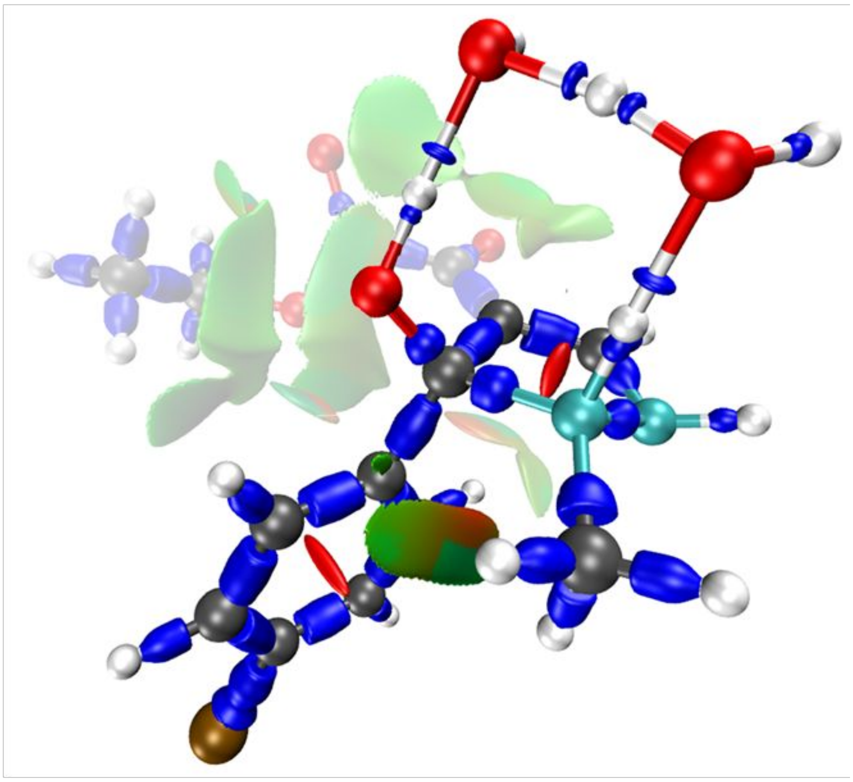

TS5A-Z

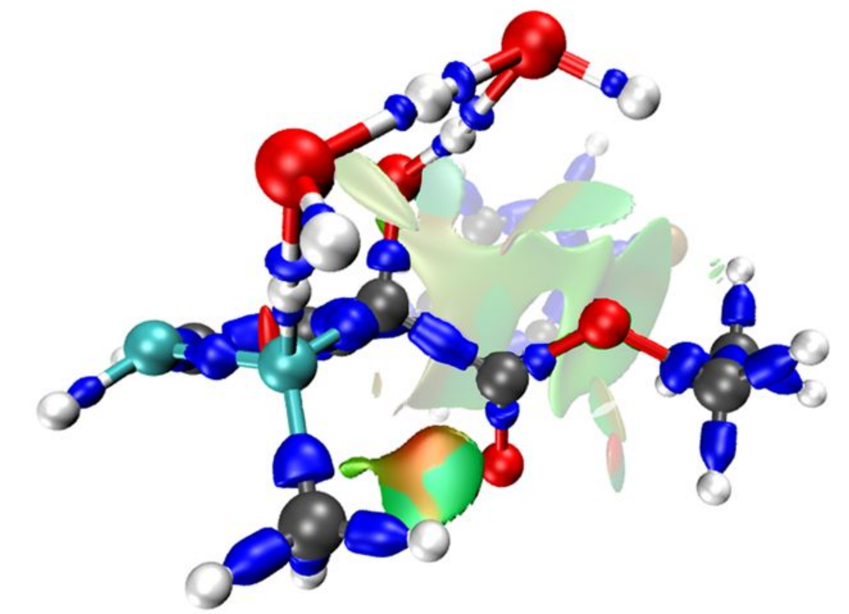

Figure S32. Interaction Region Indicator (IRI) of transition states **TS5A-E** and **TS5A-Z**. The interaction between the nucleophilic moiety NHMe and the carbonyl substituent Ar (**TS5A-E**) or CO_2Et (**TS5A-Z**) is highlighted. The blue regions indicate strong, covalent-like, interactions. Green areas indicate NCI interactions. Isovalue: 1.0

We analyzed the strong, covalent-like, and weak interactions employing the IRI analysis. We TS5A-E and TS5A-Z because these transitions states have a methyl approaching an aryl group (on TS5A-E) or a carboxy methyl group (on TS5A-Z). The results demonstrate that the methyl group has a similar interaction with both carbonyl substituents (aryl or carboxy methyl). Therefore, sterics should not be important for the reactivity of these transition states.

S8.Intrinsic Reaction Coordinate (IRC) for the cyclization transition states

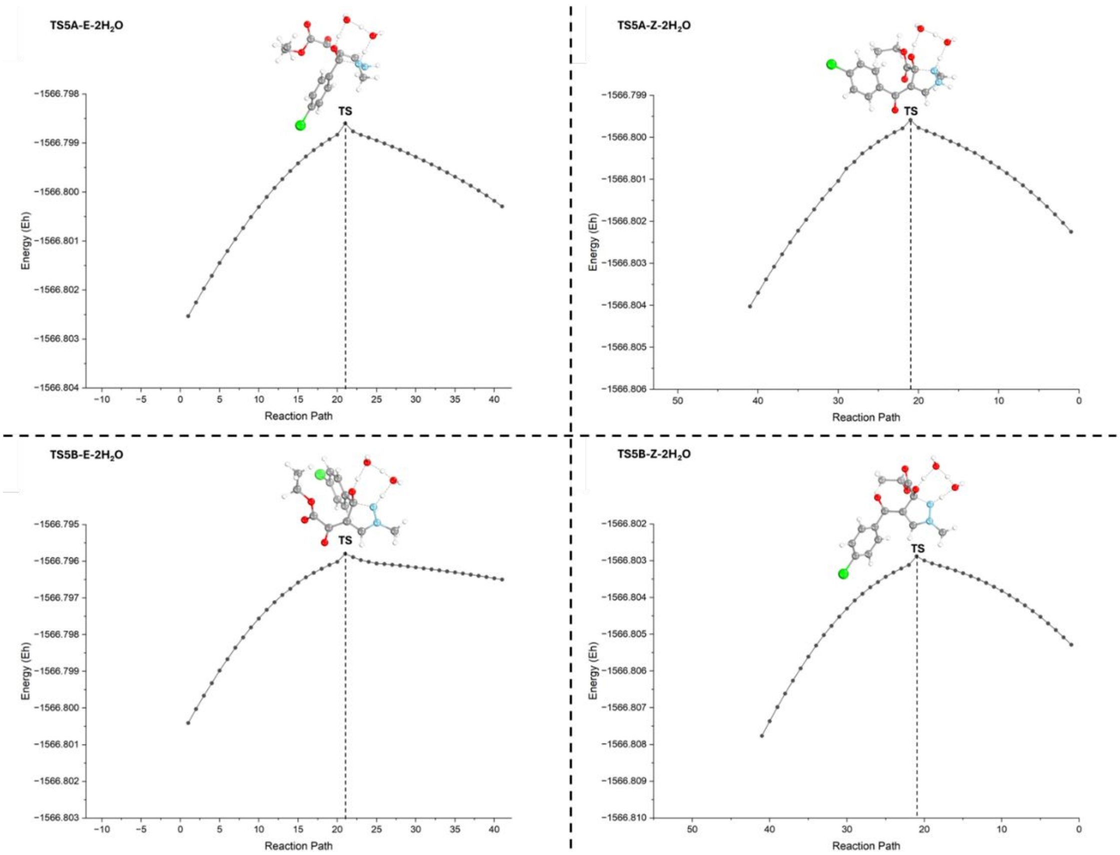

Figure S33. Intrinsic Reaction Coordinate (IRC) computed for the four cyclization transition states with 2xH_2O as PTC, computed with ωB97X-D3(BJ)/def2-TZVP, CPCM(acetonitrile).

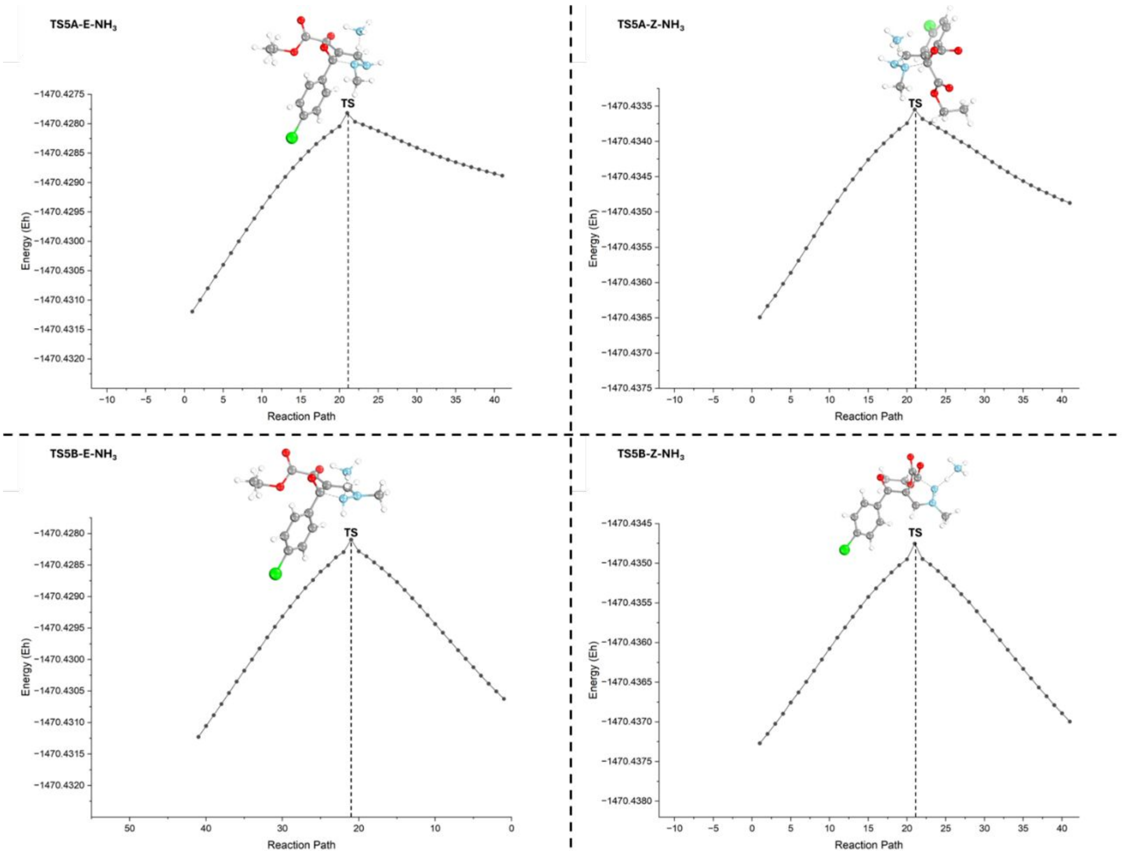

Figure S34. Intrinsic Reaction Coordinate (IRC) computed for the four cyclization transition states with NH_3 as PTC, computed with ωB97X-D3(BJ)/def2-TZVP, CPCM(acetonitrile).

S9.Charge Variation Analysis

We computed the IBO charges for each structure of the NEB path connecting the reactants GS4 to the products GS5. We analyzed the charge variation for the most important atoms involved in the transition states: Nucleophilic nitrogen, carbonyl carbon, carbonyl oxygen and the core atoms of the PTCs, nitrogen for NH_3 and the oxygens for H_2O. Then, we plotted this charger as a function of the nucleophilic nitrogen and carbonylic carbon distance. For the case of 2 H_2O molecules as PTC, we report the average charge between both oxygens for clarity. Lines with hollow circles refer to the systems using NH_3 as PTC, filled circles refer to systems with 2 H_2O as PTC.

In this analysis, we note that the negative charge of the nucleophilic nitrogen decreases across the path (blue lines). In contrast, the negative charge of the carbonyl carbon and oxygen increases, indicating charge transfer among these atoms. We note charge variation peaks on the nucleophilic nitrogen and carbonylic oxygen, which indicates the point where the proton is transferred. Upon removal of the proton, the negative charge of the nitrogen increases while upon receival of this proton, the negative charge of the carbonylic oxygen decreases, stabilizing the system's charge. We note that the charge variation of the H_2O oxygens (green lines filled circles) change just slightly which is a consequence of the synchronous proton transfer.

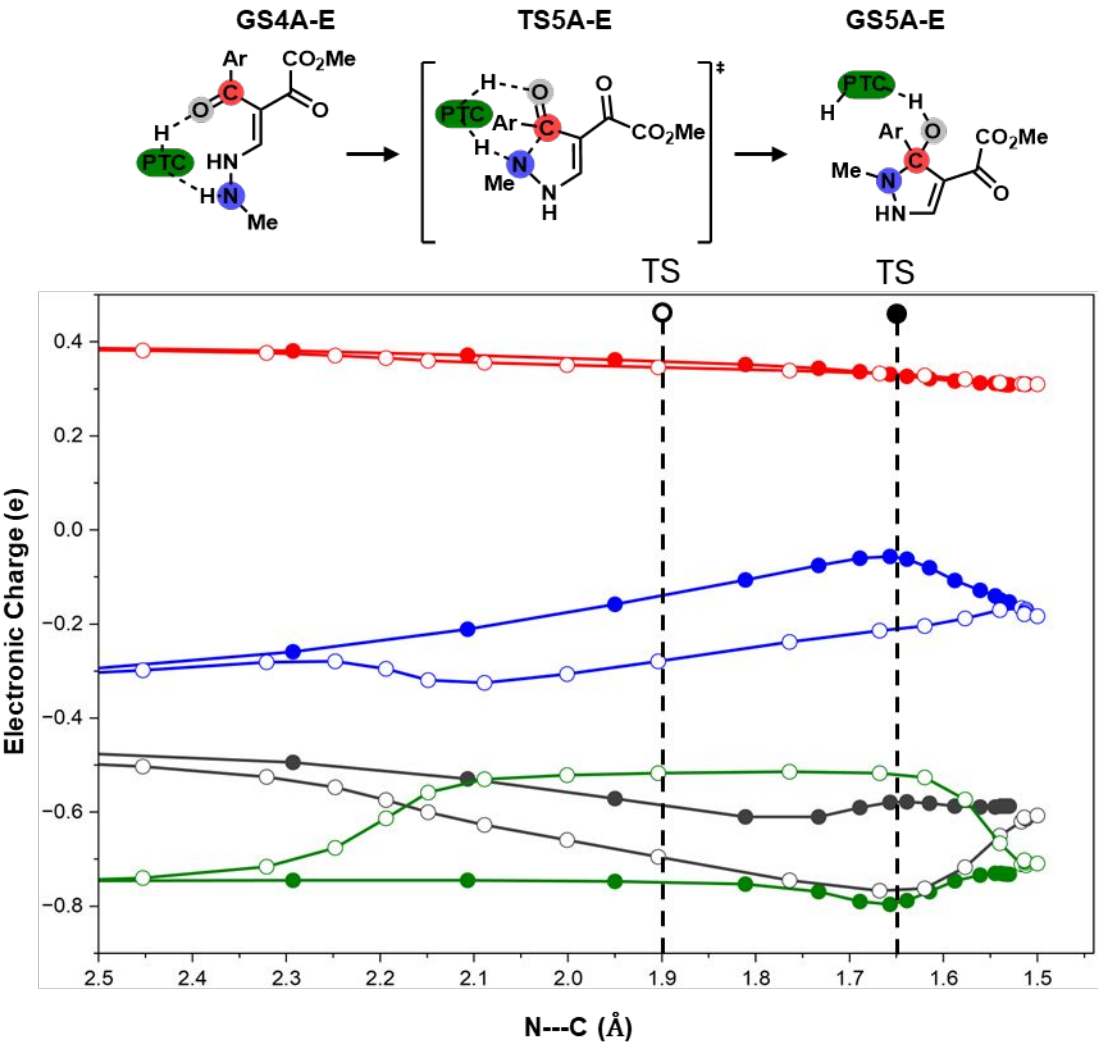

Figure S35. IBO charge variation of the most important atoms (C: red, N: blue, O: grey, PTC: green, as shown above) for the reaction path connecting the reactant **GS4A-E** to the cyclization product **GS5A-E** considering both PTCs: NH_3 (hollow circles) and 2xH_2O (filled circles). For the PTC 2xH_2O, we report the average charge of both oxygens. Computed at B3LYP-D3BJ/def2-TZVP, CPCM (water/acetonitrile).

In contrast, for NH_3, we note an earlier decrease in the nitrogen's negative charge (blue lines, hollow circles), at the same time the negative charge of the NH_3 nitrogen (green line hollow circles) decrease, indicating proton transfer. We also note a large charge accumulation on the carbonylic oxygen when NH_3 is the PTC (black lines hollow circles) compared to H_2O (black lines filled circles)

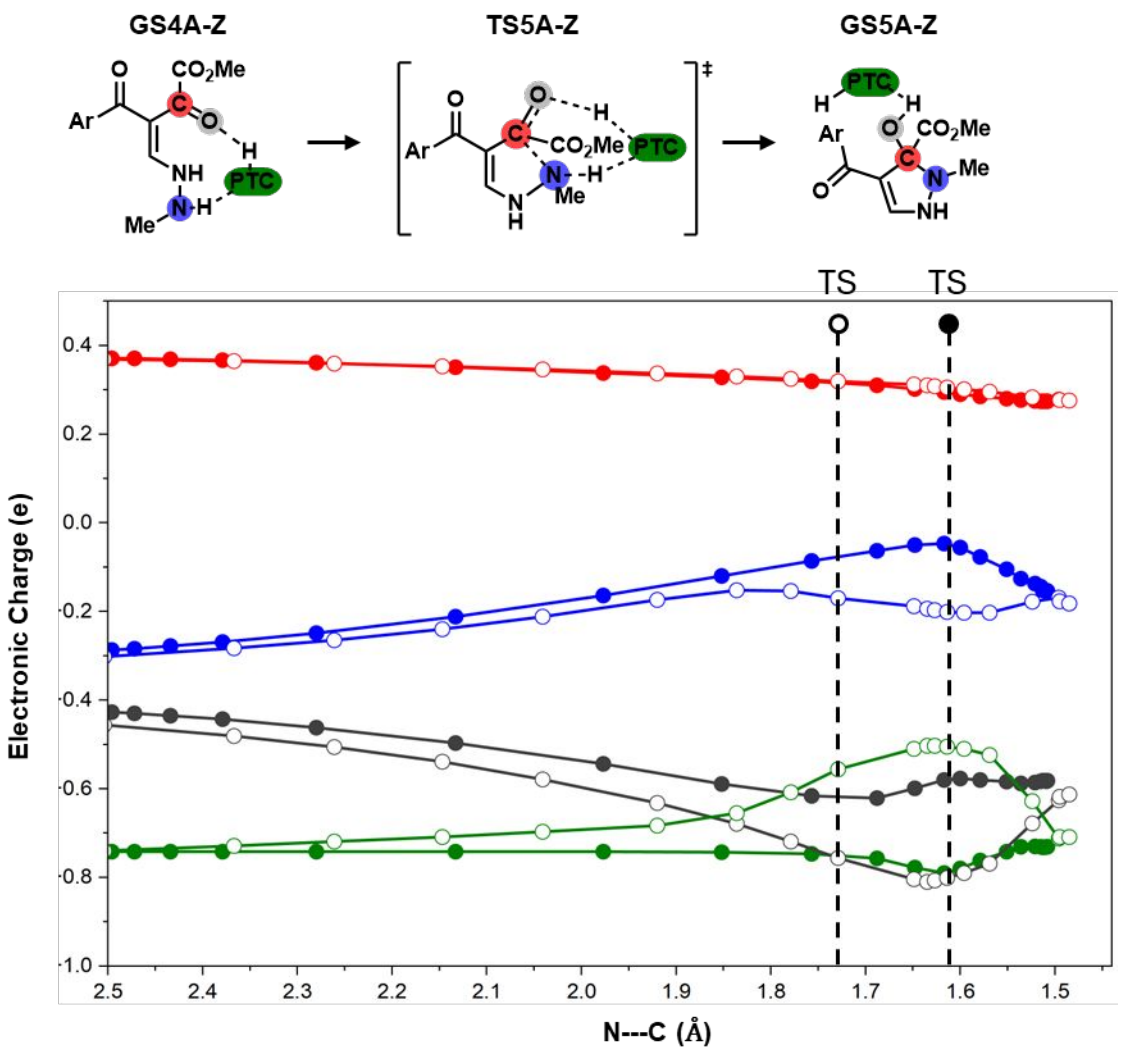

Figure S36. IBO charge variation of the most important atoms (C: red, N: blue, O: grey, PTC: green, as shown above) for the reaction path connecting the reactant **GS4A-Z** to the cyclization product **GS5A-Z** considering both PTCs: NH_3 (hollow circles) and $2xH_2O$ (filled circles). For the PTC $2xH_2O$, we report the average charge of both oxygens. Computed at B3LYP-D3BJ/def2-TZVP, CPCM(water/acetonitrile).

S10.Kinetic investigation of different BEDs

We computed the cyclization transition states for the intermediates originating from the reaction between tert-butyl hydrazine and the BEDs reported by Rosa and coworkers in 2008.[17] Later, these systems were investigated computationally by Rosada and coworkers.[18] In order to find the cyclization transition states, Rosada and coworkers protonated the oxygen of the carbonyl that is attacked. This approach can be justified when considering that the tert-butyl hydrazine was used in its salt form with HCl ($H_2NNHCMe_3$ · HCl). However, the computational results from Rosada demonstrate that cyclization on the carbonyl attached to a para substituted aryl ring is favored over the competing carbonyl with a carboxy ethyl substituent. This contradicts

the experimental findings where only the product originating from the cyclization on the CO_2Et attached carbonyl is found.

We instead modeled these cyclization transition states employing one MeOH as Proton Transfer Catalyst which was the solvent used in these reactions. The results (Figures S35 and 36) demonstrate that when a PTC is considered, cyclization on the CO_2Et attached carbonyl is always favored, agreeing with the experimental results

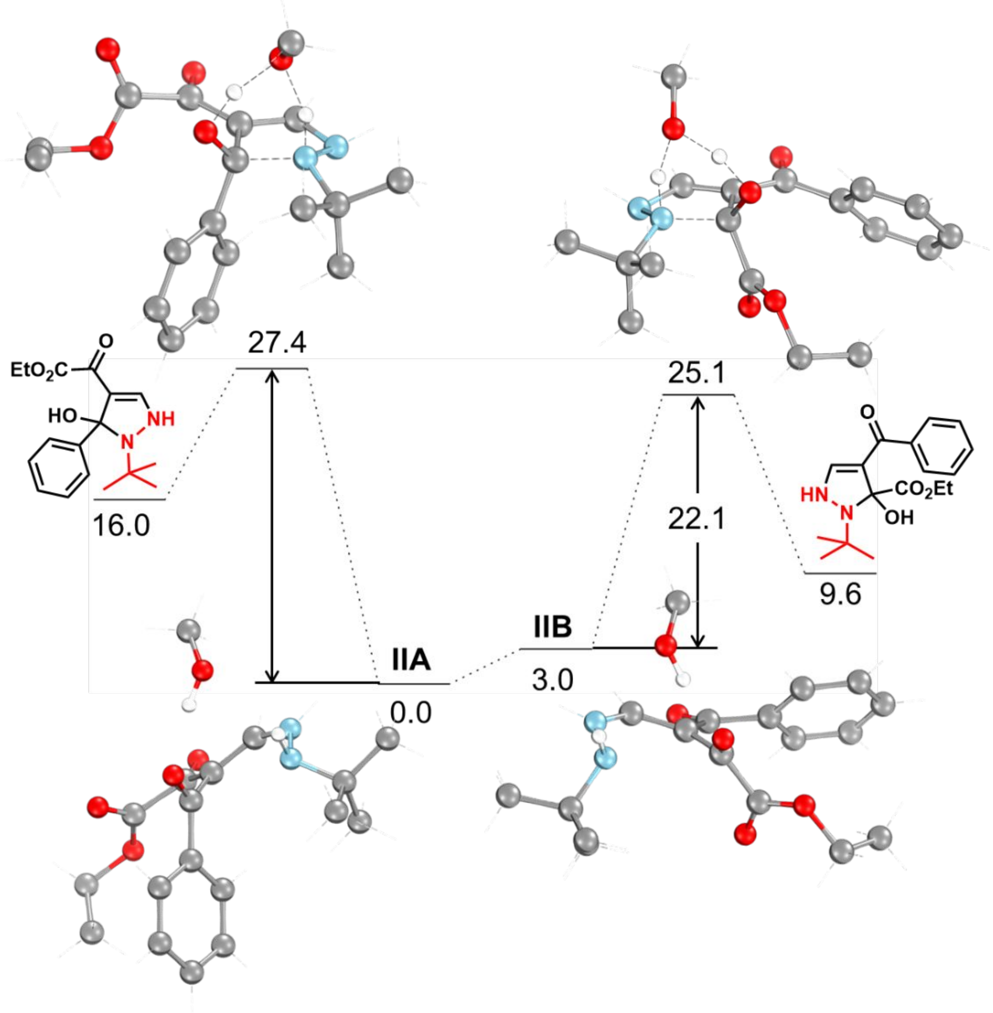

Figure S37. Cyclization transition states and barriers for the aryl substituted carbonyl (left) and carboxy ethyl substituted carbonyl (right). Computed with ωB97X-D3(BJ)/def2-TZVP, CPCM(methanol)

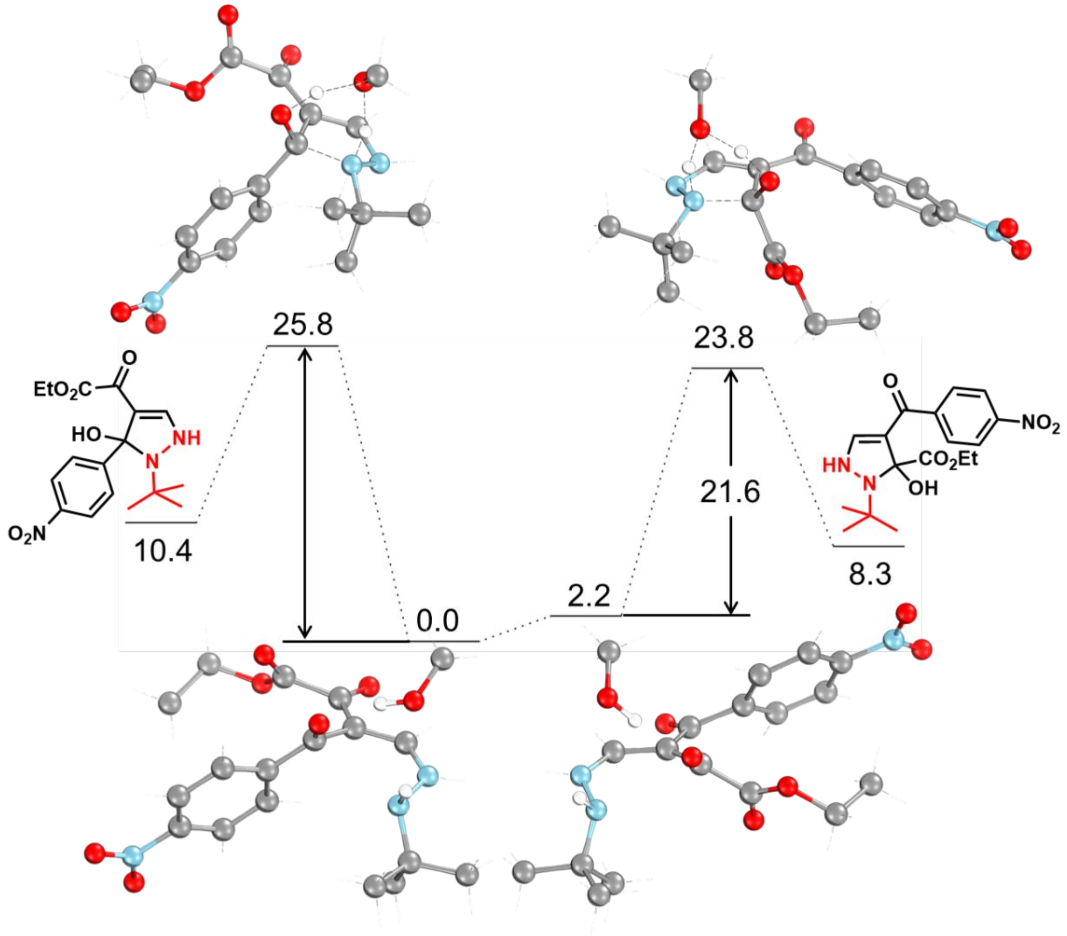

Figure S38. Cyclization transition states and barriers for the intermediates with 4-NO_2-aryl (left) and carboxy ethyl (right). Computed with ωB97X-D3(BJ)/def2-TZVP, CPCM(methanol)

S11. References

(1) Poletto, J.; da Silva, M. J. V.; Pianoski, K. E.; Willig, J. C. M.; Rosa, F. A. Regiodivergent Synthesis of 3,4- and 4,5-Disubstituted N-Methylpyrazoles from 4-Acyl-1 H-Pyrrole-2,3-Dione and Methylhydrazine. *J. Org. Chem.* **2022**, *87* (13), 8544–8550.

(2) Perrin, D. D.; Armarego, W. L. F. *Purification of Laboratory Chemicals*, 3rd ed.; Pergamon Press: New York, 1996.

(3) Rosa, F. A.; Machado, P.; Rossatto, M.; Vargas, P. S.; Bonacorso, H. G.; Zanatta, N.; Martins, M. A. P. N- and C-Acylation in β-Enamino Ketones: Structural Effects on Regiocontrol. *Synlett* **2007**, No. 20, 3165–3171.

(4) Pracht, P.; Bohle, F.; Grimme, S. Automated Exploration of the Low-Energy Chemical Space with Fast Quantum Chemical Methods. *Phys. Chem. Chem. Phys.* **2020**, *22* (14), 7169–7192.

(5) Bannwarth, C.; Caldeweyher, E.; Ehlert, S.; Hansen, A.; Pracht, P.; Seibert, J.; Spicher, S.; Grimme, S. Extended Tight-Binding Quantum Chemistry Methods. *Wiley Interdiscip. Rev. Comput. Mol. Sci.* **2021**, *11* (2), e1493.

(6) Grimme, S.; Brandenburg, J. G.; Bannwarth, C.; Hansen, A. Consistent Structures and Interactions by Density Functional Theory with Small Atomic Orbital Basis Sets. *J. Chem. Phys.* **2015**, *143* (5), 054107–054127.

(7) Barone, V.; Cossi, M. Quantum Calculation of Molecular Energies and Energy Gradients in Solution by a Conductor Solvent Model. *J. Phys. Chem. A* **1998,** 102 (11), 1995–2001.

(8) Neese, F. The ORCA Program System. *Wiley Interdiscip. Rev. Comput. Mol. Sci.* **2012**, *2* (1), 73–78.

(9) Neese, F. Software Update: The ORCA Program System—Version 6.0. *Wiley Interdiscip. Rev. Comput. Mol. Sci.* **2025**, *15* (2), e70019.

(10) Chai, J. Da; Head-Gordon, M. Systematic Optimization of Long-Range Corrected Hybrid Density Functionals. *J. Chem. Phys.* **2008**, *128* (8), 084106-1–084106-15.

(11) Johnson, E. R.; Becke, A. D. A Post-Hartree-Fock Model of Intermolecular Interactions. *J. Chem. Phys.* **2005**, *123* (2), 024101–024109.

(12) Weigend, F.; Ahlrichs, R. Balanced Basis Sets of Split Valence, Triple Zeta Valence and Quadruple Zeta Valence Quality for H to Rn: Design and Assessment of Accuracy. *PhysChemChemPhys.* **2005**, *7* (18), 3297–3305.

(13) Spiegel, M.; Gamian, A.; Sroka, Z. A Statistically Supported Antioxidant Activity DFT Benchmark—The Effects of Hartree–Fock Exchange and Basis Set Selection on Accuracy and Resources Uptake. *Molecules* **2021**, 26, 5058-5080.

(14) Rayne, S.; Forest, F. A comparative examination of density functional performance against the ISOL24/11 isomerization energy benchmark. *Comput.Theor.Chem.* **2016**, 1090, 147-152.

(15) Li, H.; Kermani, M. M.; Ottochian, A.; Crescenzi, O.; Janesko, B. G.; Truhlar, D. G.; Scalmani, G.; Frisch, M. J.; Ciofini, I.; Adamo, C. Modeling Multi-Step Organic Reactions: Can Density Functional Theory Deliver Misleading Chemistry? *J. Am. Chem. Soc.* **2024**, 146, 6721−6732.

(16) Sharma, J.; Champagne, P. A. Benchmark of density functional theory methods for the study of organic polysulfides. *J Comput Chem*. **2022**, 43, 2131-2138.

(17) Rosa, F. A.; Machado, P.; Vargas, P. S.; Bonacorso, H.G.; Zanatta, N.; Martins, M. A. P. Straightforward and Regiospecific Synthesis of Pyrazole-5-carboxylates from Unsymmetrical Enaminodiketones. *Synlett*, **2008**, 11, 1673-1678.

(18) Rozada, T. C.; da Silva, M. J. V.; Gonçalves, D. S.; Martins, M. A. P.; Pontes, R. M.; Gauze, G. F.; Basso, E. A.; Rosa, F. A. Theoretical Aspects of the Unexpected Regiospecific Synthesis of Pyrazole-5-Carboxylates from Unsymmetrical Enaminodiketones. *Struct. Chem.* **2015**, *26* (4), 1007–1011.

S12. ^{1}H and ^{13}C{^{1}H} NMR Spectra for *N*-methylpyrazoles

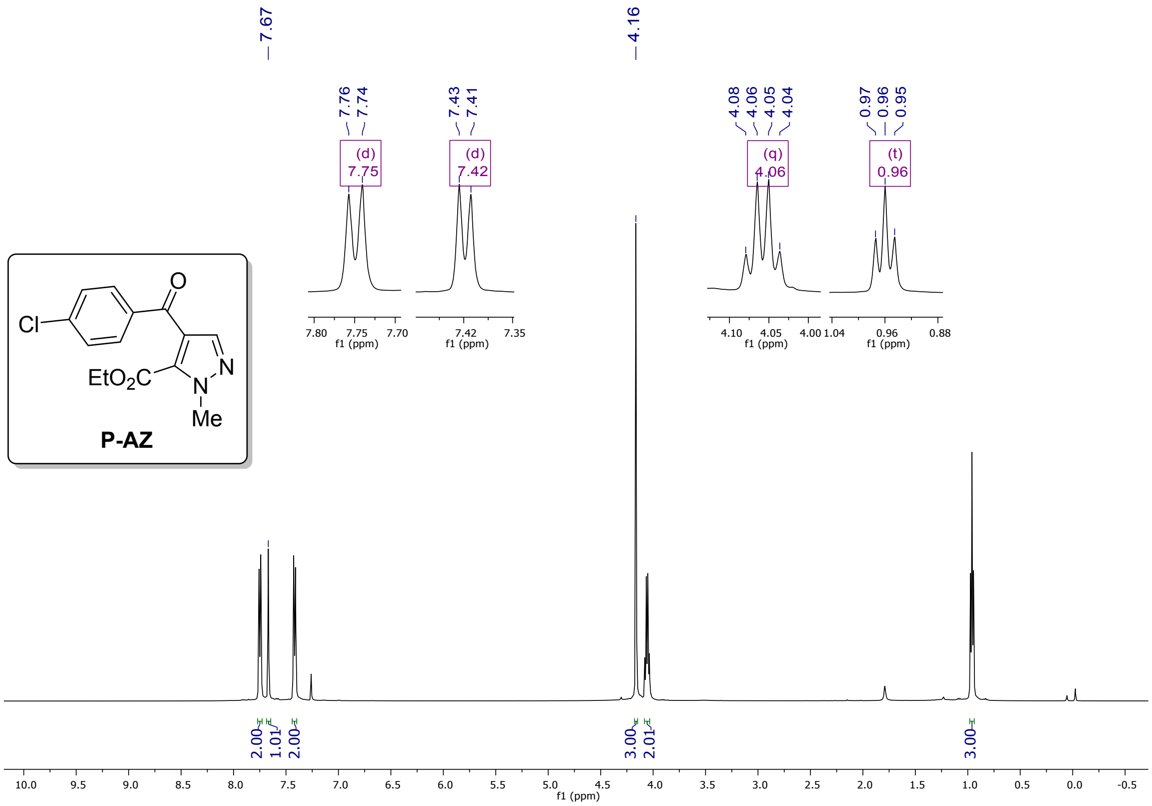

Figure S39 – ^{1}H NMR spectrum of compound **P-AZ** in $CDCl_3$ at 500.13 MHz

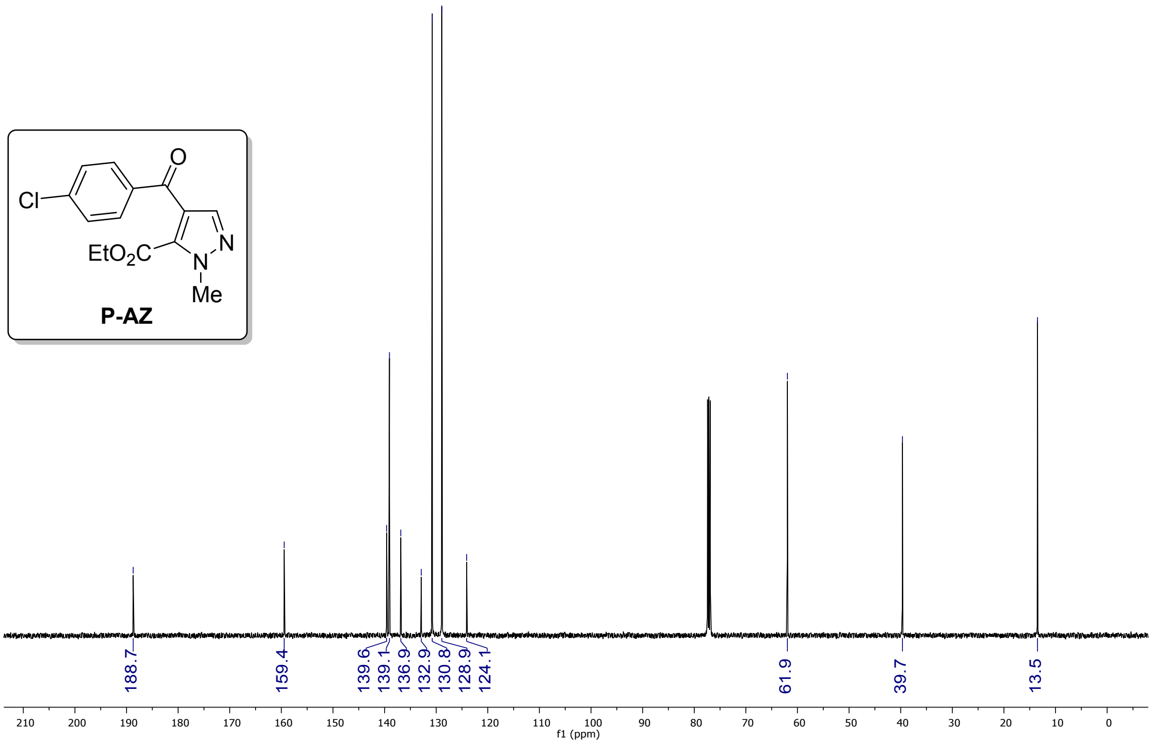

Figure S40 – $^{13}C\{^{1}H\}$ NMR spectrum of compound **P-AZ** in $CDCl_3$ at 125.77 MHz

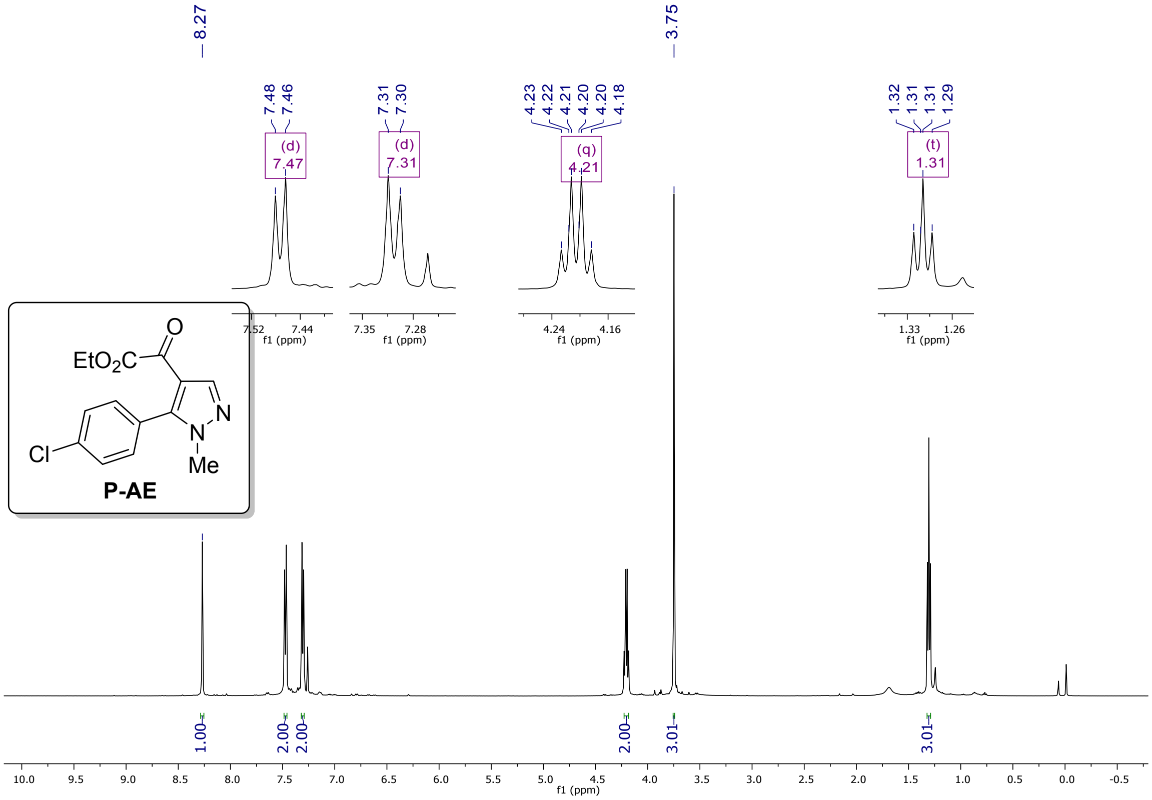

Figure S41 – ^{1}H NMR spectrum of compound **P-AE** in $CDCl_3$ at 500.13 MHz

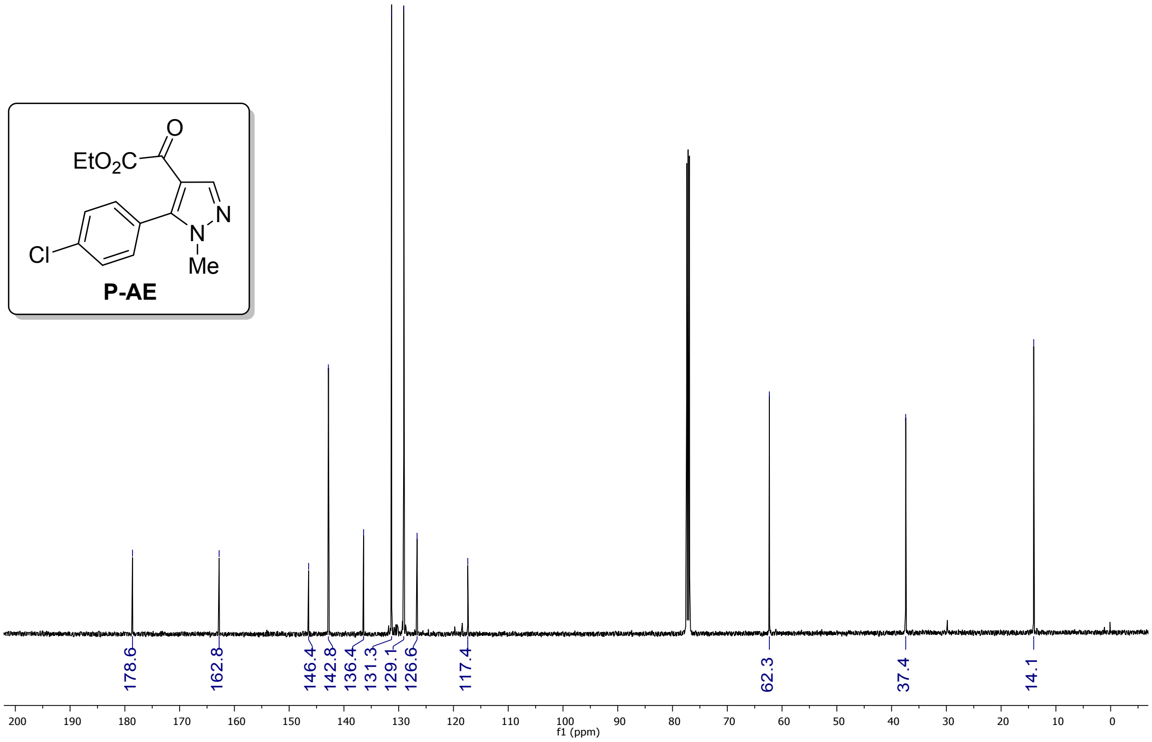

Figure S42 – ^{13}C{^{1}H} NMR spectrum of compound **P-AE** in $CDCl_3$ at 125.77 MHz

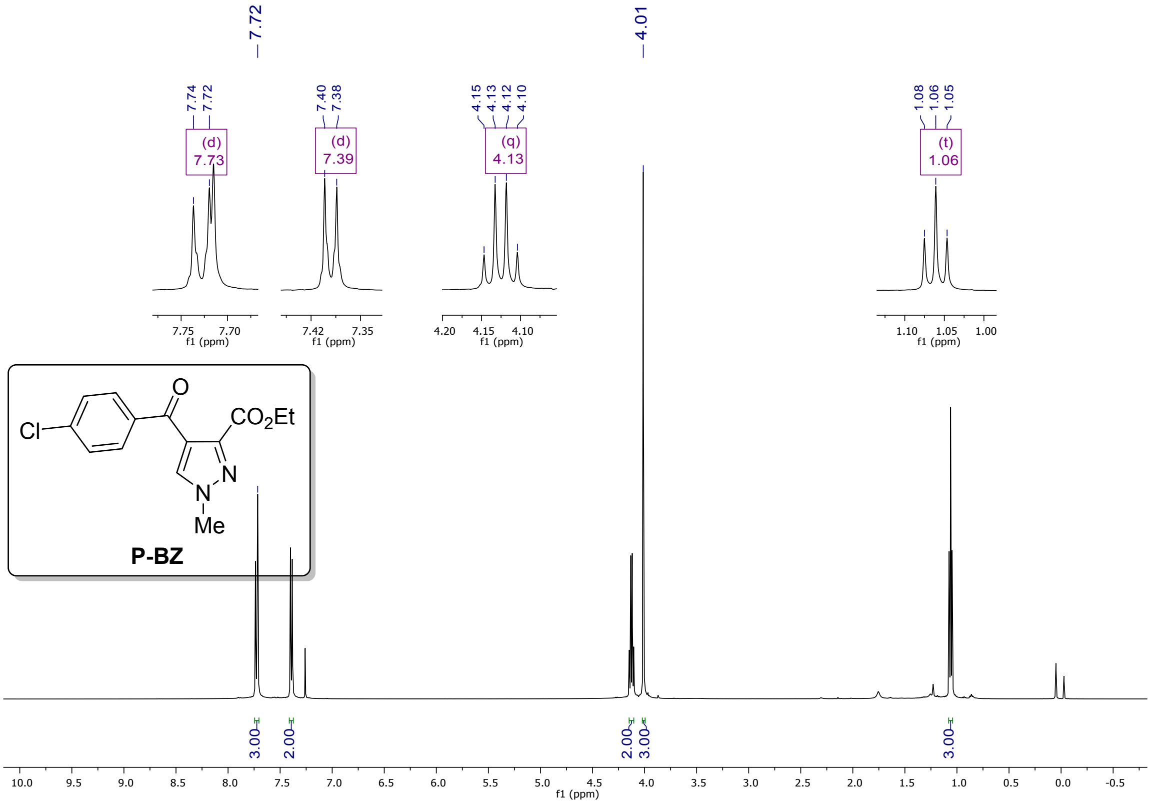

Figure S43 – ^{1}H NMR spectrum of compound **P-BZ** in $CDCl_3$ at 500.13 MHz

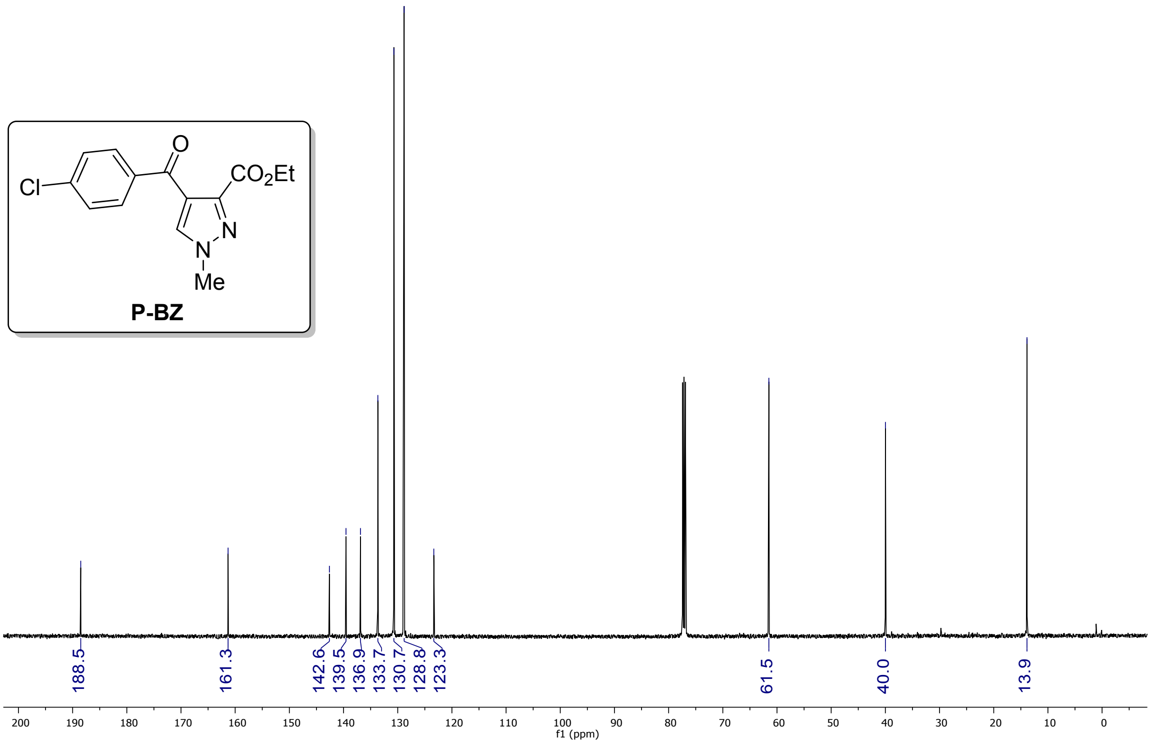

Figure S44 – $^{13}C\{^1H\}$ NMR spectrum of compound **P-BZ** in $CDCl_3$ at 125.77 MHz

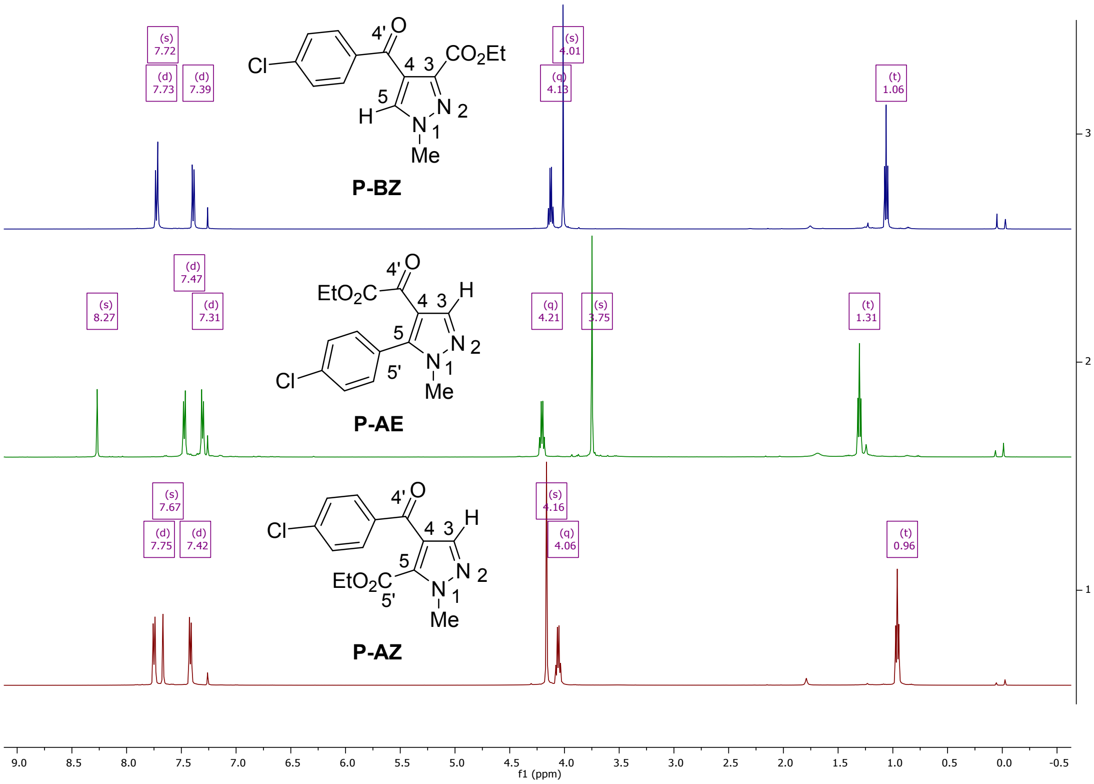

Figure S45 –^{1}H NMR spectra of compounds **P-BZ**, **P-AE**, and **P-AZ** ($CDCl_3$, 500.13 MHz)

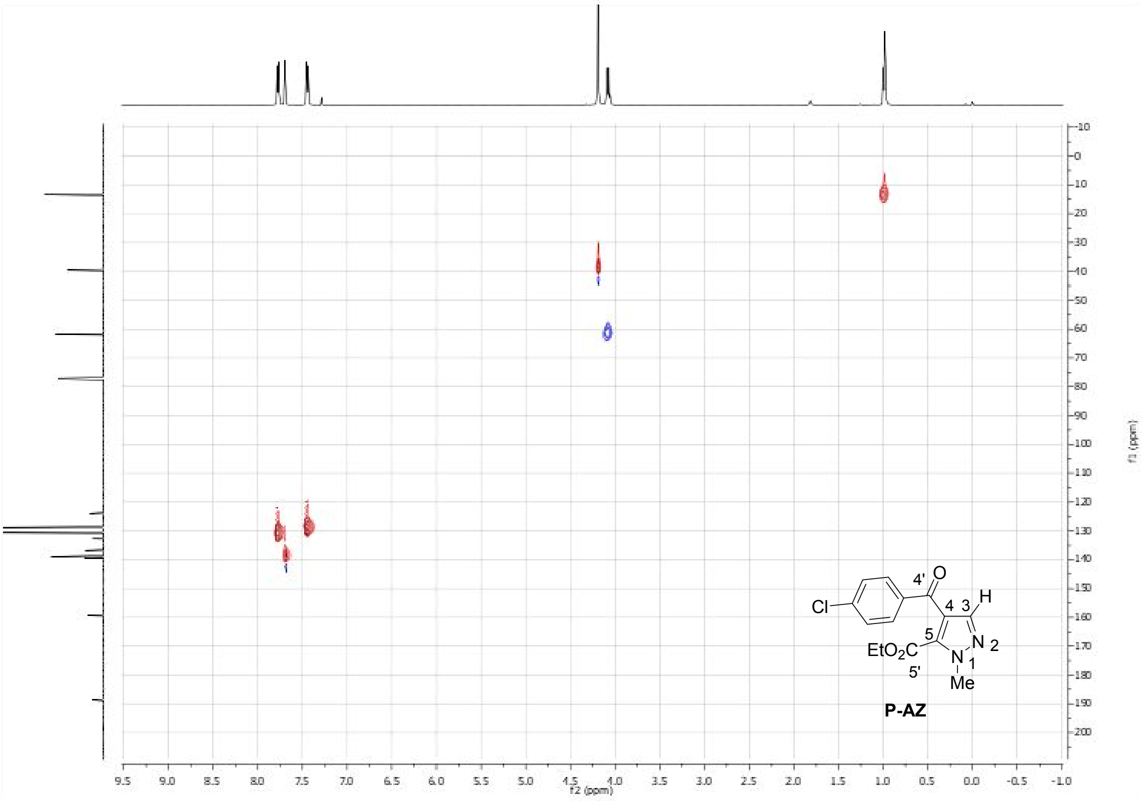

Figure S46 – HSQC NMR spectrum of **P-AZ** ($CDCl_3$, 500.13 x 125.77 MHz)

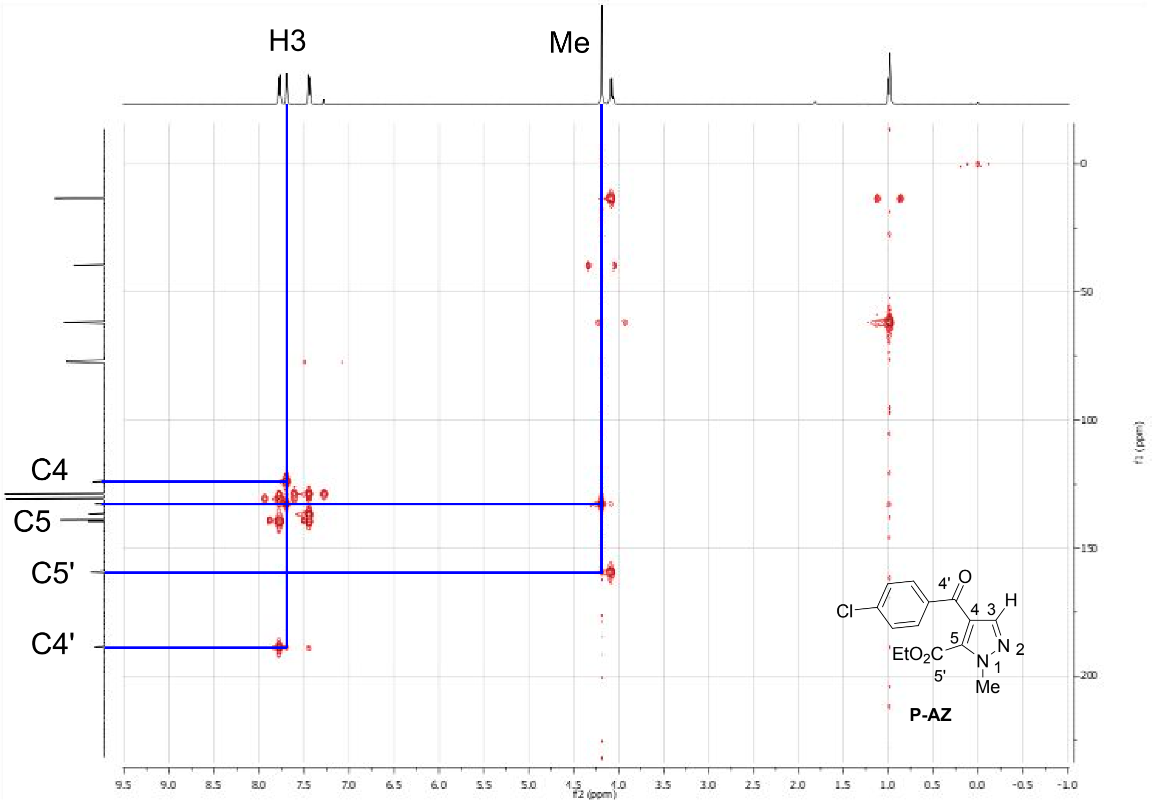

Figure S47 – HMBC NMR spectrum of **P-AZ** ($CDCl_3$, 500.13 x 125.77 MHz)

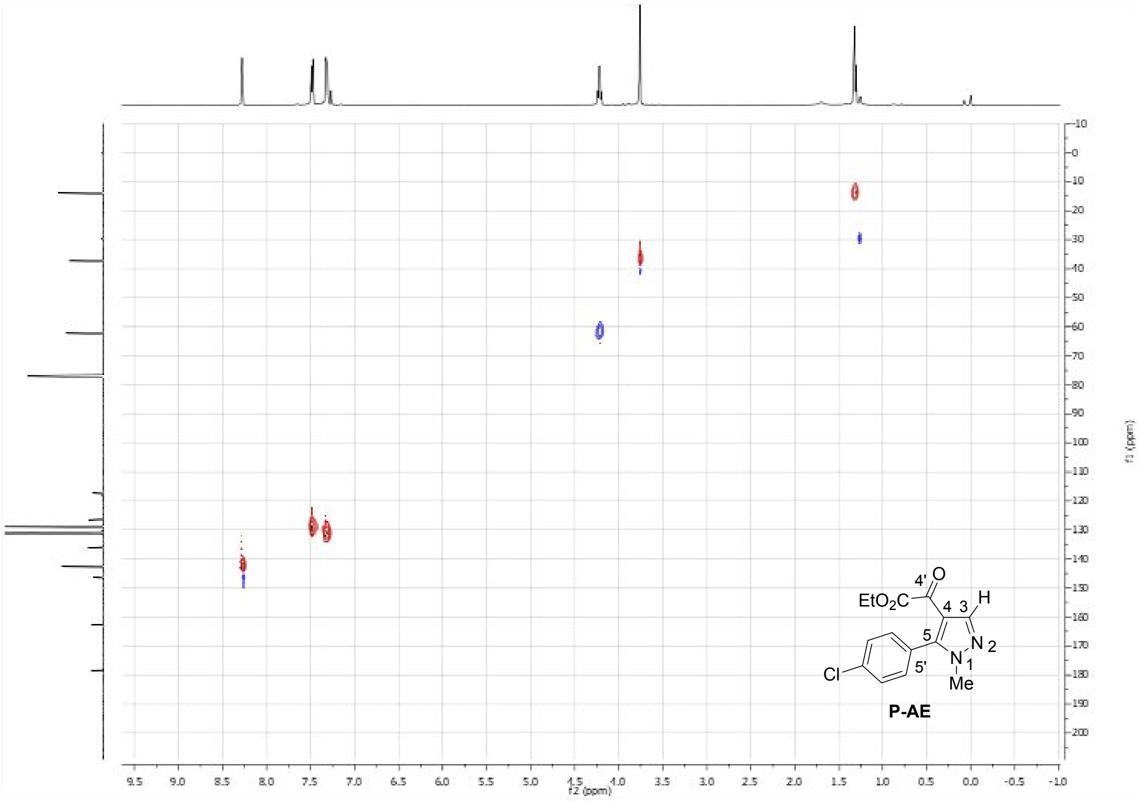

Figure S48 – HSQC NMR spectrum of **P-AE** ($CDCl_3$, 500.13 x 125.77 MHz)

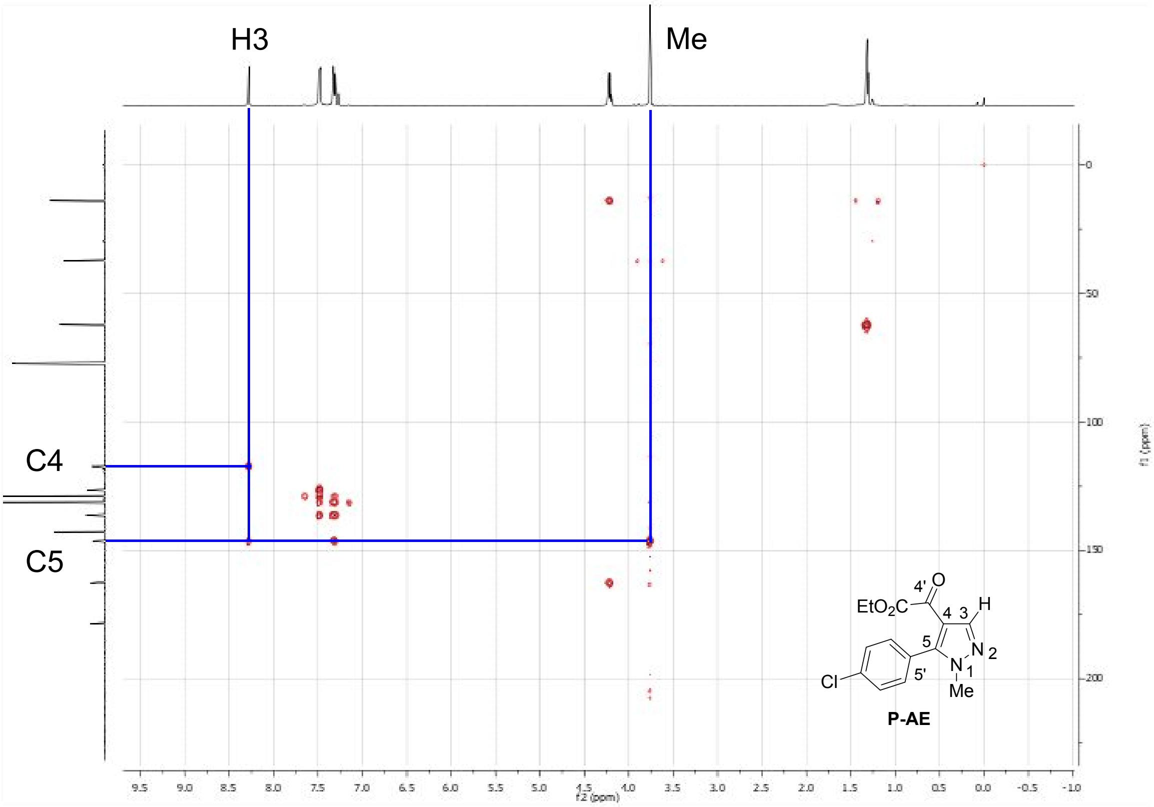

Figure S49 – HMBC NMR spectrum of **P-AE** ($CDCl_3$, 500.13 x 125.77 MHz)

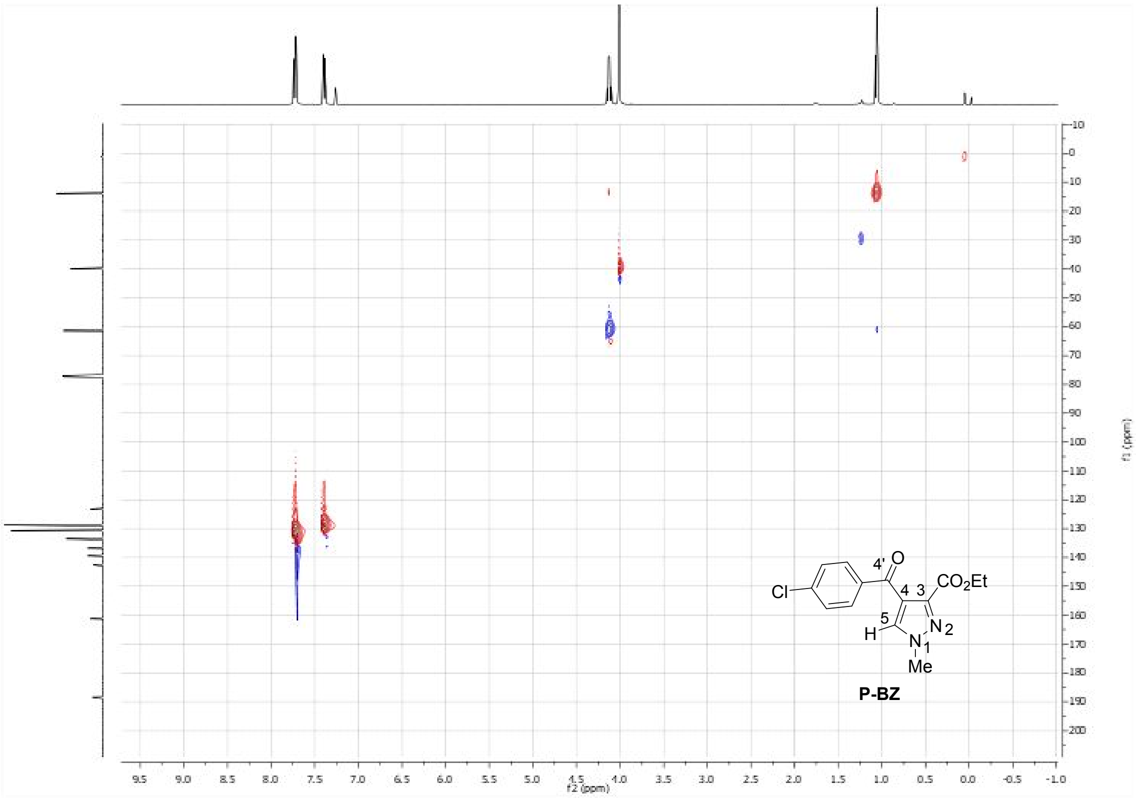

Figure S50 – HSQC NMR spectrum of **P-BZ** ($CDCl_3$, 500.13 x 125.77 MHz)

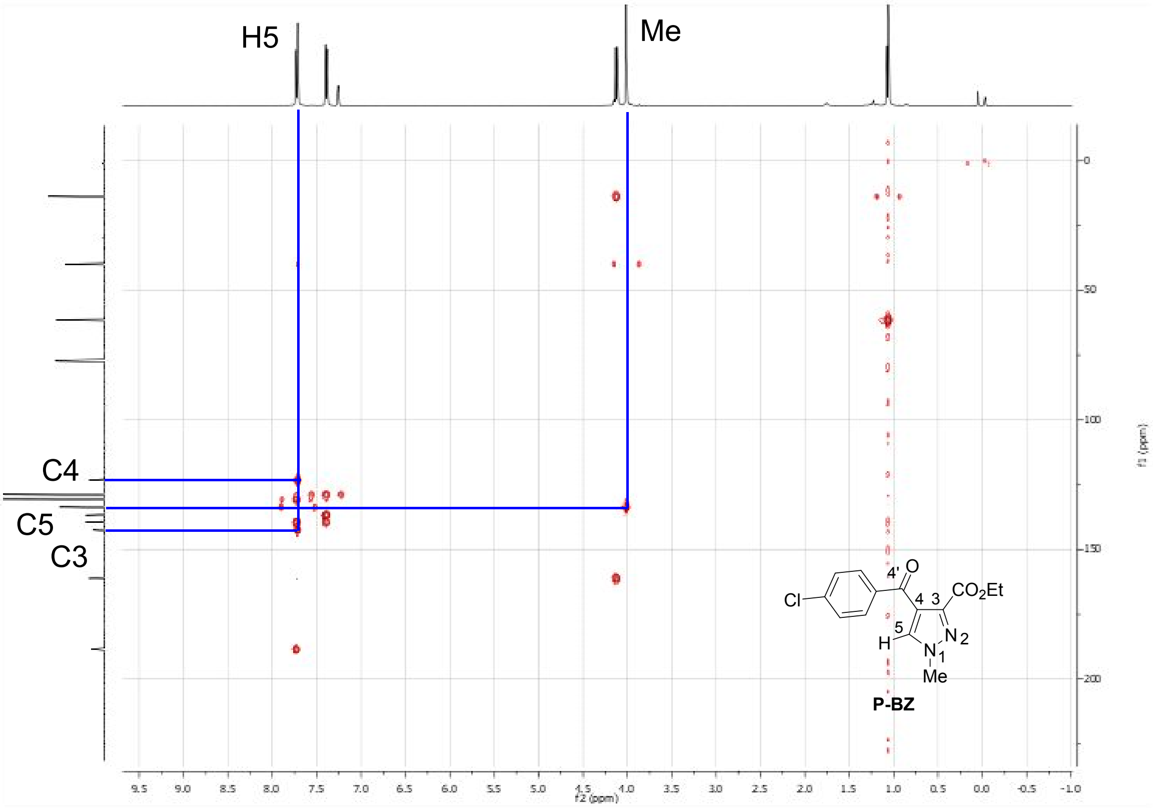

Figure S51 – HMBC NMR spectrum of **P-BZ** ($CDCl_3$, 500.13 x 125.77 MHz)

S13. XYZ Structures

S13.1 CPCM(water)

BED-E

C	-7.61093127183130	12.65682201103260	3.86336847712435
C	-8.17003251228892	11.84045231647425	2.88670461494974
C	-7.32962704046529	11.09689757372188	2.07827216330745
C	-5.94528023340663	11.18136219461532	2.22614726935874
C	-5.40906517627799	12.00453800086243	3.21097193523926
C	-6.24086371121304	12.74344056191507	4.04256429075928
H	-9.24531742803017	11.79451903573547	2.76575073473452
H	-7.74317230146505	10.45356150144836	1.31079229087782
H	-4.33504159962441	12.06374370334474	3.34459748635295
H	-5.82966769254435	13.37937487833107	4.81657520867659
Cl	-8.65966078584512	13.58955048881991	4.88320253558147
C	-5.06477523191984	10.34376046545874	1.35120471625601
C	-3.71800570744246	10.83632916997504	1.02299205488291

C	-3.48722568325995	12.20378340805371	0.63385336271843
C	-4.68852074849042	13.14327258071585	0.41562889505129
O	-5.47827407427485	9.26696236740921	0.93644566341493
O	-2.38787603595635	12.69949029516398	0.39979383994027
O	-4.74588468662136	14.25627068539672	0.87715038013084
O	-5.59181284162510	12.59807920719704	-0.38668289275271
C	-6.78674608012985	13.38125259686413	-0.64609364345974
H	-6.48709617765742	14.31230549977941	-1.12986201647371
H	-7.24747171146718	13.61485551099039	0.31583102291872
C	-7.69152280041802	12.54670740878732	-1.52088906124402
H	-7.96464422952281	11.61628533260446	-1.01864819446827
H	-8.60440436339788	13.10837106444671	-1.73116899316560
H	-7.20442922045358	12.30878438676712	-2.46891446533867
C	-2.61554281003646	9.99159097958031	0.84346655597055
H	-1.79783753693222	10.38808636129560	0.24826803535770
N	-2.40023304539320	8.79434931450344	1.32988823927207
C	-3.23542404461379	8.16415607009484	2.34256613125273
H	-2.59938671989109	7.51263715936644	2.94117546183245
H	-3.67114950733817	8.92871094831464	2.98502211418776
H	-4.03595948817603	7.58129953778050	1.88566131348263
C	-1.23918689579653	8.01885011315894	0.89741722236231
H	-0.62031334082853	7.77710673446793	1.76338780989581
H	-1.57747020633569	7.09139475683505	0.43050534878760
H	-0.66159782902868	8.59442256869090	0.17681216222548

BED Z

C	-2.46455593078482	-2.10004505233092	6.58618446799674
C	-2.83355959701596	-1.93742511358710	5.25650789520706
C	-2.15839545132255	-1.00808814340204	4.48481916786462
C	-1.10813199224569	-0.26731110987885	5.02461248285602
C	-0.75670202546606	-0.44794499880605	6.35847425428591
C	-1.44162837649112	-1.35995099219517	7.15154705294416
H	-3.63556788934105	-2.53276236582647	4.83770801498899
H	-2.43644969254089	-0.86098305705629	3.44771860834481
H	0.04080391409656	0.13959135696408	6.79897557004583
H	-1.17673263650309	-1.49805377702018	8.19241859347212

Cl	-3.29645803883241	-3.27444931228762	7.55553012596766
C	-0.43907489048237	0.77583107018409	4.17985028735869
C	0.99887296182274	0.99062646565759	4.30261209732228
C	1.87948905548804	-0.00854938621547	4.86567205785390
C	1.54752981231564	-1.48867626485271	4.56471501128899
O	-1.14053674125240	1.47646698858824	3.45048178197123
O	2.90656373084275	0.21851996116322	5.48922464540965
O	1.10647057726511	-1.84033339595876	3.49576457698152
O	1.89734296257906	-2.28828220872345	5.55619537087197
C	1.74238358643971	-3.71688049224075	5.33863169905551
H	2.45913392712863	-4.16784631007151	6.02364939258119
H	2.03163245661666	-3.94176028308169	4.31205605642507
C	0.32744270632611	-4.16387493337027	5.63759312769788
H	0.25693275555685	-5.24383112556983	5.48599942291028
H	0.06103121603418	-3.94100675721226	6.67230127549485
H	-0.38759684699704	-3.67258016213567	4.97605596341112
C	1.41541266494846	2.29217200050778	3.98759357876474
H	0.64055626592577	3.05325489826640	3.96373192542372
N	2.60835438070801	2.73056740579419	3.66938265611245
C	3.73632869080717	1.86702824798706	3.35151503960799
H	4.34667723830315	2.37931128377372	2.60814547264878
H	4.33405925612976	1.65459023691709	4.23821032300063
H	3.36968278870549	0.93015345806628	2.93370813280937
C	2.85483364485412	4.16552043692875	3.53706751809661
H	3.20765850142019	4.38187111777109	2.52719844299289
H	1.93660903915884	4.71459872349076	3.73615663858350
H	3.61968797580246	4.46873158976277	4.25499127135102

Methylhydrazine

N	-2.01612394961796	0.00302508767125	-0.02546846916148
H	-1.60727215887857	-0.92947827699468	-0.03147750650722
H	-1.57749993459775	0.46397449032095	-0.81925324369587
N	-1.45337002273690	0.65424025191182	1.15614744549946
H	-1.85904201492275	1.58499234804786	1.14385728326366
C	-1.97936109518483	-0.02165160705427	2.33856371682368
H	-1.54671020310869	-1.02401862303870	2.40767303504547

H	-1.68107363030773	0.54133205000147	3.22533521673496
H	-3.07398503064478	-0.11748384086569	2.32134606199733

$HNMe_2$

N	-0.31227068954852	0.19634133342621	-0.08017527140979
C	-0.23032157152653	1.63871810472363	0.12555767462593
C	0.84033425004194	-0.30883226624725	-0.81877151543790
H	-1.15227226117724	-0.00930597505466	-0.60852302551512
H	0.61883992748253	1.86563058117084	0.77724195708581
H	-1.13916051079685	1.99650730060428	0.61459218894810
H	-0.09349072559622	2.20082182693030	-0.81175391732956
H	1.74373085795973	-0.18208910901154	-0.21454792921162
H	1.00121589412655	0.21201990579745	-1.77590147822933
H	0.71369482903459	-1.37471170233927	-1.02131868352651

GS1A

C	-2.67012891364161	-2.59399472374696	-0.19317316220685
C	-2.67637216262538	-2.10475806408640	1.10782945929434
C	-1.97314074840235	-0.94631447225395	1.38900411730287
C	-1.24327663921961	-0.30082190741134	0.39215503726184
C	-1.26140109703424	-0.80245886496259	-0.90355272272710
C	-1.98353681185999	-1.95060561034090	-1.20706466284420
H	-3.22431555131049	-2.62682292883170	1.88270262780871
H	-1.97141050339920	-0.54669145163632	2.39663444286595
H	-0.71135162150496	-0.29330954713866	-1.68620608358470
H	-2.00341320329421	-2.34315186241994	-2.21617392889172
Cl	-3.54414505620388	-4.05148811248426	-0.55317322033138
C	-0.52822203263082	0.97924231944783	0.70660626471393
C	0.81430013388005	1.18861928199750	0.28925801492025
C	1.73450810975709	0.15707091322113	-0.01729763659067
C	1.43073698662325	-1.30683769014186	0.32212759828570
O	-1.20499189619986	1.85672961592411	1.29813446960384
O	2.89058063442666	0.32868495353981	-0.43997169644210
O	1.30217198330469	-1.70627643053479	1.45687312819830
O	1.46109955905619	-2.08131048778208	-0.75347649037594
C	1.20758590625167	-3.49188111209452	-0.53867124563113

H	0.27545091610199	-3.58869572990892	0.02124390347720
H	2.02230084304320	-3.90019706164418	0.06242316570942
C	1.11869022722541	-4.14678557426995	-1.89703973466224
H	0.30170725585779	-3.71620955587920	-2.48005114685453
H	2.05218727815072	-4.02422156642367	-2.45063641632200
H	0.92846333920656	-5.21493471157304	-1.77011872512259
C	1.38228950572954	2.58724513675011	0.21751681554244
H	2.46668616244742	2.51082736071913	0.23468838780919
N	1.03011014633633	3.43100081115729	-0.89900446429917
C	-0.36530895592913	3.37895927678092	-1.31795129791520
H	-1.02658653375762	3.63113715417691	-0.48890648462622
H	-0.51320261184811	4.11376403895061	-2.11117547037880
H	-0.65097041542788	2.38916152807998	-1.70212073156607
C	1.92897804082299	3.23519663235879	-2.03154011423130
H	1.80949521606996	2.24439963194874	-2.49385419109800
H	1.71659405987147	3.99524391967591	-2.78591347897188
H	2.96525811735895	3.34523118231096	-1.70703131933450
N	0.98744532920116	3.31660224640607	1.50157452318344
H	-0.02579783173786	3.11486917785638	1.63296287524674
H	1.46243731050683	2.86321705816732	2.28385690308952
N	1.23947233300106	4.71996090906346	1.57758968601200
H	0.81024427223159	5.09986924175942	0.73771445932877
C	2.67794793112032	4.99875794939912	1.53566668512674
H	3.16281058808132	4.65603844379016	0.61635836681926
H	2.79554006783346	6.07934354051151	1.61530469703483
H	3.15998339252954	4.53219655157202	2.39736330637300

GS1B

C	-2.67767430079289	-2.44513851065862	-0.01392188920268
C	-2.62238988986131	-1.90090959240105	1.26390095314638
C	-1.88301459026571	-0.74889143356492	1.46750880357444
C	-1.17873499521081	-0.16399741882702	0.41627381851054
C	-1.25744500402889	-0.72036428673860	-0.85464264397863
C	-2.01613273271715	-1.86282854157119	-1.08013777756006
H	-3.15138453641540	-2.37597671226664	2.08102439568020
H	-1.83288357049983	-0.30663996901460	2.45585203561708

H	-0.72672234829603	-0.25849912347921	-1.67889341698961
H	-2.08367443357148	-2.29781221108090	-2.06957132211156
Cl	-3.59842030271782	-3.89439909949859	-0.27670074125899
C	-0.42258780846836	1.10881417338667	0.64882709473877
C	0.90082773884031	1.27732106151792	0.15821092227842
C	1.79567450980178	0.21849351282618	-0.12657207986628
C	1.47671391144349	-1.22130336981900	0.29196206258174
O	-1.04302605288196	2.02062232709821	1.25054062425046
O	2.94226794594288	0.35129789727732	-0.58649593679548
O	1.38599660040959	-1.56305934434069	1.44909126250983
O	1.44608096308066	-2.04605658712639	-0.74536613424919
C	1.17079611384394	-3.43861232766454	-0.45272008064667
H	0.25687244454940	-3.48657395537028	0.14250392617516
H	1.99719769914953	-3.83583924571742	0.13968481595124
C	1.02005492161134	-4.15646367296515	-1.77328157517202
H	1.93592569122191	-4.08274997275329	-2.36372147844779
H	0.81143384488026	-5.21243254173436	-1.58712593582735
H	0.19248840237720	-3.73592969335940	-2.34863192863381
C	1.47674406481730	2.66789242830198	0.00158227003351
H	2.56406186780544	2.59462476074390	-0.02274842280169
N	1.08094166839460	3.43604797884727	-1.15657721478034
C	-0.34678242364620	3.44617509203586	-1.44799374988147
H	-0.51920976882393	4.11590380783234	-2.29243445134405
H	-0.73062884964742	2.44999875590063	-1.71194769558063
H	-0.91432499081154	3.81865515124771	-0.59469567410914
C	1.84918041707860	3.04395017298588	-2.33377780123362
H	2.91743610684083	3.10977726839230	-2.12105981303166
H	1.62011572510855	2.01873115244660	-2.66090409777357
H	1.61101456222202	3.72751287134864	-3.15102108068657
N	1.81670848661733	2.89212153280197	2.42241822829718
H	2.81851031168674	2.94034750303353	2.24820113328226
H	1.57047928196402	1.90421398685974	2.42952638941654
N	1.17147226529113	3.45920321622550	1.27355409259812
H	0.15637607998449	3.30126364730114	1.43624420495161
C	1.49204661401186	4.90176121349281	1.19614377767685
H	2.54854454955316	5.00715581893214	0.94975579102954

H	0.88157379389250	5.35809842511590	0.42451132758982
H	1.27952127623589	5.33333779399966	2.17161481207318

GS2A

C	-3.72171699686930	1.52385622499650	-0.76703146464279
C	-2.49825874993104	1.93132274109118	-1.26688851977888
C	-1.41756268557874	1.06135073326091	-1.18739307055310
C	-1.56744082817658	-0.19688755500336	-0.61693943731916
C	-2.81897923019027	-0.60059347666983	-0.15134544540410
C	-3.89891809958898	0.26160549040187	-0.21193864933124
H	-2.38587057599742	2.91434062525619	-1.70671491769938
H	-0.45394226147697	1.36822004448884	-1.57706122437609
H	-2.94012074789077	-1.58635227377834	0.28260849367464
H	-4.86866692097462	-0.03453749721502	0.16855448888307
Cl	-5.06985548944119	2.61473761533413	-0.83275821555684
C	-0.42247891152265	-1.13689156053391	-0.54451118403925
C	0.78874413284210	-0.85225510438840	0.01237072236350
C	1.04389962999080	0.36641329472533	0.76136672732825
C	-0.10166173665199	1.08397496257470	1.49470711105272
O	-0.67942408464858	-2.30114901141956	-1.12925415254831
O	2.16324163422008	0.80150082329154	0.99048959962755
O	-0.80127654508635	0.51578251259636	2.29924702166545
O	-0.13290265603279	2.37725015082048	1.22606608411440
C	-1.17688154653276	3.14454184255891	1.88111402362976
H	-0.97822462063106	3.14357473915937	2.95434158982588
H	-2.12951807032469	2.64385989357798	1.69826061263324
C	-1.14934749817782	4.53623952787627	1.29561762509936
H	-1.34346566954587	4.50687321908273	0.22128521447852
H	-1.92494849320403	5.14044466494706	1.77154673470264
H	-0.18249420245601	5.01347566216839	1.46913741827333
C	1.97553859749762	-1.79872206621947	-0.05799540955906
H	2.86684351130385	-1.19506865997155	-0.24483921490804
N	2.18080435782693	-2.45903740994045	1.21271293169287
C	1.10316136873725	-3.34478896288363	1.63358596916940
H	1.27621513788331	-3.64272145691013	2.67007043803243
H	0.14835887079522	-2.81893936650217	1.58547099419274

H	1.03237431875360	-4.25958234435257	1.02595999290826
C	3.49012928529143	-3.08411947102483	1.33924004612503
H	3.65802730880178	-3.35278118755867	2.38474360682245
H	3.60082272489913	-4.00152032820117	0.73953215851703
H	4.26425591562289	-2.37689770002098	1.03372602900821
N	1.81086071900051	-2.76028811866027	-1.17823823458521
H	2.34092362809731	-3.60680982235752	-0.98293649973627
H	0.22833870327997	-2.77926086843917	-1.19333855339684
N	2.21980881636466	-2.17934248749466	-2.41144300131636
C	3.64814104141519	-2.36030099133949	-2.66570201851226
H	1.68581693921008	-2.63549970316750	-3.14139261257393
H	3.95165081847372	-3.41547182433037	-2.62096772714384
H	3.87523489883302	-1.96798137282405	-3.65845648205400
H	4.23026426178981	-1.79846414700140	-1.93220959878573

GS2A’

C	-3.39736656839695	1.47425148287476	-0.45755604484768
C	-2.22461614271556	1.53695215006815	-1.18848029920665
C	-1.24124478172038	0.58244640936423	-0.96051352604463
C	-1.43703798924097	-0.41841585448746	-0.01591195323699
C	-2.64269563795182	-0.48531683024228	0.68230381716644
C	-3.62313275336248	0.46848757522791	0.47548734021742
H	-2.07644408146278	2.32080828596318	-1.92066826511933
H	-0.31862602473515	0.61692540211175	-1.52822192200243
H	-2.80129048003981	-1.27397290556966	1.40858355309547
H	-4.55267793269878	0.43991235147670	1.03048243379615
Cl	-4.61875737116555	2.68038886138289	-0.71147991972734
C	-0.39062067052619	-1.44198643128941	0.21695442733061
C	0.92889239213913	-1.15919262087423	0.46674170133978
C	1.42369153517667	0.16387535954686	0.75411374247171
C	0.49951375708524	1.23231634817618	1.36103533147668
O	-0.82806488024160	-2.67839265567695	0.11524191899022
O	2.61070083980504	0.46902728943481	0.68968640342985
O	-0.11808599767041	1.04639313376858	2.38273971257324
O	0.56195855004025	2.37597638923518	0.70073177782857

C	-0.27361737849806	3.45224154603450	1.20113419489010
H	0.09180709905850	3.73647076278302	2.18958071214717
H	-1.29233135073885	3.07204524469722	1.29794883974488
C	-0.18670885477048	4.58930820345061	0.21125859578362
H	-0.55195307786930	4.27684582468304	-0.76950347568260
H	-0.80467031088333	5.41838210357200	0.56324612957191
H	0.84219484207942	4.94162530491126	0.11152388354369
C	1.99839595973511	-2.24407824130710	0.38352545399275
H	2.82296738164463	-1.97741745367974	1.05472022544843
N	1.41181247546730	-3.53411809302001	0.82114270769374
C	2.14602571199537	-4.71415065098374	0.36210620590542
H	1.66024635712342	-5.59613488556695	0.78251106248221
H	2.12034361867335	-4.77470963592276	-0.72304158415420
H	3.19062995514588	-4.69038987762791	0.69539593789816
C	1.28802727456314	-3.54165506027243	2.28428569782494
H	0.74636352139948	-4.43627559712405	2.59331658780789
H	2.27556657940211	-3.53469844568406	2.76204029939952
H	0.73232571128368	-2.66183617960694	2.61228384779421
N	2.60973254114736	-2.34929567693711	-0.92883512448226
H	3.18619307495647	-1.52123463021371	-1.06369151639004
H	0.02334216729475	-3.27992585589161	0.33786012517977
N	1.64705228231641	-2.46631004405824	-1.96990694076807
H	1.21706242327499	-1.55962547130931	-2.13864574774000
C	2.29970172077589	-2.91914251473941	-3.19130009210582
H	2.67591388446927	-3.93435022616776	-3.04733358357303
H	3.13953333378712	-2.27633085711413	-3.49150316666240
H	1.56254729484842	-2.92752333339581	-3.99718950508105

GS2B

C	-2.63919944021530	-2.41541380742590	0.02081258540966
C	-2.62237887413082	-1.80529859573797	-1.22860998451525
C	-1.88793457235466	-0.64585116301882	-1.39736893106064
C	-1.15453638818031	-0.11591271897670	-0.33491262463613
C	-1.20235943914983	-0.73070478593711	0.91140574248615
C	-1.94899316636767	-1.88703696656066	1.09702890240206
H	-3.17508653935627	-2.23839444117557	-2.05306013061119

H	-1.86041407569540	-0.16032193671784	-2.36567841017115
H	-0.66127718214992	-0.30164081034740	1.74648888635686
H	-1.98889417793222	-2.37107192021224	2.06472882891857
Cl	-3.55015038136628	-3.87595769893584	0.23406120143666
C	-0.36801890408210	1.12161727198056	-0.52676340256311
C	0.92134230895789	1.30621094013643	-0.10283935149154
C	1.79270083008678	0.20607710874714	0.24684870750573
C	1.49577198008601	-1.21412473785282	-0.26247774468503
O	-1.05433023895981	2.08282025048368	-1.12707939575383
O	2.87988008486808	0.33854351305391	0.79512539959111
O	1.39476076237554	-1.46822019317716	-1.43947085932297
O	1.48894158099179	-2.10108176616491	0.71739672544391
C	1.22586448279670	-3.47718947017839	0.33703230784929
H	2.05208670493637	-3.82302883626860	-0.28662067550530
H	0.30730110894352	-3.49479733153336	-0.25262704572867
C	1.09493086858044	-4.27988349637859	1.60935971728772
H	2.01503608120497	-4.23389692767227	2.19585029168473
H	0.26713428844246	-3.90802410834072	2.21703477583957
H	0.89703057597442	-5.32370400970695	1.35552624649748
C	1.53332193161554	2.70483544874924	-0.03309583391319
H	2.62465043318961	2.60598129021975	-0.04328585117531
N	1.20681601378646	3.45185099400365	1.16961895252356
C	-0.21024091310682	3.55177756503317	1.48574464223605
H	-0.32389534012682	4.20182041800108	2.35536006368845
H	-0.76304379375923	3.99708428100055	0.65747925445834
H	-0.66461115486190	2.57835783184223	1.72620767182247
C	1.96800039390940	3.00471573969375	2.32791142316525
H	1.80587143172857	3.70873988319680	3.14737113617964
H	1.67100139865068	2.00244119899696	2.67188091710260
H	3.03213437988586	2.98326291137537	2.08877089058141
N	1.44031863285978	4.85095543813105	-1.19025168123968
H	2.42767337290388	4.93653255288008	-0.94072546095333
H	0.92766454292839	5.25399501010699	-0.41422771113062
N	1.10613987072449	3.46765552955178	-1.24054338822586
H	-0.39965691505126	2.85331945062692	-1.28020049069824
C	1.72163139462784	2.90477135562054	-2.44520043424427

H	1.33766461560611	3.43799010545187	-3.31385617569217
H	2.81419266496199	3.00825116735427	-2.40934684070348
H	1.47257697122295	1.84729622608191	-2.53081520644634

GS2B’

C	-3.57926438860923	-0.78283223973921	-0.83850422481617
C	-2.93881782524624	-1.75159905124963	-0.07450456842171
C	-1.55679272465651	-1.75768490176922	-0.02276916883918
C	-0.82070918456447	-0.79059585312685	-0.70823694645660
C	-1.48009956770749	0.15899704885551	-1.47842478959422
C	-2.86840211814763	0.16730006585111	-1.54879465550778
H	-3.51838233277133	-2.48571118385712	0.47145014937937
H	-1.04316907479085	-2.50472448948094	0.57136622351231
H	-0.91255954514503	0.89618654797083	-2.03490140660611
H	-3.38724880795400	0.90589710540239	-2.14703995809542
Cl	-5.31396804174712	-0.77020463986806	-0.90251389506799
C	0.66159175202912	-0.82718811145048	-0.64444807731260
C	1.46237856234957	0.22244360603948	-0.29808824346915
C	0.93457723669607	1.51341703252428	0.11105540132313
C	-0.41103126900611	1.63841398227848	0.86074325104909
O	1.15781623197029	-1.99024376397525	-1.03838498331539
O	1.54423147739268	2.56366631373951	-0.02197575615645
O	-1.20191321618996	2.51904304195789	0.63138550159301
O	-0.51562944562888	0.73270275876289	1.82086714539728
C	-1.73037611502479	0.74574078409507	2.61726306238173
H	-1.82303485083333	-0.27940984019888	2.97338759745788
H	-2.56846775414264	0.97609287165569	1.95915801228212
C	-1.61593276171026	1.72884864814407	3.76272885894830
H	-0.75455751779028	1.48847420644010	4.38979015590480
H	-1.51421224984515	2.75084059896995	3.39389538950791
H	-2.51793130616261	1.66959070336047	4.37666255032126
C	2.98774705543820	0.10622897413552	-0.31922867672639
H	3.40680156518249	1.09368117397017	-0.54231881636385
N	3.40553706926122	-0.85295264150799	-1.35989848850475
C	3.17876299647006	-0.27128238080211	-2.68707639736421
H	2.13959241811306	0.04359476387077	-2.78859325712417

H	3.82655228262943	0.59990316392543	-2.85275835104262
H	3.39292539318951	-1.02273279964395	-3.44788630956639
C	4.79824333184784	-1.28374678363148	-1.23488179481870
H	4.94160852945761	-1.84338681195617	-0.31443245370891
H	5.02963308769072	-1.92747578335349	-2.08520957042921
H	5.48553586177140	-0.42717172982978	-1.24333784188476
N	2.98233862864978	-1.49841232988380	1.42674029933721
H	2.09819303083202	-1.32227272108366	1.90653694702523
H	3.62106852481537	-1.88419803529901	2.11328022854279
N	3.54147452371002	-0.27303205774787	0.97364487096552
H	2.17577929226409	-1.83460393229457	-1.13468449778172
C	3.44252996241327	0.78095599023941	1.97471697478573
H	4.02000985593632	0.48067412047958	2.85146723041875
H	3.85717882252407	1.70921512895394	1.57828291018904
H	2.40529987503968	0.96186998012695	2.29535019865197

GS3A

C	-3.66407857599264	-0.86419534996337	0.69580749297980
C	-2.80677284563954	-1.58779627913997	-0.11332319450270
C	-1.44109316826105	-1.33754612055052	-0.03728647570623
C	-0.94535370801269	-0.38691152154392	0.84596205192003
C	-1.82757669936001	0.30929085212350	1.67139900035946
C	-3.19093263598174	0.08759916669169	1.59149542369940
H	-3.19677537702851	-2.33248413806888	-0.79614439677133
H	-0.75915140847640	-1.89542179678821	-0.66863513369562
H	-1.44025349057068	1.04249261332780	2.36934421369838
H	-3.88309075706546	0.64245863257610	2.21300186409630
Cl	-5.37472597617961	-1.14986411866013	0.59277044198911
C	0.53053678916491	-0.17170036811416	1.00205054790051
C	1.38245641720595	0.01190812453251	-0.12124762071267
C	0.98899809595544	0.42129943274844	-1.41577348051056
C	-0.43909665184164	0.89764031785951	-1.71638023660355
O	0.97353723966722	-0.21716948532971	2.17465352537732
O	1.74686222615161	0.51769019317454	-2.39635512065018
O	-1.12210104789916	0.43157457797123	-2.59743310307212
O	-0.77159128118759	1.94903262309764	-0.97694134533973

C	-2.10139922918008	2.48812702499494	-1.18461039113733
H	-2.81944416748317	1.68206098064274	-1.02152166932104
H	-2.17987270005853	2.82486884132833	-2.21987910880453
C	-2.28995178727684	3.62010517722525	-0.20260003473068
H	-1.55142188232971	4.40730370276897	-0.36967459622078
H	-3.28686496738136	4.04706428545445	-0.33351572973148
H	-2.19902959670850	3.26092274437734	0.82457549764952
C	2.86093366936761	-0.18689694269422	0.07673620983929
H	3.40838731357712	0.18168468318255	-0.79144792108011
N	3.33471509168957	0.68385513627743	1.24322923644945
C	4.74364705843649	0.43600526062932	1.62997156637905
H	4.97553866813892	1.06856241500047	2.48541226669465
H	4.86219047558761	-0.61067923079589	1.89093568078380
H	5.38491686156591	0.68984614661274	0.78675485988254
C	3.11201274500583	2.12314878669274	0.96075285231314
H	2.05638081438323	2.28679394289145	0.75951715578567
H	3.42449566569777	2.69816255847578	1.83021370453518
H	3.70860378578274	2.40203982579686	0.09235057422507
N	3.20016131216744	-1.56097744672537	0.35712636043846
H	2.40468172650537	-2.15410833371645	0.14155253976161
H	2.69697815612913	0.40107232535341	2.00631672912266
N	4.36859271900220	-1.99691024181738	-0.31849813180708
H	4.79991169790751	-2.70575828894346	0.26206074730946
C	4.07263814219306	-2.54920142800631	-1.63858074856443
H	3.70626729535145	-1.75408163254534	-2.29225753085042
H	3.31533944017877	-3.34556292588149	-1.60042743776468
H	4.99189812710204	-2.95544520252297	-2.06558668561259

GS3B

C	-3.33265635271023	1.26213044092066	-0.85140822138460
C	-2.88006658979782	1.99047172294477	0.24286956835167
C	-1.53913365500545	1.92694067860798	0.57839097047626
C	-0.66381834711165	1.12102121925258	-0.14803662253771
C	-1.13736227730190	0.41169354630873	-1.24498348741143
C	-2.47700853356461	0.48308170916889	-1.60908217100975
H	-3.57145238646727	2.59428670199813	0.81789449290729

H	-1.16637845127444	2.49078844679376	1.42572778205177
H	-0.45644429984063	-0.20185096670014	-1.82336018595165
H	-2.84996954442493	-0.06596368865131	-2.46488281114781
Cl	-5.01581262440956	1.33397136763641	-1.27597712569043
C	0.79367440594321	1.10025019088109	0.20719059204299
C	1.50283109680424	-0.13421348003595	0.27367138632846
C	0.90046366716069	-1.37706899557428	0.56993405825432
C	-0.55940017454555	-1.46566995546068	1.02742089989220
O	1.33403134930062	2.21607554190879	0.38775260507615
O	1.49476235418013	-2.46877501103773	0.63847584323699
O	-0.94811241362414	-0.99845068867256	2.07357968005425
O	-1.28758411815558	-2.21680762706148	0.21074455455275
C	-2.68139596775528	-2.40982975821024	0.55912490308069
H	-3.17013084554259	-2.60461701512466	-0.39504451069222
H	-3.06686685793804	-1.47824371774767	0.97472135061797
C	-2.84660243529919	-3.57014857365320	1.51809954107666
H	-2.34603669697248	-3.36741712761371	2.46641227709427
H	-3.91029616511235	-3.72726803380769	1.71372322976313
H	-2.43582797252379	-4.48605403372668	1.08733464873972
C	3.01171955251952	-0.17148185910080	0.15194024137651
H	3.37938487019256	-1.08892808149944	0.61224665151191
N	3.61499648939467	0.97040871893064	0.93995157691194
C	3.39708162813205	0.75471442688877	2.39417215865006
H	2.34128139554458	0.55981528941589	2.56672044226022
H	3.99598956664120	-0.09853399724527	2.71095546159926
H	3.70674144448095	1.65249022132698	2.92545597970717
C	5.04875986920123	1.21797117479472	0.65496827987287
H	5.16179094764350	1.53508553026713	-0.37589993010219
H	5.39047345063801	1.99791695831093	1.33386627861419
H	5.60381470321884	0.29697824321311	0.82965773361870
N	3.07624624454312	0.98925950368861	-1.90091790141986
H	2.13587072281315	0.81363754237345	-2.25914757988936
H	3.68198721121989	1.11434030503179	-2.70442156400790
N	3.54860697041400	-0.15078627431608	-1.19552184768345
H	3.03468128899331	1.78814921475886	0.67116640341733
C	3.27461064175259	-1.39818861243641	-1.90212202366573

H	2.19757520233923	-1.55126001896097	-2.06299552165673
H	3.77504971951001	-1.36013400524713	-2.87126385025813
H	3.66904528679616	-2.23905589353859	-1.33008253662876

GS4A-E

C	-2.80453551221961	-1.01933890951562	0.18595585540632
C	-1.98867177897616	-0.40777515356464	1.12164310346734
C	-0.83981636769444	0.23924512277589	0.68614307164683
C	-0.52385273812269	0.28398848745254	-0.66829191783058
C	-1.37112127788133	-0.32609113940863	-1.59306776854995
C	-2.51058833439836	-0.98772484768366	-1.17239943239972
H	-2.24212382572822	-0.44181990651163	2.17385242569608
H	-0.19822062125804	0.72667029929265	1.41088920097741
H	-1.12167677335446	-0.28823658499846	-2.64673210845775
H	-3.16484274353926	-1.47914125256612	-1.88191260032313
Cl	-4.22281105497358	-1.86143619271632	0.72181033067190
C	0.68289542669951	1.02250264369294	-1.15345004969177
C	1.82425266908818	1.18871036786210	-0.22998576129321
C	2.37971078312872	0.06878529070423	0.48735715719399
C	1.89303703754877	-1.33929649938581	0.09831241897724
O	0.70716180597638	1.46966736895051	-2.29406896069549
O	3.28935618181564	0.12768874051613	1.30845953232037
O	1.84398088944208	-1.70298489199163	-1.05377613250305
O	1.63624213254031	-2.08511262586930	1.15792633293077
C	1.24938556875838	-3.46649713305793	0.91623457854732
H	1.51258864428439	-3.97972293170121	1.84004002452319
H	1.85670558312448	-3.85608206016523	0.09944425084052
C	-0.23154683930651	-3.56959598317353	0.62080349784948
H	-0.49118492188525	-3.01640986908266	-0.28307941892149
H	-0.49246824741029	-4.62034479937534	0.47252381089683
H	-0.82006457095406	-3.18004761438504	1.45335629060878
C	2.53773012133705	2.37477044833295	-0.12462150474790
H	3.52746396026006	2.34572744282150	0.32058458254676
N	2.15389935022190	3.57888200562524	-0.47993488264110
H	2.81876138760581	4.34206049711036	-0.42514682186387
N	0.85665008737014	3.87963614846481	-0.89920118326998

C	0.55747675566123	5.28972746274807	-0.66507162444663
H	0.77311111388407	3.64145980217736	-1.88439258300263
H	1.29853693222144	5.96098285571331	-1.11839772103137
H	-0.41957933770019	5.49597998241667	-1.10161771979997
H	0.51281223443389	5.47877232849549	0.40889551636847

GS4A-E-H_2O

C	-2.76737651919843	-0.75457069451216	0.07468532659835
C	-1.93499199334523	-0.16910931068634	1.01340026324132
C	-0.72991453289934	0.37486083797749	0.59113092888351
C	-0.37389735241174	0.34374340370820	-0.75395055367647
C	-1.23667357072042	-0.23793658696715	-1.68282489282772
C	-2.43426475879295	-0.79628682749003	-1.27435071725600
H	-2.21997866125943	-0.14563028557984	2.05774407260329
H	-0.07231464534864	0.83888699001649	1.31677458440971
H	-0.95356857060526	-0.26176627548077	-2.72828136362213
H	-3.10333655546270	-1.26471961908938	-1.98551729521304
Cl	-4.25920199330414	-1.46783880724737	0.59570457576927
C	0.90730907728605	0.94853971322454	-1.22186573360589
C	2.00609907896866	1.13478055848467	-0.23774942462232
C	2.54139395662115	0.01869802815129	0.49573101032018
C	2.00263945267455	-1.37612012222010	0.13069497591022
O	1.05538842864234	1.25399533027933	-2.39676412856320
O	3.43993951953056	0.06762796240827	1.33035066679619
O	1.93654655245277	-1.75990650016805	-1.01411239021589
O	1.69866276253872	-2.08338766146937	1.20395446276182
C	1.21355271283860	-3.43791123631182	0.98830099325083
H	1.43876209370545	-3.95106167758284	1.92210508663902
H	1.79028069230278	-3.88651974430435	0.17965653023613
C	-0.27156606203552	-3.43604995049233	0.69373305919706
H	-0.49039816149388	-2.88209082523638	-0.22058590300521
H	-0.61082677881590	-4.46674658423339	0.56495082700818
H	-0.82847620482700	-2.98800339919472	1.51873382054700
C	2.64370369304642	2.34711844573547	-0.05233458710217
H	3.59317070025098	2.35462519326905	0.47470942852843
N	2.22165737720406	3.54103021081983	-0.41306235732356

H	2.81641034290797	4.34438804327607	-0.24434774246228
N	0.93122237256848	3.76552651957318	-0.89879152407198
C	0.33644446742291	4.93077851284568	-0.24842325124203
H	0.97372487412025	3.89892245233522	-1.90744312565746
H	0.96823091863224	5.82602924040754	-0.32278972358314
H	-0.61789095192249	5.13145571477156	-0.73672696660284
H	0.15657653562291	4.70849444352503	0.80501092609723
O	0.66547787016331	3.55494586550341	-4.01920730795418
H	-0.25604680165825	3.71547847359896	-4.25014747406405
H	0.68143063460003	2.65423016835495	-3.66099507612612

GS4A-E-2H_2O

C	-1.69736566161394	3.16393572406949	2.28435452066454
C	-2.67317999542699	2.17666422073478	2.21115292916471
C	-2.30986555595541	0.90975215624763	1.79218796135343
C	-0.99003895102714	0.63404824679914	1.43373235743920
C	-0.03132016015451	1.64008960980883	1.51034253653633
C	-0.37834791482315	2.91195556036838	1.94455860161566
H	-3.69851988292200	2.40371926859845	2.47552740429455
H	-3.05852399803816	0.13000645165217	1.72056939174586
H	0.99786318787903	1.42937225082820	1.24401075227410
H	0.36411674577847	3.69685774780410	2.01601713503689
Cl	-2.14613876812587	4.75305694717620	2.81274458997597
C	-0.61612238882999	-0.74204625449563	0.99619503816206
C	0.53267046873285	-0.90762817805914	0.08096129958053
C	0.63212165223704	-0.15885076074169	-1.14505534873164
C	-0.52187932397247	0.78591435973292	-1.53030285164291
O	-1.27134715859311	-1.70908502961721	1.37102905606630
O	1.55852653320573	-0.22111115764888	-1.94743252800080
O	-0.34723476943289	1.92585977394528	-1.88315820650850
O	-1.69348779614659	0.16939828843663	-1.47210554065355
C	-2.86668014349197	0.96831233001338	-1.78077657342427
H	-2.76565462839248	1.34343243887251	-2.80039558894632
H	-2.88355296115834	1.81542299477216	-1.09207929083236
C	-4.07455840782484	0.07729459905110	-1.61560695240156
H	-4.14203144823684	-0.29826048700289	-0.59242622708513

H	-4.97665505560923	0.65309773825793	-1.83394999926860
H	-4.02902405311904	-0.77030452131370	-2.30263957081455
C	1.50757424313787	-1.87660868246830	0.27321028372450
H	2.14034507693129	-2.14247950342854	-0.56834459765535
N	1.26733197285589	-2.16335639770634	2.62619447709248
C	2.31620393126447	-2.16788668960277	3.64441695436873
H	0.54612203391938	-2.84241500414122	2.87154741974268
N	1.80603403037394	-2.50936520936703	1.38394255367132
H	2.52023724440521	-3.22954124868465	1.35823829219082
H	3.01869067337895	-1.35535576952557	3.45018046882891
H	2.86686095351139	-3.11743530148154	3.68197895309057
H	1.83860156545051	-2.00054295607606	4.61034086960916
O	-0.68756587259695	-4.20339951184467	3.86980421323179
H	-0.98777404443593	-4.81604534380682	3.18959913672378
H	-1.40225582136518	-3.54088672411243	3.94230369322464
O	-2.56622202040450	-2.10890686409156	3.79810404900358
H	-2.23615420184639	-1.85406518132997	2.91444549123645
H	-3.48659932951812	-2.36111393062262	3.66468284631596

GS4A-E-3H_2O

C	0.90236838151646	4.04980688583948	1.18151578315248
C	-0.24732066640077	3.32240230477216	1.44124461677873
C	-0.29696175795638	1.98184338777259	1.07658503005998
C	0.78628943677226	1.37084407880630	0.45716560041546
C	1.93032080671739	2.12629062851331	0.20681637236906
C	1.99910827083424	3.46403156320325	0.56598436773682
H	-1.09702801556692	3.79668842516227	1.91755321752459
H	-1.20293218880682	1.42505876691345	1.27420883137170
H	2.78563874226874	1.67166275932771	-0.28010663419920
H	2.89015043880369	4.04708481892079	0.36686555948374
Cl	0.97175219964582	5.72883373796850	1.62885324568487
C	0.76856725733546	-0.10800695447721	0.05496560780976
C	1.39751683240447	-0.34197621925500	-1.30629549108891
C	0.84913373822231	0.00104686945791	-2.58207357040094
C	-0.53638458863066	0.65597291060353	-2.61064966902897

O	-0.52803033174572	-0.64200754464030	0.07475803424607
O	1.39671964425641	-0.18152387535776	-3.66568339018959
O	-0.71581686004830	1.81829836746230	-2.33508404949712
O	-1.46113125698872	-0.18897940717995	-3.03580717756292
C	-2.81530250834831	0.32800229258065	-3.13333308023425
H	-2.81502542232777	1.15082438269573	-3.85041311769651
H	-3.10128876653363	0.71496440287641	-2.15348181179904
C	-3.70298281157817	-0.81124043227972	-3.57483759245339
H	-3.67796804514158	-1.62645369085854	-2.84848121460420
H	-4.73150854939671	-0.45286232623670	-3.65791698665433
H	-3.38983271655251	-1.19375075967282	-4.54870565701111
C	2.58258782792051	-0.98835246316410	-1.12976111566802
H	3.27866500560631	-1.32829565626742	-1.88480426259858
N	1.66976082672910	-0.93600098832936	0.92860673804634
C	1.98957718163841	-0.43830870047659	2.26305093732742
H	0.88740703437447	-2.63187234999317	1.02259253950813
N	2.84580094557303	-1.19468741411782	0.16580402202515
H	3.39578734135766	-1.98736751789801	0.47154095068597
H	2.60691406882463	0.46412918771336	2.24383923585926
H	2.51506702395882	-1.22714152894587	2.80413460287998
H	1.05253019346158	-0.23075485304055	2.77994231498736
O	0.54749257130273	-3.55599252107553	1.04582176618773
H	-0.02419054642688	-3.62079001263371	0.27224187359101
H	-0.46908397063992	-3.52189838283937	2.52440089745110
O	-0.97576951371690	-3.32813014642606	3.34284217615918
H	-1.47188744086998	-1.65255423958998	2.94447231341673
H	-1.71427896712079	-3.94711975994910	3.34331091492380
O	-1.61248231471385	-0.73516061167610	2.62398973116408
H	-0.89406487188978	-0.63135640407984	0.98682991478803
H	-2.56798365812340	-0.61480101012908	2.58739762505258

GS4A-Z

C	-2.85568464266712	-1.99298589220650	-0.03666249497332
C	-2.65812717850464	-1.66638736962720	1.29941493490680
C	-1.90855284377928	-0.54515169894975	1.60919366159366
C	-1.33869676928616	0.22725611567868	0.59846747529411

C	-1.55075561302512	-0.11915920423503	-0.73216011696078
C	-2.32129842885062	-1.22802539468731	-1.05800055314065
H	-3.08492189567915	-2.28475176862914	2.07946164093748
H	-1.74888912176036	-0.26963223237828	2.64509408600062
H	-1.13608952440338	0.48760942952162	-1.52912696986765
H	-2.49566738980849	-1.49737892795464	-2.09224700569958
Cl	-3.78058463235799	-3.40653724793768	-0.43242200088110
C	-0.58384106656264	1.46926282890953	0.96510907950141
C	0.59628151619324	1.85424934229529	0.19037144736477
C	1.34757651953721	0.91142851824403	-0.59516622555867
C	1.44934525307232	-0.53340620655607	-0.05845338337677
O	-1.02192061227115	2.17964999902091	1.86733539975055
O	1.97339071611654	1.17073299074869	-1.62106034467200
O	1.55071685033224	-0.77210103940632	1.12191565745631
O	1.50966649152260	-1.42173851716581	-1.03384879785024
C	1.72115950944871	-2.80824884193050	-0.65203799887250
H	2.43935580731431	-2.83343781993593	0.16752482753055
H	2.16608714135768	-3.26077533005562	-1.53723648035378
C	0.41532318463475	-3.47689056516166	-0.27951837862729
H	-0.04283793531745	-2.99085620393154	0.58313912353275
H	-0.28700526818692	-3.44792844058223	-1.11446773726219
H	0.61110413182183	-4.52192130641299	-0.02689889713960
C	0.84039605679396	3.23402175787535	0.15817629203375
H	0.03875817898169	3.90472951524374	0.45003535472937
N	1.94452246897383	3.85694659740009	-0.15784098762111
H	1.93002468110374	4.86904458039140	-0.19898185778413
N	3.18394407044483	3.24522760600646	-0.33893098625481
C	4.23931135967600	4.24668410048675	-0.47753634545722
H	3.11962292732300	2.64434218859373	-1.16076824281249
H	5.16466499966884	3.71562448537711	-0.69825846990018
H	4.03918438842633	4.96463584808040	-1.28246097341593
H	4.36004312971677	4.78468994387042	0.46462195784986

GS4A-Z-H_2O

C	-2.82200524847221	-2.03226415854232	0.07205649567162
C	-2.57215756876188	-1.68953653263209	1.39523073831281

C	-1.85940761897061	-0.53404213323064	1.66295877215624
C	-1.37783234240644	0.25850501358862	0.62227588834651
C	-1.64253818588046	-0.10366563720727	-0.69465544265262
C	-2.37659840157102	-1.24864378043111	-0.97750495330964
H	-2.92954268147089	-2.32277126991268	2.19791961040676
H	-1.65824714197362	-0.24686287282475	2.68840698027729
H	-1.29955762140469	0.51744785207785	-1.51435397903648
H	-2.59146330869263	-1.53075139523537	-2.00067518649454
Cl	-3.69890727449776	-3.48932235416503	-0.27141153298677
C	-0.64777389383384	1.52675342372309	0.94955513250510
C	0.49275492631547	1.92634181881213	0.12855993877112
C	1.18706850792747	0.98044503560536	-0.72669746697569
C	1.40700269470385	-0.44147742155001	-0.15780756946442
O	-1.06664945289091	2.22697130289679	1.86955023479996
O	1.64611068393787	1.22395344715100	-1.83081077244149
O	1.58795265243387	-0.63592462110064	1.02069083416869
O	1.45571037561408	-1.35385758081681	-1.11038124770513
C	1.74662169560201	-2.71858495931751	-0.70306958759528
H	2.50467611409578	-2.69002718469676	0.07964514837878
H	2.16664753912653	-3.17746407161916	-1.59708399351510
C	0.48962039519034	-3.42821068678672	-0.24773307862693
H	0.05739051534888	-2.93910844325058	0.62657040481511
H	-0.25528333032868	-3.44632474104459	-1.04535038052561
H	0.73894202880857	-4.45846301864449	0.01790839186479
C	0.77182851465851	3.28995390091252	0.13951499358982
H	0.01055533502600	3.95978593763359	0.52841212571372
N	1.86795142271490	3.90900141760932	-0.22967102118540
H	1.87600435562947	4.92332538926662	-0.21968069983664
N	3.06736504279123	3.25696025033689	-0.52640831730007
C	4.19639245710707	4.04080765781478	-0.02949379275186
H	3.13369168971161	3.14383482714755	-1.53634955098639
H	5.11047395778431	3.56063096011542	-0.37965305463223
H	4.18241844641491	5.07911428789119	-0.38726413042471
H	4.19073572065485	4.03974536037355	1.06193201001199
O	3.50513305224438	2.53884082586302	-3.60287921594651
H	2.83932433839967	1.92591262189700	-3.26094980102695

H	3.02369160891395	3.11137153229211	-4.21030292436981

GS4A-Z-2H_2O

C	-4.12169930649636	-0.39736194805211	-0.40125520978134
C	-3.19438512185291	-0.50392294547689	0.62276430182880
C	-1.84188862366059	-0.48972888023558	0.30818730585780
C	-1.42664283291575	-0.38620974423604	-1.01592826606898
C	-2.37710217567752	-0.30059788091293	-2.03009315059573
C	-3.73020977785498	-0.29298275760188	-1.72850144838589
H	-3.52364451003263	-0.59627277122302	1.65042351027827
H	-1.10919144397390	-0.57588869179508	1.10273910283848
H	-2.05487508149119	-0.23216752262763	-3.06305876892347
H	-4.47393416651586	-0.21119993433890	-2.51166601549076
Cl	-5.81532952295477	-0.39939270389806	-0.01578182213097
C	0.02758430439491	-0.44495922068475	-1.39238035816499
C	0.95920084179279	0.46074776231496	-0.75646495132177
C	2.40545805410154	0.23285924385452	-1.04750094485998
C	3.30092416008987	-0.40136912811131	0.03887304332832
O	0.38525507056783	-1.27952386511706	-2.22436171306406
O	2.90104125442230	0.41400010521029	-2.13831814246015
O	4.49545623551454	-0.50316902426500	-0.08517419937852
O	2.58937577043970	-0.81812255663321	1.07427542167613
C	3.30946611140065	-1.46532410473482	2.15724068543188
H	2.66851093162732	-1.32099444373345	3.02593611439469
H	4.25150634249707	-0.93855571390244	2.31019145396893
C	3.52156540531430	-2.93407294889708	1.85731735614569
H	2.56476256146998	-3.43339447244414	1.68990817956340
H	4.15225786037860	-3.06771888746301	0.97670994845845
H	4.01376527282097	-3.40618800826193	2.71103011205061
C	0.53667584534669	1.52457598723060	-0.00804304798233
H	-0.52093912753696	1.72564663474379	0.11475765516656
N	2.71357663650985	2.28962990235791	0.45128921457090
H	3.03097519399804	3.02287714403904	-0.18449882559585
C	3.39419579880618	2.36024569992510	1.74115462373935
N	1.32944853815034	2.40958860555740	0.58325648145026
H	0.94056823720396	3.24002715130045	1.01056426703421

H	3.06651576542901	1.53134192803302	2.37009186740578
H	4.46619776337709	2.26895543036714	1.56030928080066
H	3.19953735419303	3.30317282445300	2.26602028378039
O	2.88448262942504	4.30034634113596	-1.76916490944477
H	3.74847506627031	4.48861188474997	-2.15093790375396
H	2.43707902164243	3.72668648680601	-2.42231105546579
O	1.53676918008820	2.50933314602333	-3.48151137194977
H	1.76993259900067	2.56305979276838	-4.41511437767342
H	1.93528188469017	1.67981208377542	-3.16187372727707

GS4A-Z-3H_2O

C	-2.82810419115852	-1.98081414996575	-0.14263716746899
C	-2.70233850532578	-1.61321685780457	1.19145714698080
C	-2.00448267219200	-0.45980821669731	1.50388240038443
C	-1.41502721242596	0.30543236794679	0.49893983240776
C	-1.55696594257735	-0.08087309946431	-0.83011332267069
C	-2.27462738914325	-1.22371133383509	-1.15967973951006
H	-3.14279009122358	-2.22575953823506	1.96849914413860
H	-1.89931839116605	-0.15394646461763	2.53823075879224
H	-1.12900151261903	0.51921461592491	-1.62520715252330
H	-2.39359388020422	-1.52448604505898	-2.19310697799816
Cl	-3.68584582859785	-3.43545435926331	-0.54027436574954
C	-0.70287627204564	1.57198728335999	0.86857886486588
C	0.51561741717338	1.94178819156422	0.15130734456502
C	1.28164996831933	0.97316348264863	-0.60114032336622
C	1.41001355777650	-0.44462672610773	0.00106488852166
O	-1.19812203150454	2.29800898269103	1.72824525163029
O	1.87542047849838	1.19529087246915	-1.64871873257056
O	1.48027822948204	-0.63275633841479	1.19156773072066
O	1.52456706082798	-1.36366942206929	-0.93969710103888
C	1.77689746265512	-2.72879424127477	-0.50968178554111
H	2.46747594052608	-2.70144632970832	0.33325423687805
H	2.26899616249148	-3.18777849876733	-1.36624537055064
C	0.48466452936673	-3.43516431609016	-0.16061124747414
H	-0.01581351510481	-2.94710682696516	0.67714601156369
H	-0.19273441715817	-3.44883213488884	-1.01641425511699

H	0.70924965741402	-4.46678154537716	0.12123255555930
C	0.80144199150055	3.30681553581426	0.14938129044255
H	0.00822985312696	3.98969330769019	0.43914534453176
N	1.93065491396461	3.91107447389304	-0.12445979540192
H	1.93999108624559	4.92560863414030	-0.14432524852844
N	3.15077355737486	3.25125235298994	-0.29765888675819
C	4.22813827923079	4.05692637601891	0.27491525544982
H	3.30025943892815	3.14007647688629	-1.30204480823689
H	5.17161681421349	3.57265689735518	0.02168057817777
H	4.24223790625102	5.08401713495520	-0.11464586860480
H	4.12640992536907	4.08918581528289	1.36120158713988
O	3.49184992835765	3.66664635786662	-3.32534957831585
H	2.54918708534536	3.60020875479190	-3.51251850326374
H	3.89703208943006	2.91801967393510	-3.80854986412035
O	3.88599145404031	-0.41686325618006	-2.78677846039699
H	3.13098612627261	0.03376161007537	-2.36215964162239
H	3.53208154298014	-1.25344923792798	-3.10773546027258
O	4.73778631926873	1.50037751437519	-4.58877027727175
H	4.46815124626227	0.74224586052875	-4.02847090597718
H	4.37989182975327	1.31318636550964	-5.46343538239968

GS4B-E

C	-1.99407718736352	3.21228758912988	2.11448643604739
C	-2.88626984489169	2.14597570947141	2.10773627446696
C	-2.40757019056379	0.87765138086668	1.83460349131434
C	-1.05758906748408	0.67562212418864	1.54691657989886
C	-0.18343975079624	1.75734960910191	1.56286911319424
C	-0.64631827721216	3.03430228073898	1.85441594908144
H	-3.93654931534349	2.31384063770909	2.31192468690747
H	-3.08536721692035	0.03243999758065	1.82506627835180
H	0.87077532462843	1.60712238310666	1.36066108048816
H	0.03135368508496	3.87862934980019	1.87612616080394
Cl	-2.58802524782420	4.80558971924941	2.45967571400354
C	-0.56686709587234	-0.71400975864627	1.28786378504900
C	0.53142453523891	-0.91801667784583	0.35560441201269
C	0.66423532111471	-0.15361533783596	-0.86011691588770

C	-0.46540163404935	0.78980800907236	-1.31347822975590
O	-1.10595377899408	-1.65151277435700	1.88621753091661
O	1.60669926111000	-0.24042873448070	-1.64308896141637
O	-0.26835597432012	1.92254803805241	-1.67862950563881
O	-1.63719990274276	0.17123001676708	-1.33011995223188
C	-2.78374956864999	0.96741251107842	-1.72983023397030
H	-2.61843674414858	1.31744557981753	-2.75008424959495
H	-2.83829920890007	1.83105315349702	-1.06410993076027
C	-4.00552228240486	0.08703283120909	-1.61840115573226
H	-4.14174931127455	-0.25864894143635	-0.59150173078156
H	-4.88801356990723	0.66014561413338	-1.91134660401414
H	-3.92100120940804	-0.78018090201693	-2.27676841739709
C	1.41844498295869	-2.01293100525449	0.42830743618375
H	1.92382955830223	-2.30004943269470	-0.48686079882858
N	1.37561873168034	-2.37414903820122	2.76139417578135
H	1.74063173151697	-3.08002742557783	3.39094335692111
H	0.35467752103119	-2.38094338298101	2.80829634177509
N	1.76371620392291	-2.72134748279744	1.46491785615808
C	2.66729370008944	-3.86475041196593	1.35976429079197
H	2.13507728729865	-4.76922996967128	1.65905949907806
H	3.01132808080278	-3.95728474692272	0.33275555239203
H	3.51835045429135	-3.70186051188511	2.02363068439188

GS4B-E-2H_2O

C	-1.81475809372771	3.14997612351324	2.26424601730338
C	-2.76007475241295	2.13496433972073	2.17296778833150
C	-2.35016963088613	0.87597706588447	1.77349495556313
C	-1.01454076938276	0.63534269839882	1.45043329195187
C	-0.08692487735685	1.66826866723897	1.54523632206496
C	-0.48079255371823	2.93272348252293	1.96139323672575
H	-3.79805755264488	2.33473153366734	2.40849520746986
H	-3.07448249050956	0.07486372355619	1.68771703961162
H	0.95425089890163	1.48486940157542	1.30688341895627
H	0.23743940731332	3.73855533488398	2.04663982507229
Cl	-2.32200710685210	4.72978737209664	2.76803725873210
C	-0.59091573449226	-0.73534809093535	1.03822275165142

C	0.55374834035554	-0.88042913674360	0.12516808277235
C	0.65687541415567	-0.11194346928694	-1.08487059874705
C	-0.49622490130692	0.83088338130536	-1.47480578670476
O	-1.21102549074352	-1.71535128373629	1.44461449554757
O	1.58906879167495	-0.16212190387434	-1.88443206791691
O	-0.32392047672722	1.97898840460213	-1.80233846975326
O	-1.66382625116944	0.20323689576003	-1.46153450378465
C	-2.83318357512988	0.99957695278711	-1.78982652221301
H	-2.70579039480509	1.39375947107000	-2.79927732294392
H	-2.87599586881738	1.83440322123821	-1.08737408803908
C	-4.03909425947093	0.09802665312866	-1.67534109362331
H	-4.13485715958724	-0.29436623774298	-0.66074067059508
H	-4.93856973002236	0.67072477726973	-1.91190763922930
H	-3.96652865462747	-0.73826411345409	-2.37387072718601
C	1.51378393346474	-1.88367690403082	0.27106517086171
H	2.09029540866872	-2.15796354872902	-0.60664158750828
N	1.33793296147317	-2.12956397267303	2.60381384560443
H	2.10051581548959	-2.20393567650512	3.27142425849969
H	0.61350876646697	-2.79026463659347	2.89453392112841
N	1.85356660862607	-2.52454984193567	1.36084674153391
C	2.79633105729134	-3.63957600255180	1.34857894239937
H	2.28372696133669	-4.54745526228566	1.67225952256074
H	3.18678000713253	-3.77540742166562	0.34178823453209
H	3.61527099495065	-3.42086087986842	2.03691710755673
O	-0.61609788633958	-4.13013405661588	3.97902357125645
H	-0.92610542153404	-4.76234049876373	3.32148865550279
H	-1.33558422367348	-3.47300576414640	4.05136857335474
O	-2.50720549965470	-2.04772022537975	3.87340429372367
H	-2.17814973849184	-1.81612881610694	2.98240738952647
H	-3.42531227321684	-2.31115175659491	3.74699115844901

GS4B-Z

C	-3.88489226279540	-0.63330438501026	0.33477885510775
C	-3.04063702215817	0.09341748871225	1.15907256825773
C	-1.69152548279917	0.16633796499246	0.84029947896769
C	-1.19242502045015	-0.49496855539478	-0.27907906469672

C	-2.05853209353539	-1.23609742929283	-1.08076906907538
C	-3.41094046012677	-1.29980076009239	-0.78709848802695
H	-3.43091184970570	0.59443299122599	2.03628382893203
H	-1.02544387257065	0.72629289611753	1.48699514610829
H	-1.66774150874052	-1.75925304186418	-1.94574904650379
H	-4.09150618704399	-1.86055914770374	-1.41593302683952
Cl	-5.57604199093209	-0.71646617890278	0.71590076993138
C	0.27016336621522	-0.49256162554730	-0.61258159761655
C	1.03056940591370	0.74266015974967	-0.47344803422488
C	2.45202811765640	0.66348048897755	-0.58018171037102
C	3.15200976989590	-0.68112546489121	-0.32993342876954
O	0.78855923049992	-1.54322004807488	-0.98899645990506
O	3.21628433018306	1.59256861670828	-0.86989865972183
O	3.88684841462697	-1.19953366644186	-1.13486050347934
O	2.96398144572460	-1.08299235896186	0.92132333769805
C	3.65236201511905	-2.29054115667982	1.34032530128387
H	3.71474858249401	-2.19733365832641	2.42385358202950
H	4.65785485876428	-2.28024932209692	0.91894447731914
C	2.88256501691366	-3.52869752717409	0.93194359194017
H	1.87121853395170	-3.50719695165510	1.34329910808338
H	2.81987597343733	-3.61197592891334	-0.15384134811558
H	3.39711348155278	-4.41105009047048	1.32083616222434
C	0.31557259606374	1.96848588688167	-0.48486866907162
H	-0.68703768421827	1.97467267924724	-0.89543064068056
N	1.84332439264964	3.30135224707732	0.72842936607217
H	2.63370905534490	2.91837212187869	0.20116267118970
H	1.98363104134263	4.29814854486576	0.85221936294596
N	0.68720522250530	3.12890082952860	-0.03255102930958
C	-0.16074194172901	4.31392320425468	-0.15229558438930
H	-1.10233504029417	4.03532099886905	-0.61799460080057
H	-0.34095288151324	4.71369373424698	0.84688692190115
H	0.35326503775788	5.05910364416051	-0.76134525839453

GS4B-Z-2H_2O

C	-4.10864531340478	-0.28760092197705	-0.44682732333630
C	-3.17510897007155	-0.57954970236889	0.53461588747790

C	-1.82764039146705	-0.58692828597922	0.19874797754963
C	-1.42404078726812	-0.32060063597290	-1.10588901092514
C	-2.38129950433597	-0.04935848515629	-2.07982930244505
C	-3.72869075585523	-0.01897675456058	-1.75407672238882
H	-3.49538971962253	-0.79658609123801	1.54625803857279
H	-1.08989396160590	-0.81377190335826	0.96048809597046
H	-2.06845338821252	0.14686912431423	-3.09927204597011
H	-4.47710082947485	0.20627208543519	-2.50402503557102
Cl	-5.79548241672080	-0.26090349710479	-0.03256566205720
C	0.02276987202696	-0.39811757883993	-1.51503256305694
C	0.99959139145835	0.39171447714355	-0.79403298524827
C	2.42914960846460	0.12580766998585	-1.09226070582444
C	3.33749977576966	-0.44386622185287	0.02247192439235
O	0.32081698163043	-1.15155715152993	-2.44206208572606
O	2.91701982059029	0.22584043401608	-2.19793862351908
O	4.52725924379282	-0.57365781338783	-0.12051892493046
O	2.64486958170885	-0.77951162420934	1.09935837905797
C	3.38871600364788	-1.31718239130610	2.22419827387166
H	2.76068128126702	-1.09829627396668	3.08677139557816
H	4.32771684436123	-0.76997362276925	2.31049815441326
C	3.61262673312738	-2.80579151160091	2.06079572331700
H	2.65912963698086	-3.32811706035649	1.95606233726131
H	4.23144199848358	-3.01586971736043	1.18680980012999
H	4.12251390264808	-3.19069322286859	2.94732022759726
C	0.59766491811240	1.39843668494202	0.05208300803630
H	-0.46042454668444	1.56366797213524	0.21130146046072
N	2.75491815920446	2.20056248594653	0.44377240902568
H	3.02748649045431	3.03823215222882	-0.07203212955784
H	3.23950455675091	2.16532224708963	1.33540070571403
N	1.37621970814384	2.26833550807130	0.67344622213331
C	0.86297277296333	3.39454944797349	1.44027997138998
H	-0.22035796862310	3.31614676961846	1.51712313484083
H	1.30104057517215	3.38191389617223	2.44050451080529
H	1.13054530493550	4.32833797380043	0.94002934889963
O	2.49513378622634	4.27485020773120	-1.70572388129546
H	3.29166674006729	4.65051370741419	-2.09581884268168

H	2.17320603355110	3.62759731246588	-2.36358189100236
O	1.51291689131137	2.32606489870883	-3.51207746943822
H	1.80405678767945	2.44878604852604	-4.42265414869388
H	1.94819315281563	1.50838936404489	-3.21051763282708

GS5A-E

C	-2.74474794800590	2.32907986644403	-0.00022356891734
C	-2.02946309129790	2.82318099790194	-1.07859778401117
C	-0.73148048091909	2.37618932976078	-1.29024298881037
C	-0.15380594042252	1.44202104878566	-0.43814744074459
C	-0.89253919215279	0.96432711119259	0.63987406899471
C	-2.18915948689994	1.40156943218895	0.86714106175367
H	-2.48015804779101	3.54758787179073	-1.74620535971615
H	-0.16823262639354	2.75957290468094	-2.13061703482383
H	-0.45595706148130	0.23683159087379	1.31507000957495
H	-2.76230906991051	1.02499576081656	1.70555844230274
Cl	-4.37118945392247	2.88103927436049	0.27023565743164
C	1.28504268529095	0.97327866572476	-0.62140762085209
C	1.48631984990007	-0.48461881094014	-0.25885915299049
C	0.79007271174682	-1.56556975019425	-0.86578646835486
C	1.14026588488921	-2.97943813135623	-0.36356534769543
O	1.65394187911170	1.27877457917457	-1.95463944571676
O	-0.04603095933193	-1.46334015390606	-1.75864997970786
O	2.27384213039745	-3.37162940219846	-0.20853001286213
O	0.04572612234988	-3.69800679510545	-0.16852166216136
C	0.22242678197158	-5.06469038425011	0.29125721017940
H	1.07371526333309	-5.50003964683654	-0.23204428101024
H	-0.69060013813927	-5.56992009748449	-0.02088223908425
C	0.39857090754632	-5.10795743178050	1.79460782995226
H	1.30855611031549	-4.58742653254869	2.09822048767862
H	-0.45756174779447	-4.65030770095610	2.29513288603379
H	0.47149117452323	-6.14946477459165	2.11712374188569
C	2.35368919061432	-0.54970990488535	0.80210649484437
H	2.76193116201673	-1.41884633977992	1.29775042837220
N	2.69459017597169	0.66375390440309	1.21029837306637
H	3.54401243066264	0.86502446460729	1.71939203874568

N	2.23973902756051	1.64629936490161	0.30336161052398
C	1.82598782715451	2.86612092385465	0.98362029094018
H	2.57898775339636	1.02212751998436	-2.05161655352385
H	2.68923236270680	3.27487233472353	1.51174280638565
H	1.51154452527130	3.58715927641339	0.22962306596540
H	1.00898112773196	2.70305973423025	1.69488525635146

GS5A-Z

C	-4.36971269490257	-0.26639806211528	-0.01246062377620
C	-3.61071591007400	-1.36571796294196	0.35502468278987
C	-2.23990428907494	-1.21275392653210	0.51606392596416
C	-1.64121570972998	0.02934048240125	0.32543572277970
C	-2.42871440500424	1.12473143062138	-0.02346627991066
C	-3.79479951673682	0.98207357039745	-0.20638180722924
H	-4.08339868355871	-2.32675469695432	0.51606543906511
H	-1.64433931071742	-2.06603787295740	0.81856179202019
H	-1.96384568157624	2.09445979839982	-0.15970211112011
H	-4.40910984899625	1.82708811243265	-0.49225555689452
Cl	-6.08259616293005	-0.45229301006426	-0.22850629621439
C	-0.17245587473712	0.24327956579274	0.53815985094168
C	0.76467568508474	-0.69439227602182	-0.01543064991191
C	2.23499018560134	-0.65413166125721	0.30606202127332
C	2.90276120305202	0.72792073467627	0.17352691917311
O	0.21288940132165	1.23746470161660	1.15554662626481
O	2.44947733174251	-1.10993950009493	1.62495825491133
O	3.65372403556995	1.15974998618250	1.02029842053328
O	2.59834206274842	1.32813055857820	-0.95948628916046
C	3.17226554641981	2.64147060685297	-1.19259480384030
H	3.15488640506627	2.74922274698932	-2.27626740323076
H	4.20587973366856	2.63524101967617	-0.84639509959549
C	2.35258333478337	3.71756054817570	-0.51249389304671
H	2.36677479200035	3.59612372077776	0.57176154424663
H	2.77380542058604	4.69545489542093	-0.75826584875270
H	1.31721652555628	3.68866098200535	-0.85875221168057
C	0.56010608016826	-1.74987999821988	-0.84944365459059
H	-0.36257839412071	-2.11027365167021	-1.28237877761957

N	1.71340676162735	-2.37072310890341	-1.14433322098197
H	1.84241116184311	-2.81838955134857	-2.04221617919056
N	2.79431511249450	-1.54020829966224	-0.72990104372476
C	3.96835729677191	-2.31284480506330	-0.34538179340820
H	4.32652797427973	-2.85634695439068	-1.22092287373096
H	4.74710464574545	-1.61650114490131	-0.03091456376500
H	3.76281518580135	-3.01701742799999	0.46582458416553
H	3.17724206022604	-0.58661448989818	1.99402923724690

GS5B-E

C	-2.75502816507795	1.35271570125633	-0.16906328382107
C	-1.90177294070986	1.05065973443435	-1.21789557532015
C	-0.55717710795740	0.84102415408806	-0.94887899259189
C	-0.06633363780318	0.92926676507673	0.35127298915754
C	-0.94133106028726	1.24343235055935	1.38481300595197
C	-2.28980328614655	1.45675169400253	1.13162690544173
H	-2.28433470223891	0.96930906731462	-2.22788164499571
H	0.11446870658845	0.59921259716385	-1.76497983712183
H	-0.57005583840507	1.30990128015889	2.39920452589519
H	-2.97347268594515	1.69274564492174	1.93805455366321
Cl	-4.44679682485365	1.58545537860763	-0.48981047708854
C	1.42073214048709	0.71471459005240	0.62096315070541
C	2.09680566499461	-0.31625523582703	-0.26767295634196
C	1.72718633081676	-1.65834708546349	-0.51641700149930
C	0.49557631032372	-2.17729429398208	0.24432745417716
O	1.66023865310878	0.45377177514024	1.99921405170293
O	2.31966746012340	-2.45657744274941	-1.24724995353973
O	0.35784613505392	-2.02961582553632	1.44169568994988
O	-0.33753936867908	-2.82843991719872	-0.54193645973834
C	-1.53846929762476	-3.37833927017253	0.07008980097056
H	-1.26850406845365	-3.80768702460118	1.03468077684287
H	-1.83927008746790	-4.17394765733116	-0.60978039998765
C	-2.60791828445328	-2.31459034109103	0.19936266241635
H	-3.50828241470321	-2.76401721133487	0.62526886349076
H	-2.27995952260685	-1.50559740501178	0.85356698039711
H	-2.85738233559998	-1.89732339168985	-0.77808539342088

C	3.22336027576683	0.28892389156359	-0.79112649474576
H	4.01696916269970	-0.16246876251883	-1.37185962473420
N	2.16662614710199	1.95142301327742	0.30924720519864
H	1.60130276656969	2.58003491493353	-0.25564939972509
N	3.27733039719169	1.56340766147071	-0.47776564411447
C	4.33102488578185	2.52504456136007	-0.71131753055895
H	4.78440200237547	2.81770347047699	0.23781165130520
H	5.07784858505372	2.06360672567843	-1.35527049803533
H	3.91864450229984	3.40747772166756	-1.20523480226727
H	1.33506924267620	-0.44741438869674	2.15650083238164

GS5B-Z

C	-3.48577150552963	2.27248542656502	0.88420954229342
C	-2.41591448041103	2.87815487745624	0.24558405686352
C	-1.38646756231237	2.08319512733427	-0.24161852117957
C	-1.43510704672641	0.69911656413415	-0.10212112335740
C	-2.53220986600960	0.11233337004462	0.52530828432425
C	-3.55798638473451	0.89425337885140	1.03233911467735
H	-2.38851009929614	3.95442103187011	0.12729560208178
H	-0.55484643476445	2.55060693886409	-0.75603182008446
H	-2.57649410908238	-0.96644812117033	0.62250719366496
H	-4.40568497600803	0.44366761362777	1.53402621617300
Cl	-4.77170335027596	3.26002019912699	1.50777707179992
C	-0.36050487200297	-0.19235020160506	-0.65309716153907
C	1.00561387664120	0.14285728272111	-0.42972113912552
C	2.14167923634122	-0.62600666791583	-1.05038549436399
C	2.05354930009927	-2.15414675436479	-0.86895346395991
O	-0.68168033849398	-1.21985637883441	-1.26396343730783
O	2.26301328247513	-0.36432389687760	-2.43039133732857
O	2.23621876752470	-2.92355238311828	-1.78660583095553
O	1.80937292082037	-2.49226201506997	0.38073147828207
C	1.70624740068056	-3.90896138094560	0.68266160305135
H	1.94535897520844	-3.97209677213374	1.74337125362134
H	2.46362474472912	-4.44275975677293	0.10855904677954
C	0.31027687506801	-4.41884209198639	0.39396919823391
H	-0.43063755804271	-3.85381725180533	0.96379745862974

H	0.07537194442482	-4.33967085566716	-0.66874579555578
H	0.24583461565511	-5.46964994669807	0.68683504836976
C	1.55953095347887	1.11000418208755	0.37278597520466
H	1.06345604833064	1.85539790615215	0.97888235222813
N	3.31524489106783	-0.12375148412046	-0.30122774462420
H	4.03433130605933	0.14484222298485	-0.96876457772370
H	2.44855222114430	-1.21261870824870	-2.86341211479552
N	2.87963296126495	1.03966571374187	0.38487182137074
C	3.79355917552664	1.65039298608687	1.32457365621550
H	4.08903740470337	0.92643398731727	2.08864938182697
H	4.68085440606058	2.00450720422099	0.79686686660961
H	3.28946658638569	2.49686579414735	1.78887523959953

P-AE

C	-2.75824666869170	0.74064418174987	-0.32478327941207
C	-1.98372560882694	0.37635130821428	-1.41409211637205
C	-0.60268977371191	0.47846175563051	-1.32063851690400
C	-0.00768999830852	0.93918814234659	-0.14971509540291
C	-0.80655073708143	1.30608353920056	0.93154549746407
C	-2.18583896154855	1.20742159787849	0.84991847374479
H	-2.45162744531454	0.01623672117563	-2.32185143323402
H	0.01436452669200	0.19900045489781	-2.16686499959734
H	-0.34783909972805	1.65483280240639	1.84989589421754
H	-2.81110555447906	1.48084393795270	1.69070557499589
Cl	-4.48468883012116	0.60248743888611	-0.42738230690530
C	1.46159379339266	1.01792110867946	-0.05504561045316
C	2.44293180652665	0.03232817063889	-0.18594080371028
C	2.31147507312195	-1.39687777873693	-0.39835963269497
C	0.92787672635618	-2.05299193867845	-0.26160517050847
O	3.24602626327882	-2.12818815734502	-0.66859021733070
O	0.41390439771623	-2.67631259993700	-1.15710484898884
O	0.43426523906487	-1.87918017054339	0.95097200546152
C	-0.88929026134224	-2.42922607012512	1.20024573220342
H	-0.86038490284942	-3.49822334213525	0.98453612658643
H	-1.58231767819012	-1.94635427145045	0.50878750458993
C	-1.23351805945624	-2.14628431492478	2.64258637685055

H	-2.23002489059009	-2.54029813091237	2.85443497357964
H	-0.52003027327349	-2.62827143632170	3.31427184378726
H	-1.23750011948813	-1.07222615353913	2.83719706602772
C	3.66391171582098	0.72702171684876	-0.02724226692599
H	4.66560347238338	0.32271333332143	-0.05051606508875
N	3.45784977022716	2.00784784598602	0.18795037922516
N	2.11559190979900	2.16821445680566	0.15936589868781
C	1.55542781850031	3.49775358025950	0.34613775393920
H	1.45059958936884	3.71673412569902	1.40954819264216
H	2.23111476195310	4.21542914486486	-0.11442670255008
H	0.58152797879946	3.54617346120705	-0.13625243792417

P-AZ

C	-3.19211521727618	-0.33117090933815	0.54060802551433
C	-2.11050530492123	-0.16105547021505	1.38933518866832
C	-0.85942514974050	-0.59378442038896	0.97477408056647
C	-0.69903438225397	-1.20195383276646	-0.26738063438789
C	-1.80514827356457	-1.37663054213708	-1.09773655583025
C	-3.05604211638271	-0.93447691672437	-0.70335967959067
H	-2.24301306624480	0.30696825522468	2.35668389951661
H	-0.01168634476462	-0.46889683953177	1.63762003848185
H	-1.67729283302234	-1.85214953201900	-2.06273889208465
H	-3.91733631261445	-1.05096967251385	-1.34941227568771
Cl	-4.75452684173024	0.23165681673637	1.03859367971335
C	0.62614681841012	-1.72494643970004	-0.71269936313748
C	1.85661278866108	-1.17431145082777	-0.10055430256794
C	2.23115758835880	0.13742641151273	0.15316556387743
C	1.56354680147368	1.41816062893106	-0.18560417176855
O	0.70187930353483	-2.63167285001374	-1.52361087947736
O	1.70107575284045	2.43787501093642	0.44947012965496
O	0.81998500809395	1.28631736688829	-1.27686856588544
C	0.05823542447653	2.44717163128920	-1.70524219484778
H	-0.11273487609526	2.27029676024519	-2.76609655871057
H	0.68134063823056	3.33277928545980	-1.58165448926960
C	-1.24410144203817	2.55684917897690	-0.94158665101606
H	-1.80503556208769	3.41500437572682	-1.31984386192940

H	-1.84812935812698	1.65898953201066	-1.07995359376528
H	-1.06516944601547	2.70292571029941	0.12479505443757
C	2.92181654163283	-1.93729286283388	0.40290894450136
H	3.01901275521673	-3.01315486639067	0.42288373872961
N	3.86154561602448	-1.16152326508076	0.92508606670406
N	3.43281142043641	0.09258186587351	0.76409846303792
C	4.27025079512061	1.20128792750748	1.20603152913194
H	3.82731714011366	1.68100669855320	2.07752433501619
H	5.24045802996553	0.78141208153187	1.45939962632278
H	4.37631349428896	1.92852407277794	0.40299436608188

P-BE

C	-2.85874976143516	0.97059050900478	0.35990795602820
C	-2.12523907757411	0.39028239268955	1.38185422937448
C	-0.73963650426938	0.39260139969583	1.29899022434660
C	-0.09515490341181	0.96429328151063	0.20504812370461
C	-0.85446275422196	1.55308779861676	-0.80391731568507
C	-2.23885468644839	1.55818131937908	-0.73313018712522
H	-2.62821225605360	-0.05606961981016	2.23082822119859
H	-0.15895153247264	-0.04872166233400	2.10129749207799
H	-0.35807326216526	1.99433875937857	-1.66039171314650
H	-2.83246787978715	2.00408818763136	-1.52173600465350
Cl	-4.59349278604640	0.95889704467400	0.44643500947337
C	1.37783922216420	0.94948413591049	0.11337708651222
C	2.27901113849033	-0.13742494315583	0.28087876833334
C	2.04880562166401	-1.55874304990269	0.47643018405535
C	0.64820979837461	-2.13390906726297	0.19650066627115
O	2.91025123536731	-2.34427292670133	0.82320081613334
O	0.06189623947720	-2.83073733505686	0.98692187736773
O	0.22903440420072	-1.80162906145936	-1.01083093789703
C	-1.10567075993223	-2.24336106022342	-1.38253675840513
H	-1.80727263348477	-1.80483993308059	-0.67027175130103
H	-1.14281489732993	-3.33014780794917	-1.29464593361915
C	-1.36078382580485	-1.77479311346111	-2.79485087784691
H	-0.64027206195029	-2.21564621093440	-3.48703282108695
H	-2.36450119424913	-2.08250294035488	-3.09655570565315

H	-1.29827333916161	-0.68691218096165	-2.85917080685455
C	3.53697547530216	0.43695924709225	0.13793843452360
H	4.52169330018451	-0.00386931274461	0.16902095298563
N	2.04288313607269	2.06928152820410	-0.11575038838385
N	3.34841870257331	1.73016649498703	-0.10375266450522
C	4.36058072222817	2.74680564374742	-0.33653609089580
H	4.28601371241661	3.51521250270776	0.43273759723339
H	5.33879417572865	2.27214730034203	-0.29250058986177
H	4.20738778155420	3.19128135982139	-1.31969884269874

P-BZ

C	-2.22535516630860	2.89544550952522	1.75623365652908
C	-2.95103391385049	1.83783358879109	2.28724682399447
C	-2.40883716350321	0.56480637014261	2.23667135879819
C	-1.16325546025821	0.34790672545516	1.65112057212060
C	-0.44750806583697	1.42633918416974	1.13810858275701
C	-0.97393371220162	2.70799067038163	1.18919780957176
H	-3.92613049809586	2.01164734920227	2.72515401380193
H	-2.96062547911352	-0.27638926005794	2.63855403156514
H	0.53482390572074	1.27759432297977	0.70595324938032
H	-0.42154531773780	3.55138088667379	0.79414442646260
Cl	-2.90064868737418	4.49186999595721	1.80479495420488
C	-0.62209028566814	-1.04271177718158	1.62028473962260
C	0.41429987505176	-1.40471644213602	0.62949851486731
C	0.63706445863548	-1.03571257011739	-0.71881836211485
C	-0.17915255964888	-0.19948057188979	-1.63920895193164
O	-0.99543153511096	-1.87090267857041	2.43536619208932
O	0.27972153664044	0.42304301788673	-2.56942002329502
O	-1.46579327323932	-0.22938767406522	-1.31366359024232
C	-2.34200494938334	0.64545039414498	-2.06757624252678
H	-2.34573875703526	0.31240863335869	-3.10705562893210
H	-1.93189210682084	1.65620451555120	-2.02392007890529
C	-3.71297103276205	0.56595985320929	-1.43870197291231
H	-3.68271214871285	0.90864347556420	-0.40295277076060
H	-4.40042168959624	1.20571764016150	-1.99666057450020
H	-4.09447064398902	-0.45715063020855	-1.46372513826794

C	1.47539623867510	-2.24317354942682	0.91727769498555
H	1.74287601935549	-2.75274697451609	1.83021697137946
N	1.73724151006834	-1.60219265899123	-1.19476343030575
N	2.23090237752980	-2.32172629091743	-0.18663670901816
C	3.46241964228789	-3.07600551568309	-0.36242608126077
H	4.27643006930191	-2.39161107357203	-0.59983451696964
H	3.67722569179387	-3.59902398493021	0.56726868417617
H	3.33345112118655	-3.79501048089129	-1.17092820436303

TS1A

Imaginary frequency: -267.07 cm^{-1}

C	-3.20208219628493	1.54692062518171	2.72920583383118
C	-3.80658162422888	0.80068038298678	1.72416085951248
C	-3.03045068633520	-0.07356285288110	0.98419817432122
C	-1.66078226373114	-0.18302175373000	1.22211692659894
C	-1.07939439772364	0.57278135686751	2.23373789332508
C	-1.84968232356848	1.43782183886034	3.00160956529616
H	-4.86666416678834	0.90754721392346	1.52925812305556
H	-3.48266645967210	-0.66766433681004	0.19864920592708
H	-0.01800708621099	0.48172329308874	2.43362070794604
H	-1.40307697494031	2.02214279918138	3.79659342755065
Cl	-4.17136018806847	2.64212367542937	3.66561007018251
C	-0.85423670312097	-1.17203375442254	0.43156883152629
C	0.48312389343156	-0.82312559275269	0.01917483781037
C	0.86837593598613	0.49930785007330	-0.33044405759754
C	-0.19194709700749	1.60086709597901	-0.49758593026503
O	-1.36619127281937	-2.27389809146817	0.19299814035708
O	2.01275071995971	0.85888941862638	-0.63998231209100
O	-0.12311741306711	2.69313213338887	0.01098694149278
O	-1.12237951683690	1.22543757604434	-1.37026154129138
C	-2.17481419502134	2.17965127269587	-1.65731788159874
H	-1.72086818577009	3.07130297175195	-2.09348434908314
H	-2.65014727647674	2.45110512533614	-0.71270980685093
C	-3.13977179669143	1.50765973790924	-2.60541636688046
H	-3.57354140434745	0.61603448534044	-2.14734490273850
H	-3.94794205787639	2.20079523741827	-2.84925194338093

H	-2.63681639618266	1.22071927420320	-3.53167430739425
C	1.45286765273975	-1.84237034305421	-0.34285673264596
H	2.26028985403870	-1.48832909619163	-0.97046356324480
N	1.80603588521867	-2.87158687631478	0.43599889443145
C	1.01979828965445	-3.33747257669844	1.56448369505280
H	1.68971690803026	-3.86158582264668	2.24757244015386
H	0.58265963470320	-2.48821221885719	2.08734131742133
H	0.21955450073856	-4.01495086829857	1.25469190284177
C	2.83000436836118	-3.78577322737396	-0.04720165241149
H	3.44414231820083	-4.12239219077527	0.78964500953923
H	2.37846753303035	-4.66174723138200	-0.52593051682555
H	3.46161875831813	-3.27401086003770	-0.77364622621456
N	0.48920500025536	-2.67911186994177	-1.94524449003001
H	1.10656889322455	-3.24102488856974	-2.52578430990699
H	-0.17534599354568	-3.29925414638138	-1.49231662695552
N	-0.25148743151953	-1.80086103418042	-2.80046678962817
H	-0.74844047602208	-1.17801732827012	-2.16869571344956
C	0.67022903869533	-0.98990855015073	-3.59600496958111
H	1.19111264970573	-1.63159473963163	-4.31128014033622
H	0.08010041127282	-0.26004718129417	-4.15183385421486
H	1.41367333829281	-0.45788593217097	-2.99112381355694

TS1B

Imaginary frequency: -225.28 cm^{-1}

C	-3.09821749472486	1.69236627172862	2.50352273863988
C	-3.72920013657974	1.05591885368988	1.44081669307020
C	-3.01470711894998	0.13475730861766	0.69602817250582
C	-1.67631001017311	-0.12865776254389	0.98600949234723
C	-1.06822805339396	0.51775215363595	2.05554190744216
C	-1.77945695354660	1.42840150526988	2.82823381142164
H	-4.76219501929461	1.28212394938252	1.20635884219887
H	-3.49037746023491	-0.37653336863089	-0.13265667997688
H	-0.03320880844806	0.30526598007772	2.29744074044345
H	-1.31221432359681	1.92818785514245	3.66778358645194
Cl	-3.99122885097024	2.84556641637815	3.44649749536304
C	-0.94526687882676	-1.17232083183450	0.19231923173349

C	0.42678941135015	-0.95493504360117	-0.17980995996903
C	0.99894682234521	0.32389371085671	-0.41399987465988
C	0.11374778674975	1.57800926818393	-0.50642927916156
O	-1.55876232486706	-2.21788159782056	-0.07174147403144
O	2.19375670163789	0.53996703410007	-0.66305237438086
O	0.33055659170780	2.60127751009582	0.09575928353816
O	-0.83937643570815	1.42050846998330	-1.41898801101420
C	-1.73885530934072	2.53980452937416	-1.61996532876340
H	-1.15454060855763	3.39173112775517	-1.97221952533695
H	-2.18262337486855	2.79111733637906	-0.65467378402682
C	-2.77901206558949	2.10568577351840	-2.62544179736833
H	-3.34191034273483	1.24729695407767	-2.25273836261837
H	-3.47596993632052	2.92833847587266	-2.80048964988720
H	-2.31262262368971	1.83804962645172	-3.57613184633136
C	1.30221070439317	-2.06865950698590	-0.54508979820095
H	2.13249014542155	-1.79720538230110	-1.18649420808623
N	1.55640021320741	-3.10038720114932	0.25380912696593
C	0.72900821007652	-3.45977535958802	1.39378789365410
H	1.35028562040901	-4.00801241687912	2.10253740084778
H	0.35183520139516	-2.56135998943478	1.88023938004566
H	-0.11524552353177	-4.08555977041815	1.09506951315129
C	2.65133536058145	-4.01572463691612	-0.04362013511548
H	3.30352903566543	-4.09351424919254	0.82954369394938
H	2.27105109147719	-5.01199004076932	-0.28328017101159
H	3.22410398129680	-3.64106377182565	-0.89036666690013
N	0.10459636201087	-1.67042679531440	-3.08268932783714
H	-0.16859699105602	-0.86657886618260	-2.52295180443225
H	-0.67671162724772	-1.87136690684922	-3.70353619571545
N	0.27475154340392	-2.75901249353661	-2.18391472695570
H	-0.60506500641602	-3.02614642853660	-1.74795624802032
C	0.91278471696486	-3.88716240624878	-2.84696909901986
H	1.93390033673696	-3.61490960270990	-3.12021894465848
H	0.92902382545287	-4.73328826019306	-2.16055572914149
H	0.36589961638425	-4.17714742110873	-3.75051800114835

TS2A

Imaginary frequency: -876.27 cm^{-1}

C	-4.30864947827233	1.15275020462544	0.11603961747059
C	-3.59971818845579	1.36867802743298	-1.06012381281540
C	-2.33683994108623	0.82004420886208	-1.18946139972324
C	-1.77445117979272	0.08720137703591	-0.14391318646533
C	-2.50549170919884	-0.12720658012124	1.01778316055648
C	-3.78263423847387	0.40432758607888	1.15359474813689
H	-4.03420703300085	1.95870102964513	-1.85761093939987
H	-1.77105355503213	0.97647629449083	-2.10041074523915
H	-2.08416629988002	-0.72077130724926	1.82073385400482
H	-4.35821005457930	0.23828425871292	2.05567094207372
Cl	-5.89424463448969	1.83920000239043	0.28259996951193
C	-0.43303597030871	-0.52650502929136	-0.32068027319289
C	0.59831777644601	-0.44320323420479	0.59739411947347
C	0.64846101911366	0.47158011862609	1.70208438567208
C	-0.30681159928169	1.67124811994570	1.82389908503395
O	-0.31300423267124	-1.22253676743402	-1.41449135311516
O	1.50575178717462	0.42981213648717	2.58296136918544
O	-0.94569548115649	1.89972848959807	2.82244558999114
O	-0.24462991054739	2.45744096666256	0.75957014547568
C	-1.10735543597226	3.62483664181233	0.76836757879260
H	-0.82863303815803	4.25221358231830	1.61669745628911
H	-2.13389365450422	3.28006460176156	0.90668793502572
C	-0.92072479315250	4.33443393271795	-0.55154886715012
H	-1.19578814875815	3.68305381029744	-1.38356183095519
H	-1.56163165306886	5.21865194253154	-0.57709437392123
H	0.11592753414009	4.65403853928461	-0.67813648132804
C	1.81944712049122	-1.33130404630327	0.45226730716971
H	2.69064337404239	-0.80088839131035	0.84267086586284
N	1.70948014949189	-2.55956919667655	1.19683139208275
C	0.59700185978153	-3.42964068303995	0.84134244078552
H	0.54379967978103	-4.24174902504577	1.56933698117428
H	-0.34139247385183	-2.87601072005356	0.88128051561351
H	0.70043507603825	-3.87807455903786	-0.15848198942230
C	2.96596268768905	-3.28763821132529	1.31248887254340
H	2.86715826056933	-4.04973794183314	2.08864799655077

H	3.26524032132223	-3.79863621717560	0.38378467208141
H	3.76291593874557	-2.59875102008832	1.60059450621401
N	2.06114011886021	-1.56927001136723	-1.01313042919844
H	2.56984798367926	-2.44170836822148	-1.15159582887751
H	0.81292447413795	-1.52804379518949	-1.42500975361819
N	2.74864712365497	-0.46507800868493	-1.59650017217902
H	3.70138758010580	-0.47266134347307	-1.24168460555679
C	2.77117998575704	-0.60036495180965	-3.05151549890684
H	1.75569905977068	-0.50929956039535	-3.44132546441040
H	3.37514012420526	0.21291978348128	-3.45601171744798
H	3.19575366869511	-1.55703668546769	-3.38348678384875

TS2B

Imaginary frequency: -819.87 cm^{-1}

C	-3.07583925623282	-2.88750472486616	-0.20134201189256
C	-3.02878600419193	-2.27408846459099	-1.44810053807818
C	-2.27632768288449	-1.12331603697959	-1.59933750285912
C	-1.55382377559533	-0.60706203592799	-0.52361625704496
C	-1.62964744221118	-1.22637624851988	0.71877562336517
C	-2.39661990591454	-2.37213781303623	0.88821375385900
H	-3.57316591276580	-2.69683702936756	-2.28345596890907
H	-2.22735948025262	-0.63280601664650	-2.56439795902439
H	-1.09361602223121	-0.80961862396178	1.56324523949453
H	-2.45967300715493	-2.85896237031168	1.85335332747193
Cl	-4.01135543322283	-4.33617865018020	-0.00818262728960
C	-0.75673294539213	0.63128990324871	-0.69869807530278
C	0.54423977581429	0.78753150265914	-0.25224492600584
C	1.41802902308444	-0.30318298976673	0.06908055581737
C	1.09308510388584	-1.73337104438843	-0.38676755412582
O	-1.39577575589319	1.60762112098742	-1.27929612569480
O	2.53616750369104	-0.17310327470834	0.56571691483829
O	0.99038080363902	-2.03445528942102	-1.55294207942407
O	1.06855312267369	-2.58336044619051	0.62695587372060
C	0.79031001760959	-3.96892765705050	0.29788835352467
H	1.61561979029110	-4.34928845816756	-0.30672846894177
H	-0.12478538173264	-3.99847877281823	-0.29664429388951

C	0.64155755170694	-4.72070496515315	1.59923440331412
H	1.55849066832322	-4.66319793293232	2.18970267047728
H	-0.18518590065204	-4.31537038016458	2.18658323725787
H	0.43217873349519	-5.77110197762236	1.38477182993291
C	1.14367390009455	2.18687847892890	-0.16602358769957
H	2.23235638947691	2.11827074371850	-0.23350521407369
N	0.84853692027880	2.92248006361031	1.04719735136676
C	-0.54799303760980	2.92042859394101	1.46201541539870
H	-0.64895308671018	3.57031257241487	2.33312374899781
H	-1.19190023173025	3.31166594948220	0.67292550595814
H	-0.90844205226878	1.91796867659449	1.73677413062456
C	1.71966141149641	2.54263924711148	2.15247878012835
H	1.56430641298228	3.24159127723387	2.97739924212901
H	1.51958622020790	1.52513402792615	2.51884893524411
H	2.76339761841176	2.59642429355183	1.83939871354296
N	0.78381352328292	4.36286846720408	-1.29508461375576
H	1.75706841075740	4.57216275219394	-1.07262538148778
H	0.24819301028996	4.67617433601516	-0.49311049965781
N	0.64455278402440	2.94878152677689	-1.36674765565372
H	-0.59249247284600	2.42662598621391	-1.41122681089027
C	1.31845507517530	2.47971576783904	-2.58562121347898
H	0.84920790031243	2.95463194511693	-3.44557683586366
H	2.38105940237030	2.74043655011514	-2.55171700765883
H	1.21599371411702	1.39779741988834	-2.66069039776162

TS3A

Imaginary frequency: -123.59 cm^{-1}

C	-3.67664112446931	2.05628344936352	-0.50509765384098
C	-2.50410319366017	2.12127689738528	-1.23620697595799
C	-1.51679440650237	1.17158640871271	-1.00484367898196
C	-1.70885890497479	0.17281483842771	-0.05740803031484
C	-2.91411255791784	0.10355052273676	0.64105445413251
C	-3.89832294893876	1.05293688592445	0.43145189158843
H	-2.35898171549110	2.90352317947706	-1.97073822440076
H	-0.59380098970114	1.20843227941631	-1.57176773411222
H	-3.06953060957259	-0.68378007953394	1.36950002311771

H	-4.82772964797324	1.02260471818532	0.98659924190883
Cl	-4.90298348991123	3.25670871421824	-0.76318877583104
C	-0.65985161234565	-0.84920277223423	0.17612670239015
C	0.66023520879250	-0.55968218490046	0.43154199015301
C	1.15294562797799	0.75948005192181	0.72755727136579
C	0.22386833157755	1.83453142325089	1.31362023490822
O	-1.08664791682520	-2.08431531722014	0.07067537154543
O	2.34296458123662	1.06162822939578	0.68555209971373
O	-0.40126198743993	1.66239128495326	2.33327408968747
O	0.29156709643469	2.96999643995076	0.63943311516998
C	-0.54460596739526	4.05393446854558	1.12140618741291
H	-0.18604943908931	4.34845998269697	2.10934566962548
H	-1.56519891682257	3.67787289285197	1.21477396554779
C	-0.44758829184147	5.17902549498082	0.11878942398932
H	-0.80633327106061	4.85525087277363	-0.86071005492807
H	-1.06616089001092	6.01379873496187	0.45589048451217
H	0.58285588613568	5.52774404865136	0.02236289932331
C	1.72984730472341	-1.64343889928457	0.34718797870164
H	2.54636761348751	-1.39009600168358	1.03222723905531
N	1.13117040732466	-2.93781674203268	0.76494960231712
C	1.84333112871312	-4.12090591589617	0.27694699925134
H	1.34525167982632	-5.00246703841965	0.68333400696205
H	1.80862112632175	-4.15779549265576	-0.80892712922041
H	2.88929209373419	-4.11896605834677	0.60406419691925
C	1.01391458556625	-2.97089671010794	2.22931164080501
H	0.45748789350370	-3.86132835886977	2.52340015073563
H	2.00394620660728	-2.99038716932423	2.69978968524822
H	0.47553620755321	-2.08698255133694	2.57442070888655
N	2.35642570078302	-1.73839950952703	-0.95813045714971
H	2.94223020685236	-0.91420242422345	-1.07532085385447
H	-0.20616013442893	-2.68728705997153	0.30172501320315
N	1.40473638445059	-1.83244769438218	-2.01169747732899
H	0.98355257306252	-0.92010166697970	-2.17168139075222
C	2.06729601562221	-2.27311124507421	-3.23212572154825
H	2.43355623962991	-3.29339768243116	-3.09869143630087
H	2.91561624435556	-1.63305332158979	-3.51369609231554

H	1.33910167209977	-2.26376992275616	-4.04608065133926

TS3B

Imaginary frequency: - 565.37 cm^{-1}

C	-4.30976057193737	-0.78433224151491	-1.05089988885244
C	-3.65292617293442	-1.73511777946180	-0.27876873738304
C	-2.27053704723564	-1.71904821718176	-0.23056783540895
C	-1.55122630813641	-0.74732481640407	-0.92609166057192
C	-2.22663649725990	0.18518935374037	-1.70263953580797
C	-3.61510936641857	0.17018066708714	-1.77140509093087
H	-4.21980411992187	-2.47311674792408	0.27528467882477
H	-1.74365615104817	-2.45341487879610	0.36799657267654
H	-1.67146084755805	0.92865713271166	-2.26319681720104
H	-4.14682826093884	0.89512498487495	-2.37512510497226
Cl	-6.04516581818821	-0.79914076799701	-1.11158700493189
C	-0.06464525569471	-0.77230183707629	-0.87867535807671
C	0.73033698176713	0.28226270277814	-0.49535086089460
C	0.21547105183127	1.55647535638277	-0.05008119570442
C	-1.16344757353549	1.68641823027780	0.62946221750159
O	0.44936875016168	-1.89294574973730	-1.31980686011897
O	0.85399086845424	2.60156330442819	-0.09621869572222
O	-1.94585228077810	2.56204119533676	0.35392607837848
O	-1.31000613978603	0.79434598858596	1.59794793359604
C	-2.56069712590297	0.81526181229510	2.33594218780861
H	-2.67028228247912	-0.20585544141762	2.69884733600244
H	-3.36733038943903	1.03756693486650	1.63708633014082
C	-2.50106135278419	1.81132924086989	3.47440255389535
H	-1.67129299850818	1.57817242312259	4.14527440444303
H	-2.38059849261405	2.82895369364626	3.09913201759623
H	-3.43178692324371	1.75961517919238	4.04463799816625
C	2.25278461455844	0.16236249561411	-0.55127529079088
H	2.67908937271592	1.14220604070583	-0.78368894655282
N	2.61918574487883	-0.79363748780417	-1.62761939412358
C	2.42386520833394	-0.15748658271221	-2.93927272625953
H	1.41309033458931	0.24524944632481	-3.00807486490070
H	3.14247729910612	0.65669623863423	-3.08414096537483

H	2.56219961126624	-0.90492345885544	-3.72069812645510
C	3.97748120537119	-1.33605489762334	-1.52364523538044
H	4.07688510615071	-1.93089794936054	-0.62037191351685
H	4.15125193015667	-1.96915843934109	-2.39482093226522
H	4.72120759038249	-0.53178764719408	-1.51217481946580
N	2.28041098623957	-1.46257999297818	1.17176439849225
H	1.41010020232990	-1.27316908100376	1.67139966105978
H	2.92798413085769	-1.86077837440768	1.84287510827877
N	2.85033936577523	-0.24428170778777	0.71140132191036
H	1.53513581312599	-1.64599898205044	-1.46444570067540
C	2.81135079123710	0.79924765841653	1.72856324562410
H	3.41510568696877	0.47418954979191	2.57836630366094
H	3.23048065727095	1.72358375625013	1.32821081141320
H	1.79051867281363	0.99665969269564	2.08812240286888

TS4A

Imaginary frequency: - 187.17 cm^{-1}

C	-3.35644991724213	2.45778154110269	-1.13478938619604
C	-2.05674335683333	2.60518228746311	-1.58634782450566
C	-1.13520874933940	1.60290647641736	-1.31040852005643
C	-1.51621847502554	0.46467712483997	-0.60888531724921
C	-2.83951618990231	0.32616259296160	-0.19167475400747
C	-3.76430332546192	1.32515670220556	-0.43938335202698
H	-1.76572994389479	3.49236565613984	-2.13490181662312
H	-0.11085553215020	1.70970826617033	-1.64712343482700
H	-3.13504586855208	-0.56436235451955	0.35057633644653
H	-4.78793401945499	1.23657791717970	-0.09666611814329
Cl	-4.50631342509388	3.72299693939404	-1.43696391029870
C	-0.53493347144496	-0.63780844823935	-0.33841026444670
C	0.85001462838534	-0.28685579265324	-0.04879390173810
C	1.19751221724166	0.77043835887069	0.82595956351540
C	0.08179714418320	1.54866602674612	1.53165719434896
O	-0.93655485718160	-1.80095357220100	-0.36782922622759
O	2.34624735111779	1.08897393211684	1.17713190509899
O	-0.66222535207671	1.03887235627637	2.33890753255811

O	0.09608673404739	2.83595345815384	1.22057805045941
C	-0.95097834790505	3.65126406001564	1.80551895361174
H	-0.80865485233966	3.67113694563443	2.88755115767761
H	-1.90987816260963	3.17799235119807	1.58485699857007
C	-0.84870138253764	5.02772350076086	1.19189882030411
H	-0.99067687624027	4.97937493786350	0.11012259184963
H	-1.62585668918648	5.66825541041623	1.61496446190363
H	0.12402778071970	5.47735961990334	1.40283275305590
C	1.93856753147741	-1.13902971706146	-0.42409594462030
H	2.87145233535413	-0.99375295489650	0.10573489178222
N	1.56104738666620	-2.83193462984773	0.92992701087266
C	2.45125135345561	-3.94792207308898	0.65742022250366
H	2.21585133548470	-4.83051152832355	1.26568166528948
H	2.38017887583640	-4.22452920949383	-0.39625529164907
H	3.48120522370515	-3.64739139194177	0.87075809527638
C	1.57356343630863	-2.39035979514189	2.31545559272626
H	1.29537164421192	-3.19108511884624	3.01232628265814
H	2.57758367010362	-2.04190744837136	2.57315011627370
H	0.87491027408150	-1.56074625729750	2.43629161235001
N	2.09350103405588	-1.70294781897095	-1.61297907609833
H	2.95302713463901	-2.20444554474435	-1.80108122385204
H	0.61412506938615	-3.02676153759014	0.62380760385645
N	1.07095745431417	-1.78069848464521	-2.56192196490715
H	1.44331978660617	-1.40003016958176	-3.42475685529189
C	0.63334766066168	-3.16100947986607	-2.77499485345795
H	0.14517682971872	-3.52131647750922	-1.86964168266966
H	1.46340310006837	-3.83084752845339	-3.03374344896853
H	-0.09074819735795	-3.16231912854510	-3.59146124512785

TS4B

Imaginary frequency: - 206.81 cm^{-1}

C	-4.19954128266920	-1.06873741265535	-1.19570789127005
C	-3.74634313999474	-1.82905369987145	-0.12418657405093
C	-2.40489781889813	-1.77782148680220	0.21121652244835
C	-1.52925481870203	-0.95262204444879	-0.49298863904651
C	-2.00319462143111	-0.20663799689745	-1.56400830329647

C	-3.34398481123541	-0.26581743393225	-1.92784510867465
H	-4.43666535087093	-2.45003465225512	0.43355400450254
H	-2.03348707199564	-2.37054713187302	1.03953976943371
H	-1.32528834776756	0.42617678406467	-2.12524075234045
H	-3.71682441094494	0.31050689747772	-2.76558210108285
Cl	-5.88251088628520	-1.13203623697365	-1.62415292291735
C	-0.06608470515645	-0.97761945837913	-0.15771698809893
C	0.65162468812897	0.21484789644829	0.11541130922695
C	0.08002763246833	1.50092868351446	0.34129769900629
C	-1.41452250262135	1.68861447797146	0.64612594104981
O	0.48689464911848	-2.09962878727766	-0.22251762732319
O	0.72220363724038	2.55781519930480	0.40570049775353
O	-2.11858687305312	2.45418969035215	0.03305463662308
O	-1.77731471419804	1.00707823782681	1.72544956751612
C	-3.16148104065054	1.12186080399809	2.14709308428915
H	-3.35180116685060	0.19503536394395	2.68695462131897
H	-3.79060753763188	1.15396789113226	1.25737209538920
C	-3.36157746895559	2.33500684400770	3.03066347476204
H	-2.70623516839911	2.28719153913504	3.90317359484810
H	-3.15796970769767	3.25649563942092	2.48272303193170
H	-4.39755640832345	2.35928819676128	3.37769709933908
C	2.12853991271490	0.24154262375958	0.12380992446425
H	2.56113798716629	1.18603594387722	-0.17919965419142
N	2.48872902047922	-0.51887197828404	-1.77275594400341
C	2.15429182716478	0.50403666381893	-2.75627024070625
H	1.12376358866036	0.82771579148553	-2.60199612590244
H	2.81800289247921	1.36118637771622	-2.61695254034921
H	2.26512662367948	0.13880333888304	-3.78289047612811
C	3.86053041745949	-1.00170906096324	-1.86805208006018
H	4.02868309151722	-1.76149071869525	-1.10355569960645
H	4.07189136625660	-1.43521762731010	-2.85166224449659
H	4.54681742607959	-0.16907837020926	-1.69810564619178
N	2.37603252214937	-1.48277424157404	1.76840387570227
H	3.15381899707596	-2.12738619281823	1.88832704132954
H	1.69820599368398	-1.96950813064392	1.17516676634675
N	2.87407092730108	-0.40607108969152	0.99297093309400

H	1.84304858701457	-1.29846056976605	-1.82815856868151
C	4.24152359621759	-0.02842949016282	1.30637869144098
H	4.92703822079702	-0.84269558987041	1.05852960150326
H	4.50965042901391	0.85672911463328	0.73368167519868
H	4.31407582046588	0.18719540182152	2.37525066990035

TS5A-E-H_2O

Imaginary frequency: - 878.91 cm^{-1}

C	-3.06200010842927	-0.35183898435553	1.80810000242517
C	-2.00018083096903	0.23124224932896	2.48068474123874
C	-0.86097122765061	0.57566240619269	1.76641921897257
C	-0.78313225483575	0.34241733162564	0.39802910372436
C	-1.86383102025142	-0.23766491053545	-0.25546637478197
C	-3.00850044911639	-0.59076351785340	0.44349493926695
H	-2.05987082594722	0.40866414120028	3.54745164690824
H	-0.02712606636744	1.02261617020314	2.29468520007015
H	-1.80250848688318	-0.42302261250721	-1.31984653374174
H	-3.84877780718890	-1.04940703392759	-0.06339459189071
Cl	-4.48913731880238	-0.79587482182105	2.69379695579744
C	0.45219589863418	0.73637285038019	-0.39832976152064
C	1.72579842149515	0.76196105681784	0.43529256364355
C	2.52189541755129	-0.38712803091553	0.80376428417510
C	2.13797297048608	-1.76174179769773	0.23614552693253
O	0.53903894796751	0.06559732469994	-1.57350155609319
O	3.52054408506150	-0.34167648035282	1.50362100640468
O	2.83387525256978	-2.34180726026135	-0.56056141705689
O	1.03042984899896	-2.22063940395879	0.79201811281803
C	0.60100794929968	-3.56124536805545	0.41793253916776
H	-0.00984296210191	-3.88382072477721	1.25970999823283
H	1.48488306835978	-4.19523703962053	0.34702843263808
C	-0.18918175845232	-3.54452627899001	-0.87271282087130
H	0.42058154598348	-3.18415247325438	-1.70257796452320
H	-0.51655911810094	-4.56242769408114	-1.09858468905054
H	-1.07084749032657	-2.90910430740577	-0.77741309390392
C	2.06065828188734	2.02646136109469	0.74529216804432
H	2.91131207560688	2.37148297569527	1.31789418749171

N	1.16990503799241	2.94603563144178	0.26469678811451
H	1.52221288297278	3.84760748242186	-0.03714451041739
N	0.38430152936621	2.29076797473427	-0.73253940504847
C	-0.94968879493279	2.88674107612466	-0.88399032293241
H	0.94011794394789	2.30195664245615	-1.70647924996493
H	-0.82465618743396	3.91356124576147	-1.22733015369591
H	-1.48515123738702	2.31131704549945	-1.63796123470897
H	-1.48029562544819	2.86363779237163	0.06529712451633
O	1.69083789023585	1.75352795400521	-2.82975526941614
H	1.24135861554599	1.94924796458597	-3.65815164695805
H	1.14333190666252	0.73520006372985	-2.28561394400669

TS5A-E-2H_2O

Imaginary frequency: - 759.68 cm^{-1}

C	-2.78064899811890	-0.44728656468529	2.64788352317925
C	-1.59485877473712	0.13608902184636	3.06314076988952
C	-0.64538350535212	0.47998341246165	2.11091263759620
C	-0.87666402812473	0.24506161012356	0.76067780671840
C	-2.07694628692839	-0.33640883801553	0.36734668915292
C	-3.03448504319745	-0.68847352664884	1.30646076445549
H	-1.41200810425620	0.31404064793472	4.11568325441472
H	0.28530215115031	0.92534489126188	2.44033901642220
H	-2.26187301392386	-0.52481684648673	-0.68188361176459
H	-3.96660026582457	-1.14803745870336	1.00122036400210
Cl	-3.97177347228420	-0.88954077330441	3.83207367175140
C	0.14963791967601	0.64090871264842	-0.29267798375258
C	1.57505531751014	0.66914670864712	0.23460378781770
C	2.45200460372927	-0.46785436824122	0.41330741985287
C	1.96379766880383	-1.85626907821433	-0.02768292865522
O	-0.05589308038150	-0.05647669842542	-1.44043287447953
O	3.58575123963841	-0.39746699734621	0.85642044692412
O	2.48459036832101	-2.45966517818348	-0.93307074891645
O	0.98920980255256	-2.29168196403334	0.74987074895849
C	0.48699067087529	-3.63649744677650	0.50129925009054
H	0.02776719736608	-3.92111506395720	1.44665780118112
H	1.33940836263411	-4.28613296825979	0.30165380325955

C	-0.51396850940877	-3.64930759058048	-0.63367581687798
H	-0.05262531962787	-3.32911657718096	-1.56897063576889
H	-0.88774602337430	-4.66823943337938	-0.76129720812715
H	-1.35810695345471	-2.99494678736756	-0.41134915067334
C	1.92740272199490	1.93341372807333	0.52974465339483
H	2.86031510972574	2.28701603991317	0.94801802571212
N	0.92661915978455	2.83081867102026	0.28205739425060
H	1.17636384338951	3.76513267225864	-0.02412004569692
N	-0.00172066135153	2.18306976127668	-0.59433295049195
C	-1.35430432823604	2.75378941125100	-0.50160999696094
H	0.39229041921125	2.34866152287242	-1.62797530370177
H	-1.30770202931737	3.78294779411625	-0.85721433210005
H	-2.00341371810365	2.17028379948982	-1.15303762858577
H	-1.70679140637055	2.71994151500523	0.52648171767756
O	1.00210057435980	2.59366368091404	-2.92936430233857
H	1.44151969384040	1.54113076932130	-3.08962629101126
H	1.73054093195931	3.21861941904585	-2.85307435289672
O	1.71868575687912	0.30102841713554	-3.13984425817341
H	2.60333704218180	0.15825559283921	-2.78975785595027
H	0.74482296679048	0.05098636033360	-2.12485526977833

TS5A-E-3H_2O

Imaginary frequency: - 765.52 cm^{-1}

C	1.13867515502771	4.08078872786302	1.25667765145538
C	-0.01269715698930	3.39189049655032	1.60414589089316
C	-0.14856860916274	2.06556741831099	1.21276360066170
C	0.84666680906358	1.42331848298677	0.48396968642542
C	1.99180167970439	2.14260671555810	0.14734928743153
C	2.14756883236724	3.46921719308614	0.52871593876889
H	-0.79443263235821	3.88621051754490	2.16890768263211
H	-1.04848916961427	1.52566486915136	1.47473751357152
H	2.78181500585126	1.67294378777727	-0.42655566353369
H	3.03968670679837	4.02244569642568	0.26016337229093
Cl	1.31859806362794	5.74481372610649	1.73628716813073
C	0.68241545479664	-0.05796254656384	0.08507578177645
C	1.36130661841685	-0.33949788298342	-1.25780513272489

C	0.79686910078321	-0.07541412423158	-2.54868430914101
C	-0.60193007674636	0.54928546725987	-2.58645647015486
O	-0.58514539795744	-0.49508671885152	0.15702679421279
O	1.33782563232970	-0.28926720871422	-3.62912832482177
O	-0.79770549985408	1.71823620951284	-2.35099531379526
O	-1.51405814886525	-0.32074107353840	-2.98840868101650
C	-2.87686383949417	0.17029639272120	-3.08526292895005
H	-2.89840339739978	0.97729024678802	-3.81985016764674
H	-3.16128267606250	0.57336398799482	-2.11135213811388
C	-3.74872850432799	-0.99364762184767	-3.49347543773822
H	-3.70198219322983	-1.79209161712434	-2.74974433075117
H	-4.78424065802024	-0.65544189682951	-3.57443716401751
H	-3.43760493419516	-1.39177227365541	-4.46175264757366
C	2.55983681435742	-0.93121574157744	-1.05478620531533
H	3.27696184229317	-1.26926653862188	-1.79172410438814
N	1.60914032949890	-0.92489183926414	0.98714946585912
C	1.85008450024896	-0.44730149406397	2.34647062628929
H	0.98595211291838	-2.30522924095870	1.05755290255438
N	2.83604848905539	-1.08455239606576	0.26373069201781
H	3.38434013251176	-1.88509067747359	0.55609542300678
H	2.43084400183725	0.47751380893858	2.36435634082970
H	2.38210727984041	-1.22369954280472	2.90050535662145
H	0.88143984879569	-0.28251734035353	2.81948546563696
O	0.62501708464684	-3.30276871641337	1.17110268266806
H	0.24807318850159	-3.55746594126675	0.31864690976115
H	-0.26796213338506	-3.25988635065746	2.01017276284989
O	-1.09075512422026	-3.09397245363631	2.84800694885219
H	-1.47928623016802	-2.12553273723670	2.65116194697923
H	-1.80476232590910	-3.73749751679225	2.75078310026565
O	-1.78522841770119	-0.78415256314668	2.29100062499598
H	-1.25123544389847	-0.61161447297704	1.37098451644139
H	-2.72171211371320	-0.65387521692615	2.10739288580305

TS5A-Z-H_2O

Imaginary frequency: - 1055.13 cm^{-1}

C	-3.17782469517796	-2.48111249121521	0.18495885167475

C	-3.25494605881286	-1.87228400962876	1.43003827322893
C	-2.77508962780798	-0.58110212004759	1.57178176222619
C	-2.19630773502627	0.08372364970739	0.49052870567859
C	-2.12799259633552	-0.54649343462759	-0.74761600202633
C	-2.63314666054884	-1.83007879346391	-0.90825097978529
H	-3.68377207139995	-2.40212035171898	2.27169089403145
H	-2.83462671602794	-0.08618428688860	2.53413401500235
H	-1.67925099373491	-0.03913707907423	-1.59041189361302
H	-2.59090707804263	-2.32339521368093	-1.87150146010051
Cl	-3.76476707286989	-4.10405479862155	-0.00083489098287
C	-1.70707864240897	1.48205168865308	0.72095098031615
C	-0.36274367482380	1.87408425561971	0.28459792251542
C	0.64999810253086	1.06225051068602	-0.51419900904516
C	1.03172649119089	-0.21509518957983	0.28476036728845
O	-2.41140579636986	2.26085490621719	1.35143699892668
O	0.38061598831707	0.81115927045760	-1.81259801924861
O	1.07138191035042	-0.21339950317451	1.49306674931958
O	1.31289237787316	-1.24606114652886	-0.48708906219151
C	1.66504927031038	-2.48738636576475	0.18342319002772
H	2.29415206387383	-2.25131835840592	1.04150111882315
H	2.24995487688439	-3.03057868141927	-0.55740652724481
C	0.42133153451822	-3.25252145441100	0.58409715068044
H	-0.18439117267962	-2.67942295942246	1.28837487589203
H	-0.18629599001445	-3.48788073842455	-0.29141851747149
H	0.71818880352842	-4.18877453644374	1.06311201484004
C	0.22934629522542	2.97214615709135	0.77995589887678
H	-0.21817045800417	3.74513093733168	1.38975690360004
N	1.57528294004858	3.03624853159465	0.52958441256634
H	1.98409625799927	3.93934646860147	0.31279274816513
N	1.87147887301044	2.05050088318403	-0.45975997238705
C	3.20713757181198	1.45765106659424	-0.27761477119153
H	1.78166528915312	2.53096633085154	-1.48987295950692
H	3.34377613409809	0.70253756223940	-1.05139590932329
H	3.94745138276014	2.24670400727954	-0.40307979659758
H	3.28945941031282	1.01969762181779	0.71565001801530
O	1.32847195164245	2.76381468028405	-2.80528704621903

H	0.69431905225549	1.75254460559854	-2.42188874036591
H	0.74094046239016	3.52698837873295	-2.81596829439465

TS5A-Z-2H_2O

Imaginary frequency: - 833.79 cm^{-1}

C	-3.30348302442516	-2.55919086958065	0.64009039042294
C	-3.23351439115811	-2.03303675816109	1.92329199710931
C	-2.77364843789235	-0.73753945387228	2.08944161535840
C	-2.35930544358865	0.01259400833983	0.98989958161774
C	-2.43898429115465	-0.53395130673445	-0.28669581747696
C	-2.92563413908060	-1.82169807802189	-0.46853436099383
H	-3.53371704725448	-2.63103796675213	2.77492865212175
H	-2.71696464487346	-0.30691610224486	3.08234860924256
H	-2.12479699880722	0.04348104590671	-1.14586174751955
H	-2.99683757856112	-2.25109775480290	-1.46018815349290
Cl	-3.86334230715759	-4.18759063065533	0.42331375511361
C	-1.88073751613603	1.41158447141789	1.22593797989508
C	-0.69211966997164	1.90760149348046	0.53060359222096
C	0.30161575787683	1.15516898293216	-0.33393752877829
C	0.74216067618372	-0.18195110270559	0.32043909061368
O	-2.47428200296449	2.12148778295572	2.02885744023082
O	-0.12480425089134	1.05287691679281	-1.61683428126740
O	0.74277550505165	-0.34333450636751	1.51624281159778
O	1.09993941818881	-1.08031526377220	-0.57532830224020
C	1.52762111309205	-2.37833503558001	-0.07531295321172
H	2.15475659313873	-2.21790254698705	0.80169335125204
H	2.13182389560279	-2.78334830985156	-0.88558352012693
C	0.33593184582313	-3.25980785473448	0.23126518729716
H	-0.27772444529476	-2.83078278281016	1.02467494192038
H	-0.28194520129699	-3.39694563624650	-0.65814891708487
H	0.69497062142940	-4.23836946532645	0.55925289548781
C	-0.30084142427795	3.19254434796584	0.57900813715271
H	-0.81156116439791	4.02776664870944	1.03838360762839
N	0.87288455550852	3.41526216729920	-0.08615653859001
H	1.50170792084769	4.14677838935180	0.22083597175663
N	1.50800050902783	2.14864799894332	-0.28581751771225

C	2.54038289206254	1.90927201238101	0.75168521086157
H	2.03213403682866	2.15119749949598	-1.28234933852009
H	3.02678685977185	0.95788257041515	0.54139827491977
H	3.27345667312289	2.71110644970018	0.66710859328780
H	2.08471823407055	1.90406220066929	1.73937119653395
O	2.73962858649781	2.12829553053405	-2.52805925140480
H	3.67128811845453	1.93171534990599	-2.38450330091850
H	2.23347032875320	1.19672693125273	-2.96587564542640
O	1.46157074272163	0.23948219202211	-3.31185676379535
H	0.57177794945783	0.59910518477017	-2.30589182208722
H	1.86484114567192	-0.59148875003474	-3.04313712299555

TS5A-Z-3H_2O

Imaginary frequency: - 719.57 cm^{-1}

C	-3.61026157882613	-2.58678895089636	0.35848211723225
C	-3.88302504279778	-1.90366124248020	1.53565812233237
C	-3.40634217860009	-0.61148985489967	1.68170945811057
C	-2.63868132257438	-0.01784431062078	0.67897807001067
C	-2.37409750175891	-0.72417078854291	-0.48982364882847
C	-2.87307934046208	-2.00934713495604	-0.66035437925450
H	-4.45896292571860	-2.37728858804773	2.32125520065590
H	-3.61857197521663	-0.05856401480144	2.58934522741722
H	-1.75964564668253	-0.27414017601912	-1.25839064647163
H	-2.67587774075393	-2.56227271840009	-1.57073959866004
Cl	-4.19434577521195	-4.21180603373938	0.16792744810156
C	-2.16770108780071	1.38939168814821	0.90895430311928
C	-0.78337005172367	1.75899674300152	0.63993936988537
C	0.33953142347981	0.92755725891598	0.00891674549056
C	0.59284720912922	-0.30138820011277	0.94424938992617
O	-2.95741582843083	2.19162987929762	1.40567222751189
O	0.20874901274016	0.61852306203136	-1.28188255976827
O	0.48326084870255	-0.24056139973904	2.14987294110155
O	0.94693434645105	-1.39709469938202	0.28732837836897
C	1.19244885033391	-2.58647651469681	1.07680741866032
H	1.70233557271805	-2.29875188756868	1.99633812790678
H	1.86624215318736	-3.18289924552325	0.46214049607974

C	-0.10072740170457	-3.32442956096930	1.35781278126942
H	-0.79404782980762	-2.69877380607662	1.92289318056194
H	-0.58325595057748	-3.62692980290528	0.42656474207438
H	0.11629525849813	-4.22113314766480	1.94377312959714
C	-0.25700487013126	2.87594012313776	1.18207831317383
H	-0.78116463829718	3.67479472821701	1.68953250305404
N	1.10088392190367	2.91807886492626	1.11510387659383
H	1.54441521030928	3.81596757255580	0.95476386417634
N	1.52097815049403	1.90680801317568	0.19585464626595
C	2.80609558859963	1.33068159495617	0.59124977401737
H	1.68828450411337	2.61504533981655	-1.15139599713275
H	3.05551181753816	0.54326374144159	-0.12144719140547
H	3.57127768540057	2.10672631685428	0.53309596036852
H	2.78099771892973	0.92523238415195	1.60603538767209
O	1.87651525392019	3.18174320023635	-2.02814110775475
H	1.01435679272334	3.45837677868225	-2.36584873309939
H	2.39949698857688	2.44174059045919	-2.83798138626399
O	2.03427975795332	-0.45030767938632	-2.57619033645858
H	1.25439068134523	-0.05837091353438	-1.96800460807522
H	1.63771058370578	-1.09164582826470	-3.17489759275381
O	2.93872847651198	1.64520484422348	-3.54828563895982
H	2.57027765617326	0.70737357250101	-3.23168939167158
H	2.67473321363766	1.78306022249769	-4.46726036417773

TS5B-E-2H_2O

Imaginary frequency: - 516.63 cm^{-1}

C	-0.56070854337978	3.31804678802016	1.44508918912569
C	-1.56061327090357	2.37305291865694	1.28532942883304
C	-1.22222026200363	1.09680295132671	0.85628150593976
C	0.10062014484046	0.76975160804848	0.58924572640898
C	1.08844304424719	1.73929862756520	0.74883727854534
C	0.76691254993283	3.01563466228505	1.18274147821484
H	-2.59279344899789	2.63081625350377	1.48857715100765
H	-1.99917657566991	0.35626128713782	0.72607863295098
H	2.12443716767538	1.51224230128670	0.51797216316402
H	1.53484389009432	3.76932764949736	1.30476277751477

Cl	-0.97804000884872	4.91715874637622	1.97626866964331
C	0.46890508110174	-0.61862945326842	0.07774797273454
C	1.37039569831179	-0.63072796918943	-1.14836852989863
C	1.18924751985522	0.07530471757995	-2.38714007291471
C	0.08109273790479	1.13604958332577	-2.47511321525316
O	-0.57751131526007	-1.46783082597228	-0.10988205926678
O	1.89420457375011	-0.06306964688744	-3.37794164355159
O	0.32688222315119	2.31099749429792	-2.60534689183450
O	-1.12295259532972	0.59584003553796	-2.43996730422417
C	-2.25061629061142	1.51035353130555	-2.51840416011642
H	-2.21397179932878	2.01050419213042	-3.48772362537822
H	-2.13402480646799	2.25619188176724	-1.73062894847760
C	-3.50681886004171	0.69124870920510	-2.34320265728848
H	-3.51193346717149	0.19405676541023	-1.37089784324476
H	-4.37494758267098	1.35168744179643	-2.40000549060570
H	-3.59260654470999	-0.06298005098991	-3.12826148453240
C	2.39035111885765	-1.49535473620064	-0.92279296015500
H	3.15839081862608	-1.81216708377691	-1.61604305216139
N	1.45766458931193	-1.25588554999538	1.10198679307148
H	1.96464085038913	-0.52325150156447	1.60763173881266
H	0.93046172199195	-1.90772657655817	1.85005504837493
N	2.38516340417439	-2.00327553565332	0.32487525857095
C	3.51510225607962	-2.59462957319576	1.02275488287410
H	3.14945167198852	-3.31057362626467	1.75846944677325
H	4.12251566713297	-3.11366895228272	0.28333432494764
H	4.11230576322035	-1.82245270665181	1.51740250515680
O	0.19592515377015	-2.66003695771775	2.84458443460530
H	0.33595838051945	-3.60475251693110	2.71814591696469
H	-0.82765398553871	-2.42273268585436	2.48602801533244
O	-1.93248461883662	-1.95209111392241	1.90675713917720
H	-1.19189543378254	-1.62137683441301	0.75311940030059
H	-2.51294661737373	-2.68741424877104	1.68764305985858

TS5B-Z-2H_2O

Imaginary frequency: - 687.18 cm^{-1}

C	-5.03556216876679	-1.83737136597787	0.31889031506497

C	-4.17956883344643	-1.26381642638382	1.24520016132872
C	-2.87926847324233	-0.96321173641530	0.86259072665824
C	-2.44077013534128	-1.24866637604982	-0.42756068731299
C	-3.31304997157482	-1.84635424174628	-1.33529857489415
C	-4.61894859236334	-2.13272386902962	-0.97201596008023
H	-4.52242018901840	-1.05776126456095	2.25162103934694
H	-2.20051498262808	-0.53032678736861	1.58781486077640
H	-2.96683823098266	-2.07865226954243	-2.33583900854902
H	-5.30684969032050	-2.58235709781003	-1.67742919828316
Cl	-6.66691005589388	-2.20296614455581	0.78549922108207
C	-1.03122567252117	-0.98452163319383	-0.85835992023245
C	-0.41292233281612	0.27235518136558	-0.49825994391318
C	1.03214328017293	0.59859859504713	-0.83093992629559
C	2.03001827550012	-0.48817549981763	-0.36065644692478
O	-0.41312154660321	-1.83276182805327	-1.49497855634457
O	1.18082262472828	0.92858157148443	-2.13196105402413
O	2.81987792693654	-1.02380157472558	-1.09176598737574
O	1.87689719099264	-0.73794644448202	0.93397205027851
C	2.69252078944656	-1.79322940630788	1.51277544490431
H	2.72787761775749	-1.54973580453147	2.57342863097414
H	3.69490728190080	-1.72638278729689	1.09023367120634
C	2.05792616699513	-3.14531322341105	1.26654191282142
H	1.04699481268993	-3.17526325548410	1.67885723522368
H	2.01343278927953	-3.36879733655932	0.19919621756841
H	2.65639367741813	-3.91636587922497	1.75763161127194
C	-0.97823765079129	1.38475799533751	0.01580652210408
H	-2.01767226648736	1.57238657049064	0.24830810047693
N	1.21914263565957	1.83065901715871	0.07897315320780
H	1.97070277337617	2.56620113527283	-0.35112285475227
H	1.52803868588072	1.52670876363518	1.00917854980180
N	-0.08526002806501	2.39633295904709	0.19279179906484
C	-0.23593436503352	3.46778880249438	1.16505195609144
H	-1.27709440511740	3.78583461830735	1.14465436840182
H	0.02657448887760	3.12668037738290	2.17190527294491
H	0.40174638686837	4.30303451695136	0.87714863266328
O	2.93186204806564	3.39854464849938	-0.97659722675269

H	3.66950645896493	3.56685263868803	-0.38038344953063
H	3.27374331959381	2.68508067895183	-1.76391382901503
O	3.42466462490213	1.74966449728282	-2.68880754563850
H	4.10167402529760	1.12692635251319	-2.40716658108811
H	2.20470170970900	1.19951333261820	-2.38501470225578

S132 CPCM(Acetonitrile)

BED-E

C	-7.61250272738492	12.65743822829108	3.86156959842175
C	-8.17083919289941	11.83814693520309	2.88688923528770
C	-7.32976075748980	11.09453776089221	2.07924571512457
C	-5.94544765142739	11.18164232836750	2.22591819528574
C	-5.41000280794916	12.00761585851510	3.20879236760110
C	-6.24245904514612	12.74675392209619	4.03949893747492
H	-9.24615043757763	11.79006323047702	2.76700242733572
H	-7.74251229864470	10.44869791926661	1.31344479567083
H	-4.33596857577889	12.06913870709250	3.34132218331546
H	-5.83186368912231	13.38512990904838	4.81180875354645
Cl	-8.66207734457340	13.59025632780000	4.88030826284239
C	-5.06442335323314	10.34377644493905	1.35169374174457
C	-3.71742970685569	10.83608961624762	1.02366956443862
C	-3.48595198832790	12.20365522699363	0.63341139129211
C	-4.68677547335272	13.14375047346611	0.41538452544732
O	-5.47764050868576	9.26678420644985	0.93733770615580
O	-2.38650971388401	12.69783626723699	0.39790655792872
O	-4.74368322360033	14.25669415172837	0.87663496127017
O	-5.59068559553245	12.59848568962716	-0.38660795495651
C	-6.78553944455054	13.38183283668449	-0.64475952735764
H	-6.48633847754293	14.31249618871085	-1.12959191225150
H	-7.24476467678519	13.61649469989182	0.31763899693067
C	-7.69200493838523	12.54706774099727	-1.51763421352462
H	-7.96488289193649	11.61712343546246	-1.01436758992296
H	-8.60494107617445	13.10895543234484	-1.72716764924653
H	-7.20639260765520	12.30805307098836	-2.46614726297900
C	-2.61532824866149	9.99150779921800	0.84445461381253
H	-1.79703546665616	10.38895218946418	0.25068631734203

N	-2.39999509514081	8.79358786964172	1.33004806117760
C	-3.23642109585608	8.16207254332883	2.34078389714474
H	-2.60190168627213	7.50743025334325	2.93762800089906
H	-3.67060742567161	8.92571286124449	2.98545978930322
H	-4.03846651858927	7.58246546538508	1.88238466987882
C	-1.23839615602071	8.01904378271926	0.89772020116866
H	-0.62071535892869	7.77523514953708	1.76400648808035
H	-1.57556993356633	7.09260347789931	0.42791729082886
H	-0.65946558014064	8.59624878939961	0.17946693348819

BED Z

C	-2.46519050109774	-2.09995560594205	6.58609115849708
C	-2.83538537553292	-1.93583094912970	5.25692984941850
C	-2.16018399011882	-1.00644043716796	4.48533985423361
C	-1.10877685374969	-0.26701837517734	5.02469664224862
C	-0.75616237582532	-0.44910211007306	6.35802928179192
C	-1.44104503339825	-1.36122533725410	7.15101576989956
H	-3.63837479886457	-2.53008190905762	4.83847643365888
H	-2.43915725613255	-0.85807494073303	3.44867108243042
H	0.04245502667710	0.13723250342576	6.79814151949822
H	-1.17519686912538	-1.50050107362324	8.19148593520319
Cl	-3.29710610597994	-3.27447987652718	7.55526811279896
C	-0.43971036167103	0.77624921121460	4.18007124653475
C	0.99841036539824	0.99083458792178	4.30266093156110
C	1.87916752360787	-0.00819626443131	4.86598149998003
C	1.54745765970856	-1.48839829228194	4.56512099663215
O	-1.14114843270222	1.47742879173298	3.45138532302493
O	2.90660735924269	0.21923561440971	5.48871388212306
O	1.10685159827813	-1.84020879335906	3.49606624581235
O	1.89779204198705	-2.28796072730109	5.55648288115108
C	1.74347196418614	-3.71642642653213	5.33848303277009
H	2.46050834846019	-4.16730856345223	6.02328330295557
H	2.03271483967928	-3.94083970332541	4.31178182636150
C	0.32874053476160	-4.16418289514719	5.63733802565822
H	0.25860587990337	-5.24411763306730	5.48525607574210
H	0.06224660507797	-3.94190005043078	6.67215125434159

H	-0.38651886412238	-3.67291617069331	4.97602805644708
C	1.41493808297684	2.29195284436523	3.98687993619086
H	0.63986817326771	3.05277825956023	3.96144285892803
N	2.60823249983031	2.73028037072992	3.66926211991002
C	3.73661615221863	1.86644144411384	3.35374920116062
H	4.34852932861509	2.37827329390030	2.61131508965685
H	4.33261805691142	1.65397782316771	4.24161999016298
H	3.37047768243677	0.92950693458746	2.93559829068775
C	2.85460904633851	4.16508145518130	3.53553797549919
H	3.20831699848581	4.38042905245288	2.52573018897803
H	1.93609917521225	4.71426126771718	3.73309127853980
H	3.61872187505928	4.46940268022621	4.25382284951106

Methylhydrazine

N	-2.01641615034142	0.00446653606146	-0.02582994587342
H	-1.61129274371865	-0.92966598777202	-0.02949454745235
H	-1.57415632214019	0.46246466019677	-0.81929607278954
N	-1.45364845988793	0.65520131686687	1.15604256235731
H	-1.86002609354511	1.58564682472164	1.14447745137221
C	-1.97896718252236	-0.02176320942402	2.33808431457291
H	-1.54506095282085	-1.02362039476664	2.40689178288148
H	-1.68139526400595	0.54117906767702	3.22511379320795
H	-3.07347487101750	-0.11897693356107	2.32073420172345

$HNMe_2$

N	-0.31220741451257	0.19651010008168	-0.08045038518711
C	-0.23034410619959	1.63875599958080	0.12549516284992
C	0.84032570019050	-0.30882003562605	-0.81885034964294
H	-1.15239088741655	-0.00946790094600	-0.60831909741409
H	0.61877940071128	1.86556874939064	0.77725881811996
H	-1.13914987813660	1.99638845214949	0.61470020146682
H	-0.09352825375987	2.20121788160216	-0.81164472965953
H	1.74365901513420	-0.18210205241832	-0.21453365262306
H	1.00149428862947	0.21178942287150	-1.77610384180719
H	0.71366213535972	-1.37474061668590	-1.02115212610276

GS1A

C	-2.67043852333121	-2.59356578117397	-0.19284829353979
C	-2.67703503423785	-2.10321915439982	1.10773283119245
C	-1.97373376278084	-0.94469011675653	1.38825559992926
C	-1.24345630964324	-0.30008331880406	0.39117261317387
C	-1.26123650878734	-0.80274893766581	-0.90412236098425
C	-1.98339282280173	-1.95103565335422	-1.20697573305679
H	-3.22532214696991	-2.62462528837657	1.88279625349803
H	-1.97223909948721	-0.54415576581099	2.39550310280945
H	-0.71075645518671	-0.29433162452955	-1.68694478108284
H	-2.00305700129713	-2.34439451377145	-2.21576849090064
Cl	-3.54448108769545	-4.05119012067584	-0.55199153879768
C	-0.52789024113256	0.97974130847934	0.70504169425119
C	0.81399594036377	1.18882396799631	0.28624281458760
C	1.73421640160686	0.15727304956198	-0.02186879055923
C	1.43129463373586	-1.30633857777989	0.31975898933457
O	-1.20391934855828	1.85746023804615	1.29747458985851
O	2.88901747117239	0.32967566124383	-0.44680961373762
O	1.30296858290669	-1.70393222607689	1.45506396113655
O	1.46133085988109	-2.08252819470388	-0.75474170440200
C	1.20830815013537	-3.49258783046924	-0.53706036128024
H	0.27633480982406	-3.58864131475071	0.02328502174029
H	2.02317929807046	-3.89948333605648	0.06479216727458
C	1.11942283416251	-4.15031201308825	-1.89408636765957
H	0.30215643004973	-3.72124740999884	-2.47781525597430
H	2.05271738113338	-4.02835026604307	-2.44814483658431
H	0.92968086314540	-5.21830337157615	-1.76508767705104
C	1.38243011260286	2.58715331196878	0.21578205117148
H	2.46683448082074	2.51037323188188	0.23206942645314
N	1.02975518814778	3.43305426292868	-0.89907165779323
C	-0.36575242028430	3.38097637747447	-1.31786694948296
H	-1.02712283942383	3.63116560645934	-0.48830210787895
H	-0.51421756680284	4.11703545492980	-2.10983163275001
H	-0.65090095457378	2.39165376637609	-1.70358762688531
C	1.92832562074524	3.23910932564017	-2.03223781846743
H	1.80900837603886	2.24894404692718	-2.49585290600043

H	1.71554330573820	4.00024887087194	-2.78540476459218
H	2.96472214103713	3.34876323556482	-1.70799380565514
N	0.98800783929877	3.31424705645872	1.50133908766776
H	-0.02543695910031	3.11097997923562	1.63236395630960
H	1.46345850024977	2.85946735089015	2.28247949454550
N	1.23941903060829	4.71752817839990	1.57982016760867
H	0.81008553367754	5.09849967169520	0.74047887715037
C	2.67776175444137	4.99688280280098	1.53802967498368
H	3.16265488402648	4.65537393204861	0.61827578806454
H	2.79512319094152	6.07739576547959	1.61901277662320
H	3.16013852753239	4.52953977250266	2.39912864575162

GS1B

C	-2.67762181508522	-2.44488226797887	-0.01423851640126
C	-2.62289076481641	-1.89967581288712	1.26318857697504
C	-1.88369402721173	-0.74748254787750	1.46631335398947
C	-1.17900178818384	-0.16327457833680	0.41500079580079
C	-1.25719186993046	-0.72053814520489	-0.85554509230395
C	-2.01566867389265	-1.86322166024581	-1.08055128232292
H	-3.15220776571894	-2.37423620836308	2.08038473305313
H	-1.83395689250089	-0.30443089239001	2.45429937002797
H	-0.72608602421903	-0.25924619164709	-1.67986730637110
H	-2.08286898610493	-2.29890849974242	-2.06969381021171
Cl	-3.59807813729651	-3.89437672430497	-0.27638398759181
C	-0.42249435848372	1.10931531224565	0.64720626225907
C	0.90033311898352	1.27758009754388	0.15550823689652
C	1.79531097623577	0.21877198866356	-0.13046830101383
C	1.47719274840085	-1.22061225232949	0.29020778696939
O	-1.04223566837864	2.02125324982981	1.24977422989671
O	2.94082056917103	0.35229705635344	-0.59213425861011
O	1.38661109363159	-1.56041375064735	1.44782893200384
O	1.44635879377990	-2.04705628372926	-0.74586638492929
C	1.17149664219057	-3.43896112649111	-0.45046651431453
H	0.25764418580645	-3.48606560325562	0.14496578478356
H	1.99796654110636	-3.83482457332553	0.14276618215428
C	1.02091739512923	-4.15949025797150	-1.76960720714133

H	1.93666945316627	-4.08646787232760	-2.36030374852093
H	0.81270093553139	-5.21519518004100	-1.58148583180550
H	0.19315862531032	-3.74037139468355	-2.34571446418111
C	1.47667510528475	2.66795682154061	0.00026493282745
H	2.56397635637027	2.59422237112657	-0.02475906597293
N	1.08072273495736	3.43824435685398	-1.15647154140885
C	-0.34710813951602	3.44882225513737	-1.44739471661749
H	-0.51985563305278	4.11976298467071	-2.29081385036896
H	-0.73106430507702	2.45301170574212	-1.71252674389994
H	-0.91442733379920	3.81989547605589	-0.59334119727666
C	1.84843071268913	3.04754052381179	-2.33453411242967
H	2.91678600876025	3.11250595126904	-2.12211411330754
H	1.61896995534246	2.02285359394823	-2.66294801078962
H	1.61028139631776	3.73239989662391	-3.15070227084169
N	1.81775487560148	2.88839115519046	2.42132062739582
H	2.81937422733168	2.93413891718477	2.24548028857364
H	1.56909313979861	1.90108344812503	2.42850979699845
N	1.17193454555826	3.45723672337278	1.27368649931224
H	0.15667393642275	3.29783735551747	1.43630612517473
C	1.49182187968925	4.89992805412479	1.19853789395608
H	2.54828199314879	5.00632522717444	0.95233862565824
H	0.88141844515175	5.35719844700935	0.42739519423283
H	1.27909705240018	5.33000279466494	2.17463789969350

GS2A

C	-3.72014530404718	1.52564077006106	-0.76917088153582
C	-2.49615747460872	1.93230372834750	-1.26835625807026
C	-1.41614274355740	1.06151042173811	-1.18868235193312
C	-1.56713568185290	-0.19680473406185	-0.61872718088657
C	-2.81918709601675	-0.59967693923380	-0.15380491244426
C	-3.89844749694752	0.26331544507589	-0.21459180757931
H	-2.38295728403478	2.91535813120135	-1.70787679498727
H	-0.45208948136560	1.36774313327860	-1.57778014488424
H	-2.94109767791460	-1.58546053051683	0.27983795847513
H	-4.86859067908349	-0.03212828150837	0.16541654130169
Cl	-5.06740051445529	2.61753824110710	-0.83505830339651

C	-0.42272999960242	-1.13735358583182	-0.54554183669503
C	0.78852919022164	-0.85252845383737	0.01048327720528
C	1.04423681553258	0.36723021663057	0.75832407729689
C	-0.10103183559301	1.08312266237249	1.49390440330388
O	-0.68088950912750	-2.30242069710288	-1.12893659429763
O	2.16348373330472	0.80355055465396	0.98416750928807
O	-0.80027524847451	0.51339223219342	2.29752458990903
O	-0.13297936449416	2.37695501173193	1.22731041485862
C	-1.17721800227918	3.14221814142877	1.88389416104971
H	-0.97808620255478	3.13990995040326	2.95704399945358
H	-2.12954169049411	2.64093099226993	1.70099020967375
C	-1.15132342072359	4.53480119313921	1.30033467255981
H	-1.34605726215422	4.50668433916997	0.22608493157720
H	-1.92711485896262	5.13781131473364	1.77747591907060
H	-0.18473800556231	5.01259248499238	1.47377523274224
C	1.97544752656648	-1.79871497365471	-0.05901394626499
H	2.86636998747592	-1.19475909294817	-0.24683386135174
N	2.18126603510567	-2.45719050275133	1.21245317931765
C	1.10399270170580	-3.34212463517876	1.63556311110981
H	1.27713065778545	-3.63744629334496	2.67278414404899
H	0.14910246881254	-2.81658347598038	1.58629387383036
H	1.03328412864720	-4.25847938671505	1.03018760520486
C	3.49085022646042	-3.08103762039301	1.34030816358609
H	3.65897853620382	-3.34741990109564	2.38636289898460
H	3.60235782701143	-3.99972138157653	0.74256957338505
H	4.26454392007426	-2.37389599048542	1.03351416870078
N	1.81105281527355	-2.76211009522785	-1.17774848670159
H	2.34260369410629	-3.60740736142198	-0.98125994276070
H	0.22527619754978	-2.78148153861227	-1.19213535291156
N	2.21866531540901	-2.18266462736180	-2.41200386132229
C	3.64671547180829	-2.36309318691865	-2.66750425917312
H	1.68386199762534	-2.63920764082530	-3.14106515423923
H	3.95115263226076	-3.41801782905385	-2.62179750658314
H	3.87268347002430	-1.97186197934376	-3.66094979647711
H	4.22925148494116	-1.79991822954651	-1.93536538143811

GS2A

C	-3.39659208022438	1.47667957524043	-0.45858261952265
C	-2.22400984756896	1.53932875990728	-1.18976017203439
C	-1.24101030744118	0.58423653274529	-0.96272137936208
C	-1.43685214778997	-0.41717200137129	-0.01874855860091
C	-2.64233510377751	-0.48395206825573	0.67979521773049
C	-3.62239523082804	0.47038582456953	0.47388590115177
H	-2.07585350188202	2.32350003800032	-1.92160126562520
H	-0.31852176951140	0.61878443165284	-1.53061899092985
H	-2.80093778530817	-1.27301616355138	1.40560448270731
H	-4.55177317089564	0.44192067115999	1.02914640928498
Cl	-4.61752682977558	2.68340042616245	-0.71139171817098
C	-0.39074075853439	-1.44108556471653	0.21342304989891
C	0.92851181778512	-1.15949744180138	0.46302554332346
C	1.42378905502475	0.16428910025033	0.74931809764429
C	0.50051828642468	1.23146741819059	1.35992882256518
O	-0.83010913193386	-2.67751188181675	0.11125805428797
O	2.61014342664759	0.46976153414638	0.68057858905574
O	-0.11581449202001	1.04291854664670	2.38180563133320
O	0.56149650248742	2.37654350690801	0.70185492564004
C	-0.27379817565760	3.45108351070947	1.20599588473324
H	0.09257503248894	3.73279115836692	2.19481292537211
H	-1.29230065489312	3.07034407682745	1.30311726512512
C	-0.18859878666084	4.59072183217891	0.21891039842967
H	-0.55498050474691	4.28043845545038	-0.76211686232006
H	-0.80643017230753	5.41875522537721	0.57355619673390
H	0.84001749387650	4.94367579372904	0.11859696121790
C	1.99815072855649	-2.24446772201974	0.38128738838028
H	2.82301387012712	-1.97551762890273	1.05141283989667
N	1.41331414126351	-3.53398104441200	0.82108154014218
C	2.15188771147397	-4.71309643375229	0.36737833573081
H	1.66785223921149	-5.59535918004658	0.78929210329118
H	2.12888895415859	-4.77724151735685	-0.71762740385710
H	3.19588885747838	-4.68570524360769	0.70272834719111
C	1.28632647154543	-3.53768627818107	2.28370995306013
H	0.74619685776013	-4.43272634114986	2.59431400037381

H	2.27270068341447	-3.52698024767346	2.76413285062235
H	0.72777567185089	-2.65843724963223	2.60843127976021
N	2.60909888036071	-2.35120440510880	-0.93123061112221
H	3.18364681201466	-1.52213313123966	-1.06811171889707
H	0.01625158518576	-3.27931097984606	0.33262335566931
N	1.64615024759508	-2.47272509802489	-1.97144553406084
H	1.21330968219772	-1.56765746676846	-2.14158292054055
C	2.29908430016887	-2.92619246360734	-3.19235807330755
H	2.67830165350115	-3.94000410706160	-3.04656484507065
H	3.13694148505710	-2.28175085657593	-3.49467422101202
H	1.56134800410057	-2.93841390173922	-3.99766945591918

GS2B’

C	-2.63919944021530	-2.41541380742590	0.02081258540966
C	-2.62237887413082	-1.80529859573797	-1.22860998451525
C	-1.88793457235466	-0.64585116301882	-1.39736893106064
C	-1.15453638818031	-0.11591271897670	-0.33491262463613
C	-1.20235943914983	-0.73070478593711	0.91140574248615
C	-1.94899316636767	-1.88703696656066	1.09702890240206
H	-3.17508653935627	-2.23839444117557	-2.05306013061119
H	-1.86041407569540	-0.16032193671784	-2.36567841017115
H	-0.66127718214992	-0.30164081034740	1.74648888635686
H	-1.98889417793222	-2.37107192021224	2.06472882891857
Cl	-3.55015038136628	-3.87595769893584	0.23406120143666
C	-0.36801890408210	1.12161727198056	-0.52676340256311
C	0.92134230895789	1.30621094013643	-0.10283935149154
C	1.79270083008678	0.20607710874714	0.24684870750573
C	1.49577198008601	-1.21412473785282	-0.26247774468503
O	-1.05433023895981	2.08282025048368	-1.12707939575383
O	2.87988008486808	0.33854351305391	0.79512539959111
O	1.39476076237554	-1.46822019317716	-1.43947085932297
O	1.48894158099179	-2.10108176616491	0.71739672544391
C	1.22586448279670	-3.47718947017839	0.33703230784929
H	2.05208670493637	-3.82302883626860	-0.28662067550530
H	0.30730110894352	-3.49479733153336	-0.25262704572867
C	1.09493086858044	-4.27988349637859	1.60935971728772

H	2.01503608120497	-4.23389692767227	2.19585029168473
H	0.26713428844246	-3.90802410834072	2.21703477583957
H	0.89703057597442	-5.32370400970695	1.35552624649748
C	1.53332193161554	2.70483544874924	-0.03309583391319
H	2.62465043318961	2.60598129021975	-0.04328585117531
N	1.20681601378646	3.45185099400365	1.16961895252356
C	-0.21024091310682	3.55177756503317	1.48574464223605
H	-0.32389534012682	4.20182041800108	2.35536006368845
H	-0.76304379375923	3.99708428100055	0.65747925445834
H	-0.66461115486190	2.57835783184223	1.72620767182247
C	1.96800039390940	3.00471573969375	2.32791142316525
H	1.80587143172857	3.70873988319680	3.14737113617964
H	1.67100139865068	2.00244119899696	2.67188091710260
H	3.03213437988586	2.98326291137537	2.08877089058141
N	1.44031863285978	4.85095543813105	-1.19025168123968
H	2.42767337290388	4.93653255288008	-0.94072546095333
H	0.92766454292839	5.25399501010699	-0.41422771113062
N	1.10613987072449	3.46765552955178	-1.24054338822586
H	-0.39965691505126	2.85331945062692	-1.28020049069824
C	1.72163139462784	2.90477135562054	-2.44520043424427
H	1.33766461560611	3.43799010545187	-3.31385617569217
H	2.81419266496199	3.00825116735427	-2.40934684070348
H	1.47257697122295	1.84729622608191	-2.53081520644634

GS2B’

C	-3.57926438860923	-0.78283223973921	-0.83850422481617
C	-2.93881782524624	-1.75159905124963	-0.07450456842171
C	-1.55679272465651	-1.75768490176922	-0.02276916883918
C	-0.82070918456447	-0.79059585312685	-0.70823694645660
C	-1.48009956770749	0.15899704885551	-1.47842478959422
C	-2.86840211814763	0.16730006585111	-1.54879465550778
H	-3.51838233277133	-2.48571118385712	0.47145014937937
H	-1.04316907479085	-2.50472448948094	0.57136622351231
H	-0.91255954514503	0.89618654797083	-2.03490140660611
H	-3.38724880795400	0.90589710540239	-2.14703995809542
Cl	-5.31396804174712	-0.77020463986806	-0.90251389506799

C	0.66159175202912	-0.82718811145048	-0.64444807731260
C	1.46237856234957	0.22244360603948	-0.29808824346915
C	0.93457723669607	1.51341703252428	0.11105540132313
C	-0.41103126900611	1.63841398227848	0.86074325104909
O	1.15781623197029	-1.99024376397525	-1.03838498331539
O	1.54423147739268	2.56366631373951	-0.02197575615645
O	-1.20191321618996	2.51904304195789	0.63138550159301
O	-0.51562944562888	0.73270275876289	1.82086714539728
C	-1.73037611502479	0.74574078409507	2.61726306238173
H	-1.82303485083333	-0.27940984019888	2.97338759745788
H	-2.56846775414264	0.97609287165569	1.95915801228212
C	-1.61593276171026	1.72884864814407	3.76272885894830
H	-0.75455751779028	1.48847420644010	4.38979015590480
H	-1.51421224984515	2.75084059896995	3.39389538950791
H	-2.51793130616261	1.66959070336047	4.37666255032126
C	2.98774705543820	0.10622897413552	-0.31922867672639
H	3.40680156518249	1.09368117397017	-0.54231881636385
N	3.40553706926122	-0.85295264150799	-1.35989848850475
C	3.17876299647006	-0.27128238080211	-2.68707639736421
H	2.13959241811306	0.04359476387077	-2.78859325712417
H	3.82655228262943	0.59990316392543	-2.85275835104262
H	3.39292539318951	-1.02273279964395	-3.44788630956639
C	4.79824333184784	-1.28374678363148	-1.23488179481870
H	4.94160852945761	-1.84338681195617	-0.31443245370891
H	5.02963308769072	-1.92747578335349	-2.08520957042921
H	5.48553586177140	-0.42717172982978	-1.24333784188476
N	2.98233862864978	-1.49841232988380	1.42674029933721
H	2.09819303083202	-1.32227272108366	1.90653694702523
H	3.62106852481537	-1.88419803529901	2.11328022854279
N	3.54147452371002	-0.27303205774787	0.97364487096552
H	2.17577929226409	-1.83460393229457	-1.13468449778172
C	3.44252996241327	0.78095599023941	1.97471697478573
H	4.02000985593632	0.48067412047958	2.85146723041875
H	3.85717882252407	1.70921512895394	1.57828291018904
H	2.40529987503968	0.96186998012695	2.29535019865197

GS3A

C	-3.66745608188729	-0.86285577051353	0.69734122020316
C	-2.81079799033398	-1.58354726072878	-0.11640930834665
C	-1.44425114114294	-1.33438123679886	-0.04085336492815
C	-0.94614822668766	-0.38727090423940	0.84607706984304
C	-1.82789672272163	0.30621227470151	1.67582767198903
C	-3.19205209540628	0.08527921467794	1.59700578889768
H	-3.20217921643626	-2.32497751922746	-0.80216862977157
H	-0.76352729772923	-1.89020599224140	-0.67552720294356
H	-1.43899963338964	1.03597926811981	2.37668164784810
H	-3.88327399573900	0.63738278092306	2.22222052644457
Cl	-5.37900611046281	-1.14741711213355	0.59538996330388
C	0.53081220699233	-0.17190474138123	1.00127214435850
C	1.38309243012991	0.01123423480808	-0.12249310893590
C	0.99150461205901	0.42137651537821	-1.41820427016137
C	-0.43697774833570	0.89778903845884	-1.72010451465784
O	0.97440452229952	-0.21683478645325	2.17394008202345
O	1.75099919254629	0.51763623226484	-2.39718991307319
O	-1.11909502134307	0.43239022940229	-2.60198878719724
O	-0.77069744781013	1.94822239927942	-0.97927604425468
C	-2.10149941248229	2.48596950450704	-1.18557108876523
H	-2.81833937388715	1.67918358232479	-1.02046913892676
H	-2.18180620282772	2.82136723597904	-2.22114209793868
C	-2.28918443211748	3.61878529527265	-0.20425970520117
H	-1.55208331059030	4.40675303962921	-0.37402567992532
H	-3.28706615226840	4.04428321022142	-0.33281812618805
H	-2.19507558242269	3.26045559052204	0.82294986286682
C	2.86195745476876	-0.18711472856591	0.07702654411289
H	3.41011119024614	0.18100419179445	-0.79099243741217
N	3.33496072578876	0.68489239276222	1.24350972122685
C	4.74343493486546	0.43683321603278	1.63161272412632
H	4.97460802027381	1.06885797754688	2.48770144101155
H	4.86158919305766	-0.61011420165838	1.89181605999070
H	5.38572695694613	0.69104250613887	0.78919651208746
C	3.11262861066499	2.12405146766445	0.96021894397743
H	2.05712947638727	2.28772480079014	0.75827102749577

H	3.42478852827494	2.69957310137044	1.82953521259075
H	3.70956079285195	2.40257059796349	0.09187338094040
N	3.20296659959211	-1.56041398084586	0.35890469800368
H	2.40736844070796	-2.15483106918829	0.14737690893170
H	2.69558122430539	0.40231895870925	2.00559321048925
N	4.37012646986608	-1.99657005973425	-0.31871008217192
H	4.80238408341109	-2.70531192147088	0.26132473858404
C	4.07196867470275	-2.54944694108119	-1.63827544237744
H	3.70336468668130	-1.75466097644230	-2.29110922463751
H	3.31556025977583	-3.34659976255722	-1.59829673668642
H	4.99088748882616	-2.95481037198141	-2.06691174684620

GS3B

C	-3.33695905377083	1.26707193399593	-0.84853205937517
C	-2.88045872639009	1.99609420195625	0.24463260162093
C	-1.53837574778516	1.93074099271684	0.57787919453484
C	-0.66479522276670	1.12261414001754	-0.14960956308698
C	-1.14223011864235	0.41250320633258	-1.24536560950512
C	-2.48311960431798	0.48536475952065	-1.60702461981268
H	-3.56956732783598	2.60181736033656	0.82059071638105
H	-1.16358843480940	2.49516287514700	1.42407172149745
H	-0.46363096890448	-0.20311401817629	-1.82452397065774
H	-2.85836030296898	-0.06429356611847	-2.46159819828627
Cl	-5.02137277597966	1.34151217353701	-1.27037433576392
C	0.79434162266211	1.10017878486279	0.20359573796179
C	1.50278266080614	-0.13486198074078	0.27076749189259
C	0.90059549712705	-1.37885323007721	0.56712822762535
C	-0.55956019515626	-1.46802119629007	1.02563050404587
O	1.33601334437711	2.21598763917981	0.38183869934966
O	1.49573208276331	-2.46976865628317	0.63491601392233
O	-0.94777360186080	-1.00072096071036	2.07177121936284
O	-1.28835624413170	-2.21898020778753	0.20884479321134
C	-2.68158706045574	-2.41373298715706	0.55876426195751
H	-3.17097240776520	-2.61011551093829	-0.39477358965506
H	-3.06805315101474	-1.48236059978485	0.97404793401571
C	-2.84436854142584	-3.57331781268346	1.51912343537942

H	-2.34252004994418	-3.36899857073504	2.46641759289687
H	-3.90763067500955	-3.73109188187567	1.71662714764669
H	-2.43326061036711	-4.48933731919667	1.08892089614201
C	3.01251637027680	-0.17233580345740	0.15145572680441
H	3.37896538812536	-1.08947004430911	0.61347455036999
N	3.61410564799214	0.97083380393232	0.93974832215880
C	3.39364820654504	0.75596030478617	2.39368934423539
H	2.33722340847476	0.56277992105699	2.56448891889683
H	3.99062153038369	-0.09818266303825	2.71180675901451
H	3.70363957756417	1.65346823212693	2.92529395278942
C	5.04797009899485	1.21952670560662	0.65680696309733
H	5.16241126799250	1.53377645614106	-0.37478831723417
H	5.38741861276427	2.00192353206639	1.33407150367056
H	5.60406817951448	0.29976461936171	0.83489016482853
N	3.08300229217092	0.98765797627617	-1.90138780331305
H	2.14225867587437	0.81420511807489	-2.25965064070854
H	3.68961996660800	1.11140549603673	-2.70438541783751
N	3.55247445829854	-0.15273667497464	-1.19480497033130
H	3.03268497515333	1.78761990108693	0.66885694519208
C	3.27894495327023	-1.40014442467168	-1.90161428152335
H	2.20218451794351	-1.55159436962318	-2.06606750113405
H	3.78288347378316	-1.36376119818976	-2.86903773265094
H	3.66954738183685	-2.24144517733690	-1.32759102962623

GS4A-E

C	-2.80541219930822	-1.02213163131661	0.18653911624404
C	-1.99006702626971	-0.41079325426506	1.12283863844191
C	-0.84172501161866	0.23759577796859	0.68808647402216
C	-0.52560443596054	0.28397738103401	-0.66620757737938
C	-1.37225875095159	-0.32591227705259	-1.59161661421206
C	-2.51122114664776	-0.98888629801786	-1.17171863095876
H	-2.24361330578417	-0.44613346209177	2.17497829216230
H	-0.20054833331549	0.72479141771460	1.41336694219118
H	-1.12253943282430	-0.28675818168899	-2.64515849170005
H	-3.16501623568974	-1.48012765279423	-1.88177135678309
Cl	-4.22304636666523	-1.86582970598266	0.72137776747745

C	0.68052468358840	1.02391538326622	-1.15096578387442
C	1.82218670447719	1.18985596909524	-0.22753658662630
C	2.37808715237927	0.06941982224560	0.48962920880148
C	1.89221722950619	-1.33867338260320	0.09963832934994
O	0.70398848137866	1.47246812377437	-2.29087267611330
O	3.28672941792299	0.12874886212966	1.31135402713415
O	1.84346901442873	-1.70126584311939	-1.05272687397606
O	1.63575465839230	-2.08568765325201	1.15857936570140
C	1.25044695583970	-3.46684480401388	0.91512932173131
H	1.51373721238499	-3.98110128881607	1.83836693493064
H	1.85843929366392	-3.85503096083866	0.09813305074106
C	-0.23019758623337	-3.57135317114946	0.61862461540780
H	-0.48976333485754	-3.01767013546536	-0.28496978752527
H	-0.49004076996866	-4.62220249993684	0.46906778109221
H	-0.81987707959838	-3.18322394678868	1.45101333967831
C	2.53654150786998	2.37486394918032	-0.12340662489337
H	3.52605476623820	2.34467328864442	0.32228607431344
N	2.15429082833176	3.57939777103214	-0.48065316926970
H	2.81999768372823	4.34170299632894	-0.42677004491117
N	0.85657876314517	3.88196800494065	-0.89761050498235
C	0.56226538386820	5.29387937742748	-0.66917450306151
H	0.76904261748608	3.63921046422283	-1.88137615148230
H	1.30366423193048	5.96138197025605	-1.12763905798453
H	-0.41547557055031	5.50113703407308	-1.10376782337588
H	0.52104371968320	5.48823745585910	0.40401676968870

GS4A-E-NH_3

C	-3.19999518931562	-1.20224775159080	0.57614595138348
C	-2.34767142972594	-0.65017437663357	1.51694310135274
C	-1.09644268512750	-0.20910304994452	1.10789756243798
C	-0.71262845186571	-0.30767989850550	-0.22592981996897
C	-1.59530760274399	-0.85451926617549	-1.15696651758516
C	-2.84015570850535	-1.31068398448753	-0.76208517911074
H	-2.65336089740386	-0.57364618754237	2.55292796021963
H	-0.42160379774347	0.22580797580191	1.83577560455177
H	-1.29118442190501	-0.93145856367195	-2.19392374234869

H	-3.52583081365040	-1.75118322282834	-1.47535865641486
Cl	-4.75283093599788	-1.78713559338660	1.08107029841281
C	0.62437581416934	0.18537092691595	-0.68154848000737
C	1.70245631908681	0.33259261415351	0.34082523471658
C	2.16102454517604	-0.80179097922761	1.09508373440276
C	1.54215289197621	-2.16536305338234	0.74044799816996
O	0.82737327459534	0.42776690593126	-1.85846637607561
O	3.04258539779036	-0.79775856387318	1.95076185318298
O	1.47946356020376	-2.56987024545566	-0.39749056658729
O	1.15984128895948	-2.82710912000486	1.81870812980657
C	0.58138292677304	-4.14568078601697	1.61401580830652
H	0.74297674418906	-4.65590339810325	2.56251813960787
H	1.14392821622541	-4.65177287136939	0.82967901628784
C	-0.89110082356102	-4.03905373108301	1.27806931780525
H	-1.04254736906611	-3.48658273880261	0.34936789522784
H	-1.30350882934972	-5.04359319458666	1.15594556466960
H	-1.43494337609567	-3.53541518126957	2.07936481657427
C	2.36817381409760	1.52097162658720	0.56766117628869
H	3.28185737976383	1.49044471274369	1.15386991383393
N	2.01575054008251	2.73167210525226	0.18834664943325
H	2.62818720625116	3.50999147687332	0.40391857216322
N	0.78170769074811	3.00685110664265	-0.40836491533941
C	0.16725412230530	4.17056758048925	0.22519303848525
H	0.94560649860843	3.19492143461087	-1.40369535319324
H	0.82896174056639	5.04795925388575	0.23527948653105
H	-0.73361982577377	4.41879219277664	-0.33781329010019
H	-0.11357234613730	3.92770124366701	1.25213623959853
N	1.35150776345764	3.70344944503786	-3.35740377522601
H	1.47852806905664	2.83111649810026	-3.86071219166706
H	0.60767177013308	4.20705641079983	-3.82986314095786
H	2.20023692975280	4.24389224767256	-3.49043105886794

GS4A-E-AcOH

C	-2.75723089306670	-0.86893181970837	0.76622301306710
C	-1.74041480155319	-0.21765405737660	1.44350286310299
C	-0.70924635741675	0.35400839063975	0.71153153500492

C	-0.70717338564845	0.28334909371621	-0.67872028266301
C	-1.75111564692430	-0.36525171734596	-1.33938176989692
C	-2.77791438724671	-0.95025242921669	-0.62167082476082
H	-1.75064607362189	-0.16387291761721	2.52482645734254
H	0.08869545153176	0.87107210929559	1.23128077779993
H	-1.74293460992501	-0.42131306248280	-2.42108729538451
H	-3.58418162913228	-1.47038484151862	-1.12381089944656
Cl	-4.03194485140902	-1.61575189463263	1.67190188950310
C	0.38376323763741	0.91366076984580	-1.47097182196542
C	1.68721196388022	1.16086101417967	-0.81228538837284
C	2.41272954241192	0.08219598585196	-0.18763918573047
C	1.85344103214218	-1.33853187992561	-0.38504985034032
O	0.19439036719050	1.19116642889769	-2.65328929680800
O	3.47729095745699	0.18624844183958	0.41047830534328
O	1.51481630146214	-1.74725218488504	-1.47151653449060
O	1.86034520652175	-2.03265289874042	0.73787039687854
C	1.38986832512207	-3.40770538681814	0.66734017534343
H	1.85942313643025	-3.89146280189719	1.52257102404967
H	1.76558200456412	-3.85379461350883	-0.25335065585434
C	-0.12067777253124	-3.46333363568390	0.75055353474242
H	-0.57955340589586	-2.94208446441385	-0.09100528231324
H	-0.44077234333971	-4.50786983276447	0.72843012369773
H	-0.47328953877072	-3.01170037031692	1.67961077809484
C	2.32790984701427	2.38677318856218	-0.82854502523206
H	3.37306391464696	2.41922583384814	-0.53631061594836
N	1.81849860846592	3.56249365877954	-1.12323920746198
H	2.42896979270402	4.37150773245477	-1.14617171183631
N	0.45539019206837	3.75693915964735	-1.35965766221000
C	0.01287781915928	5.01085841004887	-0.75386232076218
H	0.32179751199490	3.78012102955011	-2.36939110526390
H	0.61566614090707	5.87097680999095	-1.07474950062429
H	-1.02292881575718	5.17548721766610	-1.05188337887895
H	0.06079367479556	4.92710305872318	0.33325500003797
O	1.20466173589815	3.91074576327905	-4.33942325538756
C	2.12093813191786	3.17697456232061	-4.66219286640445
O	2.22441581363041	1.92248737786386	-4.23622667829051

H	1.45971931479976	1.69769828530681	-3.63781985456768
C	3.24979680321477	3.57887017985017	-5.56735173296350
H	4.19219715878821	3.50021753478193	-5.02067289612513
H	3.10531795954939	4.59927120539013	-5.91542743233443
H	3.29915256633275	2.89348756652318	-6.41577154168998

GS4A-Z

C	-2.85573599504557	-1.99301121358570	-0.03649338109665
C	-2.65879548033711	-1.66600652985487	1.29957124514468
C	-1.90913569412810	-0.54484842494182	1.60942244594263
C	-1.33853009510023	0.22709666241711	0.59880271833948
C	-1.54992219034462	-0.11972340878264	-0.73181169838038
C	-2.32059335758016	-1.22848202612792	-1.05773670499274
H	-3.08620773043890	-2.28397315632192	2.07958729958588
H	-1.75008906706216	-0.26891870530803	2.64529527100297
H	-1.13466333766641	0.48664416269075	-1.52876890224322
H	-2.49445878593923	-1.49808656032326	-2.09200006496294
Cl	-3.78076752462913	-3.40643185925546	-0.43232474569715
C	-0.58379759541701	1.46915470985207	0.96559340268429
C	0.59649172301714	1.85413617392311	0.19101139199787
C	1.34821780779892	0.91135557034354	-0.59431700549219
C	1.44919527940654	-0.53366706983082	-0.05805797036821
O	-1.02272004742672	2.18014871169117	1.86677670193332
O	1.97424338194721	1.17075570042469	-1.61999138482810
O	1.54850658835430	-0.77277578537005	1.12233146127496
O	1.51083506680686	-1.42174328780847	-1.03370526599226
C	1.72153786987434	-2.80819057368027	-0.65167995801967
H	2.43867136963625	-2.83353463412589	0.16882291184893
H	2.16752457580855	-3.26082135723764	-1.53631249268881
C	0.41518084412374	-3.47671563935960	-0.28076720005594
H	-0.04391431537027	-2.99072722817921	0.58140834277053
H	-0.28620058915591	-3.44761171051066	-1.11649767307159
H	0.61052903244496	-4.52180175803841	-0.02799934777693
C	0.84067985977155	3.23368785076744	0.15930023720474
H	0.03930741500583	3.90419385723326	0.45239093807364
N	1.94465453679134	3.85694204127307	-0.15740307882833

H	1.93009058923737	4.86897668044391	-0.19783105765503
N	3.18397053820070	3.24538571071207	-0.33999437322059
C	4.23913712523974	4.24689863862172	-0.47880213395598
H	3.11889032274978	2.64479828095543	-1.16202458197351
H	5.16433317895623	3.71601541976132	-0.70056628287500
H	4.03844904513671	4.96546620710709	-1.28310051601729
H	4.36069211533344	4.78423639042487	0.46364714238861

GS4A-Z-NH_3

C	-3.89057483098487	-0.85331618859736	0.24474711969585
C	-3.02173098903165	-0.25376811468374	1.14224207524306
C	-1.68058308801206	-0.13747090538302	0.80211808005895
C	-1.21412974670748	-0.63062256111813	-0.41363019070385
C	-2.10440819961458	-1.24839746923098	-1.29017861287835
C	-3.44905841869901	-1.35219032870612	-0.97330612876426
H	-3.38691870363559	0.11579608828449	2.09250338253897
H	-0.99375477347368	0.32123687182403	1.50413927313325
H	-1.73872135410008	-1.64295771393563	-2.23108920036224
H	-4.14832589886000	-1.81629827551069	-1.65790382074855
Cl	-5.57179711685575	-0.98943468931554	0.65481330098000
C	0.23833761253086	-0.58487333889148	-0.78120541856921
C	1.01872815786334	0.61087062588268	-0.53035712441052
C	2.46222059303881	0.46360358681225	-0.73224909623654
C	3.12344889228317	-0.83508478055355	-0.21882490808559
O	0.75238462373437	-1.58430995217630	-1.29035744325885
O	3.18468101110028	1.25359487191437	-1.30634099181771
O	3.99096480557407	-1.41447350322608	-0.82368521328625
O	2.67898983600211	-1.14888052173064	0.99100791014281
C	3.22851201372220	-2.34036610687033	1.61143017164685
H	3.07191504923842	-2.18115323544048	2.67753354118289
H	4.29821052355608	-2.37838285288566	1.40432750475170
C	2.51276225053985	-3.58207011227569	1.12228081088121
H	1.44104686692230	-3.51107985854002	1.32002303215863
H	2.66584105891880	-3.72703519685626	0.05178801770784
H	2.90734199699586	-4.45293315268955	1.65162490635991
C	0.38767411488669	1.82881570248048	-0.32869877949193

H	-0.67445161885271	1.91222218148312	-0.53602263104039
N	2.19095549510533	3.04873513290687	0.66101428716747
H	2.79395425490741	3.50389308581534	-0.03288866276986
C	2.14747378487515	3.86311385707924	1.87388800990669
N	0.90768687516631	2.95252442556179	0.11447572580303
H	0.31549327258471	3.77509931254492	0.14862005423128
N	3.77988324130868	4.78337858774359	-1.35450662578180
H	4.38984711456564	5.46082618557812	-0.90814631435219
H	4.36667515584356	4.21412575534919	-1.95612048121215
H	3.15956414049460	5.30712398922293	-1.96367828945061
H	1.58927681968841	3.33888861996590	2.65215500919108
H	1.68911543287384	4.84830571538044	1.70858220821978
H	3.17426974450654	4.00814426278742	2.21207551221954

GS4A-Z-AcOH

C	-2.69899751196815	-2.11748778358694	0.32337089924738
C	-2.19030144501115	-1.94729125524573	1.60525918811905
C	-1.48681548642640	-0.79191622556620	1.89740772811916
C	-1.26791723716680	0.17070033937680	0.91355509891537
C	-1.78915345115396	-0.02002131759534	-0.36219747253804
C	-2.51874972938876	-1.16324307507506	-0.66202425437927
H	-2.34343139501220	-2.71152210694060	2.35728425794091
H	-1.08629582614574	-0.63797728457349	2.89257278153919
H	-1.65114271211532	0.73314101313514	-1.12964766846625
H	-2.93259272606815	-1.31304151065381	-1.65136115685153
Cl	-3.56620178776372	-3.57155635378652	-0.05488403992658
C	-0.53699085454720	1.42859721331740	1.27177637426216
C	0.38171084434217	2.02581055300237	0.30340925915959
C	0.94316478173993	1.29116519832547	-0.79080562956343
C	1.28825321485584	-0.19514009490528	-0.56507404077873
O	-0.77618751761907	1.96978409138282	2.34829760773004
O	1.22543732390915	1.77576763142323	-1.89014835611865
O	1.69686146028952	-0.60507821420327	0.49410329220673
O	1.17103766432924	-0.89573758227251	-1.67768668754063
C	1.58712005189208	-2.28827824542007	-1.62969900489788
H	2.49391919691179	-2.35379689259963	-1.02832429036806

H	1.82106330278047	-2.52790183619432	-2.66600431870488
C	0.48248323993412	-3.16899383105099	-1.08649226290567
H	0.23449883582184	-2.90002158571744	-0.05862749034894
H	-0.41643110036825	-3.08571184093557	-1.69993241504431
H	0.81873917886594	-4.20860160569592	-1.10253551179979
C	0.56335727871130	3.41042928373089	0.43351138712078
H	-0.17227238671579	3.98552948285811	0.98713567107175
N	1.56085826291513	4.12515513300490	-0.01364913703687
H	1.51837513947986	5.13319671370129	0.09399745162151
N	2.73773391565966	3.59380642542313	-0.53700985623343
C	3.82319639831774	4.56692713110403	-0.44793714845115
H	2.58046724684038	3.30516390060359	-1.50166565877145
H	4.68531512217157	4.13852326417546	-0.95692513645935
H	3.57296749513433	5.52540248204264	-0.92104394556051
H	4.07779960564033	4.73826063804285	0.59951074976384
O	3.83232274713839	2.82669536786675	-3.37067082350701
C	3.85402285055908	1.75557127254965	-3.94592232821600
O	2.94915361477739	0.80263078320357	-3.73210295437503
H	2.29654163654840	1.11002319359434	-3.05160300114983
C	4.88578756022194	1.35593168448595	-4.96177124186228
H	5.40464582246959	0.45945086233169	-4.61561802230570
H	5.59686050329641	2.16560150039560	-5.11044245795750
H	4.39128687191700	1.11145348294093	-5.90418543469857

GS4B-E

C	-1.99407718736352	3.21228758912988	2.11448643604739
C	-2.88626984489169	2.14597570947141	2.10773627446696
C	-2.40757019056379	0.87765138086668	1.83460349131434
C	-1.05758906748408	0.67562212418864	1.54691657989886
C	-0.18343975079624	1.75734960910191	1.56286911319424
C	-0.64631827721216	3.03430228073898	1.85441594908144
H	-3.93654931534349	2.31384063770909	2.31192468690747
H	-3.08536721692035	0.03243999758065	1.82506627835180
H	0.87077532462843	1.60712238310666	1.36066108048816
H	0.03135368508496	3.87862934980019	1.87612616080394
Cl	-2.58802524782420	4.80558971924941	2.45967571400354

C	-0.56686709587234	-0.71400975864627	1.28786378504900
C	0.53142453523891	-0.91801667784583	0.35560441201269
C	0.66423532111471	-0.15361533783596	-0.86011691588770
C	-0.46540163404935	0.78980800907236	-1.31347822975590
O	-1.10595377899408	-1.65151277435700	1.88621753091661
O	1.60669926111000	-0.24042873448070	-1.64308896141637
O	-0.26835597432012	1.92254803805241	-1.67862950563881
O	-1.63719990274276	0.17123001676708	-1.33011995223188
C	-2.78374956864999	0.96741251107842	-1.72983023397030
H	-2.61843674414858	1.31744557981753	-2.75008424959495
H	-2.83829920890007	1.83105315349702	-1.06410993076027
C	-4.00552228240486	0.08703283120909	-1.61840115573226
H	-4.14174931127455	-0.25864894143635	-0.59150173078156
H	-4.88801356990723	0.66014561413338	-1.91134660401414
H	-3.92100120940804	-0.78018090201693	-2.27676841739709
C	1.41844498295869	-2.01293100525449	0.42830743618375
H	1.92382955830223	-2.30004943269470	-0.48686079882858
N	1.37561873168034	-2.37414903820122	2.76139417578135
H	1.74063173151697	-3.08002742557783	3.39094335692111
H	0.35467752103119	-2.38094338298101	2.80829634177509
N	1.76371620392291	-2.72134748279744	1.46491785615808
C	2.66729370008944	-3.86475041196593	1.35976429079197
H	2.13507728729865	-4.76922996967128	1.65905949907806
H	3.01132808080278	-3.95728474692272	0.33275555239203
H	3.51835045429135	-3.70186051188511	2.02363068439188

GS4B-E-NH_3

C	-1.61913936295955	3.34250596974455	2.16038009141197
C	-2.59700029495620	2.35627601217699	2.21988646369214
C	-2.25921023051518	1.06112689395431	1.87096199348105
C	-0.96488913320320	0.75303451198077	1.45212368599897
C	-0.00350417880045	1.75741492552976	1.40133403574166
C	-0.32341636511065	3.05986199573453	1.76158499010420
H	-3.60367715933985	2.60607979966973	2.53169783554644
H	-3.00471018446896	0.27593946542457	1.90795449734470
H	1.00788028750176	1.52444742998779	1.08924655792463

H	0.42212938750633	3.84456563522544	1.73047518523703
Cl	-2.03523432798531	4.96910583566462	2.59708968225169
C	-0.63210772063688	-0.65908470017217	1.08316792985072
C	0.51485189891302	-0.89734219949309	0.16881088708925
C	0.59174035065610	-0.25716020808329	-1.11348454730292
C	-0.55051632489463	0.68479946793701	-1.53739984196735
O	-1.31573425236414	-1.57818862048433	1.50413000337606
O	1.49360545363361	-0.39770429960694	-1.93876626112203
O	-0.35857445391037	1.79010215457275	-1.98138119101040
O	-1.73749779304448	0.11157523698474	-1.39628782861468
C	-2.89641705735840	0.92718618820850	-1.71153512537925
H	-2.83428469432034	1.21931201587227	-2.76108196399546
H	-2.85088306072526	1.82486985943546	-1.09147101237789
C	-4.12397978909754	0.09751833543418	-1.41986518683631
H	-4.15263529250665	-0.19436265236535	-0.36800163582784
H	-5.01641389670804	0.68731930242641	-1.64068567384523
H	-4.14116479618682	-0.80239339362306	-2.03833310061033
C	1.50093037450366	-1.84624941419086	0.41619982917272
H	2.10592188548255	-2.16329315420438	-0.42745534520995
N	1.26870516772632	-1.94371883502890	2.75876307920560
H	2.02614384319646	-1.87150311543807	3.43285818783575
H	0.61991415951488	-2.66811542049018	3.09288348828810
N	1.84186281496430	-2.39365468304358	1.55904908052627
C	2.83094786675285	-3.46282259786707	1.64586191407342
H	2.35404150096401	-4.36756355757665	2.02813049708585
H	3.24714568401790	-3.65577418872509	0.65871803953721
H	3.62715971958064	-3.15904425941050	2.32909928078558
N	-0.55237973566585	-4.32995609576772	3.53922242444033
H	-0.81130658689498	-4.70458931804780	2.63192393637102
H	-1.41551887936389	-4.04772774673833	3.99279612926212
H	-0.17648482389670	-5.10309257560699	4.07859898846510

GS4B-E-AcOH

C	-2.23022900271512	2.70318098078044	2.42804283121815
C	-3.05406758316282	1.61710920402153	2.15595275704342
C	-2.48180062757254	0.44311160828918	1.70204510660919

C	-1.10387311418953	0.35903838791642	1.50059943940842
C	-0.29924099016064	1.45977992198524	1.77733969818801
C	-0.85843501767334	2.63874214257918	2.25152164086879
H	-4.12482639169285	1.69696398821347	2.29668274718452
H	-3.10983128431690	-0.41081445939096	1.47791178740665
H	0.77361936332460	1.39577662071309	1.63773751220286
H	-0.23704503227196	3.49595398938841	2.47817845495189
Cl	-2.94217502181191	4.17625631102227	3.00226711734837
C	-0.50330172911494	-0.92691649033174	1.04343596242029
C	0.64140126326801	-0.91040827575629	0.15653164387598
C	0.78177938999255	0.04020140955734	-0.91817878914482
C	-0.37367468947665	0.99581170117351	-1.26687410336850
O	-1.00222562706991	-1.99516017405657	1.43437411474444
O	1.76038684731156	0.11654298686248	-1.65522084434613
O	-0.22246159220378	2.18364297159964	-1.41068105162035
O	-1.50581015856710	0.33572917237955	-1.46354351839104
C	-2.67147128571506	1.13585608062889	-1.79526102951740
H	-2.47027744007234	1.66119424752959	-2.73030580826090
H	-2.80925675173289	1.87092171030766	-0.99997668994090
C	-3.84763993262462	0.19574046337245	-1.90933128376245
H	-4.02106192782653	-0.32484912045434	-0.96520612987749
H	-4.74254824486036	0.77075196347178	-2.15752938723760
H	-3.68052737264922	-0.54274369325334	-2.69629462844118
C	1.58803216016740	-1.95643583917162	0.12473381146517
H	2.11791551097040	-2.13362015033593	-0.80504460299868
N	1.51264082124251	-2.49970041016405	2.41182668644008
H	2.09472355058038	-3.05382573483504	3.02987573847502
H	0.54615786530501	-2.80712382101874	2.51996945526738
N	1.95167224165225	-2.73057978063333	1.10590810638423
C	2.88590716170625	-3.83948270715366	0.91859627526269
H	2.36908002667011	-4.77875682518334	1.12125440956462
H	3.25747844470831	-3.83007727618959	-0.10330110519876
H	3.71669352375390	-3.71926605367403	1.61628032478159
O	-0.73203239649471	-3.44344327993556	4.30052791074794
C	-1.86974906786216	-3.05627944740036	4.48940815552970
O	-2.54229561405527	-2.33540933839082	3.59660854085176

H	-1.97331066509940	-2.17911446747009	2.79400347043283
C	-2.66336029259013	-3.32562180096411	5.73601591383938
H	-3.60686626418872	-3.80752502576329	5.47250153406077
H	-2.89899963421041	-2.37615203714784	6.22202632347811
H	-2.09339341867137	-3.95809965311746	6.41309150205387

GS4B-Z

C	-3.88442139797066	-0.63324858902386	0.33594933017349
C	-3.04037646212657	0.09329375229010	1.16062448022067
C	-1.69117984208637	0.16621517510604	0.84217430793928
C	-1.19183200104253	-0.49483894301942	-0.27728429226433
C	-2.05766256241086	-1.23599620371957	-1.07916353093671
C	-3.41015022397305	-1.29967488949097	-0.78583070434555
H	-3.43108868128693	0.59381913375069	2.03792146521059
H	-1.02489013478263	0.72560451223355	1.48913588778155
H	-1.66627880179293	-1.75932079366719	-1.94377418871757
H	-4.09071309346163	-1.86046110547336	-1.41463951817204
Cl	-5.57563817850169	-0.71647927772538	0.71668348150423
C	0.27111935111846	-0.49263412511093	-0.60988895154717
C	1.03135284669299	0.74292250271975	-0.47071183871265
C	2.45319582526130	0.66437720584545	-0.57622759003840
C	3.15346435289844	-0.68037450715541	-0.32770785491436
O	0.78939914689238	-1.54319374775608	-0.98605212458042
O	3.21697254610149	1.59470221546677	-0.86279195323349
O	3.88948583270647	-1.19698632549028	-1.13244986007326
O	2.96390948118376	-1.08438210119029	0.92277428519116
C	3.65139360124714	-2.29304088221711	1.33975172558538
H	3.71280244310790	-2.20210422154894	2.42355800976614
H	4.65725538267638	-2.28218831671066	0.91923909276227
C	2.88127273871574	-3.52980243268060	0.92767839757822
H	1.86980262383578	-3.50890461304065	1.33874840555835
H	2.81877702703000	-3.60950487436034	-0.15836569420475
H	3.39528542063305	-4.41361760281099	1.31399824278585
C	0.31625830358646	1.96832914711068	-0.48447225990793
H	-0.68613840845288	1.97331140953808	-0.89558215922481
N	1.84168186847318	3.30455853095017	0.72863446045369

H	2.63293853335172	2.91914064657798	0.20431151974087
H	1.98242563089043	4.30162183351313	0.84940003199538
N	0.68704679657296	3.13001950831808	-0.03411447264116
C	-0.16139816686864	4.31421608221042	-0.15694390477439
H	-1.10137292430971	4.03479785416858	-0.62544766490964
H	-0.34517701845856	4.71446878866340	0.84144491524957
H	0.35370273454951	5.05959245372917	-0.76488116629810

GS4B-Z-NH_3

C	-3.91001147912056	-0.80618792148701	0.29695711965869
C	-3.04339584204591	-0.16642825790335	1.16837874923031
C	-1.70057756331240	-0.06969427113538	0.82851859818642
C	-1.22977532946662	-0.62112223360727	-0.36031433969080
C	-2.11830215139903	-1.27820956074259	-1.20964022497237
C	-3.46447952944089	-1.36396435466601	-0.89379784222750
H	-3.41179766801811	0.24924776903904	2.09813542398628
H	-1.01578224863030	0.42080262109334	1.51079470785886
H	-1.74939400483620	-1.71791518979270	-2.12903392213719
H	-4.16233895680645	-1.85858680449896	-1.55819994710633
Cl	-5.59352302333621	-0.91814196988491	0.70551480556000
C	0.22451626126908	-0.59183525491635	-0.72692403436131
C	0.99745547923816	0.61604717614570	-0.52760182175308
C	2.43411155725080	0.48919241717472	-0.74647955874588
C	3.12014686029863	-0.81129066054727	-0.27550616148496
O	0.73591308296411	-1.61567059735759	-1.19056530895242
O	3.14630719320704	1.30325201824093	-1.30466314553628
O	3.95038140141794	-1.39184035085691	-0.93055878675738
O	2.75753475509458	-1.11866674038084	0.96340358572936
C	3.35593564785741	-2.30078647442859	1.55495171853724
H	3.27236990464107	-2.13283718698262	2.62798315760449
H	4.40922512266255	-2.33422931136388	1.27526316918860
C	2.61783212060671	-3.55205293669539	1.12751897873920
H	1.56202184451993	-3.48772573219272	1.39922345487408
H	2.69637499534445	-3.70498690503476	0.05016538668688
H	3.05530901371559	-4.41551955287952	1.63493046518339
C	0.34377096183731	1.83967773858933	-0.38245689647512

H	-0.70694284606191	1.89764276220788	-0.64561555029891
N	2.10072105564066	3.03122796813293	0.64882081032629
H	2.73890828819095	3.50880399409317	-0.00037608052073
H	2.00985978695793	3.62335114359763	1.47025206081497
N	0.82673167864586	2.97457698612142	0.06377463226764
C	0.05106671918113	4.21098100019562	0.02776733274711
H	-0.94725396909121	4.00642606902911	-0.35513768854653
H	-0.01707995319357	4.61867217515616	1.03865654692790
H	0.55224385762999	4.93333473365062	-0.61938643137356
N	3.91865877629668	4.77320770454117	-1.20396306307649
H	4.58542755003303	5.33178401994218	-0.68072733834202
H	4.45701143691907	4.21270463514957	-1.85692477194809
H	3.36951921333866	5.42095933525405	-1.76013778980078

GS4B-Z-AcOH

C	-2.63682188646820	-2.11443591112732	0.49296791241153
C	-1.99920804267658	-1.97878448885368	1.72037791638083
C	-1.25170460375787	-0.83986391842066	1.96284270579028
C	-1.11649483949035	0.13999111286663	0.98064998753226
C	-1.76413470789108	-0.01742601335403	-0.24043228404513
C	-2.53921573313401	-1.14319902717769	-0.48726665221564
H	-2.08957712283339	-2.75590262036517	2.46932629508182
H	-0.75319335666026	-0.71128974992350	2.91649619779164
H	-1.68742298399655	0.74873649691897	-1.00336459085647
H	-3.05137474565141	-1.26685520796223	-1.43325521599909
Cl	-3.56373871477260	-3.54633748502475	0.17656401108775
C	-0.35301992769494	1.39035883539731	1.29353650837044
C	0.45044045915737	2.03478799877766	0.25071850109432
C	0.96665769378273	1.33533632925728	-0.86429738647252
C	1.27703253519804	-0.16469271605372	-0.71352187935723
O	-0.48547198658441	1.90389336850979	2.40114267125405
O	1.27732882377987	1.85486966442809	-1.95360816317462
O	1.77697650688843	-0.62170353407167	0.28613668943471
O	1.03373662660377	-0.82657226482532	-1.83060017362568

C	1.43222711370603	-2.22423552294091	-1.87048805401929
H	2.39332049093310	-2.32008858233235	-1.36517881898815
H	1.55942782866013	-2.43059471310446	-2.93229427932290
C	0.37625141852618	-3.11362381907763	-1.25015955089328
H	0.22837347435693	-2.87244782190065	-0.19647386427904
H	-0.57571243841595	-3.00848680578943	-1.77349307097093
H	0.70119359718650	-4.15418560173956	-1.32666110755936
C	0.53625613939823	3.44560191058817	0.39058317894189
H	-0.23023524850502	3.93684606836166	0.97897926881960
N	1.45267945596633	4.25144393048098	-0.05424539973324
C	1.36838574096431	5.69840379351807	0.14411978869724
N	2.63815070279033	3.79941673589009	-0.62790668178733
H	3.15466352384914	4.60927475432377	-0.95170442725297
H	2.42781691603059	3.20240091970072	-1.43078050950476
O	4.14004409417861	2.60495003905045	-3.13913337030420
C	4.02203859913019	1.61853490636909	-3.83956746864380
O	2.95678443234586	0.82063761871227	-3.78936581257993
H	2.31880561686709	1.16083389666044	-3.10830847367698
C	5.03957713947617	1.16017259245284	-4.84560543371985
H	5.37169039070846	0.15156223245927	-4.59037523913665
H	5.88753790274227	1.84151539264323	-4.85679739159024
H	4.57807306090812	1.11812490105561	-5.83445088901904
H	2.26525496162229	6.02938238866185	0.67012005840445
H	0.48436779083825	5.92916970686645	0.73248950292213
H	1.31033330193725	6.18548021009403	-0.83031500528658

GS5A-E

C	-2.74437241018040	2.32836529251517	0.00337314584280
C	-2.03116526815745	2.81975670227328	-1.07761314844811
C	-0.73324445722755	2.37308921636877	-1.29014835794407
C	-0.15348094731902	1.44188055116943	-0.43628359546169
C	-0.89017076759902	0.96664543917126	0.64418191760777
C	-2.18670537171471	1.40363630026564	0.87235005001026
H	-2.48358771234619	3.54174909976379	-1.74665328696284
H	-0.17170896702827	2.75407071897760	-2.13274554242505
H	-0.45212225976120	0.24112308959805	1.32054890387885

H	-2.75834670347248	1.02890465404155	1.71261153246953
Cl	-4.37063055718843	2.88001998880064	0.27498249219155
C	1.28546480395361	0.97381397522745	-0.62045650678978
C	1.48770780183128	-0.48332571337338	-0.25534985850333
C	0.79151326879069	-1.56578788996830	-0.86194193048951
C	1.14200201388977	-2.97938780099257	-0.35846565671098
O	1.65266720126219	1.27729539455201	-1.95428998276254
O	-0.04376911923413	-1.46357965981715	-1.75467347944547
O	2.27544174937795	-3.36821312323704	-0.19454213568399
O	0.04813533516525	-3.70142242937453	-0.17291298558605
C	0.22555777310867	-5.06815776982698	0.28582670580325
H	1.08256398669642	-5.49981410337342	-0.23118472505943
H	-0.68320512828424	-5.57587447747082	-0.03463517099981
C	0.38963007019790	-5.11364120707401	1.79050494491364
H	1.29559699232047	-4.59097096484373	2.10233220221770
H	-0.47187382493182	-4.65942158621651	2.28490240758383
H	0.46308966106829	-6.15546783664944	2.11187016423869
C	2.35358908120278	-0.54637413056978	0.80577600004423
H	2.76183339710474	-1.41482903727084	1.30256391345316
N	2.69326236121503	0.66866183317052	1.21421883251134
H	3.54811035396820	0.86853659398173	1.71481085064551
N	2.24095330950445	1.64853783619976	0.30227039454485
C	1.82822165307952	2.87156133137584	0.97708252958169
H	2.57746585078308	1.02030047310024	-2.05219197884763
H	2.69168285781991	3.28222498379741	1.50341942120291
H	1.51431744280567	3.58949865210347	0.21987849679370
H	1.01100836929902	2.71249570360490	1.68904825658500

GS5A-Z

C	-4.36971269490257	-0.26639806211528	-0.01246062377620
C	-3.61071591007400	-1.36571796294196	0.35502468278987
C	-2.23990428907494	-1.21275392653210	0.51606392596416
C	-1.64121570972998	0.02934048240125	0.32543572277970
C	-2.42871440500424	1.12473143062138	-0.02346627991066
C	-3.79479951673682	0.98207357039745	-0.20638180722924
H	-4.08339868355871	-2.32675469695432	0.51606543906511

H	-1.64433931071742	-2.06603787295740	0.81856179202019
H	-1.96384568157624	2.09445979839982	-0.15970211112011
H	-4.40910984899625	1.82708811243265	-0.49225555689452
Cl	-6.08259616293005	-0.45229301006426	-0.22850629621439
C	-0.17245587473712	0.24327956579274	0.53815985094168
C	0.76467568508474	-0.69439227602182	-0.01543064991191
C	2.23499018560134	-0.65413166125721	0.30606202127332
C	2.90276120305202	0.72792073467627	0.17352691917311
O	0.21288940132165	1.23746470161660	1.15554662626481
O	2.44947733174251	-1.10993950009493	1.62495825491133
O	3.65372403556995	1.15974998618250	1.02029842053328
O	2.59834206274842	1.32813055857820	-0.95948628916046
C	3.17226554641981	2.64147060685297	-1.19259480384030
H	3.15488640506627	2.74922274698932	-2.27626740323076
H	4.20587973366856	2.63524101967617	-0.84639509959549
C	2.35258333478337	3.71756054817570	-0.51249389304671
H	2.36677479200035	3.59612372077776	0.57176154424663
H	2.77380542058604	4.69545489542093	-0.75826584875270
H	1.31721652555628	3.68866098200535	-0.85875221168057
C	0.56010608016826	-1.74987999821988	-0.84944365459059
H	-0.36257839412071	-2.11027365167021	-1.28237877761957
N	1.71340676162735	-2.37072310890341	-1.14433322098197
H	1.84241116184311	-2.81838955134857	-2.04221617919056
N	2.79431511249450	-1.54020829966224	-0.72990104372476
C	3.96835729677191	-2.31284480506330	-0.34538179340820
H	4.32652797427973	-2.85634695439068	-1.22092287373096
H	4.74710464574545	-1.61650114490131	-0.03091456376500
H	3.76281518580135	-3.01701742799999	0.46582458416553
H	3.17724206022604	-0.58661448989818	1.99402923724690

GS5B-E

C	-2.75602970637964	1.35289027957187	-0.16869432761412
C	-1.90343373890625	1.04962823124089	-1.21771160460765
C	-0.55868211818844	0.84012139957614	-0.94934279907339
C	-0.06696810796164	0.92968952002982	0.35037360099836
C	-0.94129013132263	1.24495191132771	1.38414887856628

C	-2.28987842049401	1.45815531102288	1.13158709053949
H	-2.28673199114194	0.96717078590423	-2.22732762779340
H	0.11237721005949	0.59705580972896	-1.76556055453668
H	-0.56914795022317	1.31229513984084	2.39816560344143
H	-2.97305566994979	1.69504755982242	1.93816335820609
Cl	-4.44788542070958	1.58551033996248	-0.48860641332478
C	1.42019252497625	0.71530247451958	0.61967556118038
C	2.09617230105555	-0.31540498967003	-0.26935629464318
C	1.72664004090116	-1.65795948555078	-0.51855670703976
C	0.49571278942558	-2.17737650310899	0.24302628326313
O	1.66012974742500	0.45443215597706	1.99762027191148
O	2.31892261603698	-2.45511622474345	-1.25007228847772
O	0.35861215945200	-2.02927971327015	1.44035998323217
O	-0.33770138250388	-2.82899200243629	-0.54258897777579
C	-1.53788827152670	-3.37884618327940	0.07056685825816
H	-1.26711839680591	-3.80770314355235	1.03516168297499
H	-1.83909643708807	-4.17481157389170	-0.60872287985153
C	-2.60747003883050	-2.31524831846385	0.20033971219769
H	-3.50746956559847	-2.76454562128145	0.62715746200755
H	-2.27903996068966	-1.50599751332211	0.85398511634890
H	-2.85778225818600	-1.89834092393444	-0.77704343095845
C	3.22290176920695	0.28928786484586	-0.79179889204913
H	4.01675995845368	-0.16272163558251	-1.37170849925983
N	2.16564177765818	1.95229387728777	0.30755619671562
H	1.60000185774487	2.57956542298119	-0.25847776620684
N	3.27712112438398	1.56416022204341	-0.47845119425702
C	4.33275338450158	2.52440353379253	-0.70819417462600
H	4.78429122104984	2.81547138590698	0.24233397022354
H	5.08061873702430	2.06262376813650	-1.35073886651788
H	3.92332290351668	3.40815355040868	-1.20227181809618
H	1.33616518363422	-0.44719927184030	2.15482861664405

GS5B-Z

C	-3.48625657102284	2.27209260402586	0.88317655676983
C	-2.41596944299970	2.87747367864568	0.24497547354207
C	-1.38628736764310	2.08233005053604	-0.24138226680417

C	-1.43505159160081	0.69830361080523	-0.10147523843564
C	-2.53261715270346	0.11176655779735	0.52530267617036
C	-3.55863167371211	0.89389155823264	1.03148042163919
H	-2.38859747984841	3.95369153829828	0.12627253162378
H	-0.55449781147493	2.54943130887601	-0.75579236969072
H	-2.57704280920788	-0.96701399807663	0.62231113960481
H	-4.40677673245133	0.44351051164995	1.53258179586350
Cl	-4.77239426593593	3.25983465578926	1.50572180560625
C	-0.36026373104664	-0.19350133389041	-0.65146638807554
C	1.00645210141831	0.14329867976839	-0.42981289026156
C	2.14212416538959	-0.62523815368002	-1.05169338718807
C	2.05458846061744	-2.15328937895965	-0.86943172100816
O	-0.68050939544881	-1.22236160661020	-1.25954245287370
O	2.26222220964041	-0.36438153756081	-2.43185714992775
O	2.23560030279404	-2.92296347970172	-1.78704671438074
O	1.81269125288157	-2.49087496833338	0.38096408004085
C	1.70793821449387	-3.90735050274724	0.68267736622640
H	1.94814122737938	-3.97112492547798	1.74312100551531
H	2.46386090267841	-4.44215477359906	0.10756509114625
C	0.31093433231602	-4.41519975782084	0.39529346116689
H	-0.42859424229130	-3.84919685824631	0.96592319528375
H	0.07508877266474	-4.33514719428684	-0.66711235000509
H	0.24524245985996	-5.46602614200496	0.68785024084478
C	1.56031413615432	1.11035733844061	0.37161733041714
H	1.06411105958902	1.85467869239416	0.97892973661442
N	3.31649732995387	-0.12164913134246	-0.30470407329274
H	4.03341829750470	0.14725521492307	-0.97439117096766
H	2.43848319860545	-1.21441290088699	-2.86538688719733
N	2.88132134667871	1.04227045724317	0.38135802721415
C	3.79327217936183	1.64680363633738	1.32699040663165
H	4.08325684501428	0.91960551378571	2.09018952720582
H	4.68402677006908	1.99976982256986	0.80437174969792
H	3.29021401232224	2.49362835310684	1.79185934128375

P-AE

C	-2.75805420373289	0.74073895714271	-0.32269235648599

C	-1.98405399023267	0.37634871141767	-1.41233196706769
C	-0.60296321594420	0.47849631222587	-1.31970379066520
C	-0.00722946364013	0.93931900610950	-0.14921312085844
C	-0.80556332868807	1.30627215270839	0.93243131239754
C	-2.18488766564457	1.20762768243196	0.85161941737394
H	-2.45246357218600	0.01597196327268	-2.31971594213221
H	0.01360229802271	0.19866722617384	-2.16614622520231
H	-0.34626128536693	1.65503659224530	1.85048440101537
H	-2.80974888032848	1.48113190472691	1.69268069252916
Cl	-4.48445268928368	0.60253626212270	-0.42422026046876
C	1.46211599435429	1.01823481124330	-0.05520130591157
C	2.44357395423501	0.03278418148698	-0.18493348505935
C	2.31249807803367	-1.39694082339069	-0.39560021479279
C	0.92842742757385	-2.05236952458453	-0.26106542711928
O	3.24755234916599	-2.12844698340200	-0.66281955520446
O	0.41414249969408	-2.67325433207406	-1.15791037443921
O	0.43413871114755	-1.88034445511233	0.95177936132241
C	-0.88977696635114	-2.43003270398484	1.19880627572022
H	-0.86117623285414	-3.49881962858176	0.98194969784118
H	-1.58191101553829	-1.94630493694359	0.50705140599764
C	-1.23566466010240	-2.14848009937525	2.64105345903930
H	-2.23254203881293	-2.54233023464318	2.85147211785921
H	-0.52308842237609	-2.63128757462960	3.31310883518268
H	-1.23945800559807	-1.07460831918143	2.83673325843211
C	3.66440700689425	0.72789492533574	-0.02692241820836
H	4.66610842229884	0.32364095722277	-0.04985929561991
N	3.45819023152850	2.00896163667501	0.18667226893992
N	2.11596624785181	2.16899617780548	0.15795060279620
C	1.55566712492987	3.49859474915435	0.34320319232014
H	1.45134363404953	3.71916873353975	1.40635325702676
H	2.23104888133952	4.21579301083109	-0.11856199756962
H	0.58150875556119	3.54625812203125	-0.13876402898862

P-AZ

C	-3.19240923994197	-0.33149236805166	0.54033350978355
C	-2.11080618815185	-0.16124713206551	1.38904597028521

C	-0.85965030491547	-0.59379949304384	0.97453146861812
C	-0.69907561053591	-1.20195929203316	-0.26757596719146
C	-1.80514377146239	-1.37689020022261	-1.09788742535431
C	-3.05611160937739	-0.93487149333451	-0.70357349224916
H	-2.24347949757029	0.30675818936060	2.35637905732941
H	-0.01189935204592	-0.46876203969244	1.63733172828223
H	-1.67704412808296	-1.85272857717234	-2.06270105775850
H	-3.91742080838096	-1.05164271731187	-1.34955463342242
Cl	-4.75486395523172	0.23111564897649	1.03822376891529
C	0.62623281285639	-1.72482061442716	-0.71304678605008
C	1.85664303331237	-1.17418356892711	-0.10058526126891
C	2.23126144245678	0.13753866477084	0.15290538099669
C	1.56408617279887	1.41829901567919	-0.18628417543155
O	0.70195747307470	-2.63122452322788	-1.52410850484053
O	1.70267903345966	2.43848655797933	0.44774328191184
O	0.81945891005007	1.28602287966694	-1.27691090797134
C	0.05792302856056	2.44687396851528	-1.70519901431400
H	-0.11390051200573	2.26964476906687	-2.76587141008309
H	0.68137088530161	3.33236336693898	-1.58238379876063
C	-1.24383628825872	2.55742387298839	-0.94064378733938
H	-1.80490628279145	3.41547869771683	-1.31893640949335
H	-1.84820360869232	1.65961902547400	-1.07788977124186
H	-1.06395386961897	2.70418836532963	0.12547863380552
C	2.92166836830589	-1.93715626681346	0.40328702229495
H	3.01880206321200	-3.01300683519068	0.42326503224176
N	3.86137232376943	-1.16143441227940	0.92553999198697
N	3.43282588254898	0.09263688763540	0.76420753927185
C	4.27031727756195	1.20122491670349	1.20615497083929
H	3.82692450698317	1.68171660735846	2.07698888945868
H	5.24012111337937	0.78099136387831	1.46048486140353
H	4.37727008943221	1.92808047575457	0.40288135534566

P-BE

C	-2.85856482658629	0.97073575876192	0.35836005003789
C	-2.12537620120827	0.38932042758419	1.37991269921333
C	-0.73974980115460	0.39178894292851	1.29766544849386

C	-0.09477497921985	0.96465808944688	0.20466018795875
C	-0.85369064672951	1.55465126335817	-0.80386656122292
C	-2.23810856039890	1.55963618859324	-0.73365046143993
H	-2.62860708864124	-0.05809305183409	2.22816695569896
H	-0.15949621345749	-0.05055959149890	2.09970065371397
H	-0.35692865388242	1.99714095575482	-1.65947728384274
H	-2.83134932568264	2.00658341382327	-1.52194757159794
Cl	-4.59326080106224	0.95881410321200	0.44404831791346
C	1.37822883098137	0.94985615551881	0.11337510560961
C	2.27931586930643	-0.13715251857981	0.28026587278703
C	2.04958202245146	-1.55891211510024	0.47492804363975
C	0.64875386300987	-2.13400703962965	0.19643844932988
O	2.91179445522740	-2.34416390074922	0.81992845182134
O	0.06249422403825	-2.82979051422027	0.98762433665282
O	0.22885764432081	-1.80234737241121	-1.01108093418863
C	-1.10600159048579	-2.24415222993853	-1.38118602843766
H	-1.80706671704981	-1.80552057348138	-0.66845364937118
H	-1.14326674497619	-3.33091525297992	-1.29279871458148
C	-1.36271334825256	-1.77614510483473	-2.79342390348420
H	-0.64300521629312	-2.21721224490496	-3.48630425467657
H	-2.36678383778563	-2.08388350791935	-3.09394328415322
H	-1.30018327549168	-0.68829982350868	-2.85826754397701
C	3.53719416435627	0.43720325045056	0.13775649242555
H	4.52176329772288	-0.00391521204489	0.16886584207087
N	2.04324433540177	2.06982241819142	-0.11520609707863
N	3.34872255892318	1.73065557191878	-0.10333066356302
C	4.36081225192897	2.74742046167761	-0.33533516069648
H	4.28626055072980	3.51537336318439	0.43440511216200
H	5.33909381728285	2.27283632982614	-0.29160913295935
H	4.20772049267694	3.19269203940512	-1.31816252425810

P-BZ

C	-2.20668741318961	2.91575410894272	1.69660370795372
C	-2.94958078142603	1.86094696562980	2.20895419085151
C	-2.39936205867318	0.59020821489958	2.19989610786362
C	-1.12970426660585	0.37295397966364	1.66974883733153

C	-0.39675074169554	1.44841353633077	1.17503567817494
C	-0.93027765656044	2.72820902961089	1.18862817328551
H	-3.94412301947405	2.03476318809403	2.60075365940657
H	-2.96444039441423	-0.24866842574809	2.58826272168053
H	0.60381866255345	1.29857333424764	0.78702941964328
H	-0.36466772645676	3.56956949095238	0.80822929191045
Cl	-2.89205575467165	4.50873527788102	1.69349469972042
C	-0.58247608062397	-1.01602709590729	1.67008784704740
C	0.43139505518554	-1.39428199144827	0.66505646143290
C	0.63963738798712	-1.00537617210475	-0.67965155930935
C	-0.17668458853771	-0.13072786045140	-1.56497058340500
O	-0.93997811547079	-1.82373483980410	2.51229541825361
O	0.28813898720140	0.57006368731291	-2.43405152533951
O	-1.47053871995849	-0.22473655899323	-1.28143123443216
C	-2.35326521835692	0.67887131623576	-1.99256164026489
H	-2.26758580229060	0.47375631493960	-3.06096146562630
H	-2.01490486385650	1.69949638206412	-1.80161776512974
C	-3.75412353818321	0.44321097321194	-1.47912397274714
H	-3.81443102535120	0.65131955994802	-0.40947458856261
H	-4.44356147382957	1.10996848726708	-2.00189669724781
H	-4.06612321920428	-0.58785751722133	-1.65933015563752
C	1.48096579212253	-2.25802360930717	0.92075107804654
H	1.75406925035726	-2.78793737327748	1.82035139382058
N	1.71969457636396	-1.58314481763958	-1.18464146755082
N	2.21580881745060	-2.33148705250705	-0.19665545722777
C	3.42361827267382	-3.11344082178266	-0.41094200619688
H	4.23921850817077	-2.44873532153279	-0.69376967520044
H	3.66741327383667	-3.62528874089682	0.51782661273633
H	3.24784387492749	-3.84304564860989	-1.20112550528154

TS1A

Imaginary frequency: - 296.76 cm^{-1}

C	-3.21106921337246	1.53926786446151	2.72693807639241
C	-3.81731097841570	0.79205326342976	1.72251133885546
C	-3.04118443590147	-0.07911405745793	0.97783182486661
C	-1.66938715070513	-0.18541423111103	1.20995377229411

C	-1.08654094132672	0.57078243214807	2.22145158468743
C	-1.85658863310782	1.43318419374985	2.99375601299016
H	-4.87863376218977	0.89588170782565	1.53208066551779
H	-3.49512575814208	-0.67375039643209	0.19351762880644
H	-0.02410183326785	0.48240267522274	2.41750885375004
H	-1.40826347536542	2.01769930691233	3.78778780678247
Cl	-4.18019286186783	2.63084318891855	3.66896221873346
C	-0.86305717851112	-1.17269978129746	0.41498777377674
C	0.47224223849220	-0.82436957996041	-0.00289256854031
C	0.86198414717312	0.49865934207382	-0.34607791121290
C	-0.19315855233082	1.60797901488352	-0.49769466509689
O	-1.37532147893873	-2.27654725535236	0.18060119107096
O	2.00618936551390	0.85440175520448	-0.66210214921949
O	-0.11462308923472	2.69532408215288	0.01969243819967
O	-1.13238899041975	1.24610627359228	-1.36754490690901
C	-2.18246428046292	2.20898461657667	-1.63414546770259
H	-1.72814621293173	3.10312814574724	-2.06486093608862
H	-2.64692829715952	2.47276509724169	-0.68192962525026
C	-3.16097658710833	1.55315864180748	-2.57969466148706
H	-3.59418780527994	0.65814236147442	-2.12772395851030
H	-3.96841241846579	2.25309637039917	-2.80607864791362
H	-2.67004684960666	1.27511582707125	-3.51503652520533
C	1.43947218545838	-1.85107106032218	-0.37260466191574
H	2.24851605160419	-1.48973508868730	-0.99521053103149
N	1.81432438175081	-2.86303217105703	0.42922498757029
C	1.02766471570010	-3.33015148556103	1.55733645523218
H	1.70059831888993	-3.83939800538142	2.24889934330560
H	0.57639393148941	-2.48289980705871	2.07144408325147
H	0.23762127140494	-4.02169224327884	1.25088294185933
C	2.84231147211986	-3.77644212965483	-0.04766279974111
H	3.45767513622690	-4.10454806557479	0.79179927452771
H	2.39800224165824	-4.65913781319551	-0.52176058955461
H	3.47320868262454	-3.26621436520586	-0.77625497810279
N	0.48444543747076	-2.69530045886586	-1.92266886908946
H	1.10291720248502	-3.27993535606891	-2.47901806334542
H	-0.19532560101809	-3.29048658330101	-1.45826554011254

N	-0.23007350621155	-1.82645986594082	-2.80589959187594
H	-0.73695975178923	-1.19026878868121	-2.19538207813461
C	0.71059607289510	-1.03837034389464	-3.60292258258375
H	1.24776191216682	-1.70117273114909	-4.28603877915378
H	0.13239382464400	-0.32761820155908	-4.19469793078328
H	1.43865105336327	-0.48594629484355	-2.99809925390925

TS1B

Imaginary frequency: - 248.76 cm^{-1}

C	-3.12328836626572	1.67536380837687	2.48545195276720
C	-3.74596982099970	1.04552129758510	1.41287333301208
C	-3.02577728576033	0.12798120944451	0.66800193165556
C	-1.68914472356036	-0.13868950497786	0.96710219706775
C	-1.08953917881851	0.50086490744236	2.04631602683156
C	-1.80676242814523	1.40779479949895	2.81916761798304
H	-4.77696427234599	1.27392059123768	1.17124748223201
H	-3.49551134796769	-0.37798810926889	-0.16750819344822
H	-0.05640253822836	0.28652263314974	2.29525394752244
H	-1.34573842640257	1.90210369266788	3.66551881704106
Cl	-4.02350356142272	2.82405628965982	3.42840639281211
C	-0.95329255530644	-1.18088853818019	0.17382984852795
C	0.41427994266669	-0.95924653798869	-0.20664026400410
C	0.99136033949891	0.32209704083524	-0.41967190703402
C	0.11392170664892	1.58347765741998	-0.49040812020940
O	-1.56475958881583	-2.23232251682490	-0.07924531406698
O	2.18712289618949	0.53604466013719	-0.66625649304636
O	0.33688350565353	2.59370091320602	0.13116663389105
O	-0.83867079427617	1.45042115334213	-1.40776755443695
C	-1.72870601190175	2.58074988979908	-1.58770057697158
H	-1.13651111826944	3.43624989782669	-1.91755099825806
H	-2.17541473924479	2.81359581605174	-0.61908388286796
C	-2.76728410778934	2.17801729865401	-2.60778169629991
H	-3.33865390516992	1.31557688371755	-2.25786505112522
H	-3.45715095628145	3.00985086636294	-2.76698972902789
H	-2.29843630918022	1.92893579202016	-3.56231447559287
C	1.29293270080377	-2.07413568167847	-0.58614930920083

H	2.11543790451998	-1.79045150656538	-1.23358695643605
N	1.58424839828513	-3.08817966896392	0.23000968852183
C	0.76822998775090	-3.45823229054079	1.37508323696180
H	1.40484157437053	-3.98043033162492	2.09005544902046
H	0.36531750149671	-2.56593413989303	1.85208795198969
H	-0.05832411476535	-4.11201358529519	1.08540506572990
C	2.70310752237417	-3.97679519922937	-0.06107900305926
H	3.35784107680603	-4.03025336637161	0.81220675920298
H	2.35283222815098	-4.98553421467984	-0.29469170640804
H	3.26604716756874	-3.59207652516873	-0.91015217772004
N	0.13077287081713	-1.72287277033017	-3.10653857577254
H	-0.11351095494705	-0.88951321753736	-2.57662780833791
H	-0.65815095795382	-1.91844193310463	-3.71921813981865
N	0.25718956940165	-2.78213313453290	-2.16621626996965
H	-0.63139465027746	-2.99706129345994	-1.71694694274990
C	0.85268312791016	-3.96116109022216	-2.77884137884334
H	1.87890853996463	-3.73560207387442	-3.07339282497979
H	0.84595352978861	-4.77314125898013	-2.05207574751519
H	0.28605062342995	-4.27334860914157	-3.66208323556942

TS2A

Imaginary frequency: -899.59 cm^{-1}

C	-4.30791273881583	1.15453360428503	0.11468326186749
C	-3.59891335928180	1.36902868598458	-1.06170747439640
C	-2.33627376831513	0.81975397083063	-1.19060796182685
C	-1.77405892070056	0.08761985190692	-0.14449364815417
C	-2.50515776905591	-0.12536228201196	1.01738858969399
C	-3.78204964921581	0.40687137932252	1.15284142654967
H	-4.03333763838586	1.95840961103460	-1.85970283788831
H	-1.77048202362683	0.97481551131051	-2.10178458989792
H	-2.08393234879666	-0.71807317639140	1.82100606877827
H	-4.35752011978682	0.24212053576091	2.05521313130945
Cl	-5.89313045353487	1.84187810998119	0.28072386114355
C	-0.43284594185689	-0.52692299210211	-0.32108583245455
C	0.59843375553674	-0.44331499152419	0.59727361054108
C	0.65000840537983	0.47224191360463	1.70142217417371

C	-0.30634854448278	1.67081575252086	1.82439893438383
O	-0.31284036973564	-1.22325345879703	-1.41432926507363
O	1.50896227649444	0.43142847028175	2.58053441015488
O	-0.94596326177890	1.89783235473331	2.82264094113872
O	-0.24474525710666	2.45806237719767	0.76047830646030
C	-1.10934575488051	3.62378547637772	0.77013922345493
H	-0.83231773700583	4.25080678064938	1.61930279851161
H	-2.13540300140785	3.27720626297135	0.90763365978734
C	-0.92330425198191	4.33515022961595	-0.54894722621050
H	-1.19675923537630	3.68410979779755	-1.38175890940727
H	-1.56566739386216	5.21833238119847	-0.57403844168092
H	0.11288041541680	4.65658234684307	-0.67471052126379
C	1.81932599148012	-1.33143798447502	0.45226436995147
H	2.69073611928619	-0.80100620093114	0.84207548944917
N	1.71000474552198	-2.55984796135016	1.19617385286276
C	0.59688836055339	-3.42964240514455	0.84255507863835
H	0.54403164613043	-4.24115916668503	1.57122847388150
H	-0.34114933496491	-2.87550293870334	0.88298954341864
H	0.69888399079396	-3.87895943870016	-0.15708007953881
C	2.96637218497246	-3.28766263552168	1.31206649015309
H	2.86763292545892	-4.04975377897800	2.08822899618717
H	3.26608680751035	-3.79882085134495	0.38347267912826
H	3.76317118618209	-2.59871036174196	1.60044889418230
N	2.06071709971161	-1.56947386115015	-1.01374683826449
H	2.56813239977526	-2.44267391419626	-1.15240006716832
H	0.81693223975965	-1.52869028574579	-1.42439336725256
N	2.74864590343143	-0.46603185810057	-1.59774565779655
H	3.70098812391444	-0.47291076515871	-1.24196968781031
C	2.77182047683704	-0.60231310095582	-3.05258699547592
H	1.75641297438221	-0.51196695766051	-3.44270882953424
H	3.37540595283788	0.21107296691727	-3.45739920460284
H	3.19698489258918	-1.55896700375542	-3.38398683010320

TS2B

Imaginary frequency: -848.85 cm^{-1}

C	-3.07627952400859	-2.88728522941333	-0.19984048841477

C	-3.02988497138693	-2.27359769399859	-1.44648151014226
C	-2.27736748015718	-1.12291037388649	-1.59799664095245
C	-1.55417519372965	-0.60682970303132	-0.52267959594533
C	-1.62931602553683	-1.22642465720083	0.71960304872769
C	-2.39625852530886	-2.37214588751778	0.88932141863585
H	-3.57487994808677	-2.69616244379414	-2.28152175224686
H	-2.22891738640001	-0.63212549381310	-2.56292714964307
H	-1.09257944179448	-0.80998133368919	1.56377199573286
H	-2.45873317191532	-2.85922376262963	1.85437046620278
Cl	-4.01187100497263	-4.33579806203352	-0.00628958300447
C	-0.75702355463759	0.63151037667442	-0.69813314095228
C	0.54362861388518	0.78761768247730	-0.25052423540936
C	1.41731759227752	-0.30294504161418	0.07211813447604
C	1.09338556220579	-1.73275055853579	-0.38569897654705
O	-1.39528332105445	1.60746869217271	-1.27980759134143
O	2.53426741511692	-0.17250277568795	0.57097720854772
O	0.99135929941529	-2.03232866611727	-1.55223530650626
O	1.06826867231646	-2.58417020766916	0.62697390958201
C	0.79125266133913	-3.96922381657502	0.29546338138406
H	1.61743476048151	-4.34819789948318	-0.30884096199301
H	-0.12308358193213	-3.99855772128453	-0.30028138760654
C	0.64140630074524	-4.72309942979510	1.59549762966941
H	1.55754345699721	-4.66577915628161	2.18720462339710
H	-0.18627881500995	-4.31910713993161	2.18245360044520
H	0.43297348890420	-5.77334948544608	1.37937554472357
C	1.14335676071259	2.18672767557160	-0.16509588697368
H	2.23204835030888	2.11774050145858	-0.23197055928817
N	0.84782111376193	2.92406356544039	1.04699284725900
C	-0.54872858726921	2.92152702160329	1.46173918078658
H	-0.65045056027790	3.57317826477543	2.33144814679024
H	-1.19304722619437	3.31024462379628	0.67174891444460
H	-0.90819436500603	1.91928556357603	1.73852684268555
C	1.71908284735930	2.54598172194255	2.15281472775775
H	1.56273847374549	3.24539592883871	2.97715544941729
H	1.52016660837513	1.52849102323243	2.51972786441326
H	2.76285086876649	2.60062104835348	1.83999795479030

N	0.78389405521481	4.36172986745588	-1.29696755843883
H	1.75724480675733	4.57142141960272	-1.07534637703807
H	0.24893057918987	4.67543670725791	-0.49470961877986
N	0.64512196342886	2.94752056229486	-1.36711751600511
H	-0.58985627436828	2.42704558947776	-1.41201562187326
C	1.31969480675656	2.47714255793365	-2.58514824277431
H	0.85115175779412	2.95154383794487	-3.44575534492860
H	2.38232655068472	2.73760789382036	-2.55071259422791
H	1.21694159250664	1.39519441372822	-2.65918524883594

TS3A

Imaginary frequency: -176.42 cm^{-1}

C	-3.67879610306543	2.05482328038599	-0.50149489608657
C	-2.50713178877222	2.11970360858611	-1.23401989506276
C	-1.51923038277730	1.17049205366835	-1.00326765086329
C	-1.70982683752504	0.17209526408378	-0.05519629437116
C	-2.91421905781216	0.10288557095322	0.64466297664751
C	-3.89895273259590	1.05189820684135	0.43583692087165
H	-2.36326763394469	2.90154155337941	-1.96922795692828
H	-0.59676180359294	1.20761067258555	-1.57101479751472
H	-3.06846607113455	-0.68424662148484	1.37355656325632
H	-4.82766524913267	1.02163726553833	0.99214138932936
Cl	-4.90573774082260	3.25477781486616	-0.75873445047689
C	-0.66040172345396	-0.84993450839486	0.17762886398021
C	0.66002564721925	-0.55924773480650	0.43241493976619
C	1.15304864714089	0.75960603995948	0.72809478851364
C	0.22407160242866	1.83558390930409	1.31240399034170
O	-1.08595929928757	-2.08469335426974	0.07275129677277
O	2.34333928772622	1.06108341713207	0.68688037611007
O	-0.40088025551459	1.66540349166756	2.33239732860951
O	0.29142372213817	2.96997231599120	0.63597926182800
C	-0.54431225811710	4.05452987756068	1.11671226216888
H	-0.18627234167717	4.34960517142067	2.10468045995402
H	-1.56518739469337	3.67914395091934	1.20991271565958
C	-0.44614239366464	5.17897608032038	0.11343548557434
H	-0.80459801557350	4.85484674222044	-0.86604752713611

H	-1.06429006690092	6.01443866988563	0.44961938878967
H	0.58458807971105	5.52687595307738	0.01719514794156
C	1.72956382958549	-1.64289739532532	0.34867917868084
H	2.54479990994276	-1.39103098198300	1.03564946975168
N	1.12894793325065	-2.93759857233695	0.76453598198725
C	1.83700583307576	-4.12154369815144	0.27225269940106
H	1.33696152767172	-5.00265351283487	0.67717395266031
H	1.80024689648214	-4.15514874116410	-0.81363117500632
H	2.88334954584833	-4.12310793246864	0.59768052569759
C	1.01333223670764	-2.97363034550557	2.22909750580779
H	0.45429644329803	-3.86287526279419	2.52177931077956
H	2.00391118081370	-2.99740538768916	2.69795781570965
H	0.47828758974246	-2.08860905175241	2.57647059737444
N	2.35869059523964	-1.73773523676297	-0.95527136305607
H	2.94603475264513	-0.91432604477367	-1.07030747452099
H	-0.20012554753012	-2.68815808940228	0.30493472256228
N	1.40886062446383	-1.82952534698510	-2.01059580883505
H	0.98801936551526	-0.91689416039526	-2.16973496614191
C	2.07293743448410	-2.26902406875977	-3.23052743883541
H	2.43845737657735	-3.28965055825090	-3.09775293470423
H	2.92209650003487	-1.62919788265631	-3.51016850178955
H	1.34592813584530	-2.25839642139931	-4.04552278519816

TS3B

Imaginary frequency: - 590.00 cm^{-1}

C	-4.30960583915279	-0.78580251082851	-1.04882125625192
C	-3.65173476909631	-1.73790937837786	-0.27918605401630
C	-2.26933714958863	-1.72122705104343	-0.23182369648620
C	-1.55087673653455	-0.74767504054924	-0.92566303852417
C	-2.22729127131775	0.18601990105938	-1.69986058697484
C	-3.61579519251262	0.17050688337911	-1.76772507884406
H	-4.21794910052153	-2.47745590674804	0.27348241891962
H	-1.74158418583180	-2.45679266783003	0.36448532401742
H	-1.67292057601966	0.93105504044102	-2.25910295869651
H	-4.14827048268171	0.89654673055219	-2.36943760402620
Cl	-6.04498108569169	-0.80129662322757	-1.10834595614361

C	-0.06419566632364	-0.77235573650838	-0.87885355947899
C	0.73059204781362	0.28237100917495	-0.49477532915952
C	0.21651744653649	1.55650314647680	-0.04833764772755
C	-1.16301591759209	1.68704409609435	0.62967838696344
O	0.45002074760061	-1.89224433279841	-1.32057228508419
O	0.85630142527056	2.60084139684873	-0.09231217605664
O	-1.94508033226670	2.56252639210724	0.35332648607050
O	-1.31080457354861	0.79497076591201	1.59839226677577
C	-2.56239521619792	0.81674061266528	2.33450156574860
H	-2.67289138658961	-0.20407797080745	2.69804897869154
H	-3.36801992101519	1.03878771800091	1.63437991556825
C	-2.50431494493183	1.81371295549711	3.47229174224436
H	-1.67546678632328	1.58110991828579	4.14449905816717
H	-2.38323746453421	2.83094956517884	3.09622473847374
H	-3.43581284343462	1.76266673148101	4.04134040544397
C	2.25293031545824	0.16254195651046	-0.55110567591980
H	2.67919612583820	1.14235000390079	-0.78358460511923
N	2.61883689677783	-0.79337282242744	-1.62802880538766
C	2.42412647653242	-0.15644027085952	-2.93943144618755
H	1.41386579493425	0.24761825924566	-3.00767937405434
H	3.14377961967996	0.65682389199919	-3.08397597286530
H	2.56127328802245	-0.90374629313659	-3.72118419837681
C	3.97646527751353	-1.33758232956036	-1.52412073376347
H	4.07479911486406	-1.93301882134367	-0.62115209310616
H	4.14955824360498	-1.97043216631244	-2.39561408012676
H	4.72107379881787	-0.53420981087042	-1.51198787414224
N	2.28121941758156	-1.46294778442782	1.17104575242464
H	1.41070834391977	-1.27368174641513	1.67036430338500
H	2.92868791834708	-1.86158575708422	1.84195193504259
N	2.85128939110911	-0.24467253584641	0.71098181223042
H	1.53772562067964	-1.64365377569918	-1.46577337752249
C	2.81343299430369	0.79849361561585	1.72856437148417
H	3.41822683663893	0.47315583212316	2.57753750596974
H	3.23195068660620	1.72303589504633	1.32809613855851
H	1.79300361325565	0.99580901510601	2.08926235786302

TS4A

Imaginary frequency: - 173.09 cm^{-1}

C	-3.36751804783204	2.44694898702577	-1.12208800412822
C	-2.06875002940029	2.59718920421395	-1.57780707386572
C	-1.14316428256386	1.59720632076797	-1.30357768845743
C	-1.51871230746217	0.45788505165019	-0.59942400936291
C	-2.84120914741860	0.31640145777001	-0.17813931563506
C	-3.76992859221272	1.31306663919848	-0.42396655082201
H	-1.78172577727217	3.48455924090660	-2.12842053382098
H	-0.12002464496420	1.70657568750854	-1.64359702871457
H	-3.13293086602211	-0.57465351486785	0.36555370361031
H	-4.79235706912979	1.22178419422948	-0.07801589439123
Cl	-4.52247314983196	3.70903978870864	-1.42232349675255
C	-0.53348525625985	-0.64352375889008	-0.33248771032343
C	0.84960063573534	-0.29167776832342	-0.03841769527272
C	1.19687504268387	0.77142982417349	0.83035127191046
C	0.08219030191420	1.55593058709068	1.53194694644236
O	-0.93296809264083	-1.80785909412109	-0.37198240625750
O	2.34585045999940	1.08988379935779	1.18112657205219
O	-0.66245700843148	1.05265090927534	2.34255777546354
O	0.09942476166659	2.84187429941248	1.21397196824327
C	-0.94466998087241	3.66341246435380	1.79537304553148
H	-0.80195648639991	3.68810177238817	2.87729677156144
H	-1.90528189086039	3.19225074875611	1.57749177633055
C	-0.83801162819501	5.03654845334584	1.17498798049426
H	-0.98037217641867	4.98337419325823	0.09348227026758
H	-1.61288594372278	5.68185795014764	1.59502607901032
H	0.13631606555535	5.48391238970944	1.38340861440934
C	1.93958783732886	-1.15131443722913	-0.40555428370111
H	2.87023471382961	-1.00386910876272	0.12840265764365
N	1.55008753212471	-2.83267178071786	0.92448114883463
C	2.43671810341115	-3.95063990242178	0.64717649168404
H	2.19757692271896	-4.83348886608453	1.25306292511766
H	2.36354720447122	-4.22367647363714	-0.40717228417486
H	3.46763629143056	-3.65409311699633	0.86119624753188
C	1.56653387912630	-2.39437829191833	2.31150131458035

H	1.28780479147239	-3.19724429748376	3.00518690298143
H	2.57196044096117	-2.04909291876548	2.56769981497085
H	0.86970288429851	-1.56388228645036	2.43572731939946
N	2.10432303482287	-1.70247669736725	-1.60134880846308
H	2.96393416864522	-2.20477914705149	-1.78659860099268
H	0.60185197788274	-3.02210951803807	0.61816279432480
N	1.08853517084811	-1.77299992359543	-2.55846521488201
H	1.46657892047085	-1.38551695258990	-3.41568600202460
C	0.64948163631498	-3.15052750719016	-2.78488199741162
H	0.15157790811120	-3.51562108512684	-1.88669871519774
H	1.48000384509343	-3.82120466576266	-3.04048583537577
H	-0.06705215300638	-3.14458284985696	-3.60803324236807

TS4B

Imaginary frequency: - 229.24 cm^{-1}

C	-4.21567589155556	-1.04968550373374	-1.19956742947371
C	-3.75180758193944	-1.82747022425989	-0.14416045437302
C	-2.40726134363048	-1.77913704959582	0.18144220878116
C	-1.53775017822098	-0.94021119016358	-0.51588857340998
C	-2.02246614943543	-0.17709630462958	-1.57096988435155
C	-3.36670840751441	-0.23252037591174	-1.92503555972280
H	-4.43646629076722	-2.45937736296761	0.40849130225070
H	-2.02799545200006	-2.38524276270542	0.99653879659273
H	-1.35045583842331	0.46653696577479	-2.12707832119971
H	-3.74724374582166	0.35731169444420	-2.74996012195915
Cl	-5.90252563116935	-1.10911152964067	-1.61564611470907
C	-0.07156045251104	-0.96753618354876	-0.19060148217593
C	0.65140499448953	0.22199304220119	0.08052882287434
C	0.08548609266808	1.50511953197350	0.33633391796288
C	-1.40713151242459	1.69277984227844	0.65204415605818
O	0.48005457482909	-2.09113690356663	-0.25903011695932
O	0.73242082088151	2.55819563950509	0.41828558974726
O	-2.11242602800369	2.46850759377360	0.05340692030842
O	-1.76680292172157	0.99647793585418	1.72342374389344
C	-3.14880205233530	1.10764845242629	2.15301490609421
H	-3.33902858779349	0.17260306664252	2.67862066544963

H	-3.78189037441667	1.15587821044267	1.26677809046271
C	-3.34196539968353	2.30692071212029	3.05688607917211
H	-2.68279198419635	2.24347988232014	3.92551214419534
H	-3.13832023382858	3.23648262187835	2.52274319705925
H	-4.37633439984298	2.32837517188455	3.40894327842864
C	2.13443997386623	0.24128586947621	0.07845605586228
H	2.56058144081821	1.19199999230609	-0.21794526113114
N	2.49730483649648	-0.54192035653061	-1.75339540928634
C	2.15422240709724	0.44791259619930	-2.76923411586203
H	1.12059800311380	0.76560996219825	-2.62594694798433
H	2.81082169149544	1.31397914834889	-2.65617426843675
H	2.27053055530855	0.04716983082657	-3.78113446739743
C	3.87557366937442	-1.01263477682523	-1.83027002884081
H	4.04895085654680	-1.74711010366848	-1.04303475961221
H	4.08835447247890	-1.47332375130698	-2.80027244912914
H	4.55193656255881	-0.16750074158555	-1.68569283960774
N	2.37522813374895	-1.46303730894047	1.75572506217290
H	3.14819388988190	-2.11818467475221	1.85199186048764
H	1.68615972681480	-1.93948616642881	1.16811146551875
N	2.87331891964958	-0.38362406323449	0.97882677401014
H	1.85967935261068	-1.33040654800570	-1.78504785638309
C	4.22683173460669	0.01583459281245	1.32007444332627
H	4.93214923433141	-0.78926001866878	1.09659063349493
H	4.49596588111273	0.89953508093633	0.74510022563250
H	4.27320263245586	0.24337646404682	2.38821612216879

TS5A-E-NH_3

Imaginary frequency: - 986.57 cm^{-1}

C	-3.28359832046028	-0.29136676482574	1.72342512879634
C	-2.23763666595506	0.26054600832311	2.44462690436781
C	-1.04405681263784	0.54016713236590	1.79019766756919
C	-0.88825816762355	0.27242853834676	0.43378609080881
C	-1.95654349595143	-0.27992455445178	-0.26246848996356
C	-3.15820222744021	-0.56567146693323	0.37027052730829
H	-2.35238767838791	0.46507833205948	3.50245500146168
H	-0.22619718940031	0.96697476150014	2.35900607804065

H	-1.82927281157354	-0.49726977466248	-1.31556506564659
H	-3.98647958827859	-1.00096153385230	-0.17635941851306
Cl	-4.78208654233300	-0.65306528309830	2.53289429093665
C	0.41800696725591	0.58237405264052	-0.32482368692158
C	1.62847033826815	0.55298523517596	0.61874788234577
C	2.35361050527777	-0.62548550595957	0.99845739685313
C	1.95242061386336	-1.96538718863385	0.36663224805647
O	0.55042191797990	-0.05890439325891	-1.46080625310727
O	3.30954713363077	-0.66163975999870	1.76661940935860
O	2.64843204378308	-2.53781994714519	-0.43725266419468
O	0.83562379335289	-2.43473622099613	0.90274601897990
C	0.40250951320538	-3.75966088114446	0.48960155752266
H	-0.20646482833314	-4.10877086776437	1.32251608844182
H	1.28333462577158	-4.39499795162799	0.39475887948672
C	-0.39401802602712	-3.70406794791226	-0.79653629812048
H	0.21468734882771	-3.32605413997844	-1.61934302411829
H	-0.73135459198294	-4.71277097706974	-1.04797785857505
H	-1.26957778718426	-3.06314130321308	-0.68017143117288
C	1.99264559147674	1.79709308292560	0.99197579499259
H	2.82538790737680	2.08854543055024	1.61938157159315
N	1.17725302123234	2.76161479974798	0.48655430542437
H	1.58663177366791	3.66172857563870	0.26306766629062
N	0.42729089034046	2.18187804697401	-0.58012631262151
C	-0.86054451633329	2.84009965342395	-0.76364809402179
H	1.16663051344687	2.26081315150561	-1.70518992272599
H	-0.69310875917235	3.86643434801431	-1.09932379931670
H	-1.40139967988265	2.30022653958804	-1.54197490214779
H	-1.44889429800753	2.84641759674576	0.15566291683127
N	1.85987768124901	2.15741734991702	-2.72402152637476
H	1.84382674984696	1.14697785731283	-2.85754563961024
H	1.45679800839960	2.61640027046281	-3.53490891501583
H	2.81667504871184	2.47549569930776	-2.60534012329843

TS5A-E-AcOH

Imaginary frequency: - 155.46 cm^{-1}

C	-2.48617771593023	0.15204080732126	1.79222683061524

C	-1.31196225404351	0.74216011259489	2.23159148213725
C	-0.34188679722987	1.08232617218292	1.29877041967425
C	-0.54139365651792	0.83491701241728	-0.05458002289551
C	-1.72852322786089	0.24308830944533	-0.47098554877943
C	-2.70813038894984	-0.10306020498219	0.44707827710650
H	-1.15655460024933	0.92952107075527	3.28697654968227
H	0.57953939352533	1.53799866262581	1.64138446733278
H	-1.88038182558658	0.04677617517062	-1.52480159680393
H	-3.63223818714286	-0.56778902143432	0.12550212497293
Cl	-3.70526208235676	-0.28117721667455	2.95167917155631
C	0.49881501478524	1.20919988778666	-1.09159949059311
C	1.90328685752754	1.42688348808908	-0.54979906931789
C	2.87849049722272	0.37167571615058	-0.38742744672930
C	2.49257220039191	-1.04968310552408	-0.82670900592876
O	0.37499480139538	0.65133248404301	-2.24394332800266
O	4.01201448988331	0.52646312897572	0.03898262803567
O	3.06820704332823	-1.62869975440715	-1.71459883556751
O	1.52812747324968	-1.54011536022004	-0.06694270014755
C	1.10464564371884	-2.90870019139051	-0.32534524549088
H	0.64871489513476	-3.22230706657309	0.61252313062418
H	1.99316564768790	-3.51086527940743	-0.51619062318358
C	0.12063131167135	-2.97035251357538	-1.47370843390207
H	0.57859770377278	-2.62773784239901	-2.40272913199185
H	-0.20235150743702	-4.00587424215491	-1.60646881644099
H	-0.75673771846550	-2.35685925002111	-1.26288165014769
C	2.20761586225089	2.71564353107974	-0.30183888521984
H	3.15214651751179	3.08945467013685	0.07234178033879
N	1.20955469152805	3.60875402442260	-0.53911307698478
H	1.44813288832519	4.55459923689308	-0.81371158965800
N	0.21126929501836	2.98430669717547	-1.32159837414889
C	-1.13278106337119	3.50944083046271	-1.07779198877632
H	0.48159415910119	3.07582356375720	-2.31410052427172
H	-1.17059784137679	4.56350096340471	-1.35828566401158
H	-1.82713372870721	2.94093064215866	-1.69616795197478
H	-1.38192885387862	3.39052143054145	-0.02533448156720
O	1.66960814728149	3.22989387327780	-3.74901276789803

C	2.33070569821112	2.31964371470958	-4.24111567292084
O	2.17079329152915	1.05718926537238	-3.92871133433782
H	1.41909493854432	0.92286961983069	-3.20582130342344
C	3.41060636313967	2.56000532742416	-5.26013156656152
H	4.36806327652827	2.23562113222735	-4.84587210107577
H	3.45829716740708	3.61530087577004	-5.52067349945535
H	3.21345617943232	1.95933862256042	-6.15016513386691

TS5A-Z-NH_3

Imaginary frequency: - 1024.50 cm^{-1}

C	-4.85323737677001	-1.40837948388982	-0.55146526361298
C	-4.02172968817365	-1.23096543915545	0.54228175730323
C	-2.66195584120559	-1.04305620474697	0.32997514659799
C	-2.14149129518303	-1.04886889355953	-0.96081723201726
C	-2.99459917896920	-1.25309637644575	-2.04329877440892
C	-4.35645672475286	-1.42073135497668	-1.84728156957179
H	-4.42977854473614	-1.24026166200474	1.54551446185151
H	-2.00441138601684	-0.91408861851980	1.18181864208890
H	-2.58854732091701	-1.27264945523306	-3.04842484272464
H	-5.02606266398152	-1.56333857723751	-2.68665724249279
Cl	-6.55736215684406	-1.62776378125321	-0.29536416913815
C	-0.66859483862879	-0.90540756919115	-1.22383230319379
C	0.04071683392426	0.20844531439349	-0.63878804458524
C	1.55325850531551	0.40052425489544	-0.80230575427242
C	2.30995488017816	-0.76463676219515	-0.07847619099666
O	-0.10865629744025	-1.74593394087750	-1.92481606909042
O	2.00604939856488	0.64488352620756	-1.99472833992641
O	3.36213340581718	-1.22577672601299	-0.45027696768610
O	1.65046770920059	-1.19872689159817	1.00011492680788
C	2.22508965751316	-2.30275276059858	1.73639932174537
H	1.82062386565828	-2.19813221158576	2.74303827371779
H	3.30803879743971	-2.18027325702517	1.76886428790733
C	1.82621458637400	-3.62609123823345	1.11540191000923
H	0.73823273236950	-3.71797291852931	1.07625194827693
H	2.22522473980142	-3.71825332187519	0.10370029100787
H	2.22146400727250	-4.44574838702720	1.72069078713590

C	-0.48873672735956	1.18297036988713	0.12352036439115
H	-1.53077989292214	1.39034425231454	0.32839641783269
N	1.72220568392623	1.69818055186991	0.14363095665942
H	1.99606376609542	2.65657123115282	-0.73625087126310
C	2.77222730988756	1.68624093855689	1.16345124992319
H	2.86924672543626	2.68945898067291	1.58381602984855
H	2.55033264233957	0.97825961545685	1.96339685095802
H	3.71181461310694	1.42093279214802	0.67599094090875
N	0.44803666782089	1.98032995891684	0.71537034334785
H	0.24719905341182	2.97124317011557	0.79536242071136
N	2.23434618963946	3.50219082372941	-1.62848670520395
H	3.13619656853291	3.95497591831273	-1.51524485074558
H	2.24947306210728	2.92716193530832	-2.46762240903426
H	1.51910240254129	4.21727745612532	-1.71850871694654

TS5A-Z-AcOH

Imaginary frequency: - 195.13 cm^{-1}

C	-2.33230183228556	-2.10181807438344	0.05156370343374
C	-2.18714115916162	-1.67761682293553	1.36626089153713
C	-1.78239469513300	-0.37450097084496	1.60742977564426
C	-1.49808063345034	0.48751812398132	0.54881795593991
C	-1.65774158959995	0.04313708099913	-0.76090402922502
C	-2.08724150325972	-1.25340443108327	-1.01546690810243
H	-2.38551191177225	-2.35938536079786	2.18429605864219
H	-1.66569500553533	-0.02525237947607	2.62691778516433
H	-1.45214609481624	0.70666654918618	-1.59055056738741
H	-2.21844407048639	-1.60286908539572	-2.03223365009602
Cl	-2.82060308481530	-3.73881651001942	-0.26106924579431
C	-1.04431314973326	1.88160877095258	0.87151837398126
C	0.11761011022466	2.42529144039602	0.17501664099335
C	0.95683751421712	1.70083992381253	-0.85612204710301
C	1.58331297446772	0.38411393479005	-0.33154558226743
O	-1.62544849303798	2.51201531395506	1.74947060419925
O	0.62114543046141	1.76758991385087	-2.07459500489511

O	1.70946256779721	0.14358024066554	0.84545457493229
O	1.93988716758379	-0.41666110312465	-1.32008226008992
C	2.47641133795597	-1.71628373216479	-0.95564132388488
H	3.15068625022914	-1.58921577907490	-0.10843434831702
H	3.04769671103688	-2.02168864026533	-1.83112301773276
C	1.35700085007868	-2.68982251837457	-0.64992791878862
H	0.77703819490421	-2.36371575510331	0.21507057190065
H	0.68669461194165	-2.78707685438511	-1.50647264399785
H	1.78612088838745	-3.67068883075675	-0.43116611798023
C	0.57660453641173	3.66765294297729	0.42825928618549
H	0.09649154571663	4.40578118606321	1.05778485029973
N	1.74095236963266	4.00687527197222	-0.18329420115889
H	2.32789742364284	4.74264958958602	0.18682624704433
N	2.40185793117919	2.86206279775398	-0.67013264262985
C	3.57028250111538	2.49429883270119	0.14195039378431
H	2.66915597717057	2.98160289338203	-1.65567137311633
H	4.03419441501252	1.61599032563862	-0.30693157068962
H	4.28625204501614	3.31908844838925	0.14717900767172
H	3.24368360472025	2.27010091267922	1.15590860549577
O	3.45961373868911	2.69217581086491	-3.35167281693908
C	3.14880468517174	1.84815624431529	-4.18213296136016
O	2.07418953874460	1.09362764437880	-4.08223298495577
H	1.54689512213245	1.31248587498577	-3.23809503133804
C	3.96093996349212	1.56741197476207	-5.41499903282811
H	4.30466818583113	0.53092555113288	-5.38798484166570
H	4.81379202893890	2.24081242054800	-5.46624120282842
H	3.33238300118259	1.68615683346528	-6.29980200167711

TS5B-E-NH_3

Imaginary frequency: - 1161.22 cm^{-1}

C	-3.41243026355794	-0.21994815318313	1.32369620316286
C	-2.45185784867357	0.30650022190322	2.17350385748639
C	-1.18179301708044	0.56168089498290	1.67357479890639
C	-0.86663104611235	0.29070961846446	0.34531001832531
C	-1.85036534196105	-0.23104173678381	-0.48435296943184
C	-3.12798747424358	-0.49015814030308	-0.00539692308373

H	-2.69286121296017	0.50901495008600	3.21028146671077
H	-0.42611248940244	0.96776803381555	2.33720464328477
H	-1.59721814603107	-0.44988935430037	-1.51424898682727
H	-3.89257513743735	-0.90323401145724	-0.65272123244856
Cl	-5.00657679253419	-0.55118728490070	1.94021427744484
C	0.53096472841288	0.57641691838310	-0.23556777984077
C	1.64454769033192	0.52893230332703	0.81760185362699
C	2.28910005386323	-0.64902357590917	1.28635535155375
C	1.87839736936028	-1.99758025778088	0.68046179542470
O	0.78978874943810	-0.00947729864144	-1.36708804601150
O	3.19600848795519	-0.70022655962833	2.12122309800040
O	2.62341790608575	-2.64961761183154	-0.01241051677045
O	0.67869892674914	-2.38009307830520	1.09548493097780
C	0.22220252669944	-3.69679908993312	0.68368433334080
H	-0.51801155067866	-3.96600897209227	1.43619962368254
H	1.06454719216489	-4.38653098776008	0.74275276460053
C	-0.38281340717948	-3.66429902219872	-0.70376530376422
H	0.35607827480163	-3.36141553193973	-1.44724316123074
H	-0.74099166936855	-4.66518184115155	-0.95784185397676
H	-1.22654094452128	-2.97353114668988	-0.74032084196439
C	2.10017305844919	1.77607889943416	1.12361464306556
H	2.93745563343141	2.02798120704596	1.76250161050644
N	1.43428083921064	2.76148289399497	0.52110070754431
C	1.87859047736807	4.13291315135030	0.36474112708389
N	0.50453556158730	2.23700144933593	-0.40375882352415
H	-0.41891253164564	2.59917464746069	-0.17831450168806
H	0.85951025168312	2.44164295496136	-1.63446417664570
N	1.25364932452364	2.40447918953455	-2.85765721999215
H	1.33946501094140	1.39756556712781	-2.99132641667304
H	0.57959159412097	2.77903251862448	-3.51782210908319
H	2.15223790563750	2.84838688376475	-3.01959684504896
H	2.59923430179054	4.34702426141214	1.15314498297582
H	1.02648105972876	4.80758691444490	0.46270152624969
H	2.34908123119826	4.28055300468756	-0.61138808404401

TS5B-E-AcOH

Imaginary frequency: - 186.26 cm^{-1}

C	-1.36706397319187	2.77311420710181	2.50859553449585
C	-2.35496123766626	1.80629133073377	2.41200229772842
C	-2.01984183621575	0.54840042251735	1.93040525861924
C	-0.71557153749325	0.25936991002647	1.54929841965330
C	0.25778915407872	1.25047831433831	1.64774474599159
C	-0.05827360412134	2.51040678482590	2.13195698066881
H	-3.37301514293184	2.03551276361905	2.70229791674268
H	-2.78660076185973	-0.20881435634604	1.83277339653519
H	1.27676436628775	1.04483668985696	1.33706561791451
H	0.69794521652324	3.28182302743492	2.20911632951296
Cl	-1.77756974899349	4.35166410884966	3.10461933453790
C	-0.38545284864274	-1.11178633137855	0.98001489866692
C	0.60444598117208	-1.12435675486071	-0.17872142313352
C	0.44859857374028	-0.44990361237709	-1.43421556728745
C	-0.68839258905203	0.57617361728886	-1.56746201289830
O	-1.32986916592762	-1.97976887768052	0.91440570822973
O	1.19219219428997	-0.57020189690516	-2.40267620288265
O	-0.47509899473402	1.74511678796161	-1.78374790149940
O	-1.88133722867155	0.02077694533917	-1.44831601498303
C	-3.02513568636974	0.91371198554188	-1.52068767651661
H	-3.02918002011521	1.38339769703989	-2.50576938630573
H	-2.89546248155576	1.68669272725258	-0.76102909947263
C	-4.26201945923782	0.08194575573032	-1.27879186617172
H	-4.22751501376601	-0.38561712262035	-0.29262832316805
H	-5.14219833010081	0.72700803945715	-1.32676087719495
H	-4.36127766276543	-0.69738693396596	-2.03734533369818
C	1.69619950590709	-1.89133455237329	0.08675899026781
H	2.48262600172618	-2.14585555727445	-0.61246124073850
N	0.80668645362736	-1.76273965847137	2.15075556938466
H	1.22990308255934	-1.05973702126948	2.75866334152121
H	0.28435419257911	-2.45643540844571	2.71465832665342
N	1.78384395940675	-2.35776724488725	1.33757528867465
C	2.91970185649411	-3.01707759037289	1.95598233954997
H	2.57086086725410	-3.85336906614816	2.56317012695692
H	3.56434502773752	-3.38901222418955	1.16126590962504

H	3.47442141042297	-2.31243673196341	2.58201104544532
O	-0.80346097471997	-3.44274131936125	3.77588756090907
C	-2.02665874414834	-3.33975978221874	3.79364585106450
O	-2.71600987565490	-2.73354206291364	2.85739768073626
H	-2.10698812538094	-2.37946478199948	2.08667905641105
C	-2.87427417291427	-3.89800381980221	4.90285928224594
H	-3.60959621329474	-4.58886905225697	4.48553301133708
H	-3.41989887863347	-3.08135849202690	5.38093445206729
H	-2.25225353564713	-4.40848086280604	5.63503865380271

TS5B-Z-NH_3

Imaginary frequency: - 1111.04 cm^{-1}

C	-4.86755753295992	-1.68150000940451	-0.05979555296793
C	-4.03461995293326	-1.15661448343456	0.91465932180854
C	-2.70191012591354	-0.91912851011115	0.60339798613328
C	-2.20724282540401	-1.21694191008645	-0.66327970671518
C	-3.06031219907688	-1.76548635875089	-1.61902011654424
C	-4.39688537768443	-1.98923424020416	-1.32877578793820
H	-4.42004317080175	-0.93966622959489	1.90335987480336
H	-2.04190078561473	-0.52253885471518	1.36576092066711
H	-2.67319751960852	-2.01146410294879	-2.60128064336866
H	-5.06698011709901	-2.40078389978619	-2.07365933868561
Cl	-6.53873526950947	-1.96956833176593	0.31704993186334
C	-0.75937970695946	-1.02415269411697	-1.01700066019742
C	-0.11431793392156	0.20858672107273	-0.66272216385516
C	1.37174002949611	0.49642826449616	-0.94186755886359
C	2.27879608247675	-0.57875320001968	-0.26195752050036
O	-0.15939279293782	-1.94019825661628	-1.58433596402229
O	1.68134425608161	0.75944858779028	-2.17564515209326

O	3.16574507292993	-1.16985450193943	-0.82583022095331
O	1.94615685196556	-0.78012698979042	1.01933745324454
C	2.65257285035510	-1.81888213456704	1.73796523581468
H	2.55767794134363	-1.53690829066984	2.78628303236157
H	3.70415531915901	-1.79306889542073	1.45148526584228
C	2.02829577263552	-3.17295476059492	1.46906810128238
H	0.97155851205283	-3.16945108975188	1.74629991293405
H	2.11598121325711	-3.44052679748843	0.41457121649692
H	2.54033446987663	-3.93347039933578	2.06390642223188
C	-0.69110307142677	1.33761394719574	-0.18688920981004
H	-1.74126097908128	1.55071560340969	-0.03482139860479
N	1.51541267664654	1.78823965930863	-0.01232038387102
H	2.31982782192156	2.58747056808928	-0.62692072346904
H	1.79773370640734	1.52676217468242	0.93449976741065
N	0.20346533862412	2.32262818561089	0.05191173667878
C	-0.00948311956612	3.46813886114726	0.91208416000677
H	-1.05943119508502	3.75167633373976	0.84614885554230
H	0.23905436366636	3.23096425577975	1.95283285398585
H	0.60899309390480	4.29987815188308	0.57268173011584
N	3.06658105949620	3.28325918619406	-1.41518400050720
H	4.02712052413467	3.36558791498703	-1.09651319782517
H	3.04542839039131	2.73971276054215	-2.27527690213791
H	2.70577832876088	4.21416376518523	-1.60020757629363

TS5B-Z-AcOH

Imaginary frequency: - 191.17 cm^{-1}

C	-2.45766665510802	-2.23169289243814	-0.04934864607416
C	-2.27128727130918	-1.84104666977504	1.26928890279810
C	-1.85908685336597	-0.54446874676521	1.52975196987471
C	-1.60855095235542	0.34400863243670	0.48517665374098
C	-1.80494133387366	-0.06908824008732	-0.82898428736322
C	-2.24288456658080	-1.35854481850073	-1.10181616522379
H	-2.44487154658447	-2.54269955940589	2.07591621090750
H	-1.71539430405574	-0.21961242298124	2.55385704520572
H	-1.62492884122234	0.61543120383193	-1.64741301781184
H	-2.40463298839916	-1.68325122725384	-2.12229801920742

Cl	-2.95776044455784	-3.85969009084935	-0.38686919593512
C	-1.16458617528294	1.73714893542514	0.83087964548983
C	0.01844518532780	2.26905014906361	0.18669363525845
C	0.85007969928691	1.55055940302257	-0.85415310974445
C	1.51703560428512	0.24286537602077	-0.34942804482683
O	-1.77997105426936	2.35750911698265	1.69776808457157
O	0.53475879188651	1.64424310351058	-2.06669154789420
O	1.85486740380223	0.08172150482790	0.79956198486225
O	1.65406361705258	-0.63994448257796	-1.32171746226548
C	2.22197999280781	-1.92907739709381	-0.96848188179147
H	3.02397268647703	-1.76980635527806	-0.24778119322711
H	2.64177300794069	-2.29975225469542	-1.90268733982752
C	1.15011476484981	-2.85344893168884	-0.43054496386539
H	0.70294173279120	-2.44834009191534	0.47900266167323
H	0.36292096431563	-3.00408257959898	-1.17162529547818
H	1.59744788240138	-3.82254245873491	-0.19641951821911
C	0.51179722878884	3.49085208613211	0.51252647191196
H	0.01467550273023	4.21090615921356	1.15017347955490
N	1.70656095948431	3.82170537783530	-0.00148613227299
C	2.52110166334425	4.96280323167470	0.37553616796347
N	2.32713843788085	2.70574546553183	-0.57707680392183
H	3.06192877748123	2.35004560642845	0.03523055645877
H	2.69435690023053	2.89110852620311	-1.51906370985627
O	3.37035120622890	2.69699917206705	-3.28764631931369
C	3.12108196195282	1.83446828931314	-4.11831337904333
O	2.08804304254231	1.01901794731548	-4.03239148189394
H	1.52368156044550	1.22043418089077	-3.21335341833441
C	3.96310059678281	1.59567849818422	-5.33980383127965
H	4.33098500425371	0.56746471049766	-5.32817185751483
H	4.79884755059999	2.29163659057657	-5.36284116087901
H	3.34698168222361	1.71809484183076	-6.23306413667851
H	3.28161205990014	4.66772298066349	1.10481968781161
H	1.86734172543169	5.71631160156288	0.81202475937148
H	3.00467579343801	5.37455652859685	-0.51133599771033

IIA

C	-2.71194356538283	-0.36914383354071	0.94838274030310
C	-1.58946783138297	0.16771120770507	1.56440012399815
C	-0.53560337182090	0.64231263543476	0.79220753085166
C	-0.60819479363107	0.59225291660008	-0.59841576136559
C	-1.74215075986095	0.05583356889662	-1.21245724698691
C	-2.78697173931858	-0.42790802397946	-0.44247596620277
H	-1.53086679679907	0.21586975760697	2.64603095958375
H	0.33646534331487	1.06962865752608	1.27338188363626
H	-1.78738776355090	0.01656846179936	-2.29443220595742
H	-3.66225863013472	-0.85290698253570	-0.92136784228185
H	-3.53199886450057	-0.74630628632209	1.55013651465337
C	0.49604689331476	1.13274063366339	-1.44471678319400
C	1.83325825863217	1.33971706037808	-0.81139929930725
C	2.55705297486484	0.21749136678584	-0.26476761033470
C	1.92669115016290	-1.17224472273492	-0.46666783696987
O	0.30491324915335	1.36309205500276	-2.63034783277697
O	3.65222750508136	0.26117865789681	0.28514580910668
O	1.52078117405663	-1.54394639589971	-1.54321490412858
O	1.94344730486435	-1.88710409798905	0.64432193769612
C	1.37518394718886	-3.22436499110781	0.57493721919083
H	1.85477083080933	-3.75928468239331	1.39343030037916
H	1.66672586583032	-3.67544133440083	-0.37332881866847
C	-0.12867766847648	-3.17208485601450	0.74238221830727
H	-0.59286278315759	-2.58806324580092	-0.05426241578016
H	-0.52838640388906	-4.18852571295090	0.70779254241823
H	-0.39393778849349	-2.72617355307492	1.70261949597291
C	2.47148892432639	2.55805319402081	-0.73208576966180
H	3.50609313261507	2.56083766423514	-0.40190820075307
N	1.98586586687730	3.76071303430515	-0.97393822766192
H	2.60894209247740	4.55549241546899	-0.91476866396680
N	0.64072986966465	3.96567413969684	-1.26876265170119
C	-0.00244453579134	4.99923511757015	-0.42483650331399
H	0.57016625688261	4.21639788105447	-2.25183218998537
C	0.68127221790856	6.36458808152958	-0.55956847988264
C	-1.43995551194878	5.08384145826853	-0.93335611149064
C	0.01727965511340	4.52954689937504	1.02737411180657

H	0.71047032423866	6.67734806082107	-1.60726323445007
H	1.70246096331454	6.34890232471082	-0.16868036058270
H	0.12766037183758	7.11532121613905	0.00907436548708
H	1.04076986880138	4.41909232774487	1.39373636664627
H	-0.49151187642473	5.26199657568758	1.65805333756029
H	-0.49308362231684	3.56883432908061	1.12407103006744
H	-1.93540235700905	4.11441017297556	-0.83834613290438
H	-1.46194290859747	5.38634217785608	-1.98461860593092
H	-1.99729635516596	5.82242058026794	-0.35359069330926
O	1.73823456683794	3.20336203110161	-4.17495976214447
C	3.11629578975555	2.89314871206927	-4.32919305016508
H	1.32005646592506	2.48492265236937	-3.66864748350699
H	3.57862685147036	3.70326861378869	-4.89624450961446
H	3.62537035619871	2.81284311259009	-3.36169457651643
H	3.26029785613441	1.95610896672151	-4.87942875616830

IIB

C	-2.57517347230627	-1.84250907508003	-0.25084788221386
C	-2.54261355901971	-1.22829688664410	0.99926219466066
C	-1.85663904074445	-0.03672133367505	1.16771658100431
C	-1.17801912129307	0.54000406268171	0.09420127734673
C	-1.20911849386613	-0.07915759162900	-1.15281487408133
C	-1.91466268741264	-1.26524514841212	-1.32595847806868
H	-3.05460492492966	-1.68218791118937	1.84085764632485
H	-1.83140983503327	0.45352521990994	2.13423057779032
H	-0.71098587454849	0.37619781779787	-2.00179099883240
H	-1.94628534217629	-1.73510228104543	-2.30266788793093
H	-3.11638999965151	-2.77324899452901	-0.38300753194018
C	-0.49589596374800	1.86096265233830	0.28872524340467
C	0.76345273279059	2.11694227788000	-0.42045511467053
C	1.56750466926922	0.99824101119298	-0.89833964486207
C	1.71014240183432	-0.19523207463339	0.07637160782605
O	-1.03940731564241	2.71054166056038	0.98960982302988
O	2.16629143679103	0.93262045213415	-1.95636452476393
O	1.69081484538546	-0.04611788156253	1.27492246350956
O	1.92518815170683	-1.33010928693194	-0.56093932552747

C	2.13347230835282	-2.51440889393874	0.25510825133834
H	2.75541382590489	-2.24118221075065	1.10767958131800
H	2.68676160888070	-3.18991883705956	-0.39576009450321
C	0.80983294962565	-3.10956685238227	0.68537251787076
H	0.25063164335403	-2.41222989862627	1.31070634946411
H	0.20145995509488	-3.36494224139602	-0.18415332075341
H	0.99745646074274	-4.02008748028255	1.25974436407824
C	1.06013029167009	3.45634825282727	-0.59751936751957
H	0.26998700183006	4.15493746400839	-0.33752530395322
N	2.16241836470146	4.06395863232804	-0.98944903762531
H	2.13588587640328	5.07258015699217	-1.06734454227537
N	3.37636116980898	3.40961514944240	-1.17478624001195
C	4.40579741114765	3.77148720764308	-0.16880498123661
H	3.70695023601237	3.63122563922142	-2.10883350130264
C	5.66119586294121	3.01811496908297	-0.60166952569183
C	4.68357722251031	5.27798540862521	-0.13370389665281
C	3.94118589032930	3.28092430576985	1.20028209106379
H	5.98337906121051	3.34034817934161	-1.59670365634949
H	6.47391247018089	3.21515715610988	0.10031250370519
H	5.47172639387453	1.94245427920118	-0.62733599791745
H	3.75677174056829	2.20424920217108	1.17863241439504
H	4.71043060588851	3.49076744576197	1.94688579137246
H	3.80974259828763	5.84157973163765	0.20462159195176
H	5.49964212208791	5.48969385615110	0.56081230373699
H	4.97276565738173	5.63918809834651	-1.12521574841506
H	3.02317843116738	3.78546818732284	1.51096570642212
O	1.91307526861681	2.86619002675119	-4.00053735034833
C	0.51169496591621	3.07163448352766	-4.11785633603922
H	2.05606148356600	2.23824718164695	-3.27249471157021
H	0.10897612439578	3.62377052435572	-3.26028417066509
H	-0.02932360992067	2.12243808452588	-4.20607857125391
H	0.33416000006207	3.65826610248032	-5.02117826463760

IIA-NO_2

C	-2.69760762139783	-0.51771774413673	0.72861470501258
C	-1.65356495982945	0.06514150382538	1.42246662011103

C	-0.59487282213316	0.59107845462441	0.69493595355839
C	-0.60233873353779	0.53512796003437	-0.69573135531526
C	-1.67594164799882	-0.04998737956249	-1.36765322166231
C	-2.73369395280407	-0.58803196776071	-0.65724887918675
H	-1.66438990985199	0.10350005874293	2.50276176740662
H	0.23122377443271	1.05718839545443	1.21761177867302
H	-1.66823745829669	-0.09005586383412	-2.44958586566448
H	-3.56809565539406	-1.05704200833938	-1.16001048542708
N	-3.81749829350463	-1.09926761323608	1.49525285128433
C	0.52776643577662	1.12036553693910	-1.49233665380610
C	1.83141553621146	1.33615830845538	-0.80494978059502
C	2.54227342166072	0.22273697138505	-0.22211535059374
C	1.93123486297178	-1.17414980804223	-0.43980128585240
O	0.35956947721053	1.37565160716420	-2.67350953084444
O	3.61342287258428	0.28242296955309	0.36979460778069
O	1.53066223692399	-1.53817129843433	-1.52117127552998
O	1.95106431470680	-1.89829517919768	0.66384380891899
C	1.41017705206086	-3.24659704360195	0.57893546783198
H	1.88416184925867	-3.77336028499295	1.40578637821545
H	1.73057495579154	-3.68841687388696	-0.36444583570386
C	-0.09763101246623	-3.22910665030084	0.71560079846001
H	-0.55956317376691	-2.67316755379151	-0.10197283052271
H	-0.47029350178090	-4.25592041801697	0.68982685287130
H	-0.39385894318863	-2.77642940661057	1.66384602847551
C	2.45658353858374	2.56212347551980	-0.70569053258319
H	3.48094852510291	2.57425914875155	-0.34528289004368
N	1.96411764986809	3.75824015859739	-0.95591633678061
H	2.57578361509659	4.56017046930481	-0.87512389947998
N	0.62342873057827	3.94999010025272	-1.27956410876313
C	-0.04749391494222	4.97484679309580	-0.44574321302742
H	0.57345586291999	4.20527455033040	-2.26293740254102
C	0.62930807593719	6.34583982440372	-0.55576430544155
C	-1.47240571823237	5.05239522606156	-0.98963854775089
C	-0.06050160356437	4.49629831033928	1.00356313837150
H	0.68481935324391	6.66372821333437	-1.60083434866947
H	1.63926592606266	6.33729366756963	-0.13645941407230

H	0.05379519823872	7.08924168451091	0.00053206317231
H	0.95417170940185	4.38818678478078	1.39435993608057
H	-0.58793926343038	5.22239321785411	1.62615533347044
H	-0.56917787290199	3.53303232968346	1.08196525495765
H	-1.96314703567466	4.07894714075279	-0.91340288534474
H	-1.47041673392273	5.36216685175637	-2.03896862921299
H	-2.04925458434986	5.78282453327731	-0.41877949663430
O	-3.76887120690253	-1.04231064475057	2.70772919751712
O	-4.72996906465850	-1.60706539576868	0.87480123816809
O	1.80684500275233	3.27862537515898	-4.16137324825812
C	3.19458863779966	3.00050851651735	-4.29361672168589
H	1.39094329683601	2.53361628401694	-3.69572205817104
H	3.65711983044788	3.85136708260581	-4.79704677187084
H	3.67702303126215	2.87005283476209	-3.31813852126613
H	3.37031991080881	2.10119879484819	-4.89494809803605

IIB-NO_2

C	-2.50524112420880	-1.84699396751148	-0.14651981060788
C	-2.47046578461656	-1.23120688056424	1.09704194945282
C	-1.81099029641105	-0.02090834036641	1.21136403909301
C	-1.18119624876182	0.54519105577765	0.10325400919840
C	-1.23512046494096	-0.09366795555109	-1.13255842868251
C	-1.90946166749029	-1.30014536883371	-1.26758916842102
H	-2.94656272067277	-1.69361813464022	1.95061114756326
H	-1.76899841742632	0.48784052569689	2.16676121774665
H	-0.77269525859238	0.35448543082780	-2.00434572868221
H	-1.96594284421268	-1.80509604084779	-2.22191452687993
N	-3.19435699172481	-3.14449487359213	-0.27453085397270
C	-0.51379434034950	1.88684049121923	0.25406467418994
C	0.75021543829569	2.12895428376688	-0.44218660362773
C	1.55356850604212	1.00662745137868	-0.91809211207916
C	1.69227835183340	-0.19190490712939	0.05300479972383
O	-1.09195883652454	2.74742835669505	0.91007078855115
O	2.15133323698977	0.94372704314362	-1.97592503822658
O	1.64870179035011	-0.05014249505240	1.25167708086863
O	1.92412841564367	-1.32020528846566	-0.58898305376737

C	2.13551313654223	-2.51047676082089	0.21892370300197
H	2.73849573956814	-2.23697628468924	1.08481221129439
H	2.70978482210506	-3.17061205738069	-0.42949860102673
C	0.81437906949159	-3.13045527403065	0.62083626392405
H	0.23708246397314	-2.45032361936207	1.24886146741415
H	0.22406375723225	-3.38671525377195	-0.26116523989075
H	1.00774701072211	-4.04516235875162	1.18645412467019
C	1.05255394989662	3.46912012878493	-0.62836958255419
H	0.26078785811956	4.17275386755533	-0.38716415196219
N	2.16174151375594	4.06608603181726	-1.00949249442511
H	2.14123291916396	5.07412075524048	-1.09998532606641
N	3.37453974175592	3.40349075252352	-1.17470945898006
C	4.39109141252518	3.76630815830872	-0.15496505285189
H	3.71935350475459	3.62125441436924	-2.10485169058533
C	5.65219229158332	3.01400839084704	-0.57279479892350
C	4.66678895552428	5.27298914253487	-0.11589227904682
C	3.90894811930611	3.27486005391525	1.20775648887967
H	5.98598911952103	3.33668147957031	-1.56364929829760
H	6.45595116464824	3.21232404291777	0.13913514127739
H	5.46454907518415	1.93799902701065	-0.60020180763839
H	3.72750217985656	2.19784492848303	1.18445470199963
H	4.66738425387784	3.48733777023724	1.96461468948793
H	3.78929559797870	5.83535261857166	0.21489977675893
H	5.47580272041785	5.48424767519369	0.58710512758972
H	4.96546436427891	5.63546967674274	-1.10392016197552
H	2.98582076843158	3.77792460937253	1.50587100108703
O	-3.68412031085700	-3.63141730728071	0.72522120018282
O	-3.23519778335168	-3.66410073135338	-1.37214823411691
O	1.90709427259795	2.88221661456243	-4.02450210715728
C	0.50466291052928	3.07899249133501	-4.14562056649690
H	2.05084174747231	2.25136438656590	-3.29968747047117
H	0.09623817149822	3.62876783461058	-3.28915078008266
H	-0.03018662011687	2.12646691489348	-4.23530917160565
H	0.32607135879015	3.66504749552584	-5.04907200485324

TS-IIA

Imaginary frequency: -668.00 cm^{-1}

C	-2.82101811362770	-0.61735590482737	2.07991989544318
C	-1.77298808822237	0.10807251930086	2.63496384738629
C	-0.72181633240658	0.53382887907105	1.83509034111640
C	-0.71397487420685	0.25213364141190	0.47209783012138
C	-1.75236417645638	-0.49172801416536	-0.07412411431151
C	-2.80410156374134	-0.92229008628218	0.72400042034161
H	-1.77173198788270	0.34100843134397	3.69420189312464
H	0.08936300973776	1.09698811845708	2.28065553235589
H	-1.72853327108840	-0.74178341752432	-1.12637709172342
H	-3.60932994466002	-1.50224133214983	0.28603080940129
C	0.43167072289458	0.72551769678877	-0.40871806347045
C	1.75012441594662	0.75601970273148	0.35262070118917
C	2.56745217048555	-0.39288416172150	0.67391404383721
C	2.22743018356546	-1.74925903835941	0.04111748074649
O	0.47550439459297	0.06756459191476	-1.59500764118242
O	3.56588069698464	-0.35416476829162	1.37480132851440
O	2.89691053415097	-2.22272180200835	-0.84408188494172
O	1.20522803394046	-2.32741038998594	0.64701130157848
C	0.85823526061024	-3.67697056621404	0.22258148311981
H	0.32497120649353	-4.08956674697909	1.07768158429699
H	1.78318904084144	-4.23326047864213	0.06938684015579
C	-0.00498108155257	-3.66204689850736	-1.02045164589152
H	0.52275775193871	-3.20792217237402	-1.86035882421156
H	-0.25809452879822	-4.69174718729057	-1.28511998110735
H	-0.93009962627303	-3.11236577991818	-0.84205390741035
C	2.09062032280320	2.01379428639817	0.66948915251137
H	2.96574091892675	2.35448161419069	1.20661328846852
N	1.17743671395812	2.93806755089956	0.24237798402510
H	1.54393280173624	3.82734538378661	-0.07498998656815
N	0.34795293388473	2.29848852606698	-0.73471291648055
C	-0.94654412277662	3.04834558458696	-0.98064264615772
H	0.92261260831093	2.28065434366727	-1.69281390601303
C	-0.53763699066127	4.45324751913198	-1.44149036048159
C	-1.68159650153001	2.34902043130362	-2.12023701689550
C	-1.82265682541571	3.15592371687090	0.26373477286191

O	1.76688148956052	1.71926364375702	-2.76018634050402
C	1.42976370583429	1.97369153267362	-4.10168144092808
H	1.14356589986088	0.68975056058205	-2.25160778429583
H	-3.64223453677909	-0.95292508570178	2.70412737998577
H	0.56505596200222	1.37760087289303	-4.43079878980668
H	1.17656850783346	3.03188275560083	-4.25807092576247
H	2.26615096319196	1.73267760087549	-4.77192301636162
H	-1.95627001811050	1.32912836947181	-1.86346165412376
H	-1.08053402458243	2.33604283921268	-3.03033030363274
H	-2.59535707037817	2.91119030457265	-2.32109181443450
H	-2.33928857442320	2.22541235046209	0.48789338262595
H	-2.57365616186449	3.92497598393903	0.07097806900629
H	-1.23869881416043	3.46108290834825	1.13335170308309
H	-0.14729737555704	5.05645223009943	-0.61901297917762
H	-1.42448899670353	4.95739203688833	-1.82745234949700
H	0.20511986688267	4.40776573084923	-2.24261808710630

TS-IIB

Imaginary frequency: -931.05 cm^{-1}

C	-3.50470995776519	-2.26521903190417	-0.18536191763839
C	-3.57234054584637	-1.68269841026300	1.07721417111314
C	-2.99237660741614	-0.44346209467361	1.29895649359846
C	-2.31363534447936	0.20963889408203	0.26864076003348
C	-2.23814066371073	-0.37948357756319	-0.98985006147500
C	-2.84618927642441	-1.60954720910043	-1.21636801305641
H	-4.08207717270674	-2.19380505849531	1.88668044220002
H	-3.05223949942483	0.02388163290508	2.27543445724993
H	-1.71151735889130	0.12300016885125	-1.78919286536310
H	-2.79755510407132	-2.05790420097832	-2.20276361363701
H	-3.96503488456603	-3.23143308681238	-0.36217733645889
C	-1.74948005323691	1.56587344840797	0.56669429078498
C	-0.36278965926171	1.90379408658228	0.21477005656235
C	0.66910684186334	1.05062056134003	-0.51670801016310
C	0.89709176669379	-0.23395750482943	0.33009050602434
O	-2.44155422779468	2.37193235758390	1.17677360975000
O	0.45215380618943	0.77771337391563	-1.82193245901760

O	0.75669639452359	-0.23293777199892	1.52974757123260
O	1.21321052686484	-1.28882981192250	-0.39657611157165
C	1.39547608875742	-2.54373539773057	0.31334264937377
H	1.91955170517568	-2.34133748005140	1.24770029769086
H	2.03856400414532	-3.12721207885436	-0.34412906426197
C	0.06402516833404	-3.22554302206546	0.54510655206235
H	-0.58961938368439	-2.61129282815380	1.16642819424426
H	-0.43859671458259	-3.42139119912424	-0.40364105964396
H	0.23416506854607	-4.17814596984010	1.05279972745693
C	0.23626988922038	2.97062643894628	0.75910169128866
H	-0.21817833648851	3.74959628440939	1.35565799711563
N	1.59536737589378	2.99960480701063	0.57437993154867
H	2.01030305494457	3.90509438934318	0.38502322062864
N	1.90274446436059	2.03910086122309	-0.44092743643754
C	3.34555426259019	1.56860961396542	-0.38742261666231
H	1.75263909812336	2.55106255163137	-1.44806660794516
C	3.59499111682289	0.68751690253167	-1.60963156689941
C	4.20362991113310	2.83345042083762	-0.49825819377974
C	3.67687465220872	0.83828097602104	0.90969827245566
O	1.26613642646129	2.82016199639851	-2.75013544995917
H	0.71052847649808	1.73008121770774	-2.40822318395311
C	0.34848654923017	3.88887869844299	-2.85208689375709
H	0.83560234705065	4.84160798876415	-2.61149561450221
H	-0.05150584795953	3.96340200224222	-3.87097144436070
H	-0.50240747857268	3.76502036190509	-2.16684681302413
H	3.89237305452838	3.44957905528680	-1.34608610817802
H	5.23891165065123	2.52990930091385	-0.65820397436268
H	4.16856854455229	3.42969022565501	0.41550028904349
H	3.28246367112216	1.37150436115897	1.77563370389690
H	4.76398236372445	0.79610200653392	0.99879080866603
H	3.31015732437345	-0.18648732542364	0.91942242571297
H	2.97066362911293	-0.20355099049331	-1.60645827989054
H	4.64155628877203	0.37838583126064	-1.58721316746278
H	3.41684471617078	1.24138482343056	-2.53313624784177

TS-IIA-NO_2

Imaginary frequency: -812.26 cm^{-1}

C	-2.79441887603096	-0.64152579356510	2.03164523467391
C	-1.78095602545547	0.09647894565691	2.61970585636999
C	-0.73884292069332	0.53441065821884	1.82090750335603
C	-0.72244955458575	0.24949162079903	0.45855986695893
C	-1.74329010186683	-0.51051008723986	-0.09801335873119
C	-2.79335424174141	-0.96141390852450	0.68489872021925
H	-1.80675552386205	0.32025164299009	3.67706299751341
H	0.06038785519928	1.10848788661334	2.27163946713199
H	-1.70859965532424	-0.75979131967250	-1.14913920692236
H	-3.59234819658797	-1.55346938877126	0.26055845521116
C	0.43104320684072	0.72712165352309	-0.41656975078030
C	1.74266665656768	0.74985079915087	0.35694488598779
C	2.56362433398219	-0.39872893715406	0.67102412161391
C	2.21351010036991	-1.75958861362941	0.05223889622150
O	0.47778213815879	0.06438592905193	-1.59868052002167
O	3.56948823527889	-0.35604925385528	1.36008526687337
O	2.90801229157535	-2.27257764607616	-0.79008109654005
O	1.14748129514706	-2.29365319107547	0.62269097473522
C	0.78987150692036	-3.64833458014942	0.22285432874956
H	0.19812141811240	-4.01811197965727	1.05880454202141
H	1.70814940439697	-4.23052448911076	0.14297943700006
C	0.00133071783877	-3.65995588269817	-1.06890676584254
H	0.58380910534452	-3.23915198605081	-1.88958692995063
H	-0.24981272046136	-4.69441202816424	-1.31640624610087
H	-0.92597590980434	-3.09577222775412	-0.96336616177602
C	2.08233988571781	2.00803160113568	0.67629471354587
H	2.95617444997887	2.34783074345978	1.21605470321441
N	1.17146250864336	2.93105859086833	0.24637301832663
H	1.53636331005943	3.82298919352465	-0.06532870090490
N	0.34634863534660	2.29288004449219	-0.73599318632786
C	-0.94732421740408	3.04420524440357	-0.98653981125181
H	0.93343378310394	2.27394940889540	-1.70394396594979
C	-0.53411016966677	4.44925898364631	-1.44299810775617
C	-1.67921520620762	2.34817491783678	-2.13015276437769
C	-1.82949106807325	3.15323149076250	0.25384524575558

O	1.76123065453968	1.72640012842829	-2.74516224348280
C	1.44354607899377	1.99427331744392	-4.09089979567960
H	1.14859014734476	0.69506668157225	-2.25884791042925
N	-3.90816356683118	-1.11319929482422	2.87257883322934
H	0.57711112554333	1.40966727084768	-4.43329259054125
H	1.20553096383983	3.05618695311281	-4.23987143243597
H	2.28730216291045	1.74762728797866	-4.74843074050466
H	-1.95848512545040	1.32814630825377	-1.87837903174294
H	-1.07560351635190	2.33591865562434	-3.03838043910048
H	-2.59132958194103	2.91231130436677	-2.33258493988688
H	-2.36515935427033	2.23039335298295	0.46507112309244
H	-2.56819094268329	3.93456304823432	0.06397771038108
H	-1.24768934746075	3.44179267529982	1.13055858076371
H	-0.14986067295645	5.05136827277303	-0.61689859317665
H	-1.41819823345397	4.95391705361583	-1.83442167334209
H	0.21417418857343	4.40501852695600	-2.23883233821535
O	-4.78707848789789	-1.76590730121530	2.34458256067034
O	-3.89501881392908	-0.82533380623660	4.05335027537922

TS-IIB-NO_2

Imaginary frequency: -993.78 cm^{-1}

C	-3.38936046705310	-2.28168324562076	-0.14605758417061
C	-3.46965124718226	-1.71833998123996	1.11810284145258
C	-2.92557018809401	-0.46116906851945	1.30938864783561
C	-2.29401751973798	0.20122897078910	0.25654047128438
C	-2.22846479783329	-0.38716689441347	-1.00183890397743
C	-2.78960021810765	-1.63855436024529	-1.21266150915782
H	-3.94472097164570	-2.25330712041358	1.92859799570616
H	-2.97800265895264	0.00872961152809	2.28400205598377
H	-1.73682771449131	0.12574763997650	-1.81593828535667
H	-2.75295657009911	-2.10805955582177	-2.18581636111461
N	-3.95284218277012	-3.62686407566044	-0.35725425928627
C	-1.75195555373610	1.57642158262871	0.53523346380333
C	-0.36407676123272	1.90718397741245	0.20857837962656
C	0.66802894576648	1.05043641777668	-0.51807948561851
C	0.89403719994883	-0.23518782250898	0.32878888188978

O	-2.47672089071607	2.38222445188416	1.10263200680867
O	0.44548145270667	0.77536925245665	-1.82153039030540
O	0.73856448881938	-0.23817561654086	1.52663608593961
O	1.22144820542815	-1.28648394435574	-0.39762093262046
C	1.41192307393773	-2.54211467007205	0.30933592157046
H	1.92975936584892	-2.33770972193200	1.24673175256626
H	2.06343861406910	-3.11702476771862	-0.34727397422370
C	0.08703838856693	-3.23888702014967	0.53434732215375
H	-0.57056473552624	-2.63679566502952	1.16312224695189
H	-0.41284486579501	-3.43389217804866	-0.41634342031062
H	0.26665076004934	-4.19383837286621	1.03411735832337
C	0.23340459603246	2.97723394484235	0.75191407605091
H	-0.22219661063889	3.76095870641838	1.34159920678262
N	1.58933415460184	3.00157165437067	0.56920587031353
H	2.01582253139520	3.90420529398200	0.39373790490037
N	1.90084160779654	2.03921483923422	-0.44270269375383
C	3.34340113014000	1.56943366224757	-0.38263879256819
H	1.75053662364240	2.55123042049975	-1.45814856348292
C	3.59772706093740	0.68832686741692	-1.60386058758448
C	4.20214692242470	2.83414205490920	-0.49047305779306
C	3.66902150002950	0.83983736635494	0.91640390435821
O	1.26882114545888	2.81165365716726	-2.74855687359851
H	0.71007215450851	1.73016275685324	-2.41298645017888
C	0.35524917520656	3.88494847931203	-2.85444967657123
H	0.84575591031114	4.83420438104521	-2.60876603956886
H	-0.03646437108803	3.96146589774442	-3.87595602289950
H	-0.50009355102758	3.76214720118129	-2.17508114217732
H	3.89312108995063	3.45115665327889	-1.33845537514494
H	5.23757405746428	2.53012929415684	-0.64846165841235
H	4.16606900291840	3.42963004953938	0.42378299839027
H	3.27087345641185	1.37356522457578	1.78040050419117
H	4.75567093498346	0.79764205215296	1.01058721194142
H	3.30265722614861	-0.18506413459456	0.92523746280018
H	2.97155770705239	-0.20140980448336	-1.60496286465687
H	4.64342874494782	0.37683803361819	-1.57578055469907
H	3.42624006037785	1.24325492493789	-2.52799362911769

O	-4.49724334367459	-4.17230866669291	0.58248011426245
O	-3.84261672515746	-4.12677700763028	-1.45953265445135

P-IIA

C	-2.65170061389282	0.14336232757838	0.38408821307330
C	-1.54158240711519	0.36956220742322	1.18512513399055
C	-0.41441476344672	0.98418772312424	0.65547118920628
C	-0.38983488256581	1.40290749601592	-0.66999320189989
C	-1.49292102160626	1.13842037775963	-1.47789942140232
C	-2.61792221086676	0.51796487410943	-0.95492032647760
H	-1.54391601680494	0.05658011482323	2.22337228614710
H	0.44883994889282	1.14356081623106	1.28947404970619
H	-1.49031987900945	1.43075789844839	-2.52045558013311
H	-3.47318116133625	0.32937380009536	-1.59472687043243
H	-3.53254241008691	-0.33864727997825	0.79420717223675
C	0.81804185553954	2.12859212902612	-1.23686948439248
C	2.15162677506452	1.79669148987760	-0.59840418590764
C	2.72442125224817	0.55059887219591	-0.21277965492548
C	1.96719824122044	-0.75631831255626	-0.50136620913048
O	0.96730079064042	1.84913977032112	-2.62613399613906
O	3.84549467845938	0.42063785942750	0.27949889269774
O	1.63946599080726	-1.08592171758569	-1.61590865797826
O	1.84129379583558	-1.49850909837935	0.58931104179124
C	1.29814300761015	-2.83529666271716	0.41554892724564
H	1.68433768474582	-3.38983624640758	1.26997199816793
H	1.70903105554757	-3.25516615677331	-0.50272170655600
C	-0.21526874613231	-2.82090880612282	0.40001008966997
H	-0.59551872496221	-2.22240356730759	-0.42930234194299
H	-0.57779193592805	-3.84571773682352	0.28606003019063
H	-0.60845242395041	-2.41549170198393	1.33355413954059
C	2.88312454216968	2.95570342628696	-0.65118850106436
H	3.94369196419819	3.08936181141611	-0.48480523626848
N	2.13004352974356	3.98189331361610	-1.00374893629039
H	2.45391085076805	4.88418722933664	-1.30810558501819
N	0.76177373568454	3.63984606363426	-1.13281039378106
C	-0.04139826916569	4.42921726940356	-0.12211945424878

H	0.06747304634132	4.26140899211295	-2.84018454804406
C	0.19327245938283	5.90802562144274	-0.47386292314854
C	-1.53729352094678	4.15460074572997	-0.29428637619915
C	0.39374320434412	4.19890804981962	1.32565007883911
H	-0.04483946970040	6.10686795300050	-1.52160906959998
H	1.21746862197084	6.22651233546270	-0.27255661586958
H	-0.46164168214802	6.52037773998545	0.14767288517254
H	1.46703279373146	4.36062286111628	1.44431030991617
H	-0.12571505218108	4.91641654667711	1.96534646325160
H	0.14519269687989	3.19937965453313	1.67422153960900
H	-1.84847526672868	3.21544331135082	0.15548398064915
H	-1.81778939260357	4.15053262011912	-1.35000148397224
H	-2.08596342311138	4.95898031553064	0.19986623844164
O	-0.16965269177559	4.34157486622838	-3.78347682209491
C	0.99045507279539	4.79579692007639	-4.47449927222174
H	0.32325610695011	2.37213141807408	-3.12576833463517
H	0.74726055363668	4.84166120127448	-5.53704841404126
H	1.28814662603542	5.79529273995535	-4.13824896214335
H	1.83289508482149	4.10933652399477	-4.33494209358406

P-IIB

C	-3.48706428650997	-2.29325472647284	-0.26356491452725
C	-3.48058466316616	-1.76357198492648	1.02360406250458
C	-2.88961514128649	-0.53247976296913	1.26197969513761
C	-2.27599318301448	0.16820946175430	0.22252689439855
C	-2.28065907640699	-0.36584114333020	-1.06208264903313
C	-2.89540939522711	-1.59001341961701	-1.30398669916185
H	-3.93954064679205	-2.31032217668599	1.84021143106305
H	-2.89130182448181	-0.10766498698797	2.25964148512181
H	-1.81675349033657	0.17620036430744	-1.87379669364639
H	-2.90820414796989	-1.99398211050209	-2.31054218170422
H	-3.95468768816527	-3.25353559650148	-0.45323066472359
C	-1.70612937179070	1.52014003814063	0.54106895638733
C	-0.33792358424266	1.86693126745422	0.18008185839856
C	0.73989364304399	1.01094226023020	-0.46617235852243
C	0.90831785429296	-0.27323299915495	0.39364308367035

O	-2.40803658836648	2.31140186988082	1.16872938838125
O	0.50166143866853	0.60551012499320	-1.78707721626972
O	0.76128760248573	-0.24514792016478	1.59257827665810
O	1.21680541716069	-1.35542893589465	-0.30154195914180
C	1.38268679973310	-2.59107124983783	0.44213683262829
H	1.90371642725926	-2.36997349150670	1.37405329094867
H	2.02353981552026	-3.19887099562173	-0.19556912271691
C	0.04379811377940	-3.25458412768395	0.68473909816313
H	-0.59929454253321	-2.62588795239664	1.30181943048642
H	-0.46473336995719	-3.45177984447765	-0.26102977010569
H	0.20165934335764	-4.20530873519514	1.19995352859779
C	0.25185523766196	2.98298932901941	0.66733612467642
H	-0.21437840719348	3.81006940415424	1.18436740803510
N	1.58572325904542	2.98361557676638	0.47937769209080
H	2.03924116606623	3.86504585005595	0.27455740484754
N	1.90458304437706	1.93880150797023	-0.43670216980468
C	3.32618928937495	1.49970580232832	-0.35723876399474
H	1.60260923272792	2.96066078587544	-2.21990176250795
C	3.60703939428536	0.57399153613531	-1.54105734089534
C	4.18724841914660	2.75915894620919	-0.52302951960603
C	3.69974020637473	0.82212189598873	0.96596060512600
O	1.06304165631823	3.09508926349028	-3.01712247652785
H	0.45857615228303	1.41242954567210	-2.33144388099364
C	0.25755368766593	4.25946175084609	-2.83313147981415
H	0.87823900959656	5.14762373846434	-2.67976858859178
H	-0.33355522170920	4.39311099607437	-3.73966882624412
H	-0.42098286291758	4.14323933720800	-1.98183759136847
H	3.87189570849024	3.33696881485673	-1.39627057519002
H	5.22590672357854	2.45765676545902	-0.66806518941002
H	4.15156263444289	3.39888980688959	0.36173709436435
H	3.32028416014560	1.39439861646036	1.81495263473923
H	4.78841479438775	0.77575520779151	1.04228910139637
H	3.32557465216891	-0.19898727694214	1.03275715393970
H	3.00218807334395	-0.33003092143849	-1.50769487559961
H	4.66016256004561	0.28588212513743	-1.51074824879284
H	3.41325197523823	1.08856836869392	-2.48492701286678

P-IIA-NO_2

C	-2.77925544584589	-0.70175708678460	2.09344960764752
C	-1.77033122935089	0.05950706572190	2.66055471304397
C	-0.75797281085458	0.52431285511075	1.84020055488900
C	-0.75932945468457	0.24578232516112	0.47536952526195
C	-1.77853738803256	-0.53323051510940	-0.05971157947455
C	-2.79936726943914	-1.01323056655037	0.74522010576596
H	-1.77732461564942	0.27915193485888	3.71914974389187
H	0.03734968555770	1.11605150996303	2.27474511407460
H	-1.77224341123988	-0.77889208273212	-1.11189777050026
H	-3.59299916444758	-1.62227062204923	0.33483938881723
C	0.36972607800733	0.80002285380152	-0.39935941006452
C	1.69758923244362	0.76374234907533	0.34034685635882
C	2.49222122973542	-0.38536086518794	0.65268127849478
C	2.12920172418721	-1.74088570890647	0.03234274314303
O	0.36910947294361	0.05701788756670	-1.59188443357904
O	3.50887147564152	-0.36992344249837	1.33959546057723
O	2.77173723483636	-2.22116178604689	-0.86994545320103
O	1.12296239739159	-2.32164434356192	0.66643177706560
C	0.78113616715523	-3.67781070382881	0.26167229277384
H	0.26920788005882	-4.08848786454196	1.13074720132796
H	1.70852600641331	-4.22621567344364	0.09518657026759
C	-0.10628844744010	-3.68762394404697	-0.96456500139948
H	0.39287618586285	-3.22180177875164	-1.81539338035445
H	-0.33735750889097	-4.72421760176072	-1.22265616672710
H	-1.04282103018400	-3.16378230259182	-0.77006990614563
C	2.04517264022823	2.03520242582735	0.66863493747028
H	2.92764067857512	2.37915205874065	1.19056201172981
N	1.13525514020596	2.91664313451755	0.23520285994014
H	1.43472259214932	3.84426598756668	-0.03185165161161
N	0.27730190123381	2.26555445497417	-0.69977325405296
C	-0.99217875213058	3.00469239607419	-0.94446664518047
H	1.49711061774008	2.33549694900475	-2.38280473193020
C	-0.59547524451874	4.40969126102102	-1.42228837076557

C	-1.73153505025404	2.31241618822248	-2.08802983185252
C	-1.90198485665562	3.14609594341631	0.28220217577954
O	2.08540752303579	1.82981671927687	-2.96878987506116
C	1.82152839900204	2.20309329872974	-4.32065577819514
H	1.03690739415947	0.45286321998506	-2.18228113661488
N	-3.85955869928352	-1.20555926437364	2.95608541514740
H	0.78402427529823	1.99023521782131	-4.59857745565499
H	2.02443036562489	3.26626066046305	-4.48266214761844
H	2.48516232241670	1.61548181157934	-4.95576727885719
H	-2.05040441798276	1.30694878163933	-1.82290525543704
H	-1.10157670693318	2.25543151929771	-2.97779829694884
H	-2.62187171033643	2.89791292399509	-2.32714275639939
H	-2.42323683881003	2.22027773764748	0.51959923116536
H	-2.65384165843002	3.91035586789870	0.07166508166512
H	-1.32940409533532	3.46367945782367	1.15651946342827
H	-0.19805261092410	5.02505589019410	-0.61111074311149
H	-1.48381249442181	4.91521925915720	-1.80443334083359
H	0.14344631068101	4.35577452167516	-2.22637776839771
O	-4.72770722555201	-1.88868438879361	2.44806623032874
O	-3.83335679295745	-0.91406592624809	4.13622907991365

P-IIB-NO_2

C	-3.36687472327371	-2.29730516575879	-0.18849971587741
C	-3.39096033976413	-1.76589905238302	1.09177410296320
C	-2.83502432624101	-0.51538757819826	1.29112420432660
C	-2.24919258501044	0.17583863675468	0.23034369058072
C	-2.24427613193072	-0.37957833339813	-1.04448245319988
C	-2.81405109612975	-1.62589267647155	-1.26327820179251
H	-3.83101737130480	-2.32110746756880	1.90841063264321
H	-2.84333650650545	-0.07148422461783	2.27926661922616
H	-1.79893053093131	0.15649470245611	-1.86959873163783
H	-2.82276182910153	-2.06862118706564	-2.24958510266838
N	-3.94121361657382	-3.63547912966605	-0.40923633203010
C	-1.70037611610182	1.54792440684793	0.52160282655299
C	-0.32843095971252	1.88165655054318	0.19377172689520

C	0.74819605909138	1.02563431905727	-0.45462062837838
C	0.90533026308003	-0.27349196815997	0.38604014891537
O	-2.43834717638019	2.34483552355311	1.09701999626789
O	0.51047377665659	0.64257370144701	-1.78221073290945
O	0.74004824841902	-0.26655899300704	1.58304435899817
O	1.22638775902611	-1.34262584398971	-0.32315467615746
C	1.40039179426239	-2.58869698111564	0.40146257902452
H	1.91276242451525	-2.37697517156700	1.34033123982367
H	2.05222153209423	-3.17854294406106	-0.24185664634304
C	0.06880925634781	-3.27298255399615	0.62587123196039
H	-0.58204707076521	-2.66445872899249	1.25481451210706
H	-0.43331109466214	-3.46041818177882	-0.32544973762351
H	0.23719099289147	-4.23104971178497	1.12370853219728
C	0.26484520418920	2.99297520414410	0.69607617689205
H	-0.19898009597766	3.82060741850047	1.21445085149468
N	1.59471090434033	2.98138034279236	0.51608199716114
H	2.07341933191397	3.85681235048168	0.34928841007240
N	1.91691621915053	1.94421892840620	-0.40405884912714
C	3.33572556175697	1.49918491212360	-0.32417308326806
H	1.61789147054703	3.06877982043005	-2.25905364126257
C	3.61994665610709	0.59831843245773	-1.52620176534124
C	4.20449030055703	2.75715746483132	-0.45708987157866
C	3.69738861316269	0.79208221884207	0.98711477073120
O	1.05394457603587	3.08829356269444	-3.04713424165678
H	0.49755935165290	1.46033869400614	-2.31519619694043
C	0.18842588383396	4.22192862453072	-2.96131227715007
H	0.76144550391395	5.15388880095299	-2.95551500663224
H	-0.45433967876375	4.20568312829682	-3.84180575426390
H	-0.43658071799731	4.17625868121404	-2.06372023310401
H	3.89716193408734	3.35593106456466	-1.31891234730994
H	5.24202723883546	2.45261566973329	-0.60360680330678
H	4.16907061882526	3.37729542965060	0.44162561178730
H	3.31650474009019	1.34932676362545	1.84555407437941
H	4.78519046902254	0.73668443397952	1.06931009051238
H	3.31646537716757	-0.22772449872567	1.03040583044623
H	3.00904430215340	-0.30220572478845	-1.51800492298959

H	4.67099348661251	0.30274699614117	-1.49493386027775
H	3.43630170875665	1.13608777702330	-2.45910559635139
O	-4.44333183575091	-4.20581160836099	0.53953201173703
O	-3.88249775621860	-4.10665683462600	-1.52822881851773
